# Metaphylogenetic analysis of global sewage reveals that bacterial strains associated with human disease show less degree of geographic clustering

**DOI:** 10.1038/s41598-020-59292-w

**Published:** 2020-02-20

**Authors:** Johanne Ahrenfeldt, Madina Waisi, Isabella C. Loft, Philip T. L. C. Clausen, Rosa Allesøe, Judit Szarvas, Rene S. Hendriksen, Frank M. Aarestrup, Ole Lund

**Affiliations:** DTU Food. Technical University of Denmark, Kongens Lyngby, Denmark 2800 Danmark

**Keywords:** Phylogeny, Phylogenetics, Bacterial infection

## Abstract

Knowledge about the difference in the global distribution of pathogens and non-pathogens is limited. Here, we investigate it using a multi-sample metagenomics phylogeny approach based on short-read metagenomic sequencing of sewage from 79 sites around the world. For each metagenomic sample, bacterial template genomes were identified in a non-redundant database of whole genome sequences. Reads were mapped to the templates identified in each sample. Phylogenetic trees were constructed for each template identified in multiple samples. The countries from which the samples were taken were grouped according to different definitions of world regions. For each tree, the tendency for regional clustering was determined. Phylogenetic trees representing 95 unique bacterial templates were created covering 4 to 71 samples. Varying degrees of regional clustering could be observed. The clustering was most pronounced for environmental bacterial species and human commensals, and less for colonizing opportunistic pathogens, opportunistic pathogens and pathogens. No pattern of significant difference in clustering between any of the organism classifications and country groupings according to income were observed. Our study suggests that while the same bacterial species might be found globally, there is a geographical regional selection or barrier to spread for individual clones of environmental and human commensal bacteria, whereas this is to a lesser degree the case for strains and clones of human pathogens and opportunistic pathogens.

## Introduction

One of the basic dogma in microbiology has for almost a century been that we for microorganisms consider that “everything is everywhere but the environment selects”^1,2^.

A large number of papers about the global transmission events of bacterial clones have been published, including descriptions of emergence and spread of specific clones of *Vibrio cholera*, MRSA, *Escherichia coli*, *Clostridium difficile*^3–6^ and many other bacterial pathogens. The main focus has been on pathogenic clones and virtually nothing is known about the global phylogeny of commensal species and clones.

The gut microbiota has so far mainly been studied in relation to diet, use of medication and diseases^7,8^, mostly within countries^9^ and in some studies between countries^10,11^. These studies have looked at the species or genera composition of the microbiota and the interaction between species, while virtually no details on within species phylogeny have been investigated.

The same has been the case for environmental bacteria; there are numerous projects, which have sequenced the metagenome of different niches^12^, but not with much focus on the importance of geographical locations or within species phylogeny.

Almost all studies into the within species phylogeny of bacterial species have been conducted using whole genome sequencing of single cultivated isolates. A recent metagenomic study where DNA was isolated both directly from faeces and from isolates cultured from the faeces, demonstrated that most pairs of isolates and metagenomic samples were adjacent to each other in a phylogenetic tree^13^.

Obtaining biological samples from global sources can be logistically difficult and further complicated by ethical constraints if the samples originate from humans. Recently, we have collected untreated human urban sewage and then conducted metagenomic sequencing as a proof of concept in establishing a global monitoring of antimicrobial resistance. Untreated, as described in the original study, means that it has not run through a sewage treatment plant, but the samples were taken from the inlet into the plants or in countries/regions where sewage is not treated, it was taken from where the sewage runs into the environment. 79 locations in 60 countries were sampled (see Supplementary Table S1 for a complete list of countries and regions)^14^.

The advantage of metagenomics is the feasibility to detect all genes related to all living organisms present in the sample analysed. Thus, the metagenomics approach has the potential to determine phylogenies among all bacterial species present in urban sewage and enable the study of real-time occurrences and transmissions across all bacterial species to detect changes attributed to climate, trade and travel.

Here, we present a bioinformatic pipeline able to construct meta-phylogenetic trees based on multi-metagenomic samples. The reference genomes representing the bacteria in urban sewage samples were determined and the reads were mapped to the identified bacterial reference genomes. The genetic single nucleotide polymorphism (SNP) distance between each sample containing genetic material similar to a given reference sequence was calculated and trees inferred from the distance matrices. It was investigated if different groups of bacteria showed different clustering patterns.

## Results

### Generation of bacterial reference genome database

A database of bacterial species was generated from NCBI (see methods), in which redundancies, very similar bacterial reference genomes, were removed by homology reduction using the Hobohm 1 algorithm^15^. Out of 6,510 complete bacterial genomes downloaded from NCBI, 3,721 were left after the homology reduction. The taxonomic composition of the database includes 34 phyla, 64 orders, 138 classes, 301 families, 843 genera, 2,319 species and 3,721 unique bacterial templates. The most abundant genera are *Burkholderia*, *Pseudomonas*, *Bacillus* and *Vibrio*. 467 of the genera contained only a single unique bacterial template. Out of the 2,319 species the most abundant were *Helicobacter pylori*, *Salmonella enterica* and *Escherichia coli* (see Supplementary Table S2).

### Bacterial template identification

A total of 15,513 hits to bacterial templates were identified in the 79 sewage samples (step A. All steps shown in the methods section); these belonged to 996 unique bacterial reference templates. In the pre-processing (step B) all hits with a depth below 1 were discarded, leaving 11,691, belonging to 834 unique bacterial templates. Mapping was done on these 11,691 bacterial templates to create 11,691 consensus sequences (step C). After the distance calculation pre-processing (step D), where only consensus sequences with less than 40% unknown bases were kept, there was 1,504 left. These belonged to 115 unique bacterial templates. The distance calculation (step E) was run for each of these 115 unique bacterial templates, resulting in 115 distance matrices. The phylogeny was inferred (step F) on all the distance matrices where the bacterial template was observed in more than three samples, resulting in a total of 95 phylogenetic trees. An overview of the pipeline results after each step can be seen in Supplementary Fig. S2. Supplementary Table S3 provides an overview of the taxonomic composition of the remaining bacterial templates after the various steps in the pipeline.

**Figure 1:**
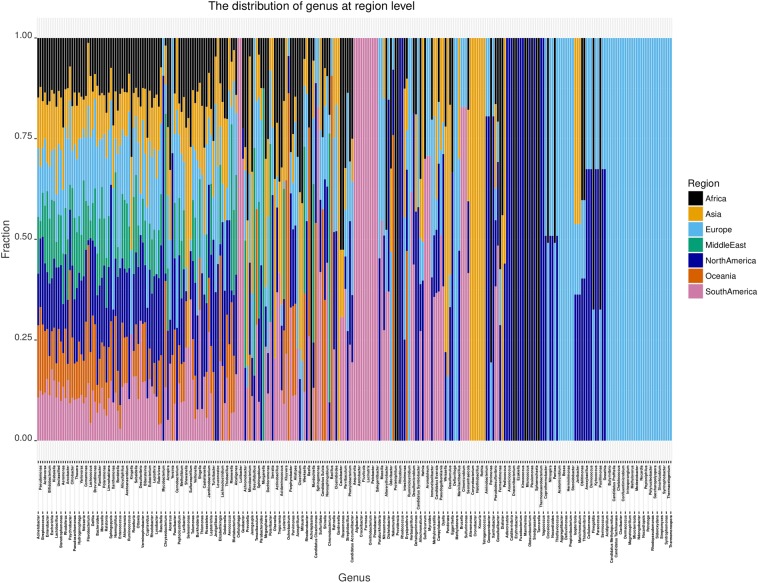
The distribution of identified genera in the different regions. The data have been standardized according to the number of samples in each region and the abundance for each genus has been calculated. The genera are sorted by abundance from high to low.

The fraction of bacterial reference template hits for each genus in each region is shown in Fig. 1. A total of 279 different genera were identified with a depth above one and have been plotted and sorted according to the total abundance. Fifty-five genera as well as an unclassified group are all present in all regions, and 64 genera were only identified in one region. The most abundant genera and present in all regions are *Acinetobacter, Pseudomonas, Streptococcus, Acidovorax, Enterobacter, Bifidobacterium, Escherichia, Klebsiella, and Lactobacillus*.

**Figure 2:**
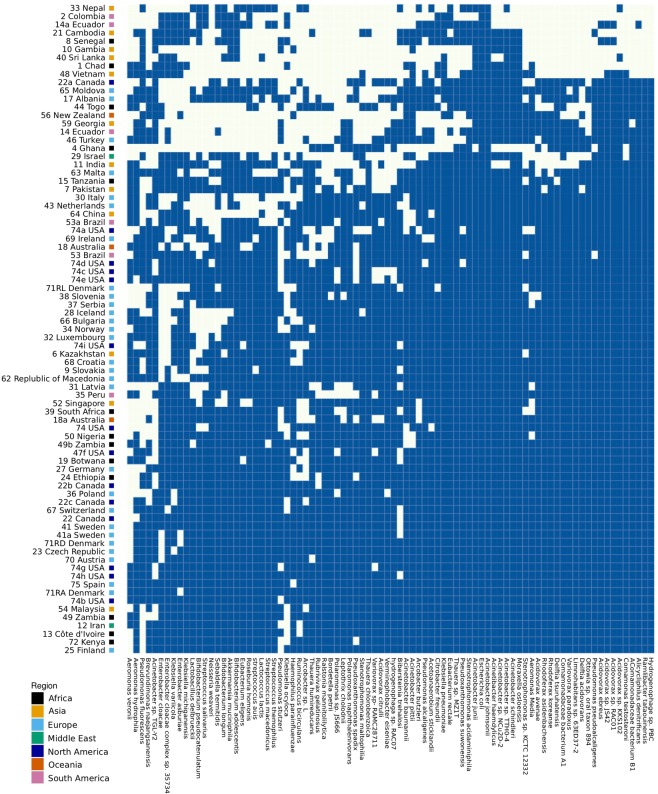
Heatmap illustrating the occurrence of the 80 most abundant species identified in the global sewage samples. A dark blue square indicates the presence of the given bacterial species in the given sample.

To provide an overview of the species that are the most common in the urban sewage samples, a count matrix with the presence/absence of each species in each sample was created. The sample counts were aggregated, and the 80 most frequently occurring species are shown in a heatmap (Fig. 2). The urban sewage samples had a high occurrence of the species *Escherichia coli*, *Streptococcus suis* and *Pseudomonas fluorescens*.

### Phylogenetic trees

**Figure 3:**
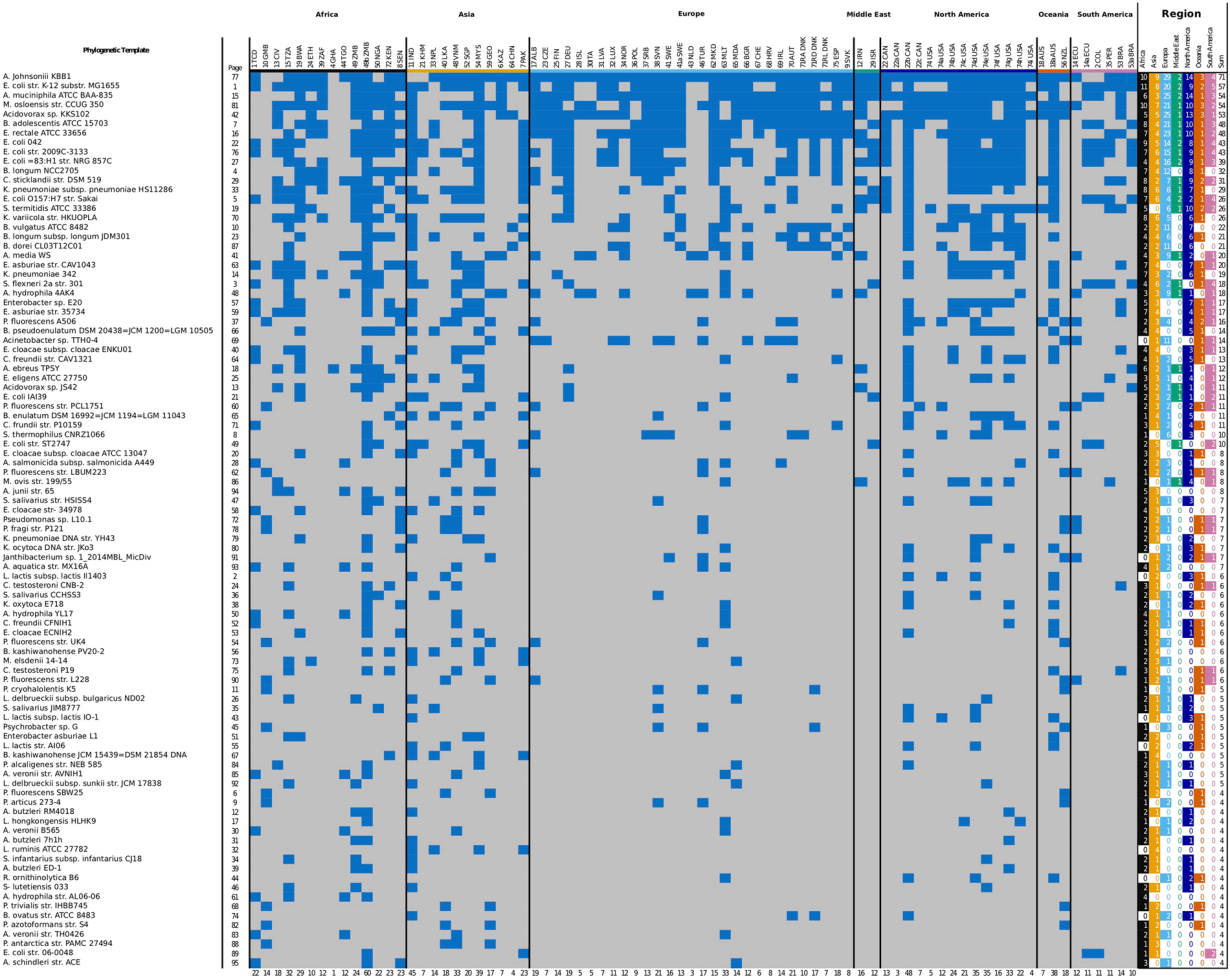
A presence/absence map of the samples in the 95 phylogenetic trees. Blue is presence, grey is absence. The samples are sorted by region according to continent and the trees by number of taxa. Page refers to the page number in the collated pdf file of all the phylogenetic trees. Each region has a column, if a region is represented in a phylogeny, it is marked by a coloured field. The number of samples per tree is summed in the last column, and the number of phylogenies that each sample appear in is summed in the last row.

Of the original 996 unique bacterial templates identified in the pre-processing step, B, the final output of the MetaPhylogeny pipeline, applied to the 79 urban sewage samples, was 95 phylogenetic trees. (Supplementary Dataset 1). In Fig. 3 the presence/absence of the samples in each tree is shown together with the total number of taxa in each tree and the total number of occurrences per sample.

### Distance-based clustering

The distance matrices for each template were tested for clustering by comparing distances within country-groupings (intra-regional distances) to distances between country-groupings (inter-regional distances). This was conducted for all distance matrices containing more than one region and at least four samples by a modified Welch *t*-test, providing a p-value and an intra/inter-regional distance ratio. The countries were grouped according to World Bank regions (WB-R), World Bank income level (WB-IL), WHO regions (WHO-R) and WHO health impact (WHO-HI).

**Figure 4:**
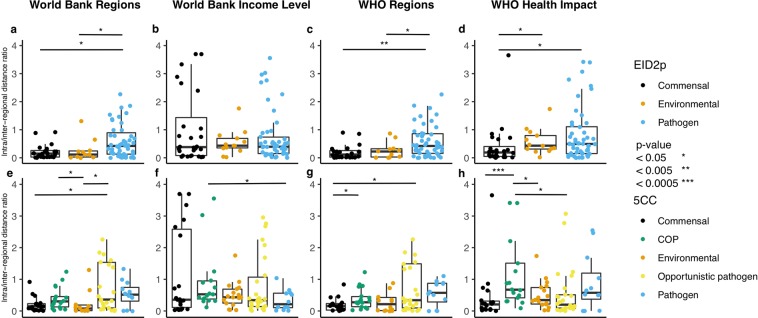
Boxplot showing the overall distribution of intra/inter-regional distance ratios for each organism group, with dots representing a single template organism. The top row (**a**–**d**) shows organism classification according to EID2p. The bottom row (**e**–**h**) shows organism classification according to 5CC. Significantly different clustering is marked with asterisk(s). Ratios above 4 not shown, the numbers are WB-R 4, WB-IL 1, WHO-R 4 and WHO-HI 1. COP, colonizing opportunistic pathogen.

The ratios for each organism were grouped afterwards according to organism classification, annotated by the two schemes EID2 plus (EID2p) and Five class classification (5CC) (Fig. 4). The lower the ratio, the higher degree an organism will cluster according to the specified country grouping. For each regional grouping, the difference in clustering according to the intra/inter-regional distance ratio was tested using Wilcoxon rank sum test in R^16^ comparing all organism classifications. The results for EID2p are shown in Supplementary Table S4, and for 5CC in Supplementary Table S5. The significant differences can be found as stars above the boxplot in Fig. 4.

Figure 4 shows that when using the EID2p classification of the bacteria into commensal, environmental and pathogen, the distribution of ratios in both the environmental and commensal bacteria are more clustered than the pathogens, when using the WB-R (a) and WHO-R (c) grouping of countries but not when using the WB-IL (b) and WHO-HI (d) clustering. For the EID2p based classification, there is a significant difference in the clustering of pathogens to environmental organisms and to commensal organisms for both WB-R (environmental p-value: 0.039, commensal p-value: 0.014) and WHO-R (environmental p-value: 0.039, commensal p-value: 0.0039), where the pathogenic bacteria has the least degree of regional clustering, and environmental and commensal the most. There is a significant difference in the clustering of commensal compared to both pathogens and environmental organism in the WHO-HI grouping (pathogen p-value: 0.022, environmental p-value: 0.029) where the commensal group is more regionally clustered and the others. No significant differences were observed for the WB-IL country grouping.

When the 5CC classification of bacteria into five categories was used (Fig. 4, bottom row (e-h)), there is no clear pattern of significantly different clustering, except that for both WB-R and WHO-R, the commensal group cluster significantly different from the opportunistic pathogen group (WB-R p-value: 0.026, WHO-R p-value: 0.028), where the commensal is the more regionally clustered group. When looking at the median ratio in Fig. 4, bottom row (e-h), although the difference between the groups is not significant, the tendency is the commensals and environmental have the lowest ratio, i.e. highest degree of clustering, and the three pathogen groups have a higher ratio and thereby less regional clustering. This goes for all but the WB-IL grouping.

Countries grouped together consistently according to regions, with a few exceptions. These countries can be seen in the Supplementary Tables S6 and S7. It is noteworthy to mention that all of Latin and North America are grouped together according to WHO, while the World Bank divides the region into two. Similarly, WHO groups Israel with Europe, while World Bank groups Israel with the Middle-Eastern countries. In addition, Pakistan changes from South Asia in WB-R to Eastern Mediterranean in WHO-R and Malta changes from Middle East & North Africa in WB-R to Europe in WHO-R.

**Figure 5:**
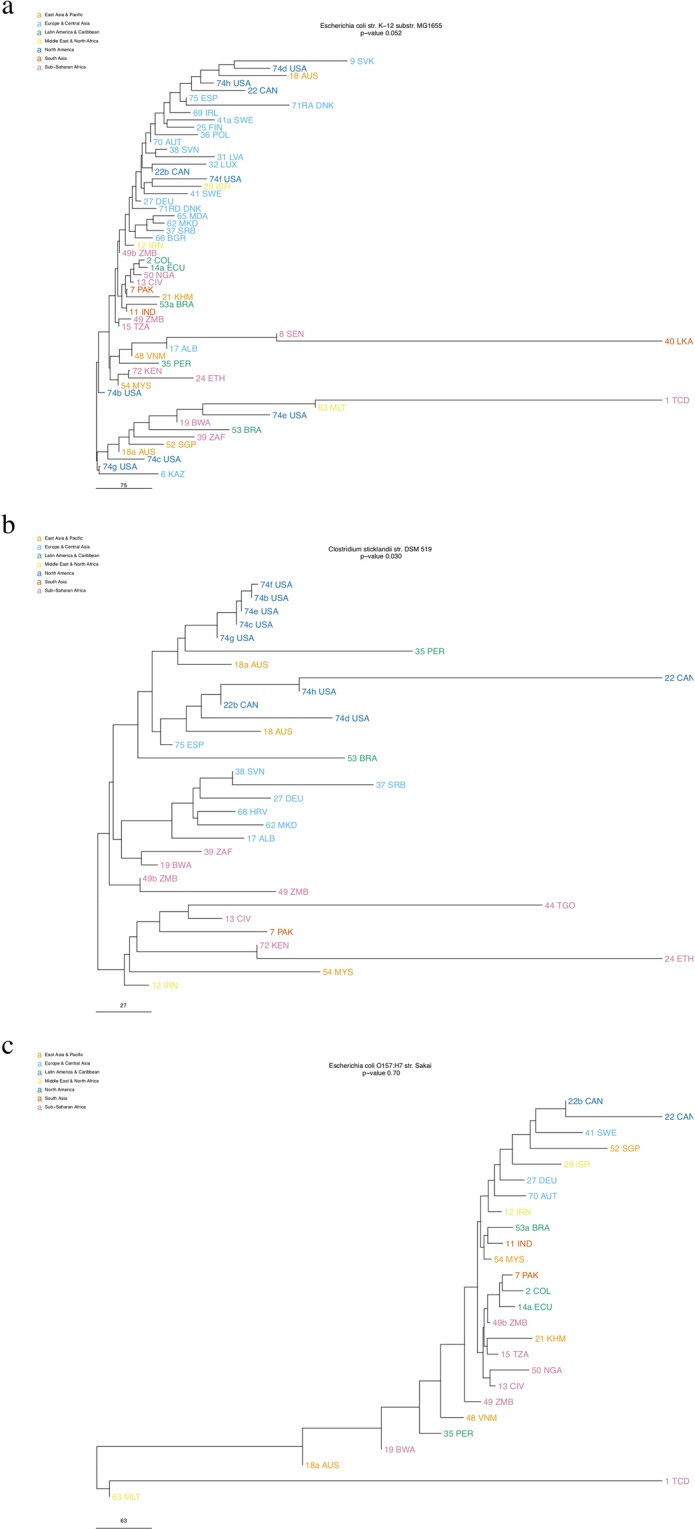
Phylogenetic tree for (**a**) the commensal *Escherichia coli* K-12 strain (**b**) the environmental *Clostridium sticklandii* (**c**) the pathogen *Escherichia coli* O157:H7 str. Sakai. The samples are coloured by World Bank regions.

To illustrate the levels of regional clustering for the classes from 5CC for the WB-R data, the phylogenetic trees representing the strain from the commensal, environmental and pathogenic classes, with the p-values (from the distance-based Welch *t*-test) closest to the median is shown in Fig. 5a–c. If the median was between two trees, the tree with the most samples was chosen. The median p-values are: commensal 0.0346 (Fig. 5a), environmental 0.0303 (Fig. 5b) and pathogen 0.605 (Fig. 5c).

## Discussion

We found a large variety of different bacterial genera in the samples, as in Hendriksen *et al*., the first study analysing the worldwide sewage samples. Compared with the original study, we identified fewer bacterial genera (279 compared with 1,546) in this study, as we were only looking for whole genomes and had a higher threshold for detection^14^. We found that the most frequently observed genera in the 79 samples were genera that have previously been commonly observed in fecal (*Streptococcus, Enterobacter, Bifidobacterium*, *Escherichia, Lactobacillus, Klebsiella*)^17,18^ and environmental samples (*Acinetobacter*, *Pseudomonas, Acidovorax*)^19–21^, and only *Enterobacter* and *Lactobacillus* among the top 10 were not present in the top 15 most abundant genera from the original study of this data^14^.

Thompson *et al*. found that the most abundant or prevalent genera in the Earth Microbiome Project, based on the number of times a tag sequence representing the genera was found in the Greengenes database, are the following: *Bacillus, Bradyrhizobium, Arthrobacter, Streptococcus, Pseudomonas, Rhodoplanes, Sphingomonas* and *Kingella*^12^. In our study, *Streptococcus* and *Pseudomonas* were among the top 10 most frequently observed genera and found in all regions, *Kingella* was found in all regions but North America, *Bacillus* was found in Oceania and Europe and *Sphingomonas* was found in Europe and South America. Further studies are needed to verify whether this is a consistent pattern or potential bias in the study. We have identified at least two biases that can affect this conclusion. Firstly, in-sufficient sequencing depth in our study can affect the ability to detect rare species. Secondly, as we only have 79 samples across the world, we cannot be certain that we sequence everything.

In the mapping step of this pipeline, all of the raw reads from a sample are mapped to each of the identified template genomes for this sample, to create the consensus sequences, which we use for distance calculation. This induces the risk that reads are mapped incorrectly, i.e. for areas on the template genomes where there is a close resemblance to other genomes, we risk mapping wrong reads to that part and hereby create a false “core” genome. This could affect the distance calculation, as the areas where wrong reads are mapped could be filled with either wrong bases or will be blurred by the noise from erroneously aligned bases and therefore marked as ambiguous. However, due to the strictness of our thresholds for mapping the reads, we believe that if any of these scenarios are true, it is the latter; that some mutations may not be found due to noise in the consensus sequence. The mapping algorithm requires an ungapped alignment with a score of 50, with a match score of 1 and penalty for mismatch at −3. Our base-calling method only calls the base if the most frequently found base at the given position is found at a significance level of 0.001^22^. Furthermore, this method of creating a consensus sequence from metagenomic raw reads was tested by Joensen *et al*. 2017, in a study where both metagenomic raw reads and raw reads from individual isolated colonies from the same faecal samples were mapped to the same reference genome and the phylogeny was inferred by the use of NDtree. They found that in most cases, the metagenomic and the isolated sequences from the same samples were placed together in the tree^13^.

When we create the database of template sequences, used both in identifying the species in each sample and later to map the raw reads to, we only use the NCBI RefSeq.^23^ genomes. This can and does lead to a bias in the species we find, as we can only look for the well-studied organisms that are in this curated database. But we found it to be of greater importance to use well-described genomes, and perhaps identify fewer species, than to use genomes where we are not as certain of their origin and how well assembled they are.

In the step prior to the distance calculation (step D), we discard consensus sequences with more than 40% unknown bases. This gave us 1,504 consensus sequences. We initially set the thresholds at both 20% and 30%, which resulted in 518 and 1,023 consensus sequences, respectively. We deemed both numbers to be too small to give us enough trees. When we looked at the percentages of unknown bases over all the 11,691 consensus sequences, we saw that the majority of the sequences contained 75%-90% unknown bases (see Supplementary Figure S2). We could have included more sequences, but if the cutoff had been at 50%, we could have had templates where no phylogenetic distance could be calculated, because none of the positions were covered in each template, by only having two “bad sequences”.

We chose to use Neighbor-Joining (NJ) to infer the phylogeny, as we have shown in previous studies, that when using a distance-based approach for phylogeny, the NJ algorithm performs as well as Maximum Likelihood-based approaches^13,22,24–26^. Furthermore, the speed gained by Neighbor-Joining is significant.

Phylogenetic trees representing unique bacterial templates in the samples were obtained from 79 sewage samples. For each tree, the tendency to regional clustering was found by calculating the statistical significance of the average genetic distance between samples from the same region versus samples from different regions. The environmental samples clustered significantly more than the pathogenic bacteria using the EID2p classification of the bacteria (commensal, environmental, pathogen), when using the WB-R or the WHO-R classification of the countries of the world. The 5CC classification expanded the bacterial classification with the following categories: opportunistic pathogens and colonizing opportunistic pathogens (COP). Using these classifications, the differences between environmental and pathogenic bacteria were still significant. Significant differences could be observed between both commensal and environmental bacteria as compared with pathogens as well as opportunistic pathogens for either the WB and WHO regions. Furthermore, it could be observed that COPs were less regionally clustered than the environmental, however, this difference was only statistically significant for the WB regions and for WHO health impact. In general, the results were comparable using the two different definitions of regions, at tendency if not significance level.

Commensal and environmental bacteria displayed the highest level of regional clustering. Commensal bacteria were in general more significantly different than environmental bacteria from both pathogens and opportunistic pathogens. COPs showed a tendency to be less regionally clustered than commensals and environmental bacteria, but more than the pathogens and opportunistic pathogens for the two regional groupings (the only statistically significant difference was to the environmental using the WB regions). We only found significant differences in clustering between two groups (COP and Pathogen) of the microorganism classifications and country groupings according to income (WB-IL). This could indicate that bacteria are spread, or are selected globally, dependent of geographic distances but independent of income status.

The bacteria were fairly equally divided in the different categories. When dividing the species by the 5CC classification, the average number of samples per distance matrix are the following: Commensal 18.4, COP 12.3, Opportunistic pathogen 10.9, Pathogen 17.5, and Environmental 13.1.

Some microorganisms in each classification have a ratio that is noticeably different from the median. Three *Klebsiella genomes* (two classified as COPs, one as commensal) and one *Acinetobacter* genome (classified as an environmental strain) have high ratios, indicating low regional clustering. These four genomes behaved more like pathogens. Two *Areomonas* genomes and one *Enterobacter* genome classified as opportunistic pathogens have a low degree of regional clustering with very high ratios (>8 for the two *Areomonas* and 2.26 for the *Enterobacter*), even for this group, where the median is 0.37. The annotation by the 5CC is ambiguous for these three strains. The first is an industrial strain as well as a zoonotic pathogen, the second is a fish pathogen and possibly also commensal bacterium, and the third could also be classified as environmental bacterium. Three *E. coli* classified as pathogens also stand out as they all have a high level of regional clustering. The first is an AIEC (Adherent-invasive *Escherichia coli*), this *E. coli* pathotype is related to Crohn’s Disease where it colonizes the ileum of the patient^27,28^, and although it is a pathogen, the colonization may still add to the regional clustering of this particular type, as the bacteria will develop together with the infected host. The second is a uropathogenic ExPEC (Extra-intestinal pathogenic *Escherichia coli*)^29^, which may be classified as a COP rather than a pathogen. The last one is an *E. coli* with the O157:H7 serotype, which most likely is a pathogen.

Although we see a pattern of clustering, not all species follow this pattern. Even some strains of the same species, with the same serotype, such as the two *E. coli* O157:H7 strains, do not have the same pattern, one has a ratio of 1.33 (WB-R) or 1.10 (WHO-R) (not regionally clustered), the other 0.48 (WB-R) or 0.38 (WHO-R) (regionally clustered). However, these two particular strains support that the mapping method we use is specific, since we get different clustering for two closely related strains.

Most global phylogenetic or phylogenomic studies have been conducted on pathogenic bacterial species or clonal sub-groups, and it has been shown for several species that such pathogenic clones can spread worldwide. This includes: *Shigella*^30^, *Staphylococcus aureus*, *Klebsiella*^31^, *Streptococcus agalactiae*^32^, *E. coli*^33,34^ and *Acinetobacter baumannii*^35^. Such studies have very rarely been conducted on colonizing or environmental bacterial species. However, studies have suggested that the fruit pathogen *Pseudomonas syringae pv. acitinidiae*^36,37^ and the environmental *Streptococcus thermophuilis*^38^ have regional phylogenies. These results are in line with the findings of this study.

## Conclusion

In general, we find that for environmental and commensal bacteria in particular, and to a lesser extent for COPs, there is a selection or barriers to spread based on geographical regions. For pathogens and opportunistic pathogens less regional clustering is observed. Income level and health impact were less correlated with the spread of the bacteria than geography-based clustering.

## Methods

### Pipeline

**Figure 6:**
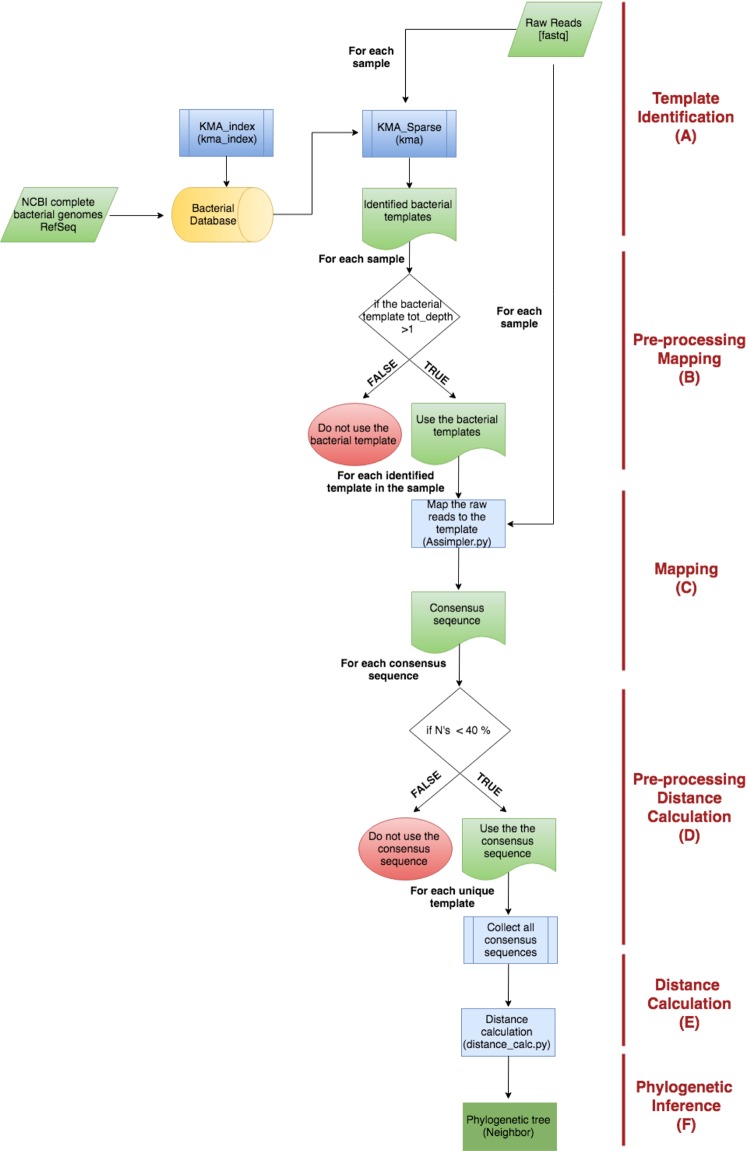
The workflow of the MetaPhylogeny pipeline for bacterial phylogeny. Blue boxes indicate algorithms and scripts used in the pipeline. Green boxes are input and output files. The red spheres illustrate discarded templates. The yellow cylinder is the database.

The workflow of the pipeline is depicted in Fig. 6 and consists of a template database creation step and six data analysis steps (A-F), including four major steps and two minor pre-processing steps.

#### Reference template database

The pipeline requires a database of unique bacterial reference template genomes. This database is both used for identification of templates in the metagenome samples and as template sequences to map the reads against in step C. This database was created by downloading all complete bacterial genomes from the NCBI RefSeq database^23^. A Hobohm 1^15^ homology reduction with a similarity threshold of 98% was performed on the database. This was done by kma_index^39^, which, in addition to homology reduction, also created a database of unique 20-mers with the prefix “ATGAC” for each genome^40^. The genomes in the homology-reduced database are representative strain templates and here referred to as unique bacterial templates.

(A) **Template identification:** The first analytic step in the pipeline is to identify all bacterial templates present in each of the metagenome samples with *KMA*^39^. *KMA* was used with its sparse mapping option and the “winner takes all” scoring method.
(B) **Mapping pre-processing:** After the bacterial template identification, the results from *KMA* were sorted on the total mapping depth, i.e. how many times the bacterial template had been covered by the raw reads. The cut-off was set to 1.
(C) **Mapping:** After discarding the bacterial templates with low depth, the raw reads for each sample were mapped to each of the remaining bacterial templates identified in that sample. This was done using the *Assimpler* mapping tool, which is also employed in *NDtree* (https://cge.cbs.dtu.dk/services/NDtree/), with default settings. *NDtree* has been validated in a number of studies^13,22,24–26^. This step yields consensus sequences for each bacterial template.
(D) **Distance calculation pre-processing:** This pre-processing step checks the consensus sequences for their content of unknown bases. If a consensus sequence contains less than 40% unknown bases, they will be used for distance matrix calculation, otherwise they are discarded.
(E) **Distance calculation:** For each unique bacterial template, the genetic distance is calculated between all the consensus sequences for the bacterial templates that passed the pre-processing step. The distance calculation is done by the same distance algorithm, which is employed by *NDtree*, with the use of the “all called” option that only uses the positions that are known in all genomes for the distance calculation. The output from this step is a distance matrix for each of the remaining unique bacterial templates.
(F) **Phylogenetic inference:** The phylogeny was inferred by the Neighbor-Joining algorithm^41^ on each of the distance matrices from the previous step, if there were more than three samples participating in the matrix. The phylogenetic inference was done by the program *Neighbor* from the Phylip package^42^.

### Statistical analysis

For the statistical analysis a Distance-based multivariate Welch *t*-test based on Alekseyenko’s multivariate Welch *t*-test on distances^43^, modified to test the difference between distances within groups (intra-regional) compared with distances between groups (inter-regional) was developed (see bitbucket repository distance_matrix_tests.R for R script). The modified Welch *t*-test is used to calculate a value for the intra- vs inter-regional clustering. Each distance matrix was shuffled 999 times, and a p-value was calculated to assess whether the original clustering was significant compared with the randomly occurring clustering. Furthermore, the ratio of intra- vs inter-regional distances was calculated by dividing the sum of squared distances for the intra-regional cluster by the sum of squared distances for the inter-regional clusters. These ratios were used to test if there were any significant differences in regional clustering of trees with different types of organism classifications. This is performed using a Wilcoxon rank sum test^16^.

The scripts for the statistical analysis, the distance matrices from the pipeline, together with the tree files and the metadata for the statistical analysis can be found on Bitbucket. (https://bitbucket.org/genomicepidemiology/metaphylogeny_paper)

### Visualization

The visualizations of the statistical analysis and of the trees were done in R (version 3.6.0)^44^ with the packages ggplot2 (3.2.1)^45^, ggtree (1.16.6) and treeio (1.8.2)^46^. Other used packages include cowplot (1.0.0)^47^, dplyr^48^, ggbeeswarm (0.6.0)^49^ and tidyr (0.8.3)^50^.

## Materials

Regional classification was obtained from the World Bank^51^ regarding region and income. Regional classification was obtained from WHO^52^ regarding region and health impact. All regional data can be found in the supplementary information (Supplementary Table S1 and Supplementary Dataset 2).

The annotation of bacterial templates was done according to two classification schemes: EID2 plus (EID2p) and 5CC. The EID2p was built from the EID2 database^53^ and Taylor *et al*.^54^. The 5CC is based on EID2p classification, which further classified pathogens into COP (colonizing opportunistic pathogen) and OP (opportunistic pathogen) according to Price *et al*.^55^. The full list can be seen in Supplementary Dataset 3 and the workflow of the annotation is further described in the Supplementary Methods (Classifications of bacterial templates).

## Data

The data was obtained from the Global Sewage project for the first 79 samples collected^14^.

## Supplementary information

Supplementary information

Supplementary information2

Supplementary information3

Supplementary information4

## Data Availability

The scripts for the statistical analysis, the distance matrices from the pipeline, together with the tree files and the metadata for the statistical analysis can be found on Bitbucket. (https://bitbucket.org/genomicepidemiology/metaphylogeny_paper)
