# Supplementary material for: Metaphylogenetic analysis of global sewage reveals that bacterial strains associated with human disease show less degree of geographic clustering
Source: Sci Rep. 2020 Feb 20;10:3033. doi: 10.1038/s41598-020-59292-w (PMC7033184; doi:10.1038/s41598-020-59292-w)
Supplement: Supplementary file 1 — Supplementary information [file 41598_2020_59292_MOESM1_ESM.pdf]

SUPPLEMENTAL INFORMATION

Metaphylogenetic analysis of global sewage reveals that bacterial strains associated with human disease show less degree of geographic clustering

Johanne Ahrenfeldt[1], Madina Waisi[1], Isabella C. Loft[1], Philip T.L.C. Clausen[1], Rosa Allesøe[1], Judit Szarvas[1], Rene S. Hendriksen[1], Frank M. Aarestrup[1], Ole Lund[1,*]

[1]DTU Food. Technical University of Denmark. DK-2800. Denmark.
[*]Corresponding author. olund@food.dtu.dk.

SUPPLEMENTARY FIGURE LEGENDS

Supplementary Figure S1: Quantitative overview after major steps of the pipeline. Overview of the number of bacterial templates and unique bacterial templates left, after each of the four major steps in the pipeline.

Supplementary Figure S2: Unknown bases in the consensus sequences. A barchart of percentage of unknown bases in the consensus sequences. X-axis illustrates percentage of unknown bases and Y-axis shows number of consensus sequence counts. The dotted line shows the cut-off at 40% which we ended up using.

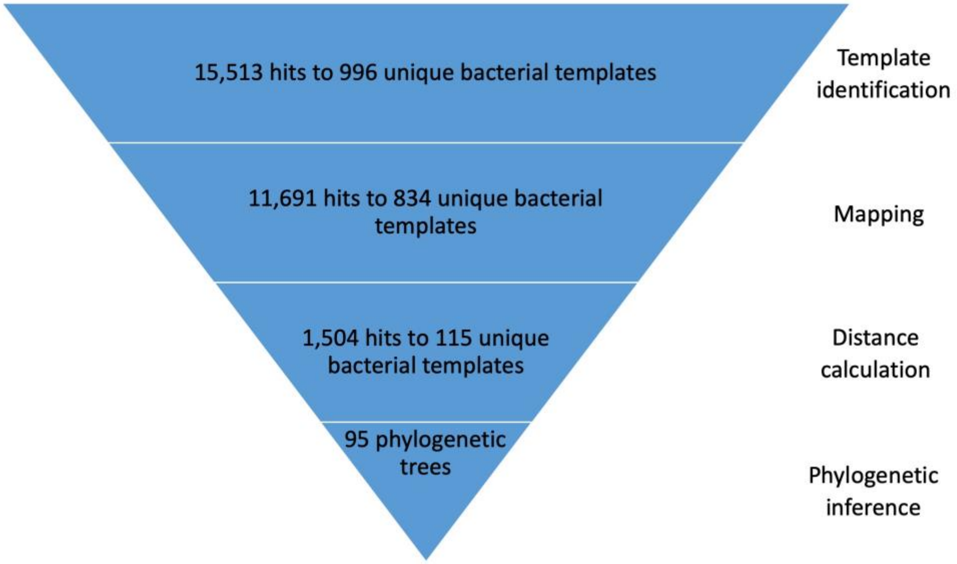

Supplementary Figure S1

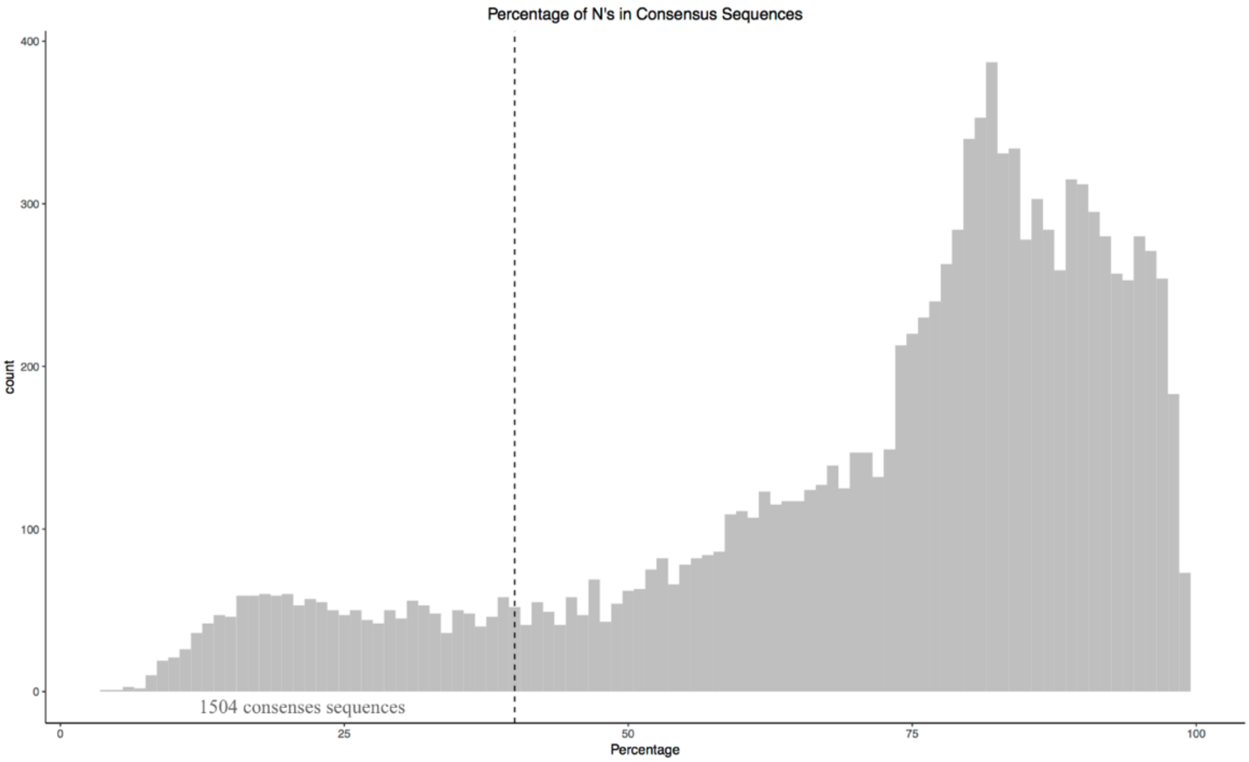

Supplementary Figure S2

SUPPLEMENTAL TABLES

Supplementary Table S1: Country and regional information on each of the 80 samples from the Global Sewage project. Only 79 samples were used in the final results, the sample from Hungary was removed due to insufficient reads.

Samplefile ID	Sample_country_region	Sample ID	Country	Region	ISO_alpha_3
17.fsa	17_Albania_Europe	17	Albania	Europe	ALB
18.fsa	18_Australia_Oceania	18	Australia	Oceania	AUS
18a.fsa	18a_Australia_Oceania	18a	Australia	Oceania	AUS
70.fsa	70_Austria_Europe	70	Austria	Europe	AUT
19.fsa	19_Botswana_Africa	19	Botswana	Africa	BWA
53.fsa	53_Brasil_SouthAmerica	53	Brasil	South America	BRA
53a.fsa	53a_Brasil_SouthAmerica	53a	Brasil	South America	BRA
66.fsa	66_Bulgaria_Europe	66	Bulgaria	Europe	BGR
21.fsa	21_Cambodia_Asia	21	Cambodia	Asia	KHM
22.fsa	22_Canada_NorthAmerica	22	Canada	North America	CAN
22a.fsa	22a_Canada_NorthAmerica	22a	Canada	North America	CAN
22b.fsa	22b_Canada_NorthAmerica	22b	Canada	North America	CAN
22c.fsa	22c_Canada_NorthAmerica	22c	Canada	North America	CAN
1.fsa	1_Chad_Africa	1	Chad	Africa	TCD
64.fsa	64_China_Asia	64	China	Asia	CHN
2.fsa	2_Colombia_SouthAmerica	2	Colombia	South America	COL
13.fsa	13_CoteD'ivoire_Africa	13	Cote D'ivoire	Africa	CIV
68.fsa	68_Croatia_Europe	68	Croatia	Europe	HRV
23.fsa	23_CzechRepublic_Europe	23	Czech Republic	Europe	CZE
RA.fsa	71RA_Denmark_Europe	71RA	Denmark	Europe	DNK
RD.fsa	71RD_Denmark_Europe	71RD	Denmark	Europe	DNK
RL.fsa	71RL_Denmark_Europe	71RL	Denmark	Europe	DNK
14.fsa	14_Ecuador_SouthAmerica	14	Ecuador	South America	ECU
14a.fsa	14a_Ecuador_SouthAmerica	14a	Ecuador	South America	ECU
24.fsa	24_Ethiopia_Africa	24	Ethiopia	Africa	ETH
25.fsa	25_Finland_Europe	25	Finland	Europe	FIN
10.fsa	10_Gambia_Africa	10	Gambia	Africa	GMB
59.fsa	59_Georgia_Asia	59	Georgia	Asia	GEO
27.fsa	27_Germany_Europe	27	Germany	Europe	DEU
4.fsa	4_Ghana_Africa	4	Ghana	Africa	GHA
61.fsa	61_Hungary_Europe	61	Hungary	Europe	HUN
28.fsa	28_Iceland_Europe	28	Iceland	Europe	ISL
11.fsa	11_India_Asia	11	India	Asia	IND
12.fsa	12_Iran_MiddleEast	12	Iran	Middle East	IRN

69.fsa	69_Ireland_Europe	69	Ireland	Europe	IRL
29.fsa	29_Israel_MiddleEast	29	Israel	Middle East	ISR
30.fsa	30_Italy_Europe	30	Italy	Europe	ITA
6.fsa	6_Kazakhstan_Asia	6	Kazakhstan	Asia	KAZ
72.fsa	72_Kenya_Africa	72	Kenya	Africa	KEN
31.fsa	31_Latvia_Europe	31	Latvia	Europe	LVA
32.fsa	32_Luxembourg_Europe	32	Luxembourg	Europe	LUX
54.fsa	54_Malaysia_Asia	54	Malaysia	Asia	MYS
63.fsa	63_Malta_Europe	63	Malta	Europe	MLT
33.fsa	33_Nepal_Asia	33	Nepal	Asia	NPL
43.fsa	43_Netherlands_Europe	43	Netherlands	Europe	NLD
56.fsa	56_NewZealand_Oceania	56	New Zealand	Oceania	NZL
50.fsa	50_Nigeria_Africa	50	Nigeria	Africa	NGA
34.fsa	34_Norway_Europe	34	Norway	Europe	NOR
7.fsa	7_Pakistan_Asia	7	Pakistan	Asia	PAK
35.fsa	35_Peru_SouthAmerica	35	Peru	South America	PER
36.fsa	36_Poland_Europe	36	Poland	Europe	POL
62.fsa	62_RepublicOfMacedonia_Europe	62	Republic Of Macedonia	Europe	MKD
65.fsa	65_RepublicOfMoldova_Europe	65	Republic Of Moldova	Europe	MDA
8.fsa	8_Senegal_Africa	8	Senegal	Africa	SEN
37.fsa	37_Serbia_Europe	37	Serbia	Europe	SRB
52.fsa	52_Singapore_Asia	52	Singapore	Asia	SGP
9.fsa	9_SlovakRepublic_Europe	9	Slovak Republic	Europe	SVK
38.fsa	38_Slovenia_Europe	38	Slovenia	Europe	SVN
39.fsa	39_SouthAfrica_Africa	39	South Africa	Africa	ZAF
75.fsa	75_Spain_Europe	75	Spain	Europe	ESP
40.fsa	40_SriLanka_Asia	40	Sri Lanka	Asia	LKA
41.fsa	41_Sweden_Europe	41	Sweden	Europe	SWE
41a.fsa	41a_Sweden_Europe	41a	Sweden	Europe	SWE
67.fsa	67_Switzerland_Europe	67	Switzerland	Europe	CHE
15.fsa	15_Tanzania_Africa	15	Tanzania	Africa	TZA
44.fsa	44_Togo_Africa	44	Togo	Africa	TGO
46.fsa	46_Turkey_Europe	46	Turkey	Europe	TUR
74.fsa	74_USA_NorthAmerica	74	USA	North America	USA
74a.fsa	74a_USA_NorthAmerica	74a	USA	North America	USA
74b.fsa	74b_USA_NorthAmerica	74b	USA	North America	USA
74c.fsa	74c_USA_NorthAmerica	74c	USA	North America	USA
74d.fsa	74d_USA_NorthAmerica	74d	USA	North America	USA
74e.fsa	74e_USA_NorthAmerica	74e	USA	North America	USA
74f.fsa	74f_USA_NorthAmerica	74f	USA	North America	USA
74g.fsa	74g_USA_NorthAmerica	74g	USA	North America	USA

74h.fsa	74h_USA_NorthAmerica	74h	USA	North America	USA
74i.fsa	74i_USA_NorthAmerica	74i	USA	North America	USA
48.fsa	48_Vietnam_Asia	48	Vietnam	Asia	VNM
49.fsa	49_Zambia_Africa	49	Zambia	Africa	ZMB
49b.fsa	49b_Zambia_Africa	49b	Zambia	Africa	ZMB

Supplementary Table S2: Taxonomic rank and classification at phylum, order, class, genus, species, and template level of the bacterial database

Taxonomic rank	Count
Phylum	34
Order	64
Class	138
Family	301
Genus	843
Species	2,319
Unique bacterial templates	3,721

Supplementary Table S3: Number of each taxonomic rank and classification at phylum, order, class, genus, species, and template level of the bacterial templates identified in the 79 sewage samples.

Taxonomic rank	After step B	After step D	Phylogenetic trees
Phylum	15	6	6
Class	25	10	10
Order	61	15	13
Family	120	19	18
Genus	279	31	27
Species	653	73	62
Unique bacterial templates	834	115	95

Supplementary Table S4: p-values for tests of organism group differences in clustering. EID2p organism classifications. P-values < 0.05 are white, while those above are colored grey.

	World Bank Regions	World Bank Income Levels	WHO Regions	WHO Health Impact
Commensal ↔ Environmental	0.79	0.94	0.91	0.029
Commensal ↔ Pathogen	0.014	0.77	0.0039	0.022
Environmental ↔ Pathogen	0.039	0.75	0.039	0.94

Supplementary Table S5: p-values for tests of organism group differences in clustering. 5CC organism classification. P-values < 0.05 are white, while those above are colored grey.

	World Bank Regions	World Bank Income Levels	WHO Regions	WHO Health Impact
Commensal ↔ COP	0.077	0.29	0.042	0.00078
Commensal ↔ Environmental	0.27	0.79	0.86	0.10
Commensal ↔ Opportunistic pathogen	0.026	0.54	0.028	0.66
Commensal ↔ Pathogen	0.12	0.17	0.074	0.23
COP ↔ Environmental	0.048	0.23	0.32	0.034
COP ↔ Opportunistic pathogen	0.33	0.067	0.48	0.0034
COP ↔ Pathogen	0.39	0.038	0.24	0.50
Environmental ↔ Opportunistic pathogen	0.019	0.80	0.14	0.31
Environmental ↔ Pathogen	0.21	0.24	0.18	0.39
Opportunistic pathogen ↔ Pathogen	0.58	0.30	0.69	0.28

Supplementary Table S6: Number of countries sampled for each of the region groupings used. The regions listed in the same row are those with the highest amount of countries in common.

World Bank Regions	Countries sampled	WHO Regions	Countries sampled
Sub-Saharan Africa	13	Africa	13
Latin America & Caribbean	4	Americas	6
North America	2		
Middle East & North Africa	3	Eastern Mediterranean	2
Europe & Central Asia	28	Europe	30
South Asia	4	South-East Asia	3

East Asia & Pacific	7	Western Pacific	7

Supplementary Table S7: Regional differences in country groupings between WB-R and WHO-R

ISO alpha 3	Country name	World Bank Regions	WHO Regions
PAK	Pakistan	South Asia	Eastern Mediterranean
ISR	Israel	Middle East & North Africa	Europe
MLT	Malta	Middle East & North Africa	Europe
COL	Colombia	Latin America & Caribbean	Americas
ECU	Ecuador	Latin America & Caribbean	Americas
PER	Peru	Latin America & Caribbean	Americas
BRA	Brazil	Latin America & Caribbean	Americas
CAN	Canada	North America	Americas
USA	United States of America	North America	Americas

SUPPLEMENTAL METHODS

Classifications of bacterial templates

Here follows the step by step description of how the two classifications schemes EID2 plus (EID2p) and Five class classification (5CC) was made. EID2p is made by step 1-3 and is mostly built on the EID2 database [1], but with an additional step (step 3) hence the name EID2 plus. The 5CC is made by adding step 4 to the EID2p scheme

Step 1 – Annotate for human interaction

The used database: EID2 (enhanced infectious diseases database) [1]: https://eid2.liverpool.ac.uk/
Terms and conditions: https://eid2.liverpool.ac.uk/Home/TermsAndConditions

EID2 categorized the information as carrier and cargo.
Carrier correspond to a vector or a host.
Cargo correspond to a potential pathogen, parasite, commensal etc.

Furthermore, all organisms are categorized into taxa: bacteria, invertebrates, mammal, plant, primates, rodents, vertebrates, and virus.

The database was scraped for information about interactions where the cargo taxa were bacteria, and carrier taxa were every possible taxon.

The scrape output was then separated according to carrier taxa, where information had to be about carriers at rank ‘species’ and cargo at either rank ‘no rank’ or ‘species’. This resulted in files containing carrier information about bacteria, invertebrates, mammal, plant primates, rodents, and vertebrates.

Lastly the information about homo sapiens interactions with bacteria were extracted from the file containing information about primate interactions with bacteria.

Step 2 – Lookup of intersection between the human interaction list and the template list

Next the EID2 homo sapiens information was used to assign each template organism to either ‘human interaction’ or ‘environmental’.

Firstly, a match between the template organism name and cargo at rank ‘no rank’ was searched for. If a match is found the template is classified as ‘human interaction’. If no match was found at ‘no rank’ level, a match at species level is searched for. If a match was found the organism is classified as ‘human interaction’, else it was classified as ‘environmental’.

When a match was identified, the name from the EID2 was noted down. If no match was found, NA was noted.

The conditions for which classification was assigned can be seen in the flow diagram below.

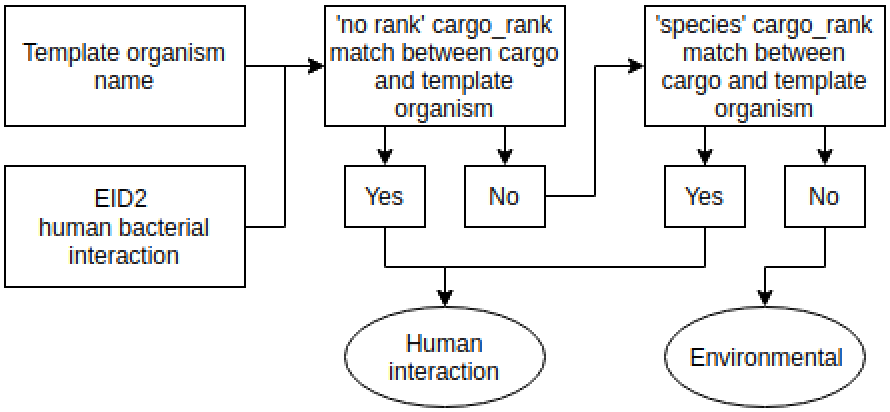

Information can be found in the columns:
- EID2.interaction.step2

Listing the classification either ‘human interaction’ or ‘environmental’
- EID2.closest.match.interactions.step2

Listing the closest match between the template organism name and the EID2 database of bacterial cargos in humans.

Step 3 – Lookup for pathogenesis in the list from the article: Risk factors for human disease emergence (2001), Taylor, L. H. et *al*. [2]

Search for a match between the list of pathogens from Taylor et *al*. and the template organism name. This can only be done at species level since Taylor et *al*. only include information about pathogenic bacteria at species level.

The template organisms were further classified as commensal, pathogen, or environmental.

If the template has a match to the Taylor list, it is classified as ‘pathogen’ regardless of its classification in step 2. If the template does not have a match in the Taylor list it will be classified as ‘commensal’ if it was classified as ‘human interaction’ in step 2. If it was classified as ‘environmental’ in step 2, it will keep its ‘environmental’ classification.

Note that if Taylor et *al*. has listed a genus (e.g. *Lactobacillus sp*. and *Megaspaera sp*.) as pathogenic, this will not be accepted as an evidence to classify the relevant templates as ‘pathogen’.

The flow diagram below gives an overview of the decision flow.

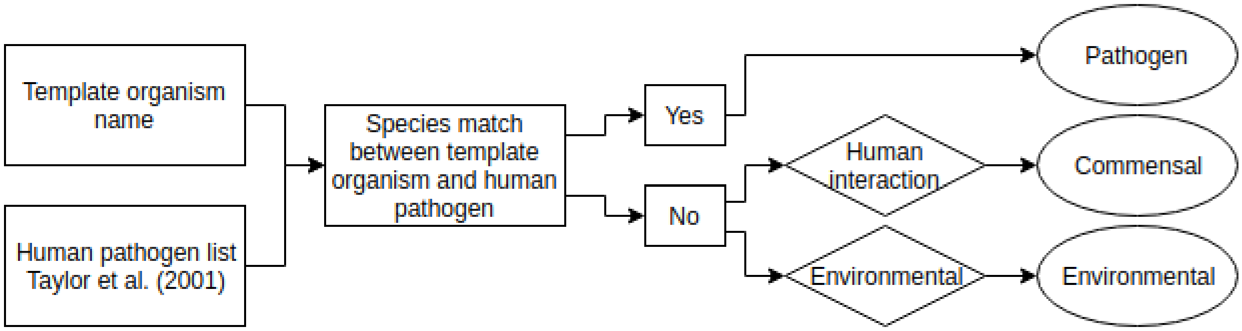

Information can be found in the columns:
- classification.pathogen.Taylor.step3
List classification of either 'pathogen', 'commensal', or 'environmental'

- classification.pathogen.zoonotic.Taylor.step3
From Taylor et al. 2001, list if pathogen is defined as zoonotic

Step 4 – Five class classification (5CC). Hand curated classification, made by web search for pathogenesis grouping as well as confirming commensal and environmental status.

In this step pathogens are grouped according to the definition made by Price L.B et *al.* in Colonizing opportunistic pathogens (COPs): The beast in all of us (2017) [3]

In the paper, pathogens are divided into three groups:
Colonizing opportunistic pathogens (COPs)
Simple opportunistic pathogens (SOPs)
Frank pathogens

Definitions listed by the paper:
Colonizing opportunistic pathogens (COPs) are microbes that asymptomatically colonize the human body and, when the conditions are right, can cause infections.

The broad category of opportunistic pathogens can be divided into two distinct subgroups: the COPs and the noncolonizing, simple opportunistic pathogens (SOP).

The defining feature of all opportunistic pathogens is their capacity to cause disease when they are introduced into a susceptible body site or when hosts are immunologically compromised.

Whereas SOPs, such as *Vibrio vulnificus*, *Mycobacterium marinum*, and *Legionella pneumophila*, are only present in environmental reservoirs, COPs can also take up long-term residence in/on the human body as part of the "normal" human microbiome.

Frank pathogens, can cause acute, chronic, or latent infections that can be symptomatic or asymptomatic.

The detection of frank pathogens is often associated with a diseased status, whether active or latent, and identifying cases of active or recent infections is usually enough to trace transmission routes.

Groupings used in our study:
- Commensal
- COP
- Environmental
- Opportunistic pathogen
- Pathogen

Pathogens = frank pathogens
Opportunistic pathogens = noncolonizing/simple opportunistic pathogens

Search protocol
First look for the templates' names, and investigate what the specific paper for the sequencing of the organism (if it exists) mention about the organism.
Afterward, search if there are any other articles mentioning the same organism.

Hereafter, search for descriptions of the species itself, to investigate what tendencies the species has.

Search also for the keywords pathogen, commensal, human, environment in relation to the organism.

Commensal definition:
If an article search gives results mentioning human interaction. For example, used in food production of yogurt, fermentation procedures *etc*.
If found in human derived samples, and there are no results when searching for the organism with pathogen as an additional search term.

COPs definition:
Can cause disease in immunocompromised people. Mentioned as pathogen.
Can colonize the microbiome without causing disease, in some instances the bacteria are ubiquitous to the environment, meaning it can be present in soil, domestic animals as well as human microbiomes, plants etc.

Environmental definition:
If an article search shows no evidence of human interaction, or optimal growth conditions are far removed from those of the human body, e.g. a psychrophilic bacterium with optimal growth temperature below that of the human body.

Opportunistic pathogen definition:
Can cause disease in immunocompromised people.
Does not colonize the human microbiome, normally act as environmental bacteria except when causing infections.

Pathogen defintions:
Obligate/frank pathogen, cause infection when in contact with human, both immunocompetent as well as immunocompromised people.
There should be no evidence of the bacteria colonizing the human microbiome, it normally acts as environmental when not infecting humans.

Columns
- Web.search.step4
Listing classification; commensal, COP, environmental, opportunistic pathogen, pathogen
- Search.notes.step4
Notes taken while searching for information regarding each organism
- Ref1.step4
Link to paper with information regarding the organism
- Ref2.step4
Additional link to paper with information regarding the organism
- Ref3.step4
Additional link to paper with information regarding the organism

References

1. Wardeh, M., Risley, C., McIntyre, M. K., Setzkorn, C. & Baylis, M. Database of host-pathogen and related species interactions, and their global distribution. *Sci. Data* **2,** 150049 (2015).
2. Taylor, L. H., Latham, S. M. & Woolhouse, M. E. Risk factors for human disease emergence. *Philos. Trans. R. Soc. Lond. B. Biol. Sci.* **356,** 983–9 (2001).
3. Price, L. B., Hungate, B. A., Koch, B. J., Davis, G. S. & Liu, C. M. Colonizing opportunistic pathogens (COPs): The beasts in all of us. *PLOS Pathog.* **13,** e1006369 (2017).
