# Supplementary material for: Metaphylogenetic analysis of global sewage reveals that bacterial strains associated with human disease show less degree of geographic clustering
Source: Sci Rep. 2020 Feb 20;10:3033. doi: 10.1038/s41598-020-59292-w (PMC7033184; doi:10.1038/s41598-020-59292-w)

SUPPLEMETARY DATASET 1

Metaphylogenetic analysis of global sewage reveals that bacterial strains associated with human disease show less degree of geographic clustering

Johanne Ahrenfeldt[1], Madina Waisi[1], Isabella C. Loft[1], Philip T.L.C. Clausen[1], Rosa Allesøe[1], Judit Szarvas[1], Rene S. Hendriksen[1], Frank M. Aarestrup[1], Ole Lund[1,*]

[1]DTU Food. Technical University of Denmark. DK-2800. Denmark.
[*]Corresponding author. olund@food.dtu.dk.

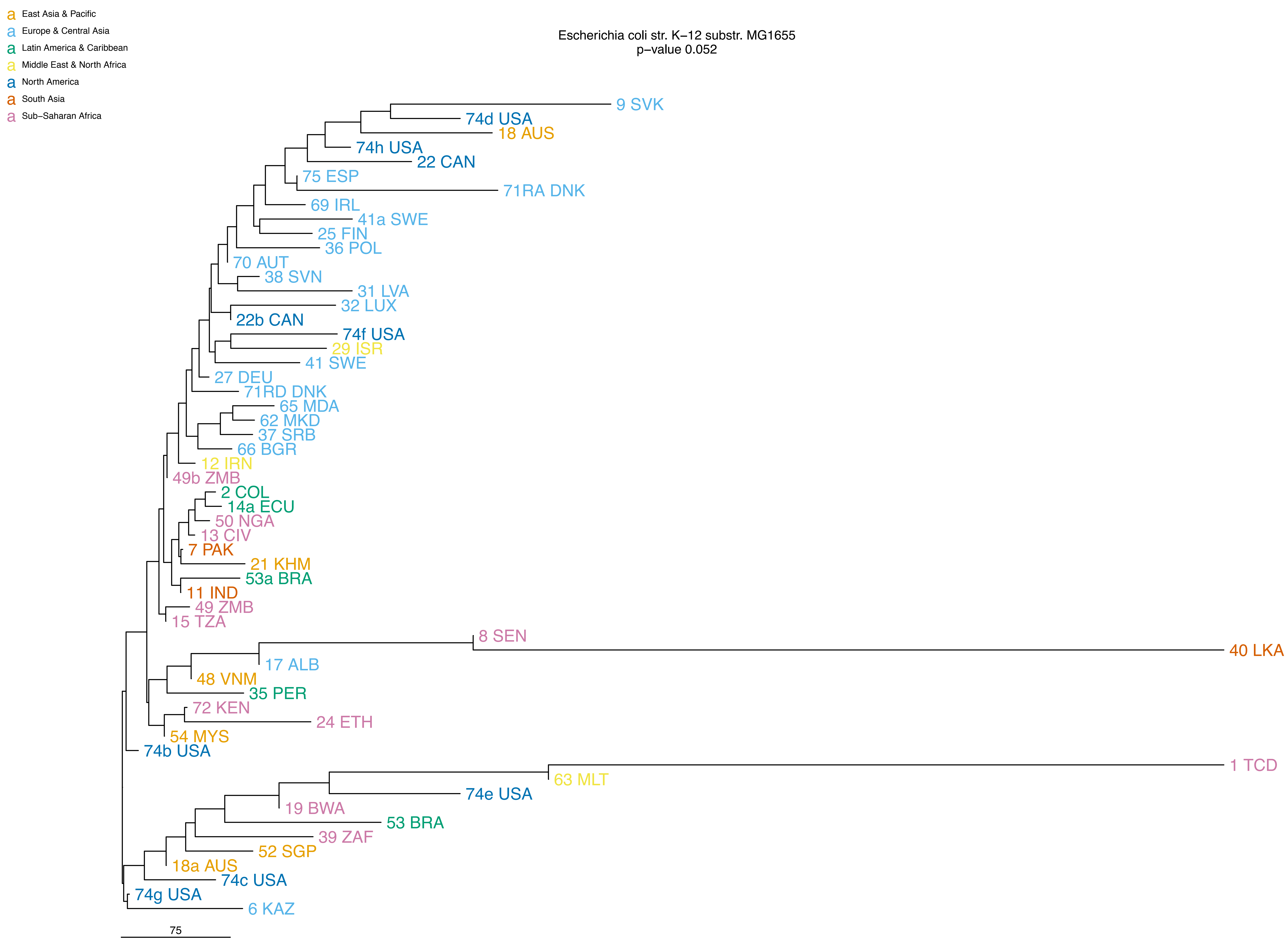

a East Asia & Pacific
a Europe & Central Asia
a Latin America & Caribbean
a Middle East & North Africa
a North America
a South Asia
a Sub–Saharan Africa
Escherichia coli str. K–12 substr. MG1655
p–value 0.052
9 SVK
74d USA
18 AUS
74h USA
22 CAN
75 ESP
71RA DNK
69 IRL
41a SWE
25 FIN
36 POL
70 AUT
38 SVN
31 LVA
32 LUX
22b CAN
74f USA
29 ISR
41 SWE
27 DEU
71RD DNK
65 MDA
62 MKD
37 SRB
66 BGR
12 IRN
49b ZMB
2 COL
14a ECU
50 NGA
13 CIV
7 PAK
21 KHM
53a BRA
11 IND
49 ZMB
15 TZA
8 SEN
40 LKA
17 ALB
48 VNM
35 PER
72 KEN
24 ETH
54 MYS
74b USA
1 TCD
63 MLT
74e USA
19 BWA
53 BRA
39 ZAF
52 SGP
18a AUS
74c USA
74g USA
6 KAZ
75

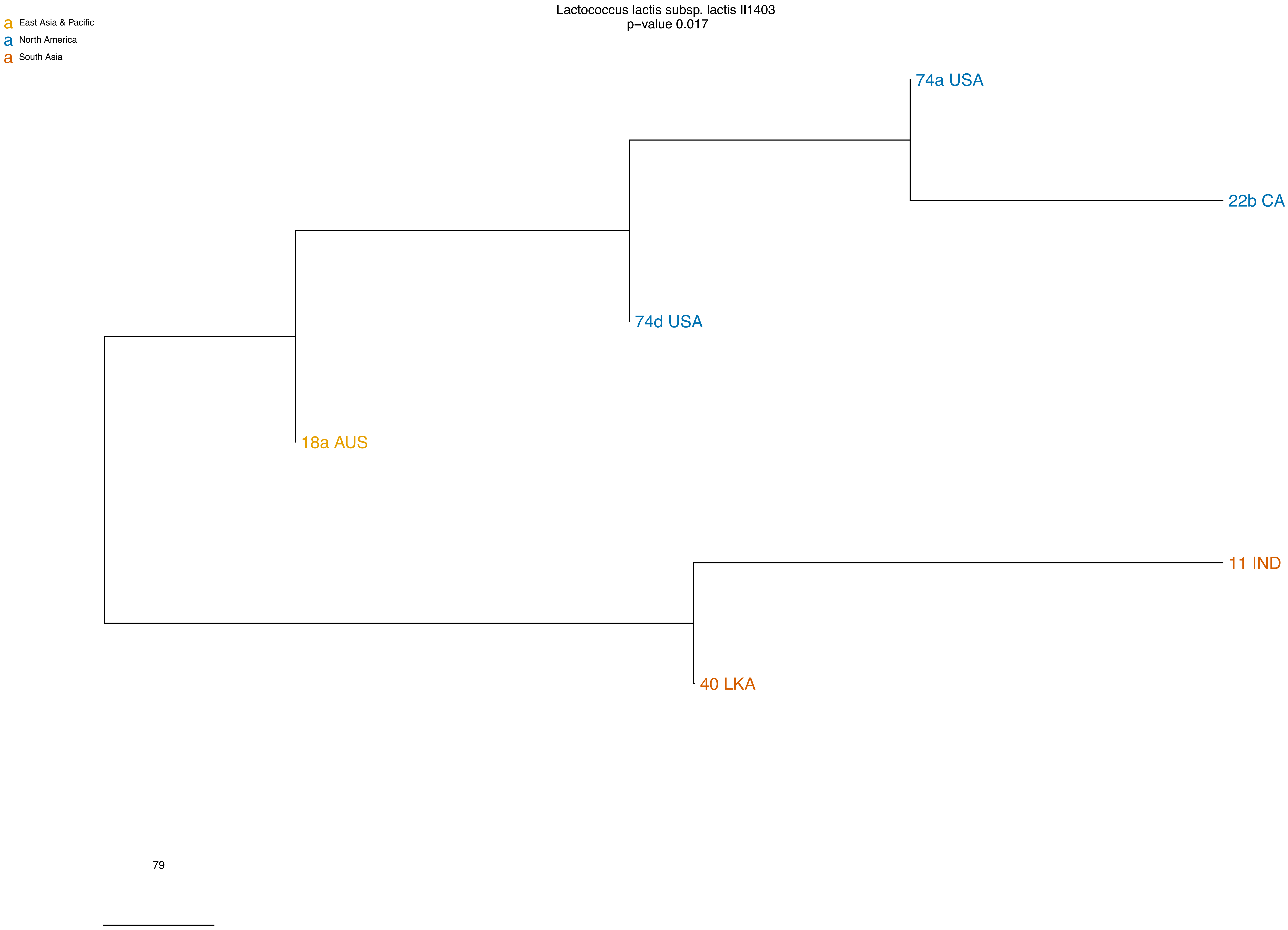
East Asia & Pacific
North America
South Asia
Lactococcus lactis subsp. lactis Il1403
p–value 0.017
74a USA
22b CA
74d USA
18a AUS
11 IND
40 LKA
79

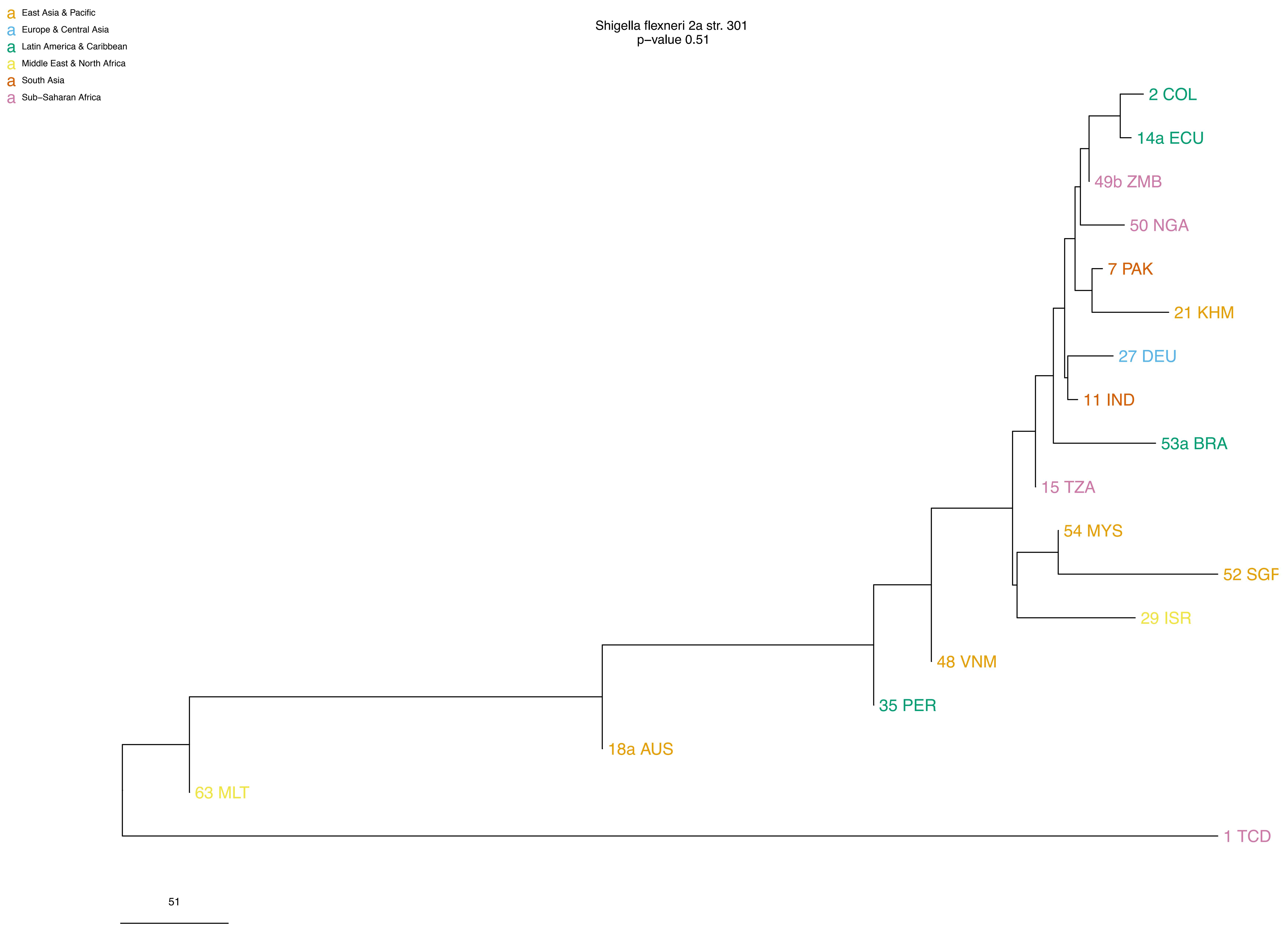

Shigella flexneri 2a str. 301
p–value 0.51
a East Asia & Pacific
a Europe & Central Asia
a Latin America & Caribbean
a Middle East & North Africa
a South Asia
a Sub–Saharan Africa
2 COL
14a ECU
49b ZMB
50 NGA
7 PAK
21 KHM
27 DEU
11 IND
53a BRA
15 TZA
54 MYS
52 SGF
29 ISR
48 VNM
35 PER
18a AUS
63 MLT
1 TCD
51

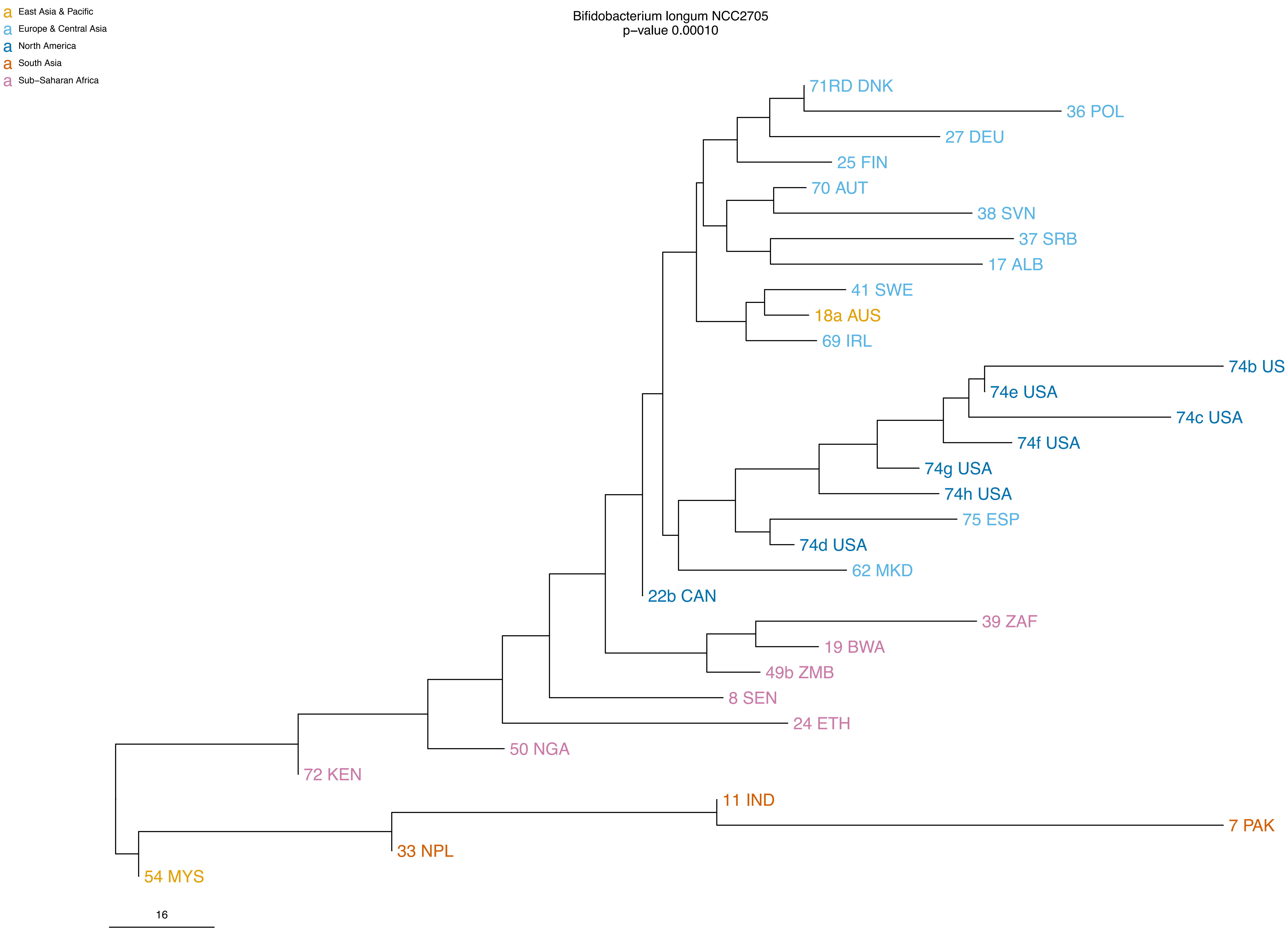

a East Asia & Pacific
a Europe & Central Asia
a North America
a South Asia
a Sub–Saharan Africa
Bifidobacterium longum NCC2705
p–value 0.00010
71RD DNK
36 POL
27 DEU
25 FIN
70 AUT
38 SVN
37 SRB
17 ALB
41 SWE
18a AUS
69 IRL
74b US
74e USA
74c USA
74f USA
74g USA
74h USA
75 ESP
74d USA
62 MKD
22b CAN
39 ZAF
19 BWA
49b ZMB
8 SEN
24 ETH
50 NGA
72 KEN
11 IND
7 PAK
33 NPL
54 MYS
16

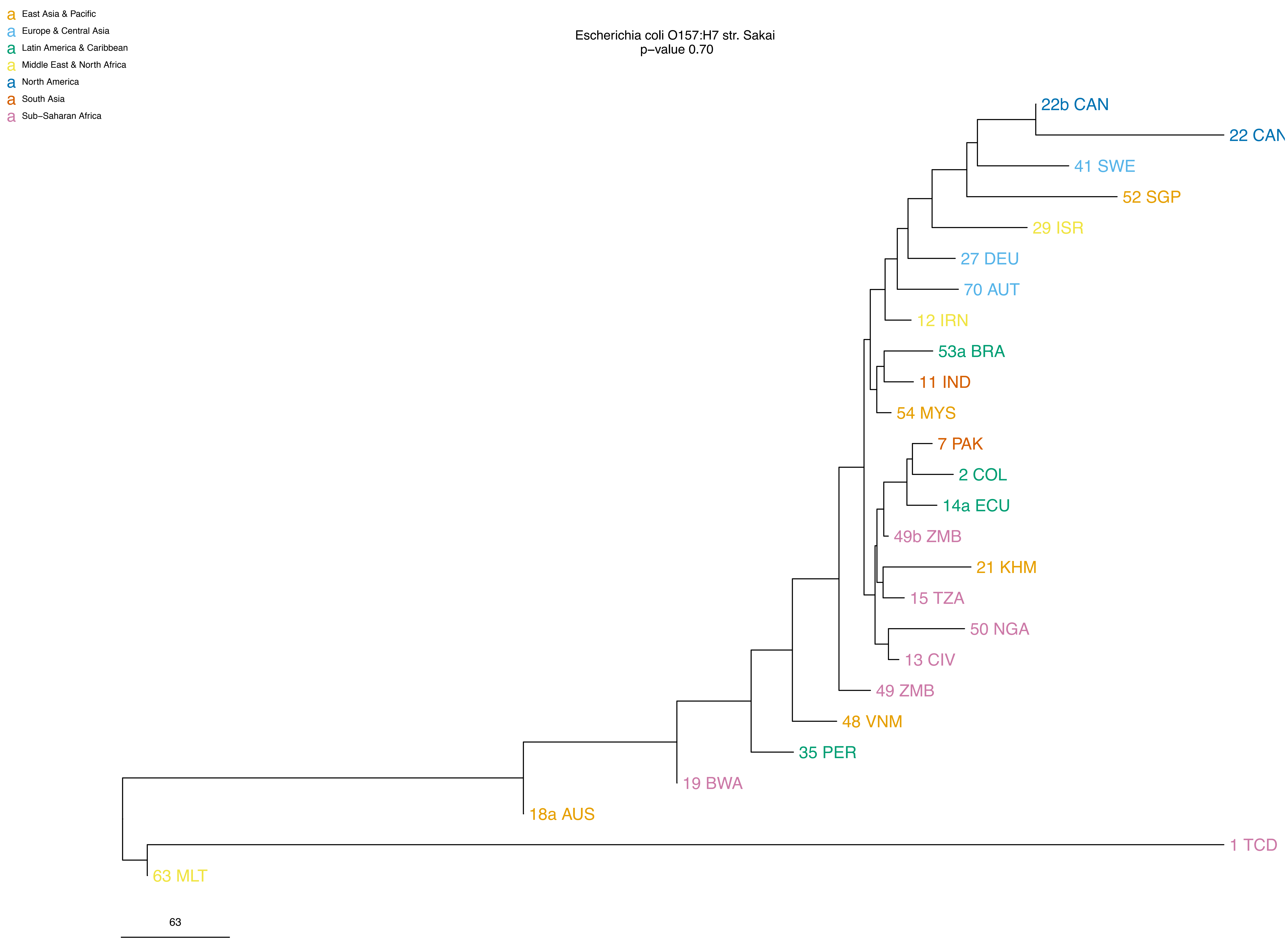

a East Asia & Pacific
a Europe & Central Asia
a Latin America & Caribbean
a Middle East & North Africa
a North America
a South Asia
a Sub–Saharan Africa
Escherichia coli O157:H7 str. Sakai
p–value 0.70
22b CAN
22 CAN
41 SWE
52 SGP
29 ISR
27 DEU
70 AUT
12 IRN
53a BRA
11 IND
54 MYS
7 PAK
2 COL
14a ECU
49b ZMB
21 KHM
15 TZA
50 NGA
13 CIV
49 ZMB
48 VNM
35 PER
19 BWA
18a AUS
1 TCD
63 MLT
63

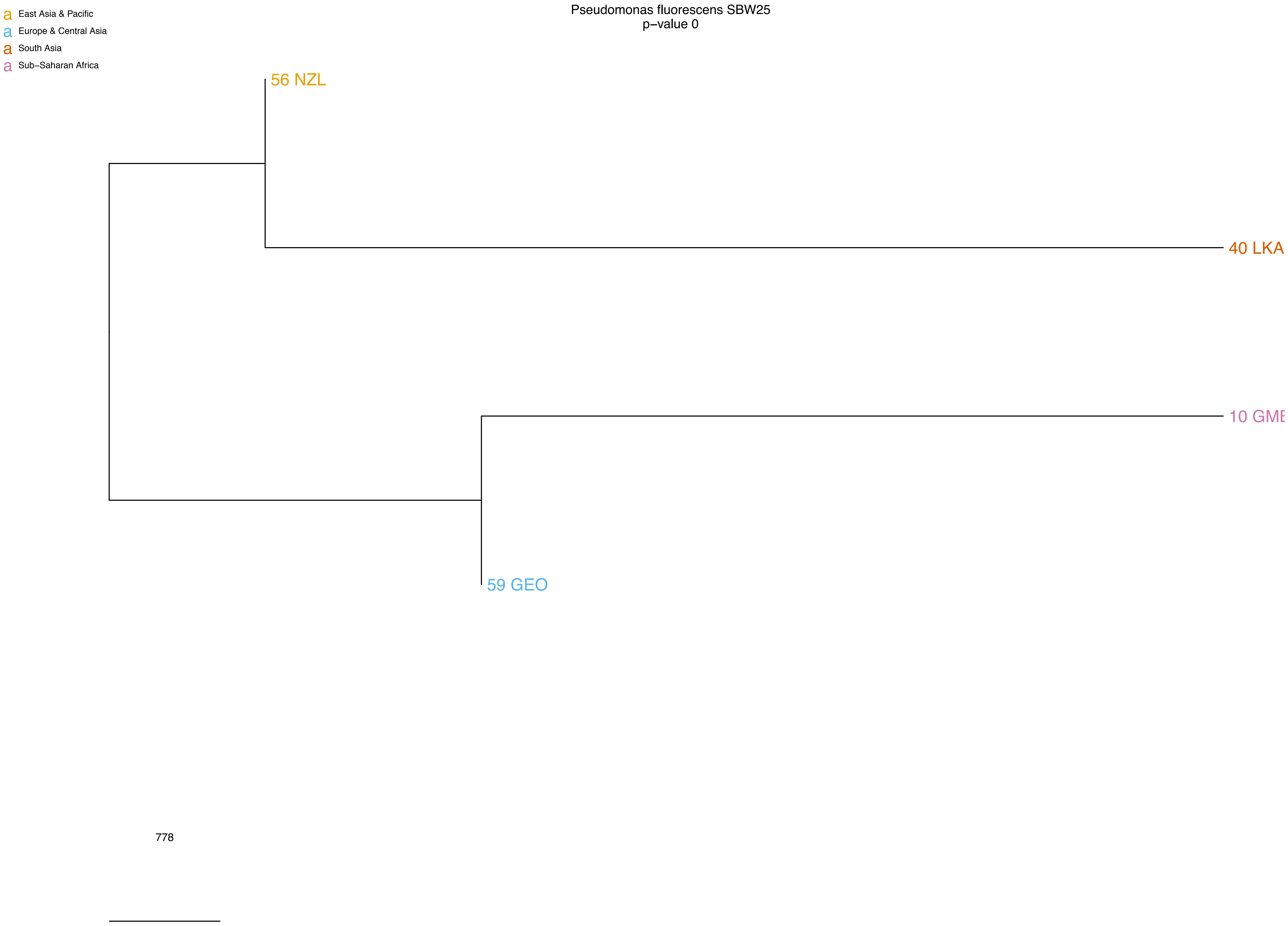
a East Asia & Pacific
a Europe & Central Asia
a South Asia
a Sub–Saharan Africa
Pseudomonas fluorescens SBW25
p–value 0
56 NZL
40 LKA
10 GMI
59 GEO
778

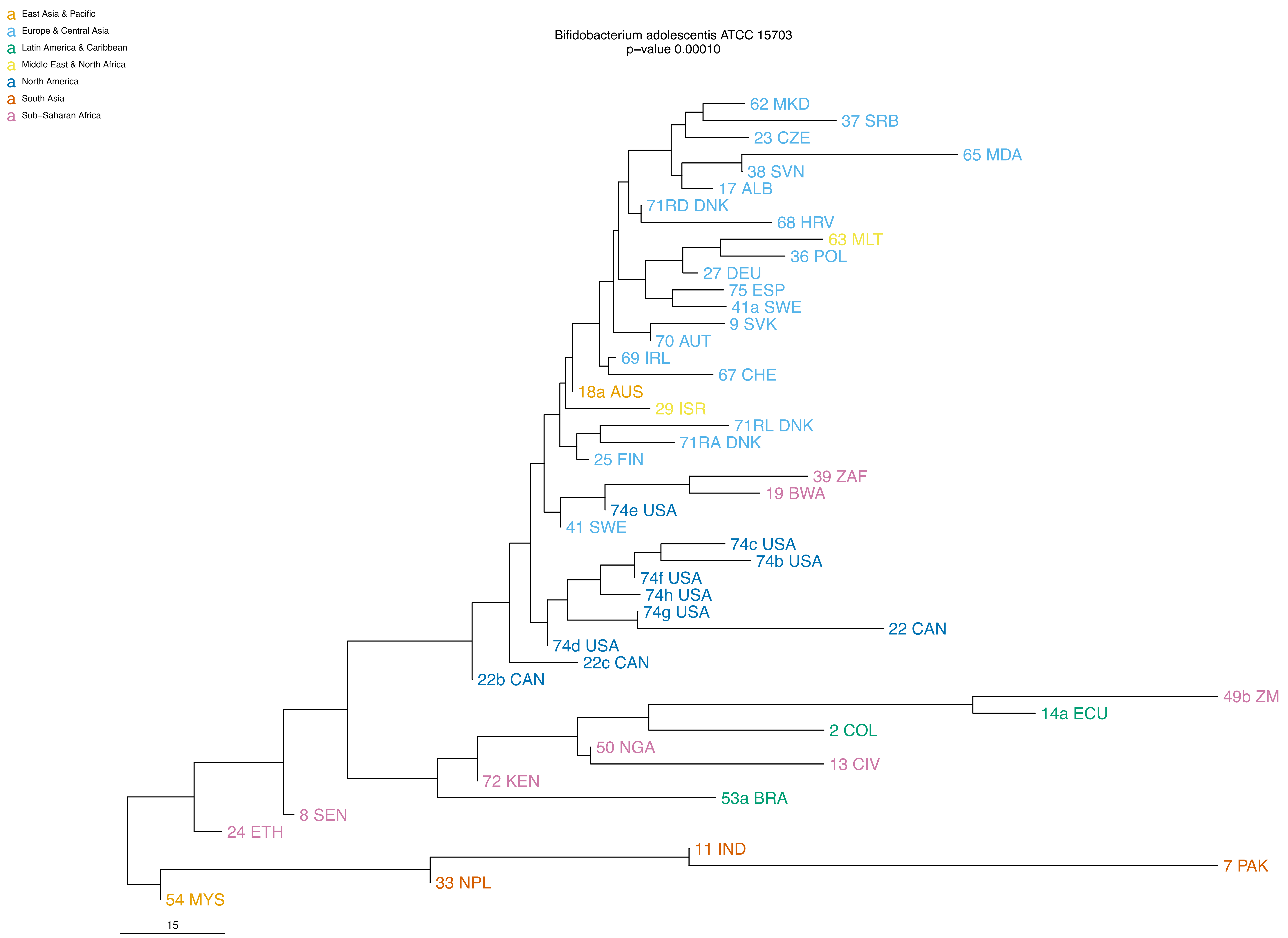

a East Asia & Pacific
a Europe & Central Asia
a Latin America & Caribbean
a Middle East & North Africa
a North America
a South Asia
a Sub–Saharan Africa
Bifidobacterium adolescentis ATCC 15703
p–value 0.00010
62 MKD
37 SRB
23 CZE
65 MDA
38 SVN
17 ALB
71RD DNK
68 HRV
63 MLT
36 POL
27 DEU
75 ESP
41a SWE
9 SVK
70 AUT
69 IRL
67 CHE
18a AUS
29 ISR
71RL DNK
71RA DNK
25 FIN
39 ZAF
19 BWA
74e USA
41 SWE
74c USA
74b USA
74f USA
74h USA
74g USA
22 CAN
74d USA
22c CAN
22b CAN
49b ZM
14a ECU
2 COL
50 NGA
13 CIV
72 KEN
53a BRA
8 SEN
24 ETH
11 IND
7 PAK
33 NPL
54 MYS
15

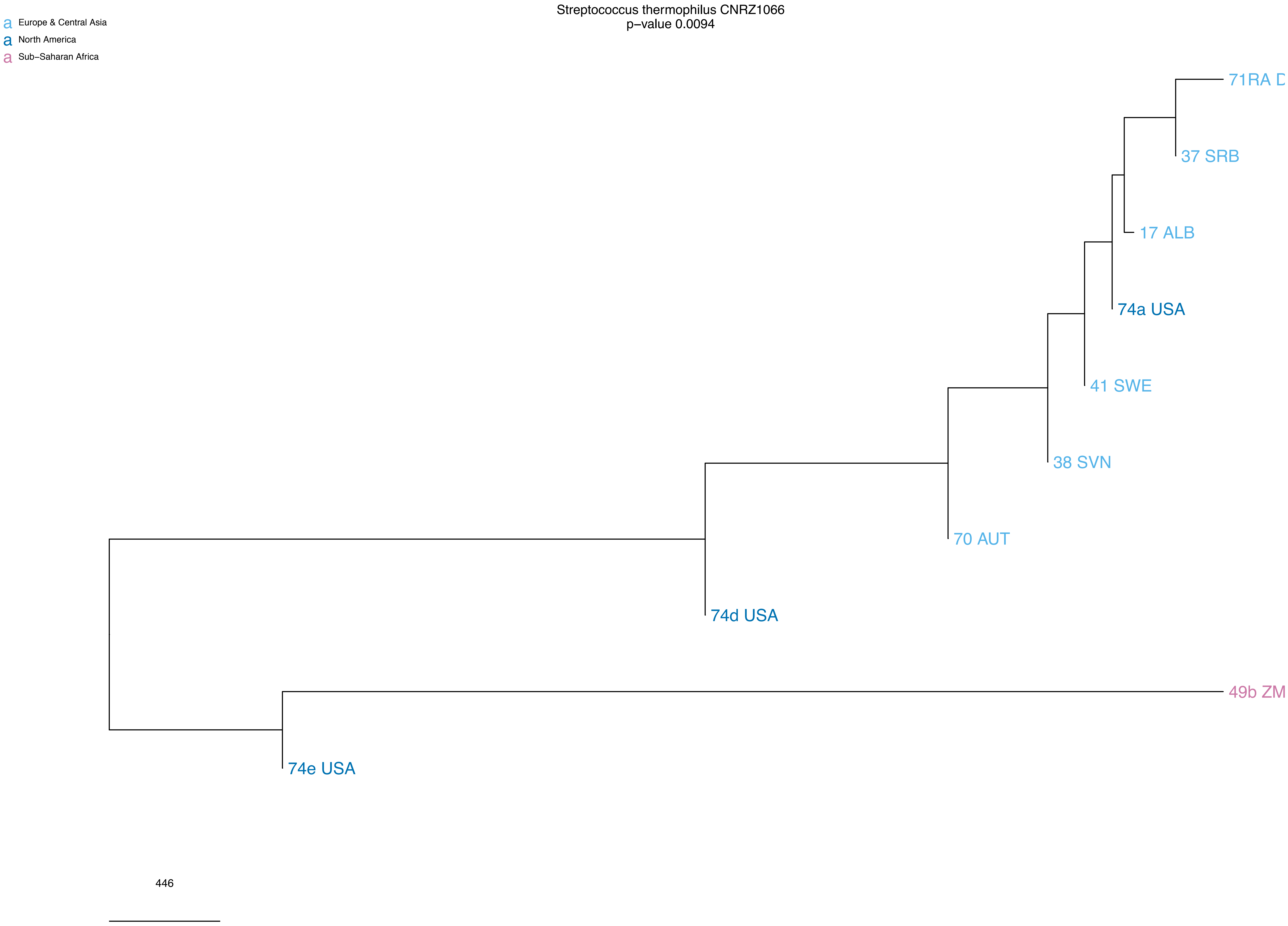

Streptococcus thermophilus CNRZ1066
p–value 0.0094
a Europe & Central Asia
a North America
a Sub–Saharan Africa
71RA D
37 SRB
17 ALB
74a USA
41 SWE
38 SVN
70 AUT
74d USA
49b ZM
74e USA
446

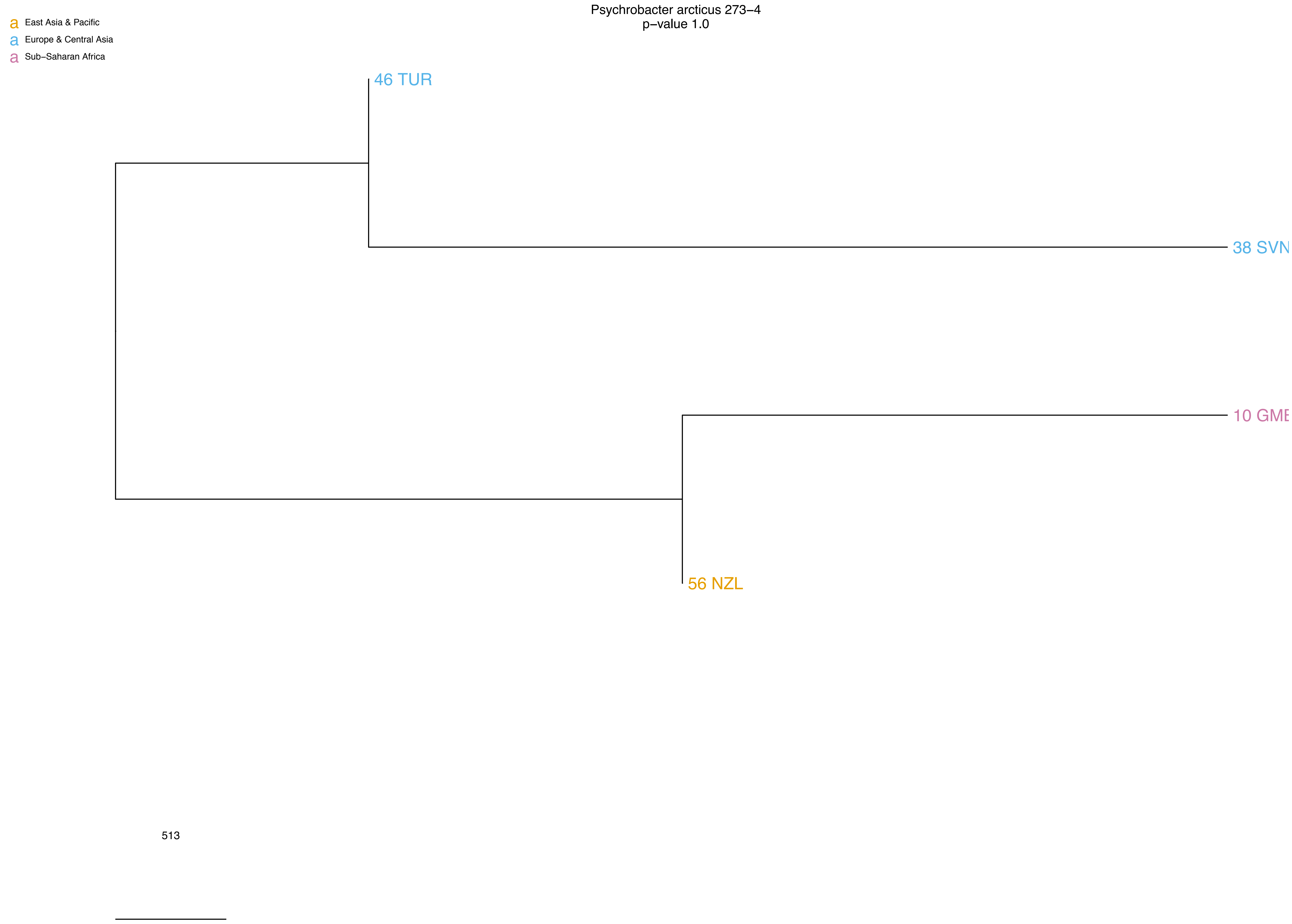

Psychrobacter arcticus 273−4
p−value 1.0
East Asia & Pacific
Europe & Central Asia
Sub−Saharan Africa
46 TUR
38 SVN
10 GMB
56 NZL
513

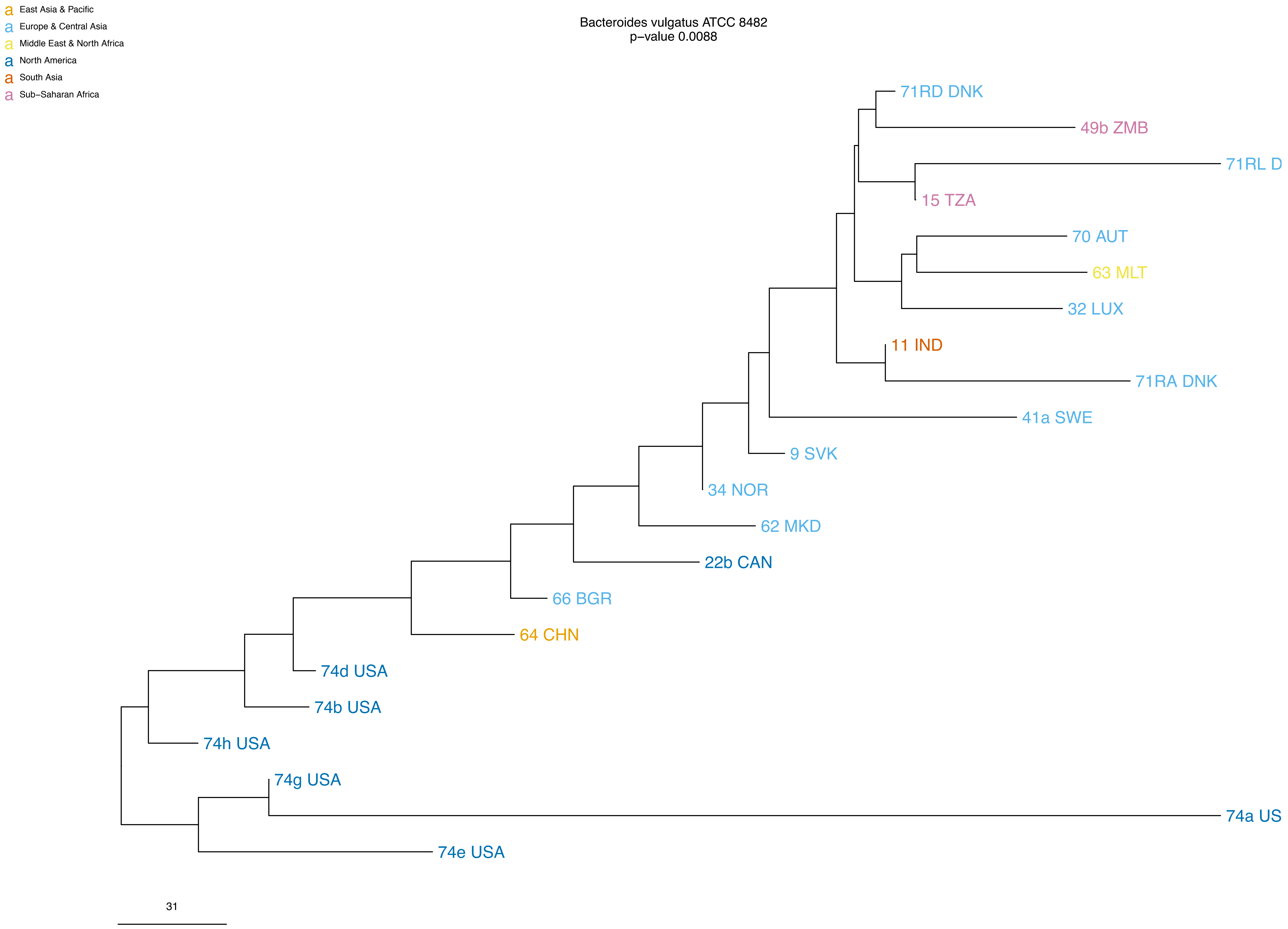

East Asia & Pacific
Europe & Central Asia
Middle East & North Africa
North America
South Asia
Sub–Saharan Africa
Bacteroides vulgatus ATCC 8482
p–value 0.0088
71RD DNK
49b ZMB
71RL D
15 TZA
70 AUT
63 MLT
32 LUX
11 IND
71RA DNK
41a SWE
9 SVK
34 NOR
62 MKD
22b CAN
66 BGR
64 CHN
74d USA
74b USA
74h USA
74g USA
74a US
74e USA
31

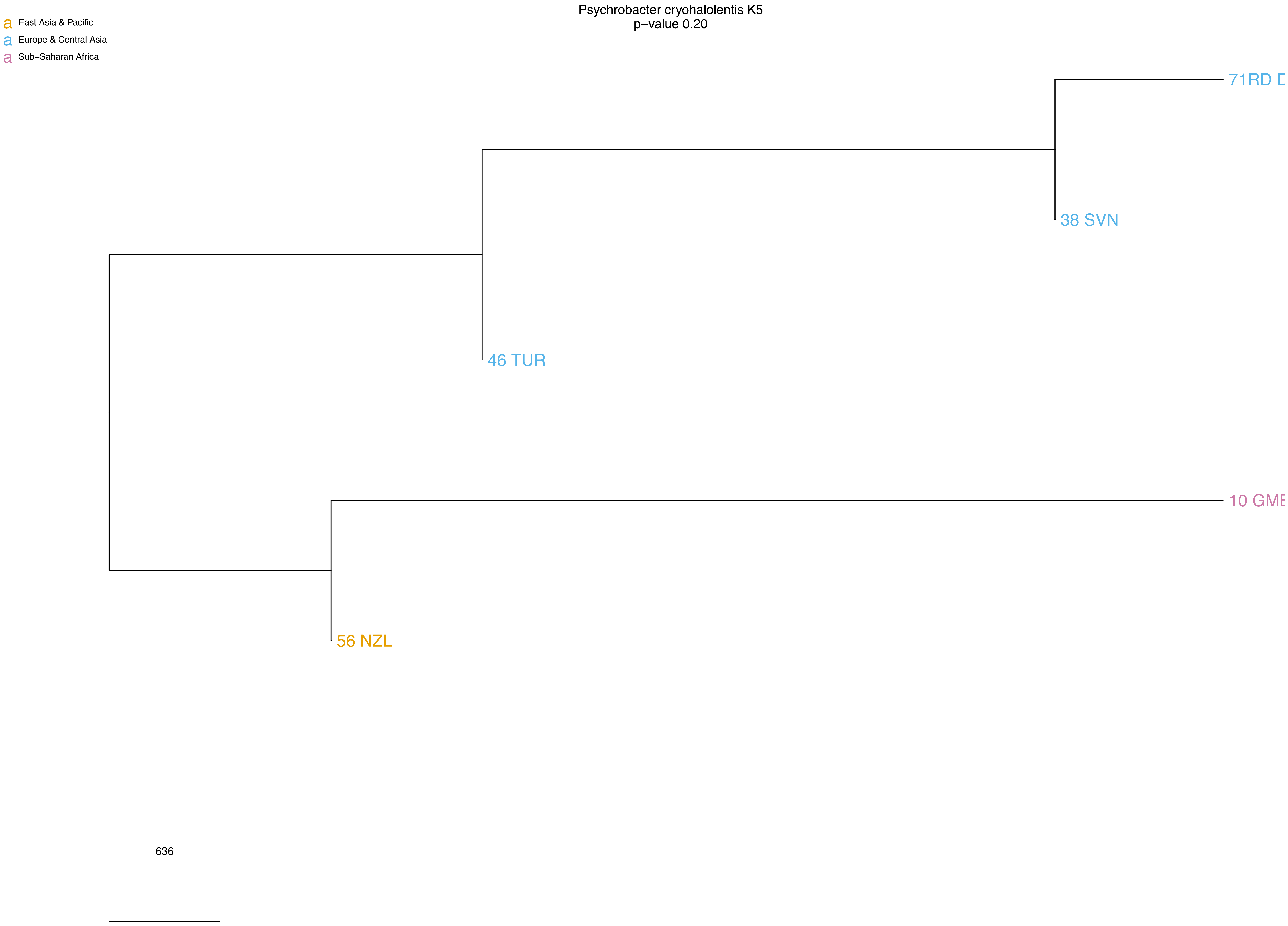

Psychrobacter cryohalolentis K5
p–value 0.20
East Asia & Pacific
Europe & Central Asia
Sub–Saharan Africa
71RD D
38 SVN
46 TUR
10 GMB
56 NZL
636

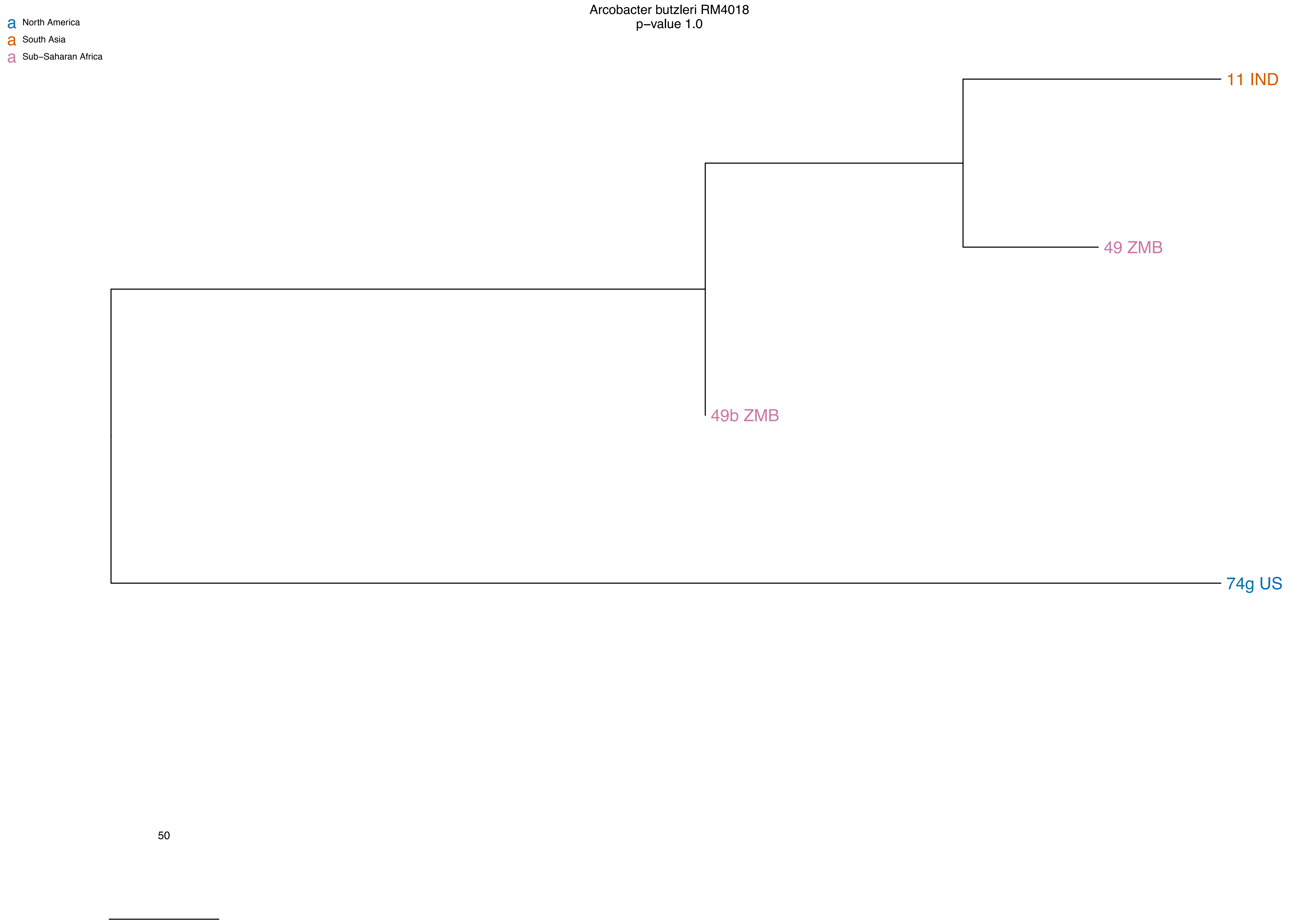

Arcobacter butzleri RM4018
p–value 1.0
a North America
a South Asia
a Sub–Saharan Africa
11 IND
49 ZMB
49b ZMB
74g US
50

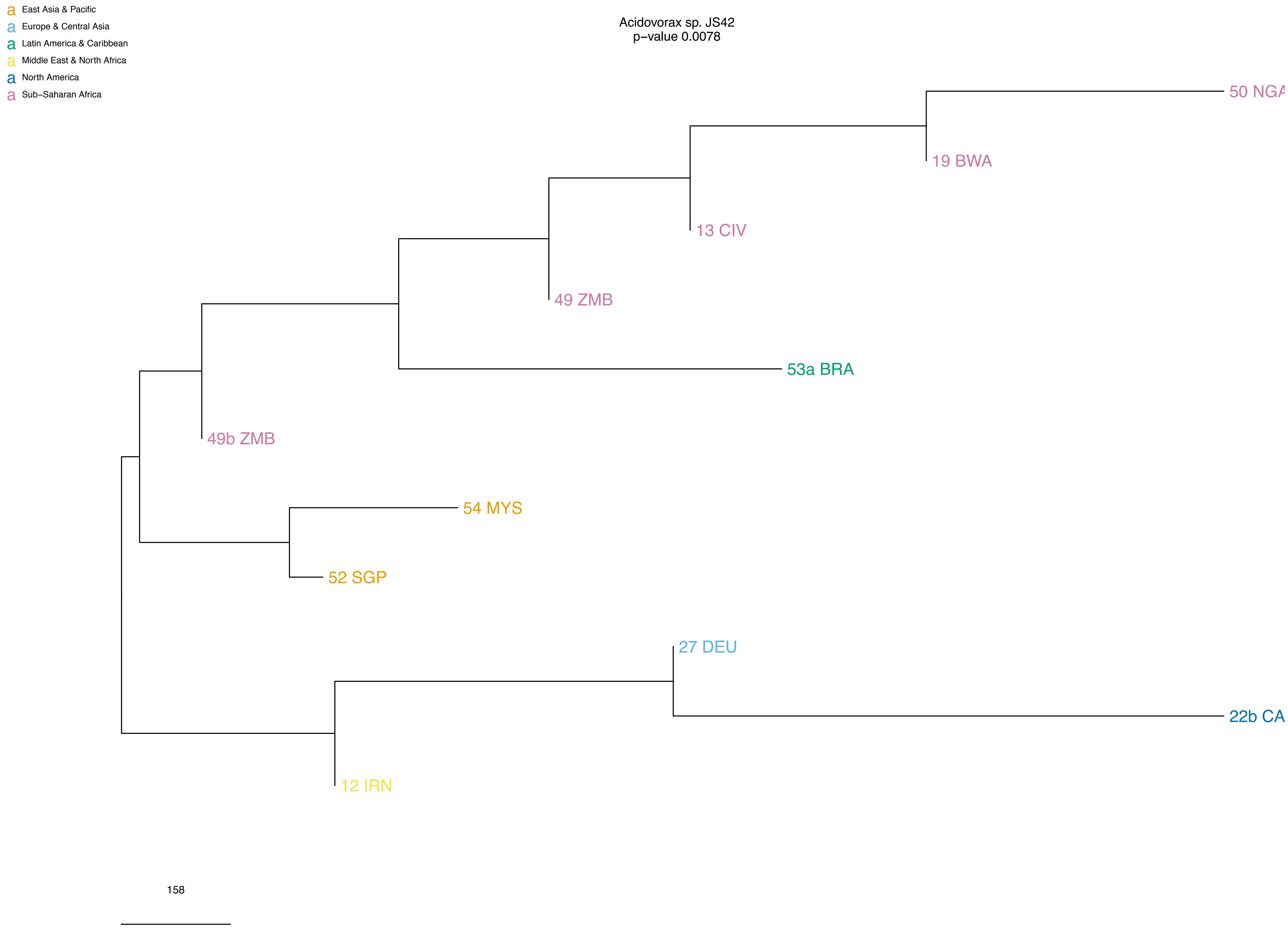
East Asia & Pacific
Europe & Central Asia
Latin America & Caribbean
Middle East & North Africa
North America
Sub–Saharan Africa
Acidovorax sp. JS42
p–value 0.0078
50 NGA
19 BWA
13 CIV
49 ZMB
53a BRA
49b ZMB
54 MYS
52 SGP
27 DEU
22b CA
12 IRN
158

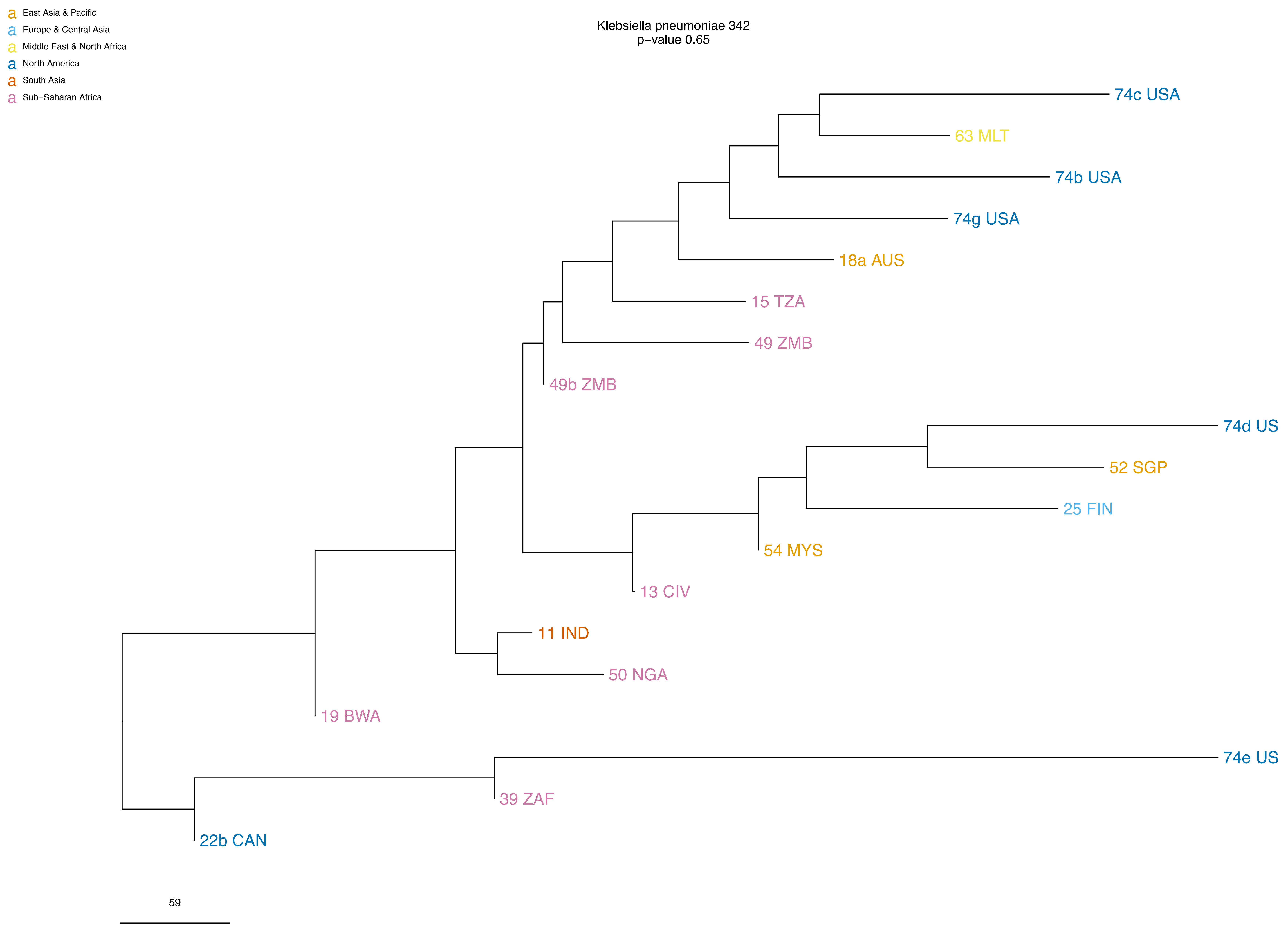
East Asia & Pacific
Europe & Central Asia
Middle East & North Africa
North America
South Asia
Sub–Saharan Africa
Klebsiella pneumoniae 342
p–value 0.65
74c USA
63 MLT
74b USA
74g USA
18a AUS
15 TZA
49 ZMB
49b ZMB
74d US
52 SGP
25 FIN
54 MYS
13 CIV
11 IND
50 NGA
19 BWA
74e US
39 ZAF
22b CAN
59

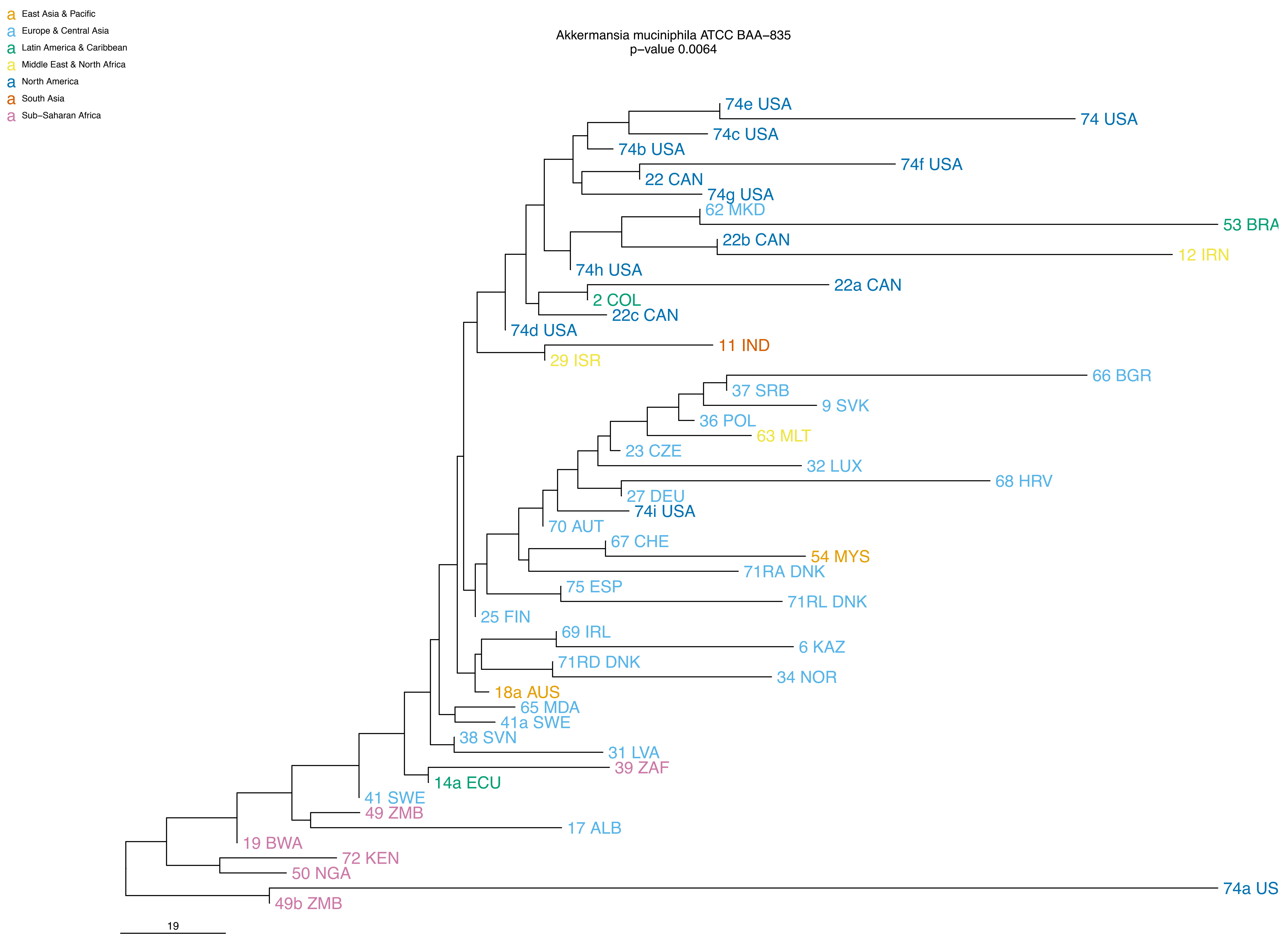

a East Asia & Pacific
a Europe & Central Asia
a Latin America & Caribbean
a Middle East & North Africa
a North America
a South Asia
a Sub–Saharan Africa
Akkermansia muciniphila ATCC BAA–835
p–value 0.0064
74e USA
74 USA
74c USA
74b USA
74f USA
22 CAN
74g USA
62 MKD
53 BRA
22b CAN
12 IRN
74h USA
22a CAN
2 COL
22c CAN
74d USA
11 IND
29 ISR
66 BGR
37 SRB
9 SVK
36 POL
63 MLT
23 CZE
32 LUX
68 HRV
27 DEU
74i USA
70 AUT
67 CHE
54 MYS
71RA DNK
75 ESP
71RL DNK
25 FIN
69 IRL
6 KAZ
71RD DNK
34 NOR
18a AUS
65 MDA
41a SWE
38 SVN
31 LVA
39 ZAF
14a ECU
41 SWE
49 ZMB
17 ALB
19 BWA
72 KEN
50 NGA
74a US
49b ZMB
19

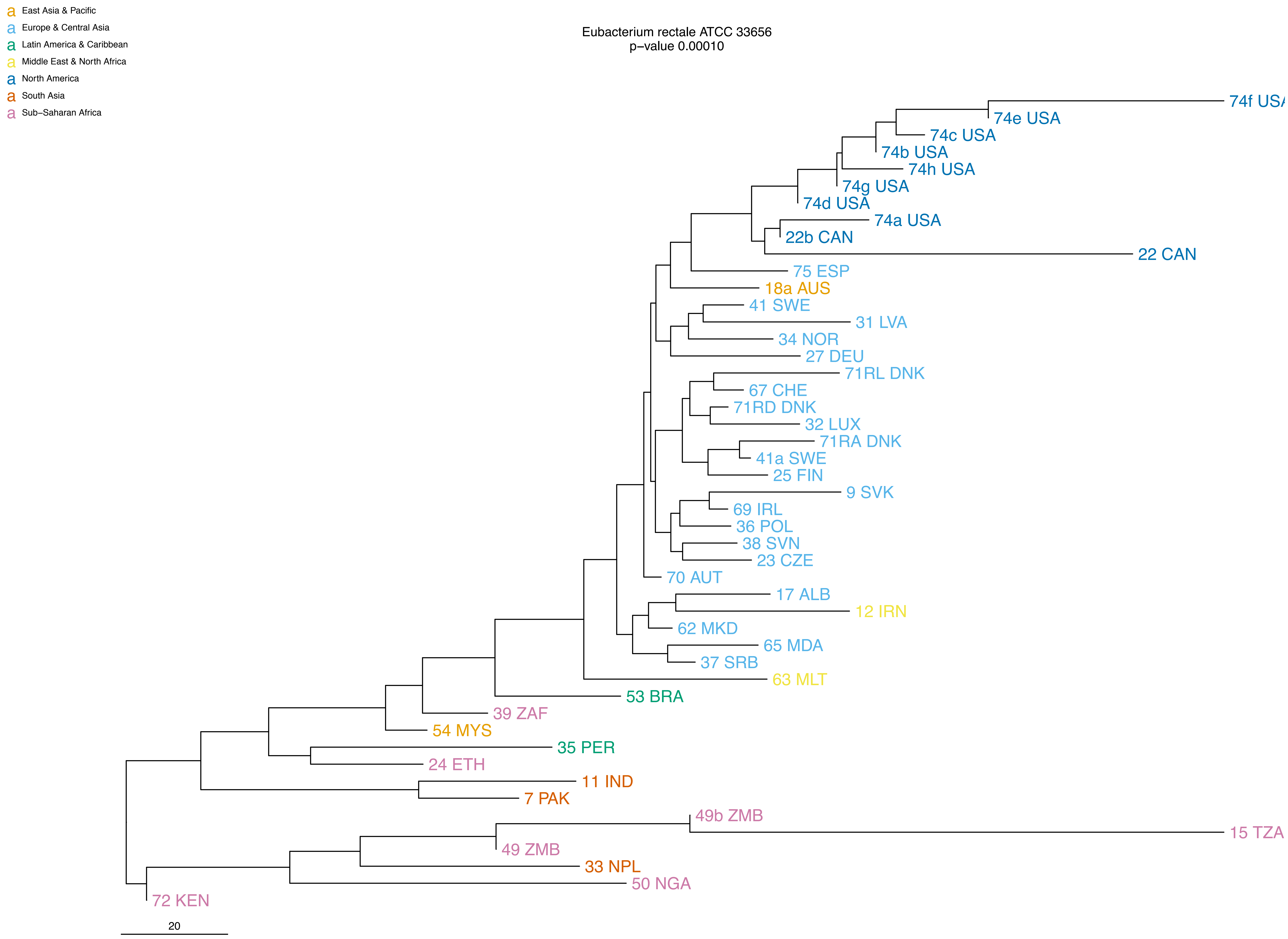
a East Asia & Pacific
a Europe & Central Asia
a Latin America & Caribbean
a Middle East & North Africa
a North America
a South Asia
a Sub−Saharan Africa
Eubacterium rectale ATCC 33656
p−value 0.00010
74f USA
74e USA
74c USA
74b USA
74h USA
74g USA
74d USA
74a USA
22b CAN
22 CAN
75 ESP
18a AUS
41 SWE
31 LVA
34 NOR
27 DEU
71RL DNK
67 CHE
71RD DNK
32 LUX
71RA DNK
41a SWE
25 FIN
9 SVK
69 IRL
36 POL
38 SVN
23 CZE
70 AUT
17 ALB
12 IRN
62 MKD
65 MDA
37 SRB
63 MLT
53 BRA
39 ZAF
54 MYS
35 PER
24 ETH
11 IND
7 PAK
49b ZMB
15 TZA
49 ZMB
33 NPL
50 NGA
72 KEN
20

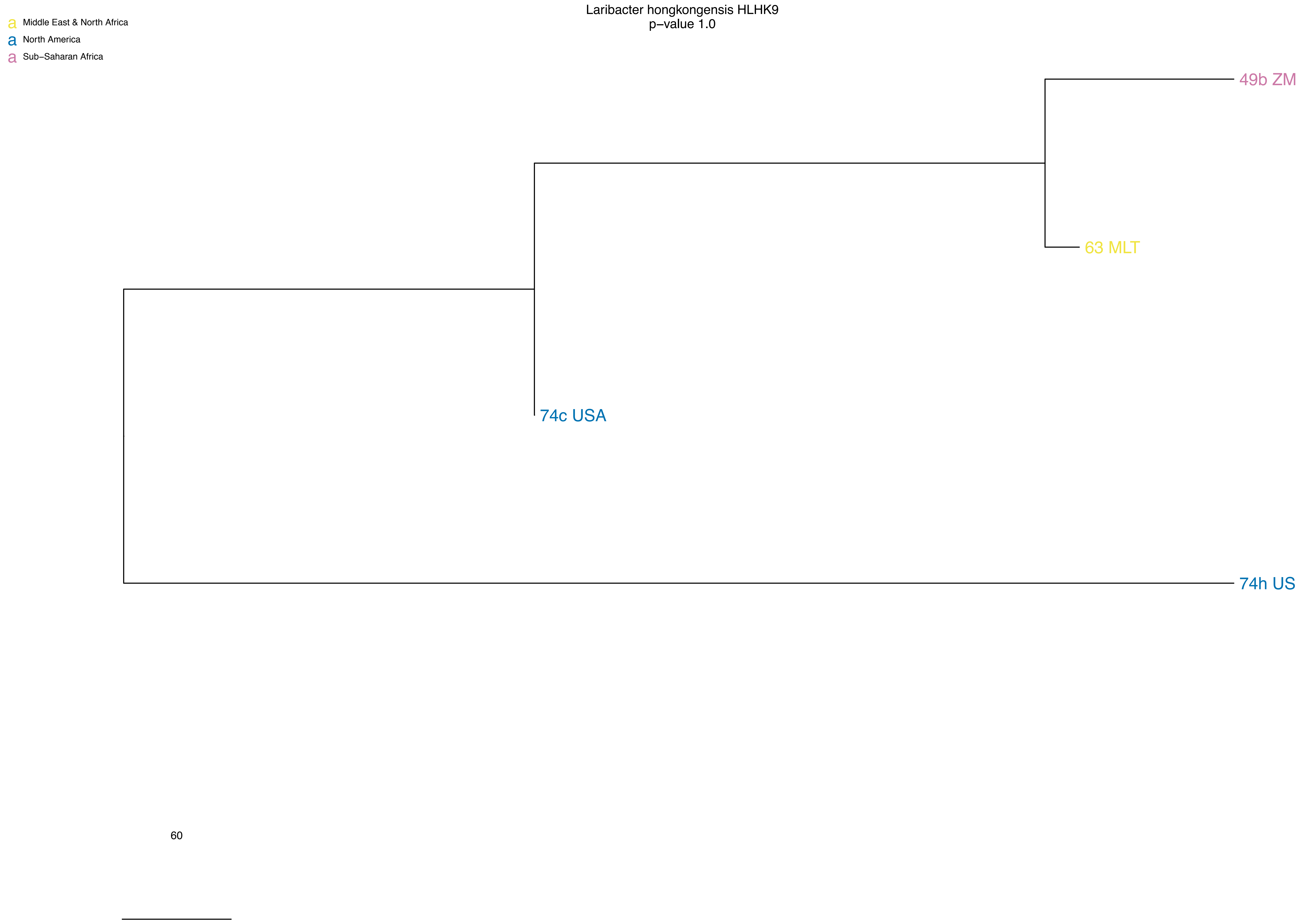

Laribacter hongkongensis HLHK9
p–value 1.0
a Middle East & North Africa
a North America
a Sub–Saharan Africa
49b ZM
63 MLT
74c USA
74h US
60

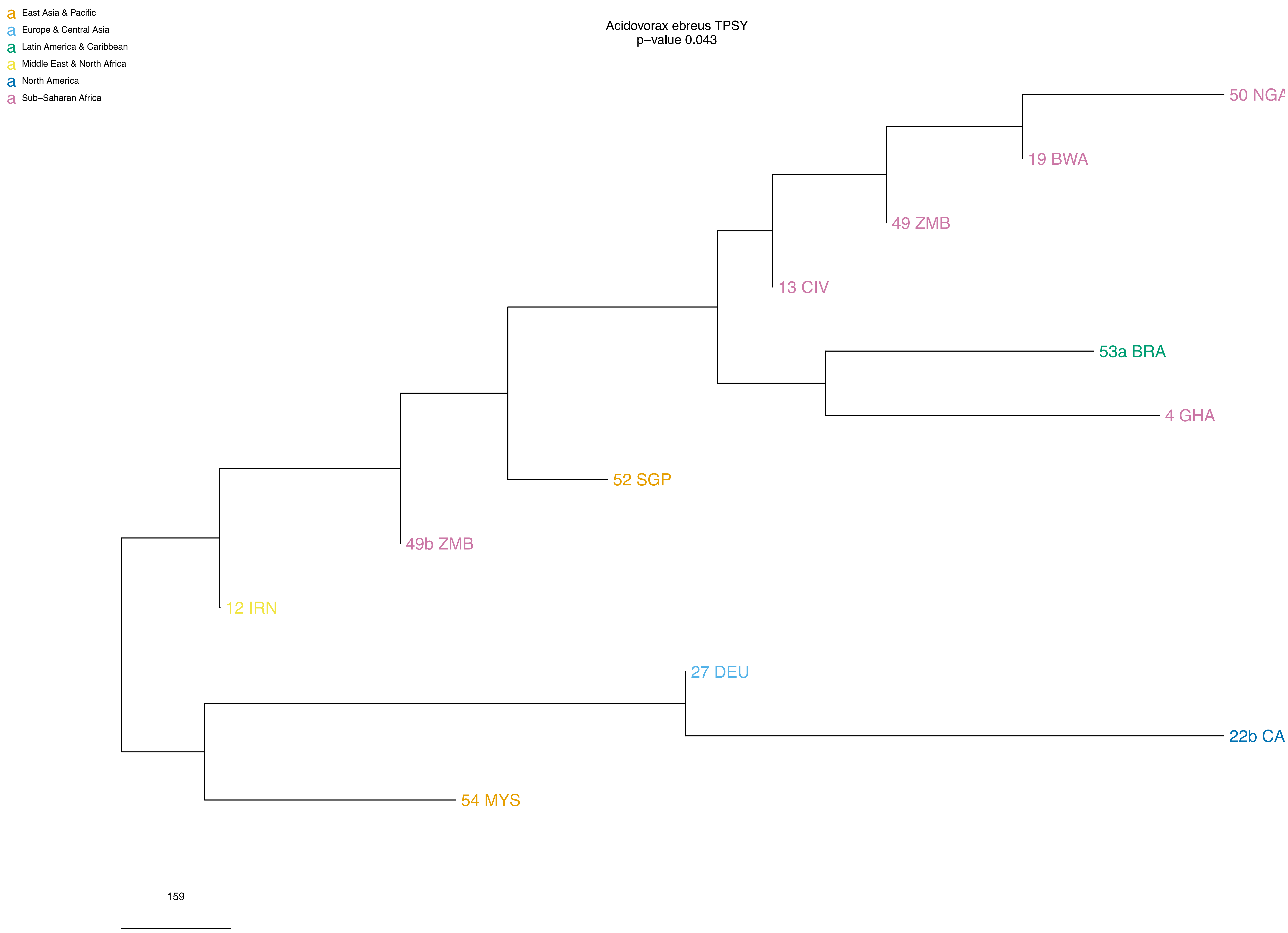

East Asia & Pacific
Europe & Central Asia
Latin America & Caribbean
Middle East & North Africa
North America
Sub–Saharan Africa
Acidovorax ebreus TPSY
p–value 0.043
50 NGA
19 BWA
49 ZMB
13 CIV
53a BRA
4 GHA
52 SGP
49b ZMB
12 IRN
27 DEU
22b CA
54 MYS
159

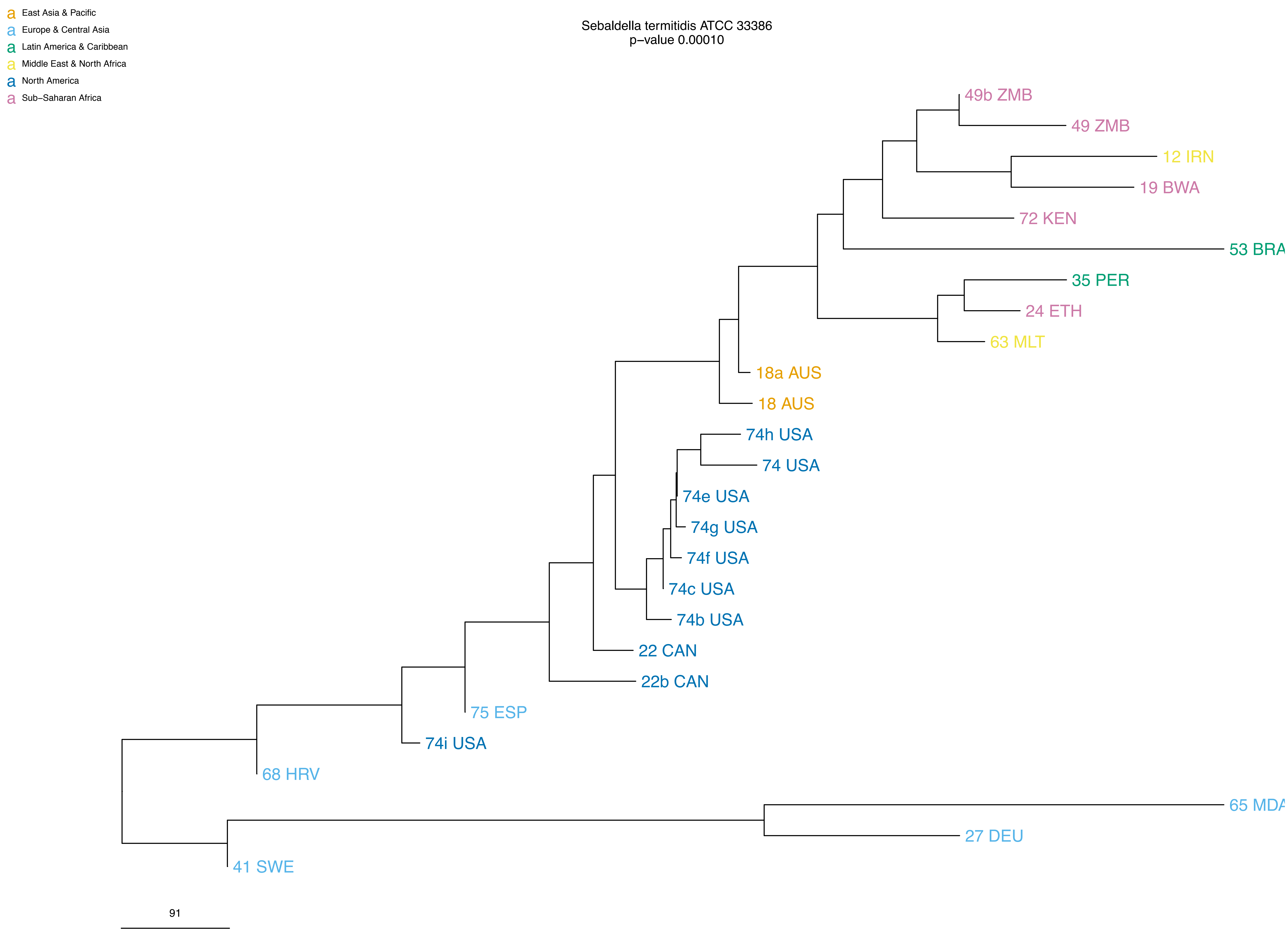

a East Asia & Pacific
a Europe & Central Asia
a Latin America & Caribbean
a Middle East & North Africa
a North America
a Sub–Saharan Africa
Sebaldella termitidis ATCC 33386
p–value 0.00010
49b ZMB
49 ZMB
12 IRN
19 BWA
72 KEN
53 BRA
35 PER
24 ETH
63 MLT
18a AUS
18 AUS
74h USA
74 USA
74e USA
74g USA
74f USA
74c USA
74b USA
22 CAN
22b CAN
75 ESP
74i USA
68 HRV
65 MDA
27 DEU
41 SWE
91

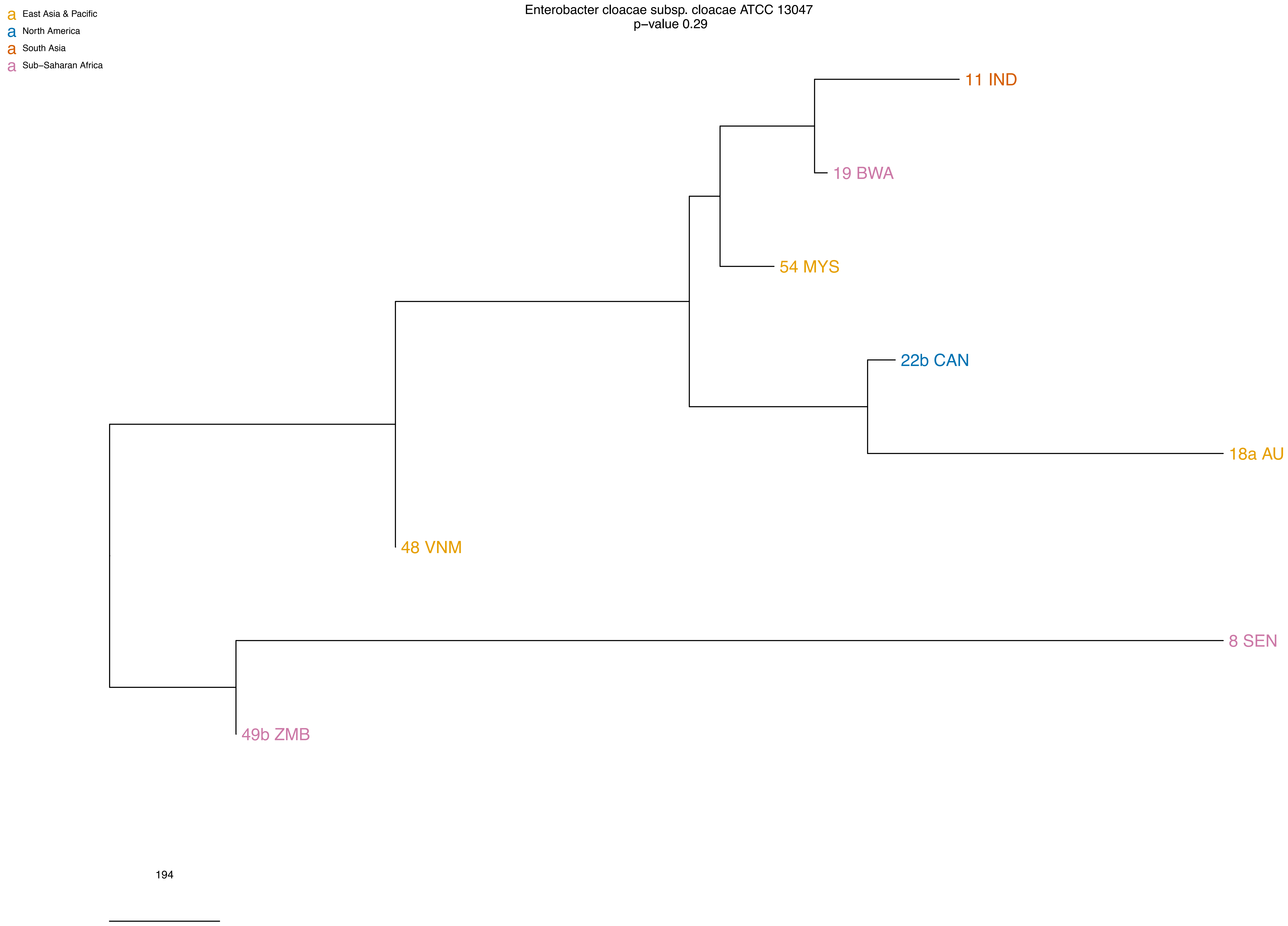

Enterobacter cloacae subsp. cloacae ATCC 13047
p−value 0.29
a East Asia & Pacific
a North America
a South Asia
a Sub−Saharan Africa
11 IND
19 BWA
54 MYS
22b CAN
18a AU
48 VNM
8 SEN
49b ZMB
194

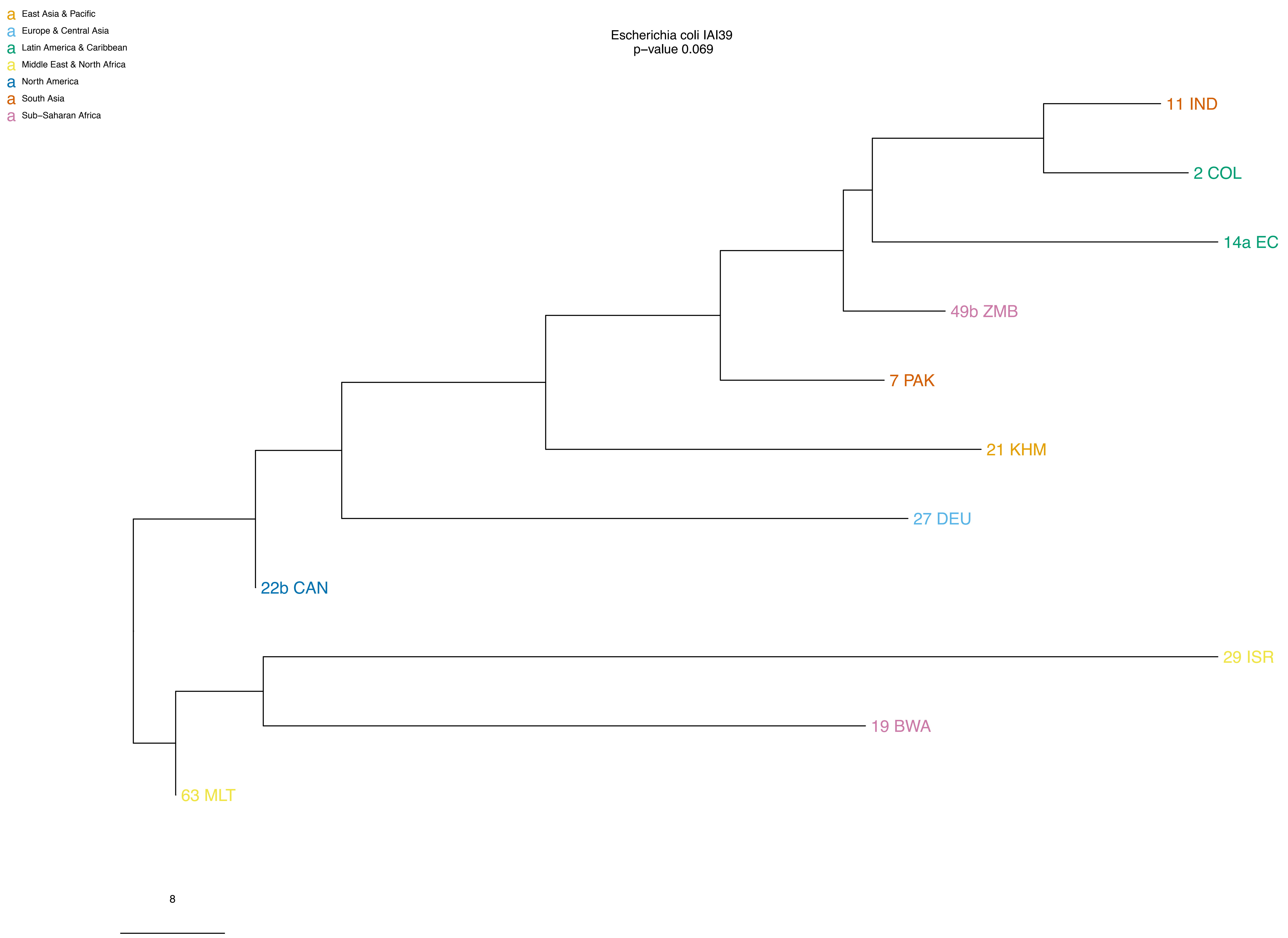

East Asia & Pacific
Europe & Central Asia
Latin America & Caribbean
Middle East & North Africa
North America
South Asia
Sub–Saharan Africa
Escherichia coli IAI39
p–value 0.069
11 IND
2 COL
14a EC
49b ZMB
7 PAK
21 KHM
27 DEU
22b CAN
29 ISR
19 BWA
63 MLT
8

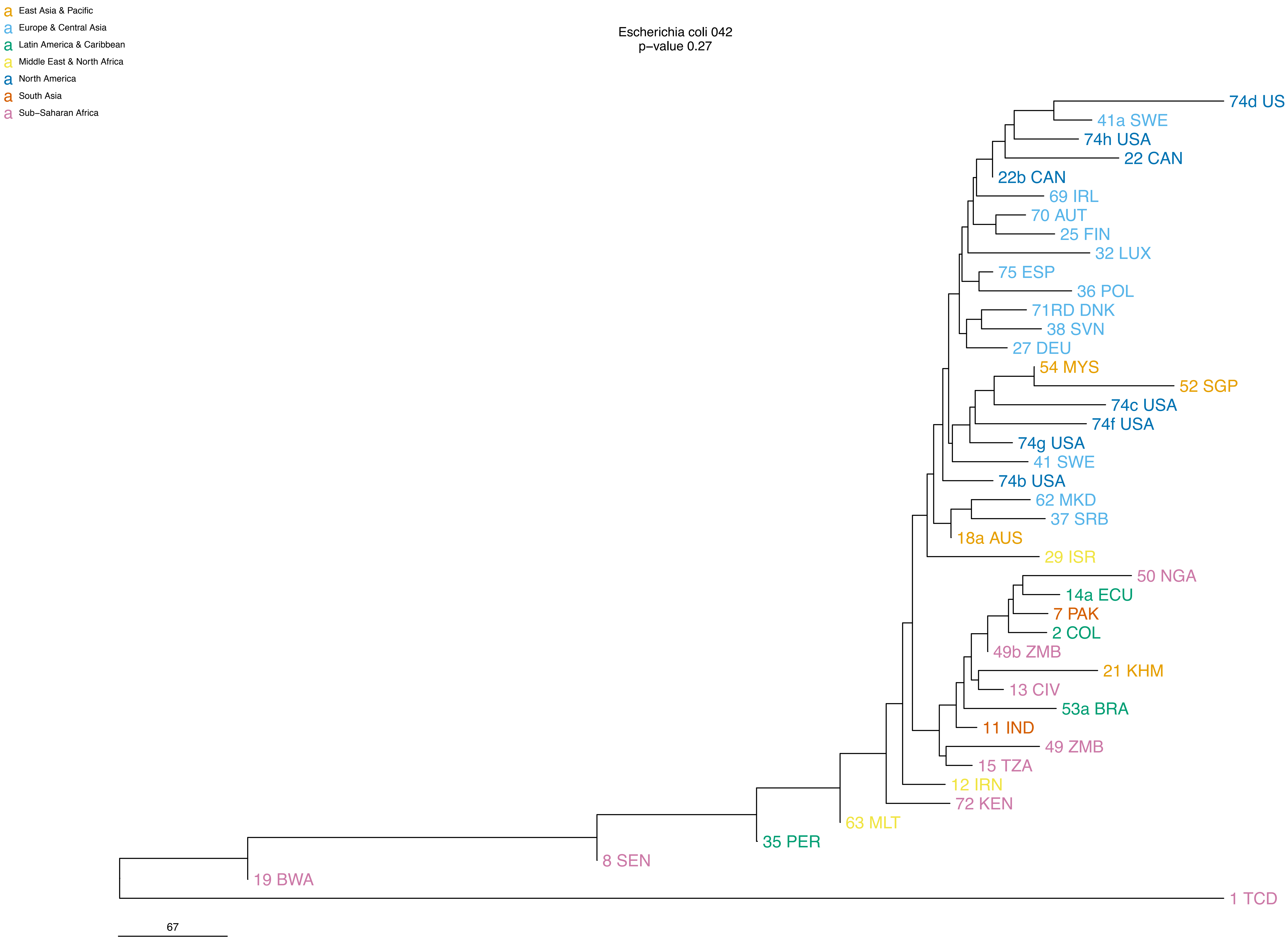

East Asia & Pacific
Europe & Central Asia
Latin America & Caribbean
Middle East & North Africa
North America
South Asia
Sub–Saharan Africa
Escherichia coli 042
p–value 0.27
74d US
41a SWE
74h USA
22 CAN
22b CAN
69 IRL
70 AUT
25 FIN
32 LUX
75 ESP
36 POL
71RD DNK
38 SVN
27 DEU
54 MYS
52 SGP
74c USA
74f USA
74g USA
41 SWE
74b USA
62 MKD
37 SRB
18a AUS
29 ISR
50 NGA
14a ECU
7 PAK
2 COL
49b ZMB
21 KHM
13 CIV
53a BRA
11 IND
49 ZMB
15 TZA
12 IRN
72 KEN
63 MLT
35 PER
8 SEN
19 BWA
1 TCD
67

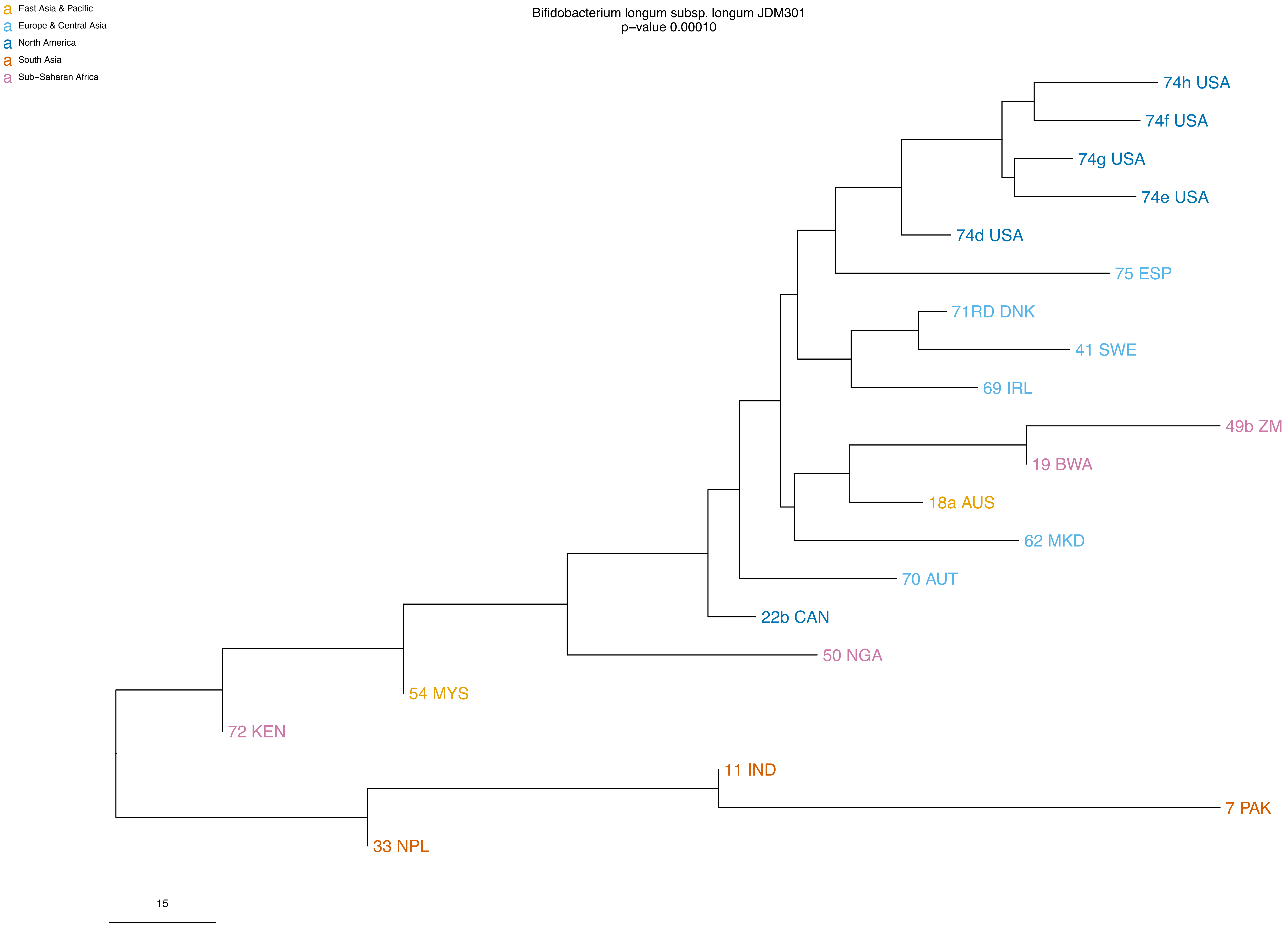

East Asia & Pacific
Europe & Central Asia
North America
South Asia
Sub-Saharan Africa
Bifidobacterium longum subsp. longum JDM301
p−value 0.00010
74h USA
74f USA
74g USA
74e USA
74d USA
75 ESP
71RD DNK
41 SWE
69 IRL
49b ZM
19 BWA
18a AUS
62 MKD
70 AUT
22b CAN
50 NGA
54 MYS
72 KEN
11 IND
7 PAK
33 NPL
15

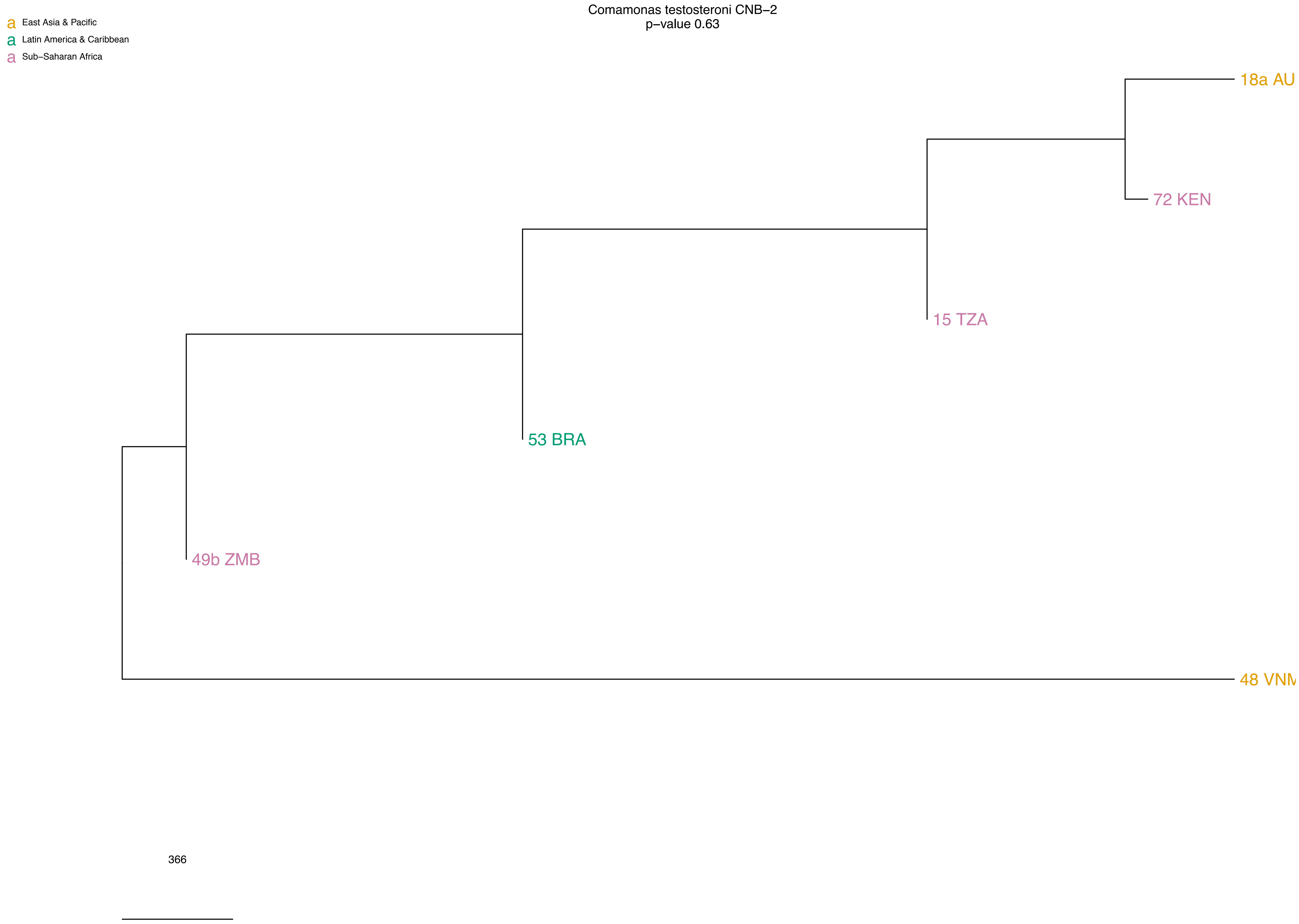
East Asia & Pacific
Latin America & Caribbean
Sub−Saharan Africa
Comamonas testosteroni CNB−2
p−value 0.63
18a AU
72 KEN
15 TZA
53 BRA
49b ZMB
48 VNM
366

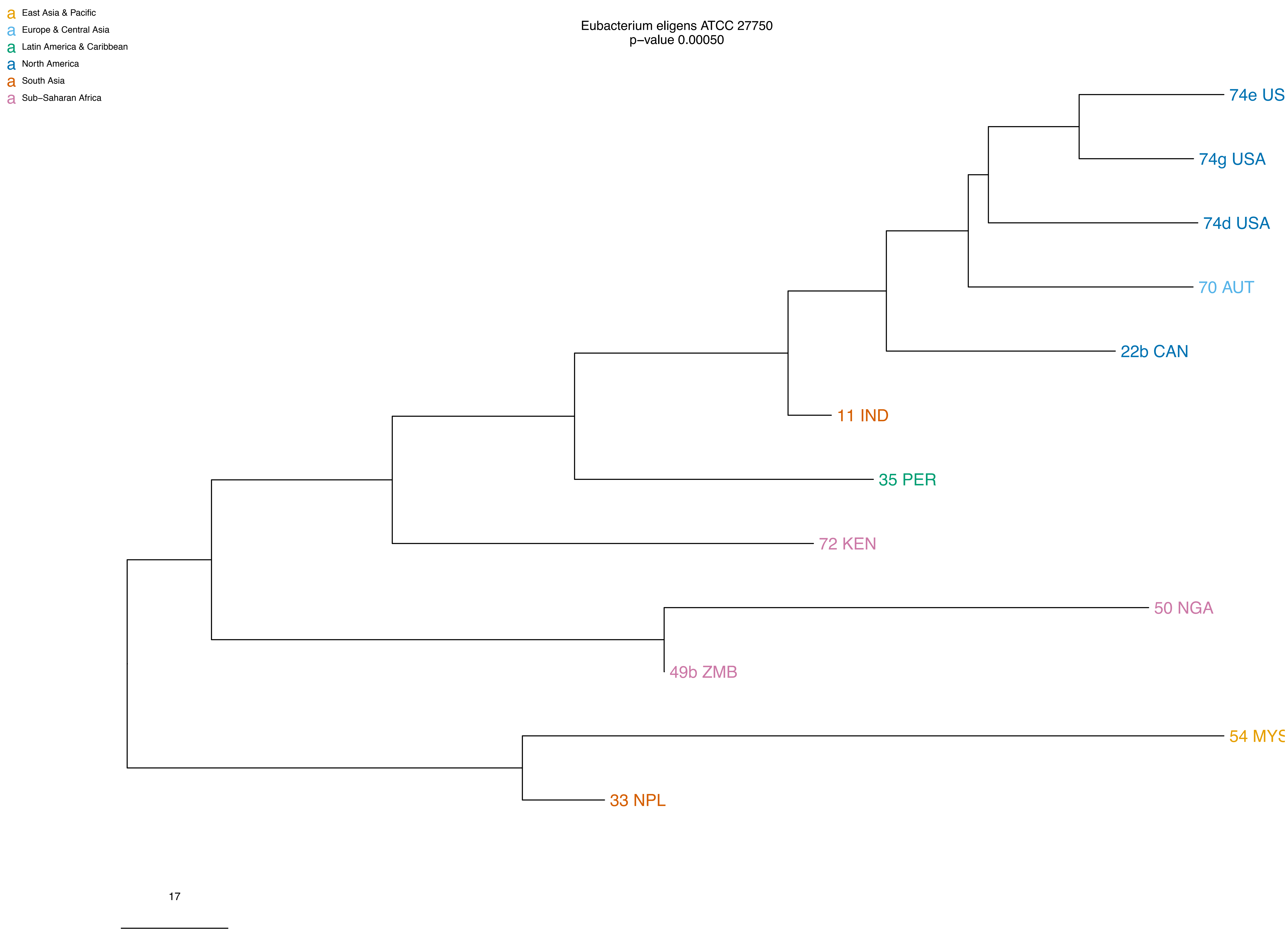

East Asia & Pacific
Europe & Central Asia
Latin America & Caribbean
North America
South Asia
Sub−Saharan Africa
Eubacterium eligens ATCC 27750
p−value 0.00050
74e US
74g USA
74d USA
70 AUT
22b CAN
11 IND
35 PER
72 KEN
50 NGA
49b ZMB
54 MYS
33 NPL
17

Lactobacillus delbrueckii subsp. bulgaricus ND02
p–value 1.0

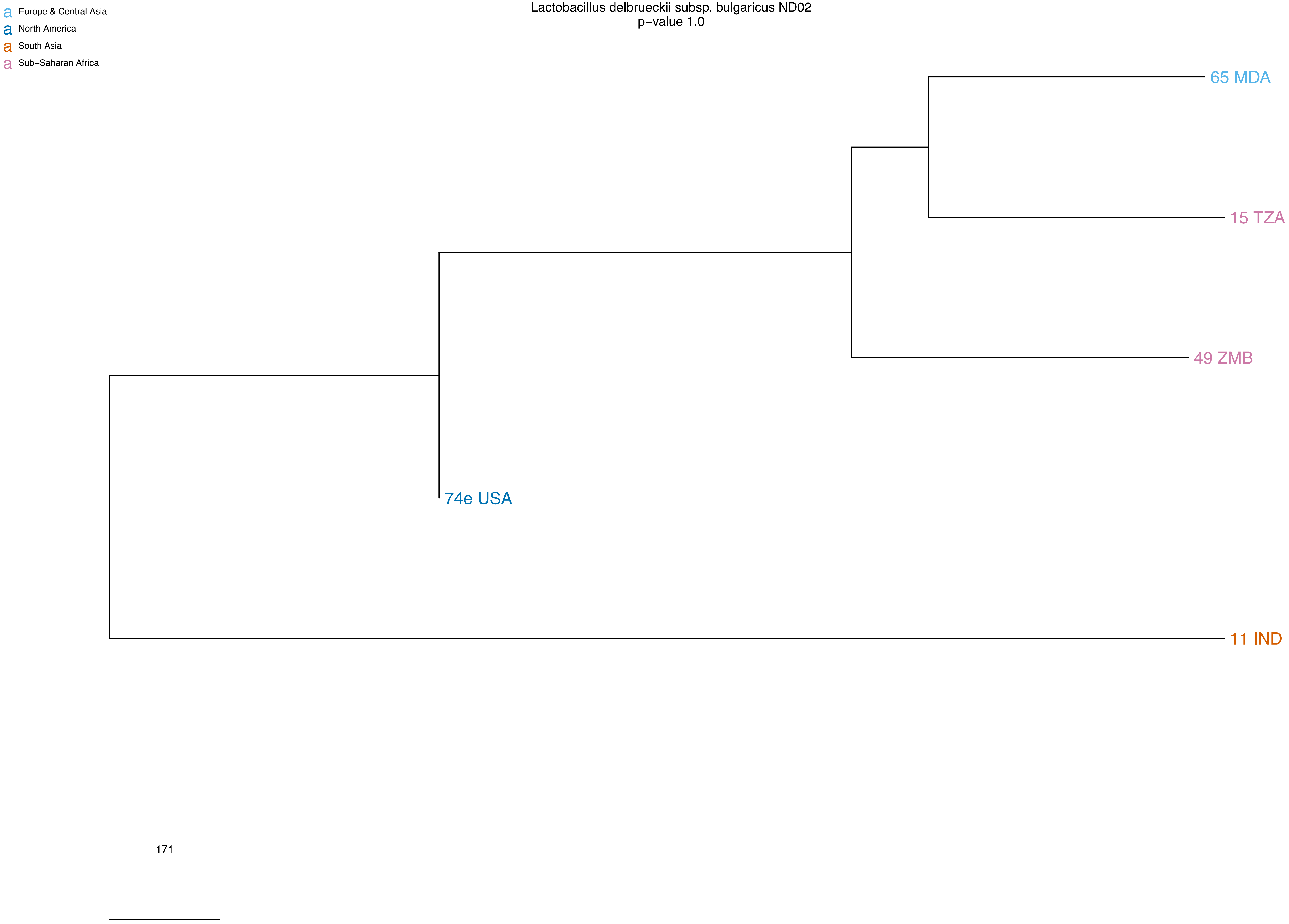

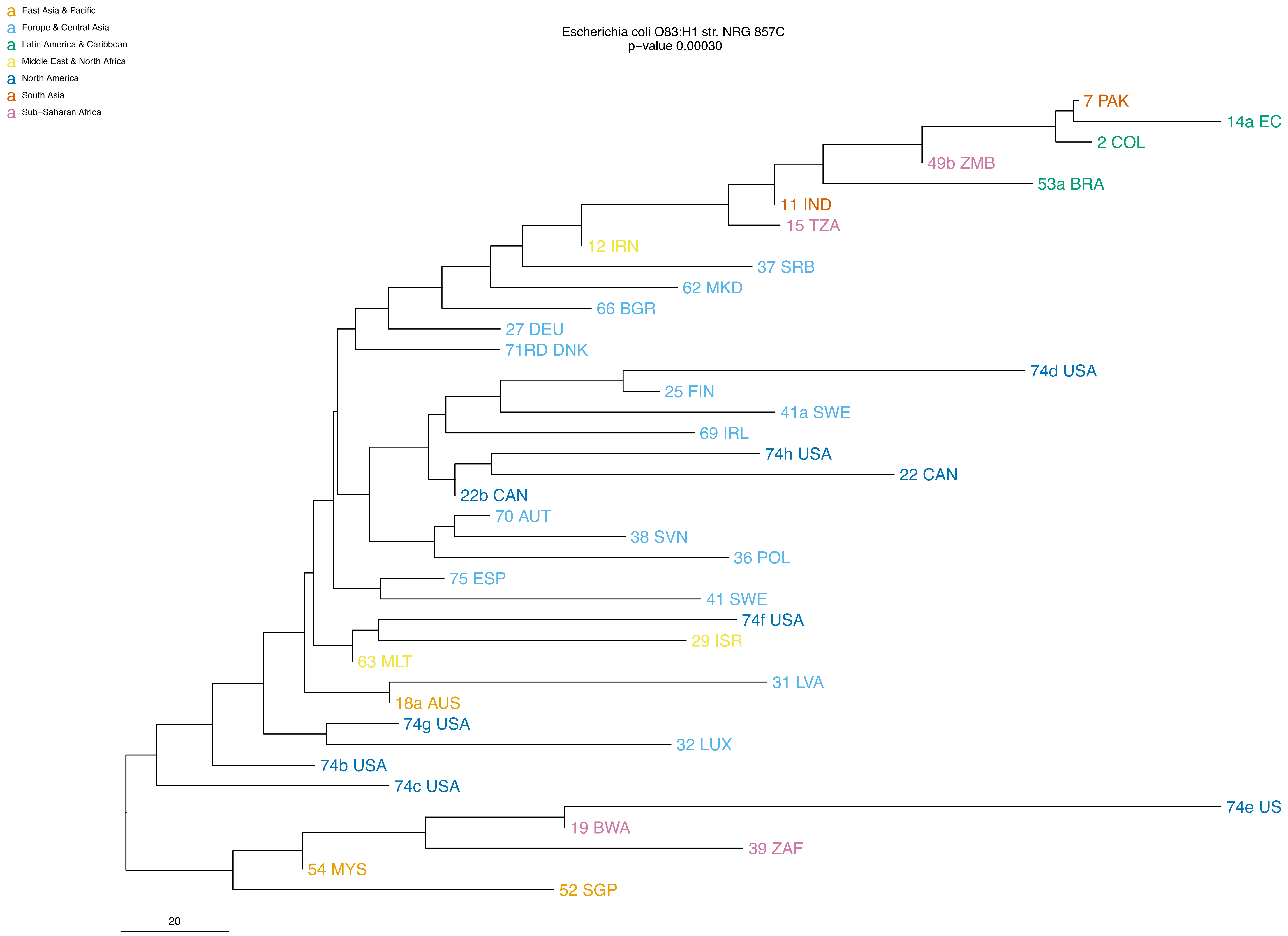

East Asia & Pacific
Europe & Central Asia
Latin America & Caribbean
Middle East & North Africa
North America
South Asia
Sub–Saharan Africa
Escherichia coli O83:H1 str. NRG 857C
p–value 0.00030
7 PAK
14a EC
2 COL
49b ZMB
53a BRA
11 IND
15 TZA
12 IRN
37 SRB
62 MKD
66 BGR
27 DEU
71RD DNK
74d USA
25 FIN
41a SWE
69 IRL
74h USA
22 CAN
22b CAN
70 AUT
38 SVN
36 POL
75 ESP
41 SWE
74f USA
29 ISR
63 MLT
31 LVA
18a AUS
74g USA
32 LUX
74b USA
74c USA
74e US
19 BWA
39 ZAF
54 MYS
52 SGP
20

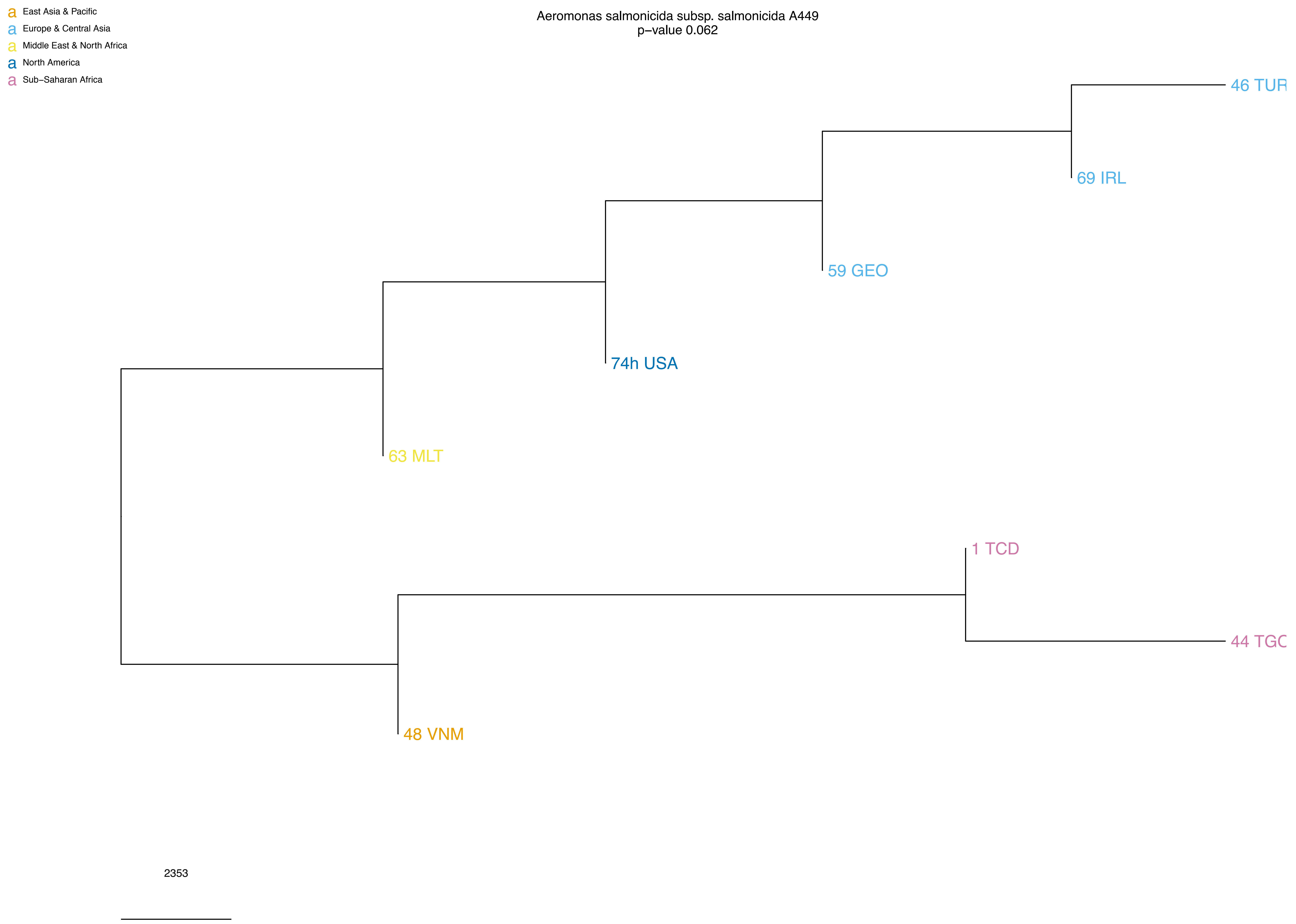

East Asia & Pacific
Europe & Central Asia
Middle East & North Africa
North America
Sub–Saharan Africa
Aeromonas salmonicida subsp. salmonicida A449
p–value 0.062
46 TUR
69 IRL
59 GEO
74h USA
63 MLT
1 TCD
44 TGO
48 VNM
2353

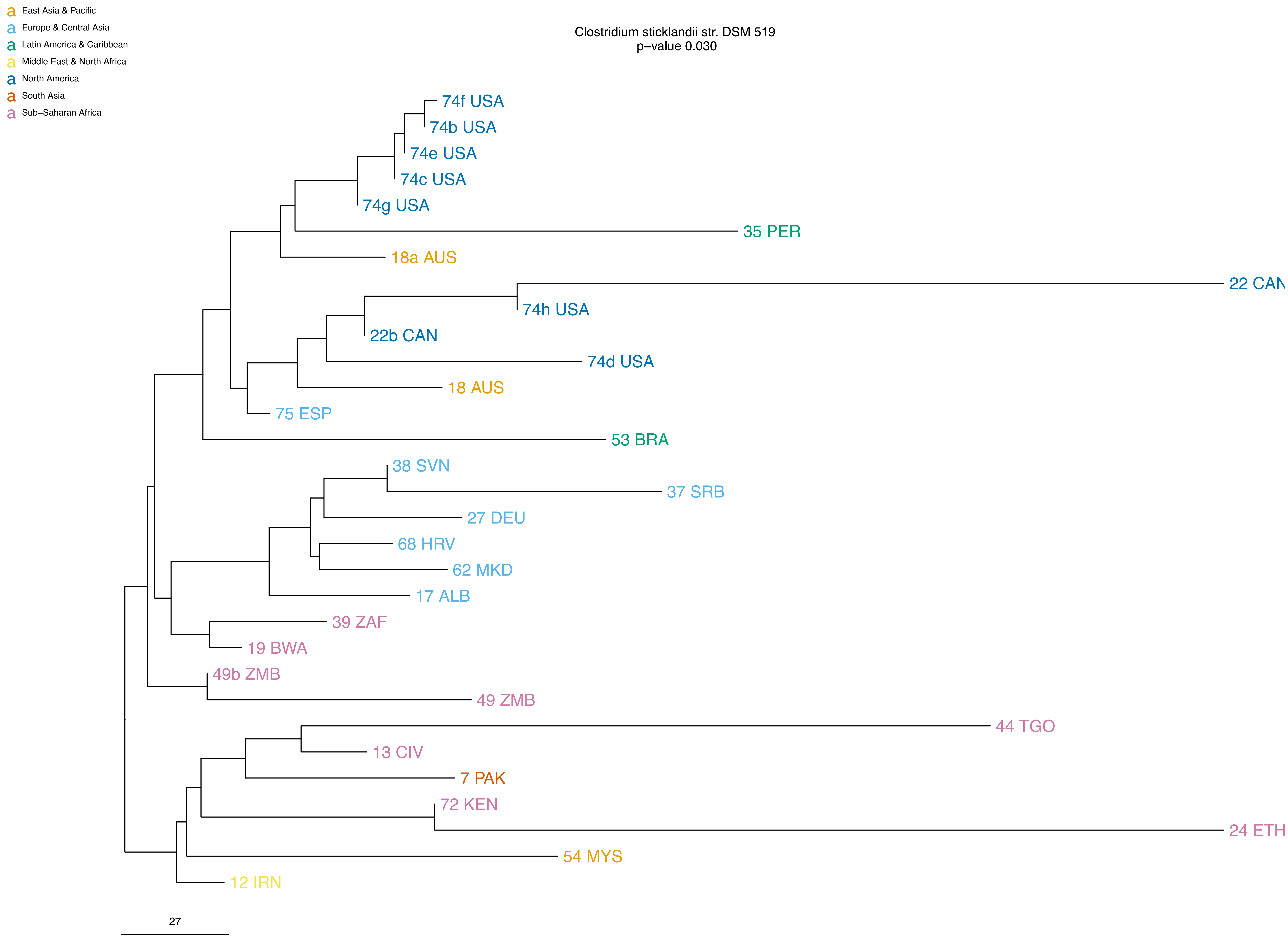

East Asia & Pacific
Europe & Central Asia
Latin America & Caribbean
Middle East & North Africa
North America
South Asia
Sub–Saharan Africa
Clostridium sticklandii str. DSM 519
p–value 0.030
74f USA
74b USA
74e USA
74c USA
74g USA
35 PER
18a AUS
22 CAN
74h USA
22b CAN
74d USA
18 AUS
75 ESP
53 BRA
38 SVN
37 SRB
27 DEU
68 HRV
62 MKD
17 ALB
39 ZAF
19 BWA
49b ZMB
49 ZMB
44 TGO
13 CIV
7 PAK
72 KEN
24 ETH
54 MYS
12 IRN
27

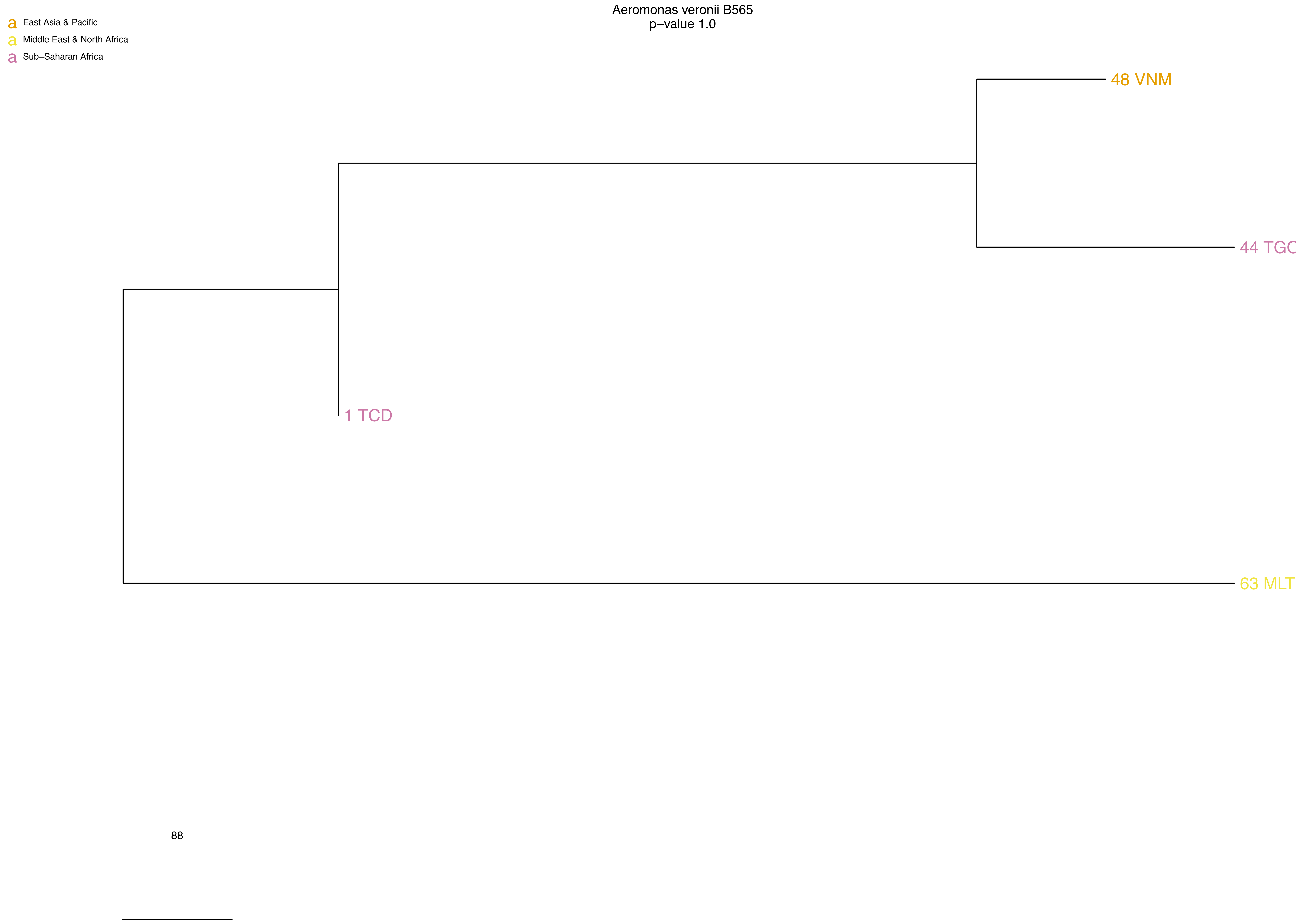
Aeromonas veronii B565
p–value 1.0
East Asia & Pacific
Middle East & North Africa
Sub–Saharan Africa
48 VNM
44 TGO
1 TCD
63 MLT
88

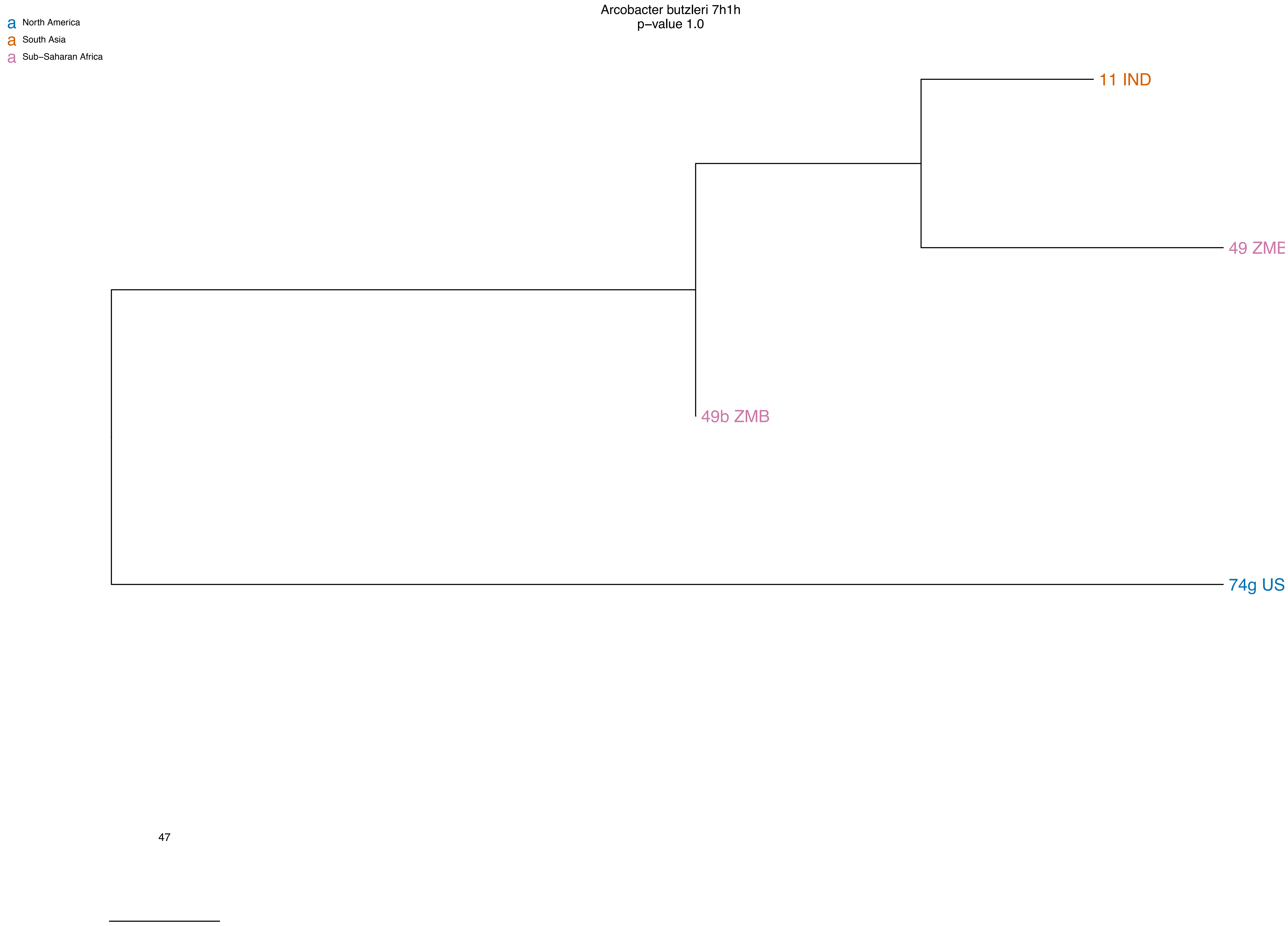

Arcobacter butzleri 7h1h
p–value 1.0
North America
South Asia
Sub–Saharan Africa
11 IND
49 ZMB
49b ZMB
74g US
47

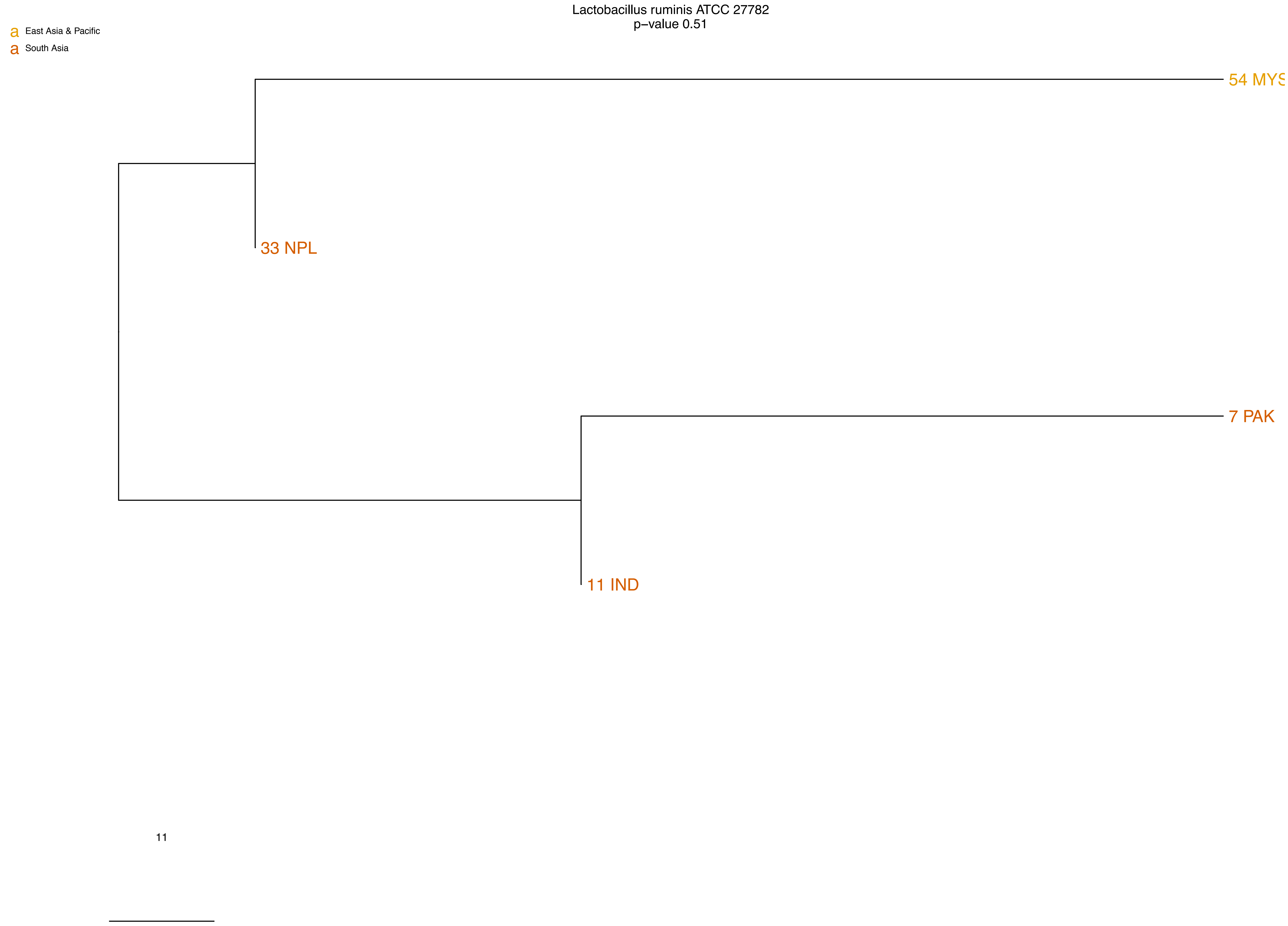

Lactobacillus ruminis ATCC 27782
p–value 0.51
a East Asia & Pacific
a South Asia
54 MYS
33 NPL
7 PAK
11 IND
11

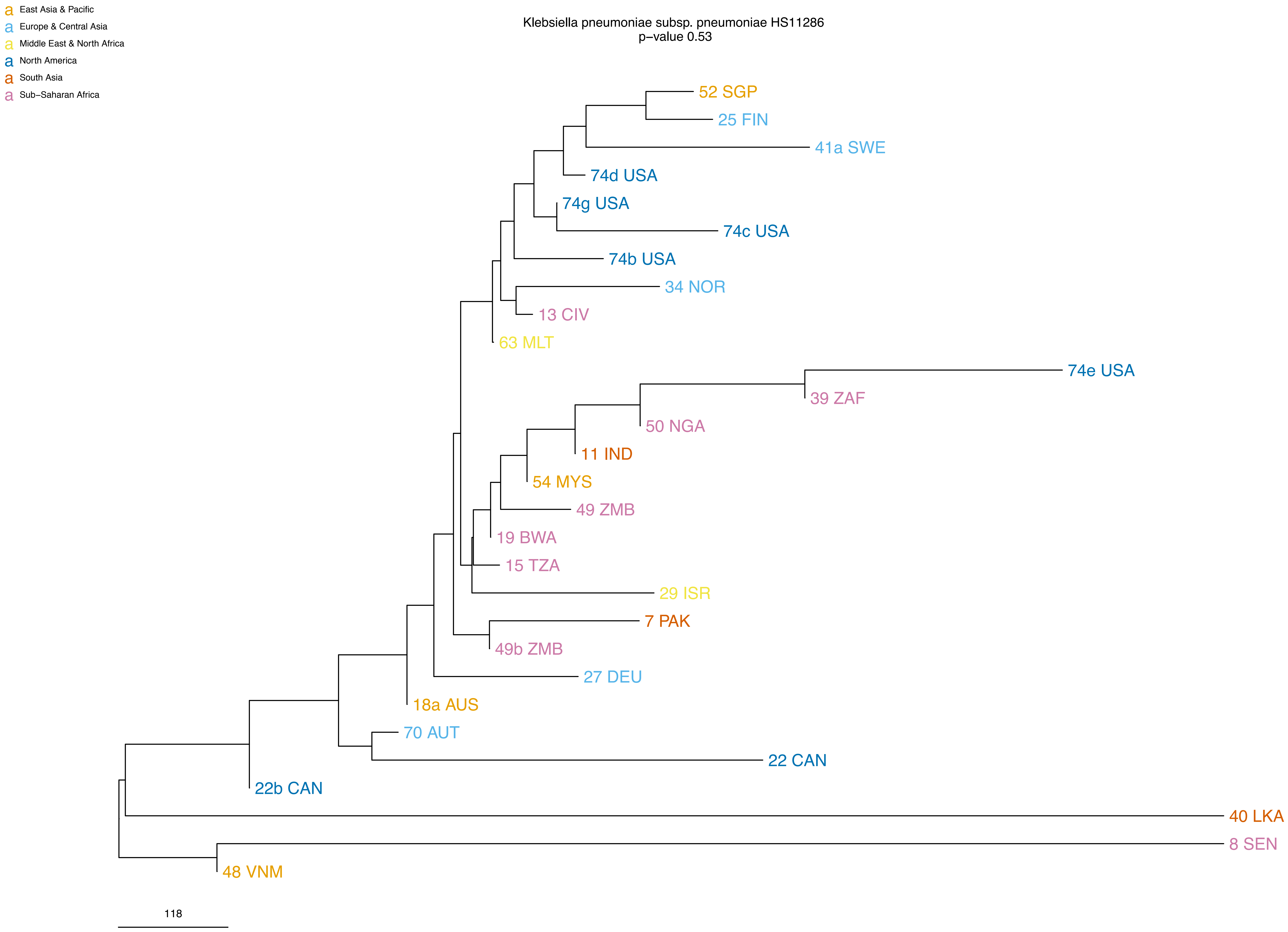

a East Asia & Pacific
a Europe & Central Asia
a Middle East & North Africa
a North America
a South Asia
a Sub–Saharan Africa
Klebsiella pneumoniae subsp. pneumoniae HS11286
p–value 0.53
52 SGP
25 FIN
41a SWE
74d USA
74g USA
74c USA
74b USA
34 NOR
13 CIV
63 MLT
74e USA
39 ZAF
50 NGA
11 IND
54 MYS
49 ZMB
19 BWA
15 TZA
29 ISR
7 PAK
49b ZMB
27 DEU
18a AUS
70 AUT
22 CAN
22b CAN
40 LKA
8 SEN
48 VNM
118

Streptococcus infantarius subsp. infantarius CJ18
p−value 1.0

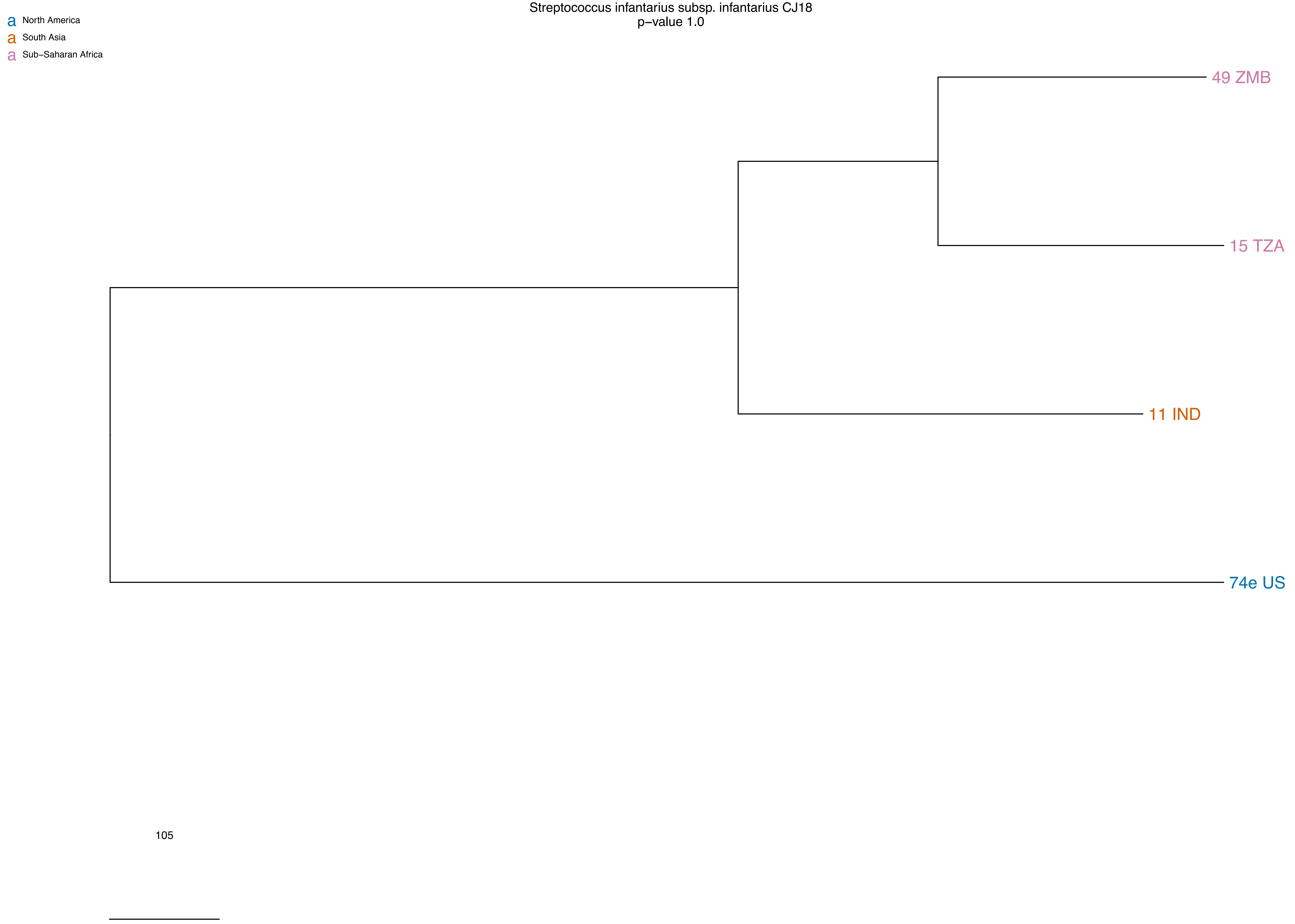

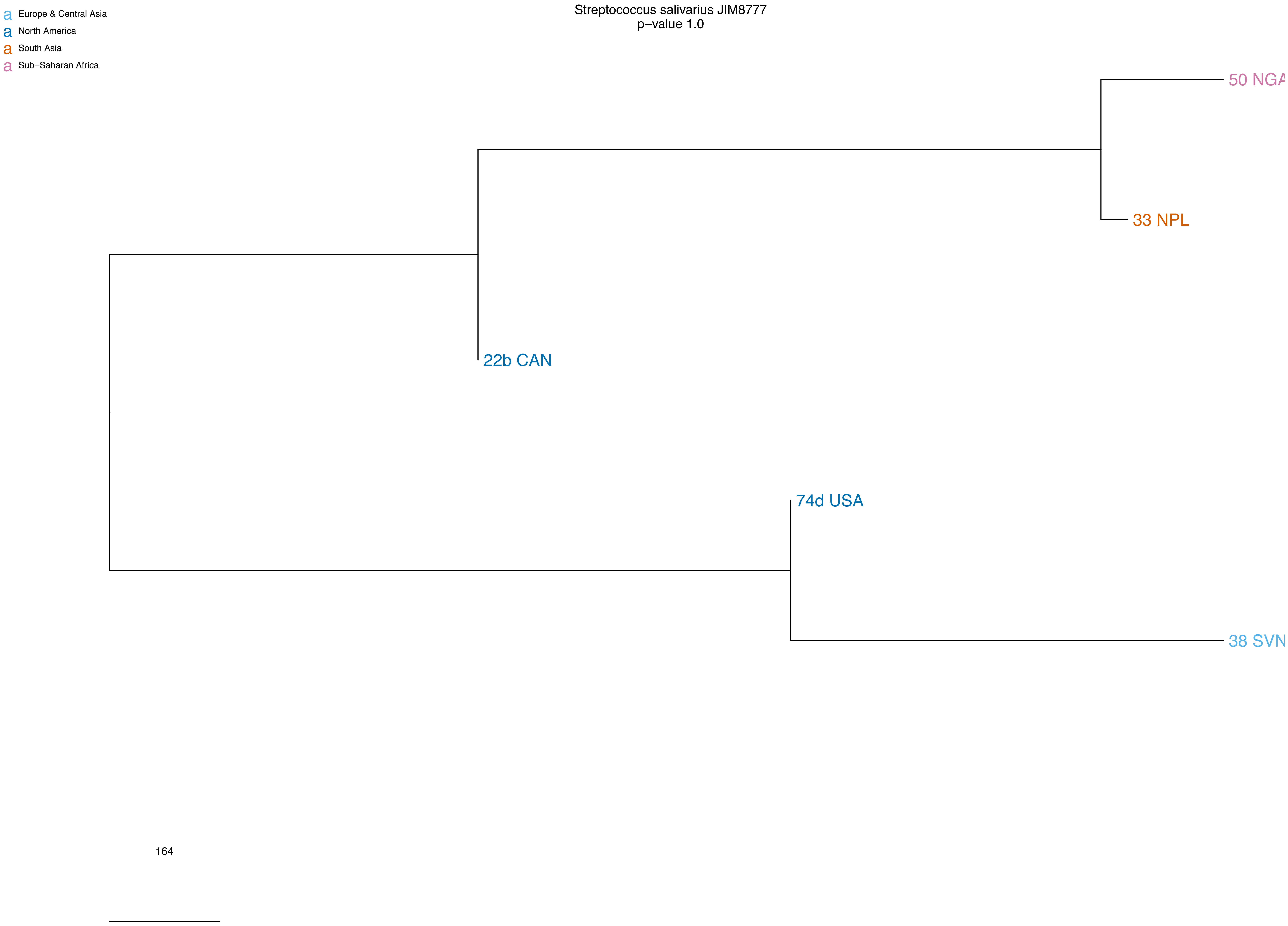

Streptococcus salivarius JIM8777
p–value 1.0
Europe & Central Asia
North America
South Asia
Sub–Saharan Africa
50 NGA
33 NPL
22b CAN
74d USA
38 SVN
164

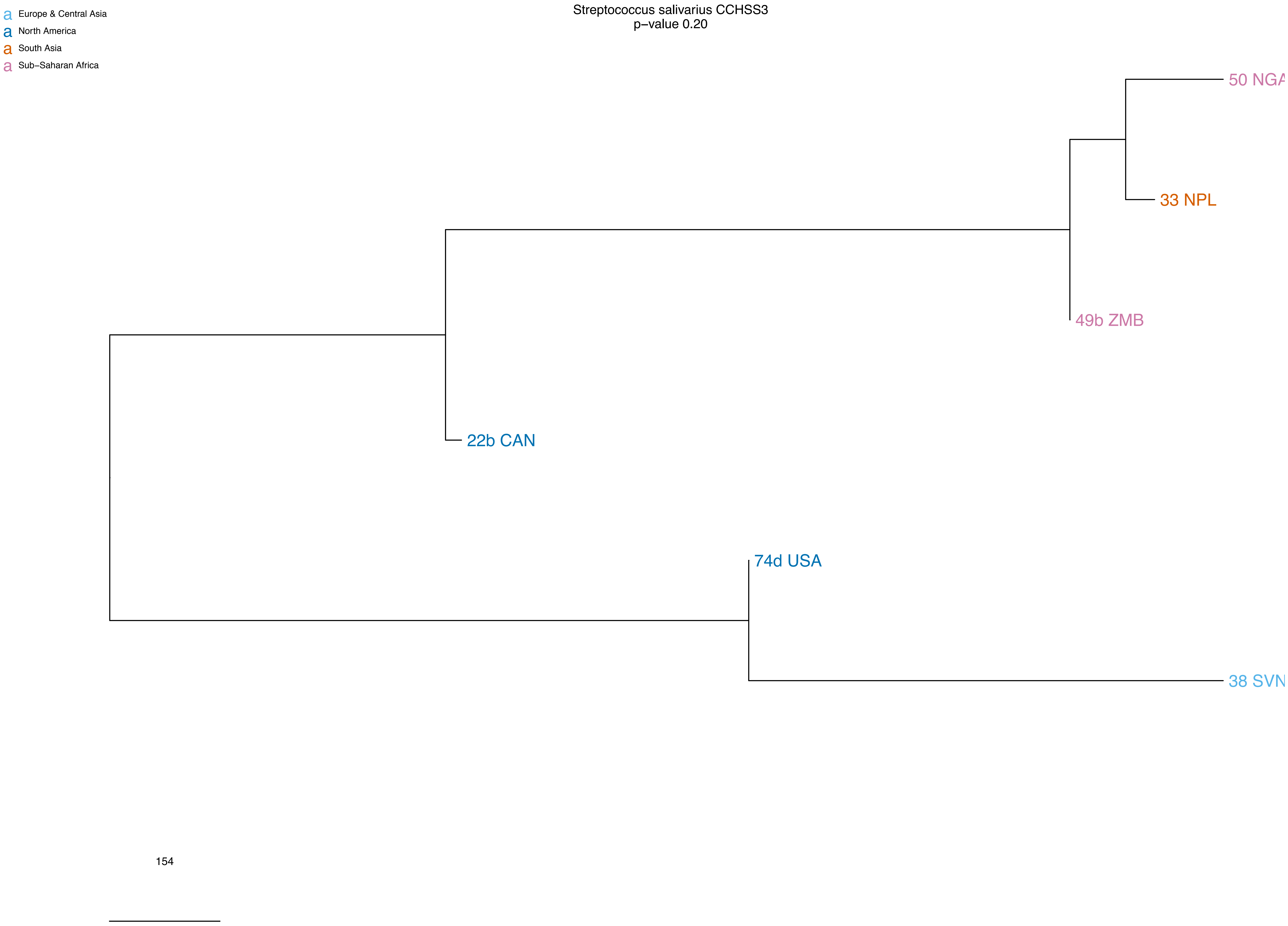

Europe & Central Asia
North America
South Asia
Sub–Saharan Africa
Streptococcus salivarius CCHSS3
p–value 0.20
50 NGA
33 NPL
49b ZMB
22b CAN
74d USA
38 SVN
154

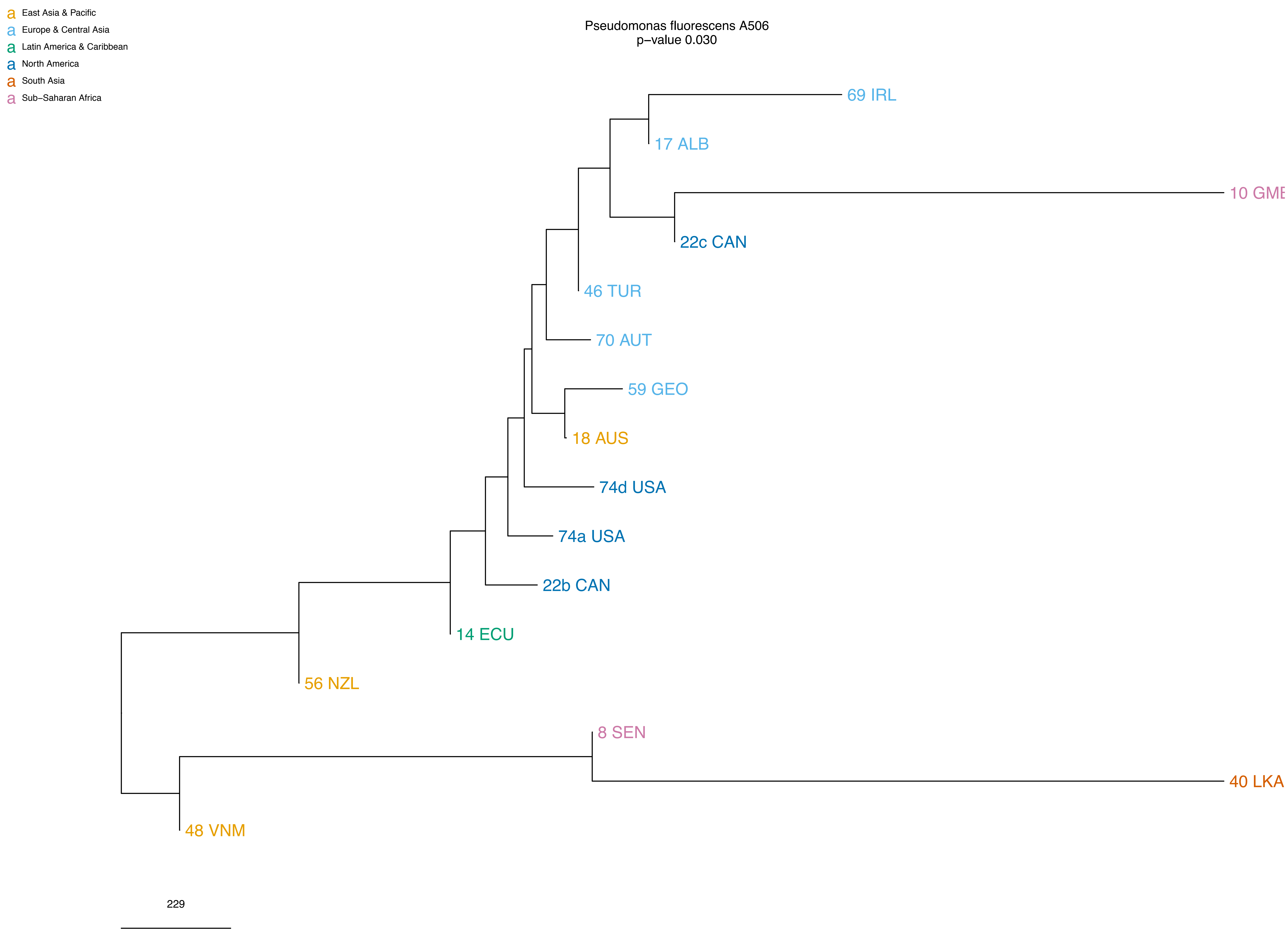

East Asia & Pacific
Europe & Central Asia
Latin America & Caribbean
North America
South Asia
Sub–Saharan Africa
Pseudomonas fluorescens A506
p–value 0.030
69 IRL
17 ALB
10 GMB
22c CAN
46 TUR
70 AUT
59 GEO
18 AUS
74d USA
74a USA
22b CAN
14 ECU
56 NZL
8 SEN
40 LKA
48 VNM
229

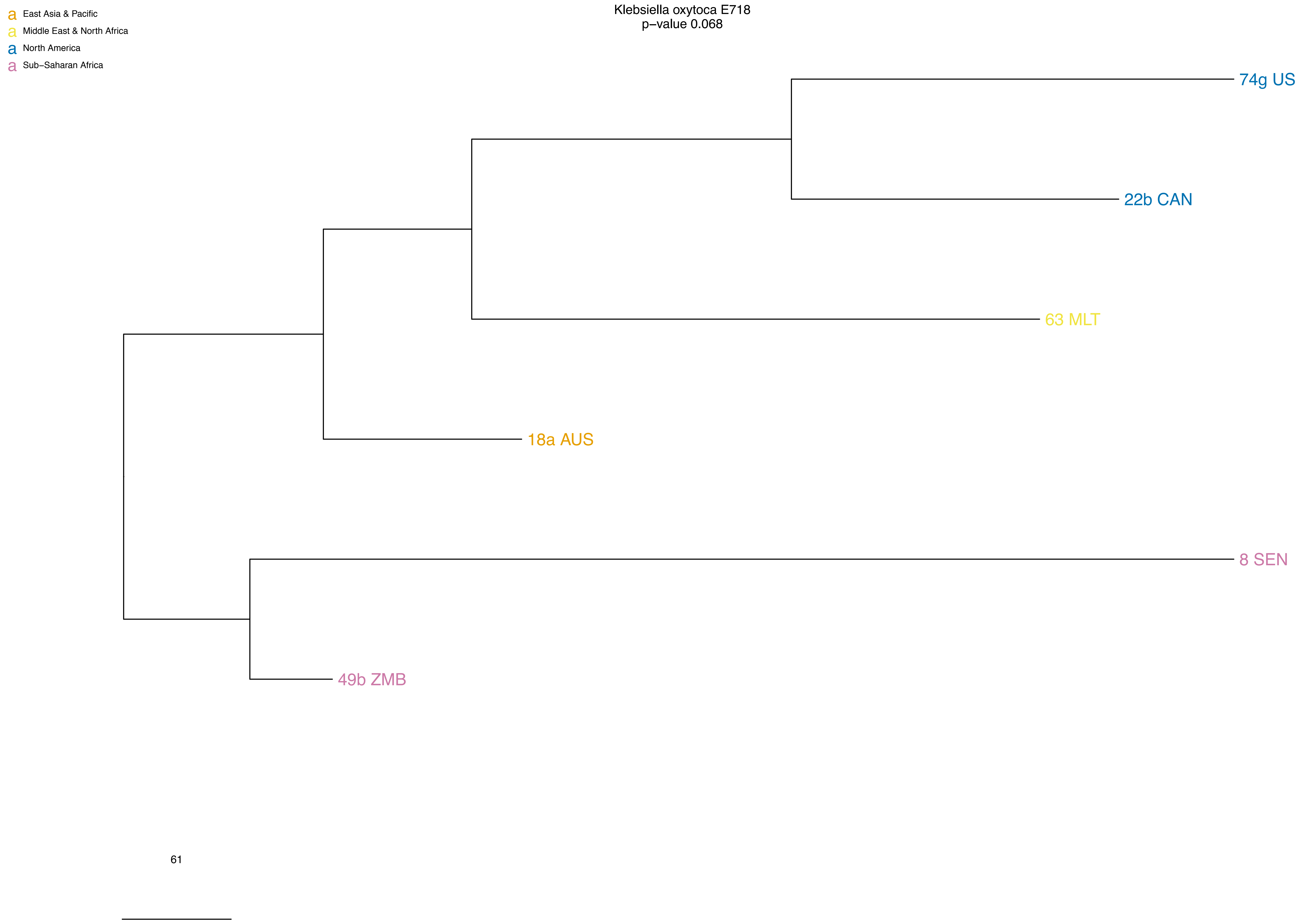
East Asia & Pacific
Middle East & North Africa
North America
Sub–Saharan Africa
Klebsiella oxytoca E718
p–value 0.068
74g US
22b CAN
63 MLT
18a AUS
8 SEN
49b ZMB
61

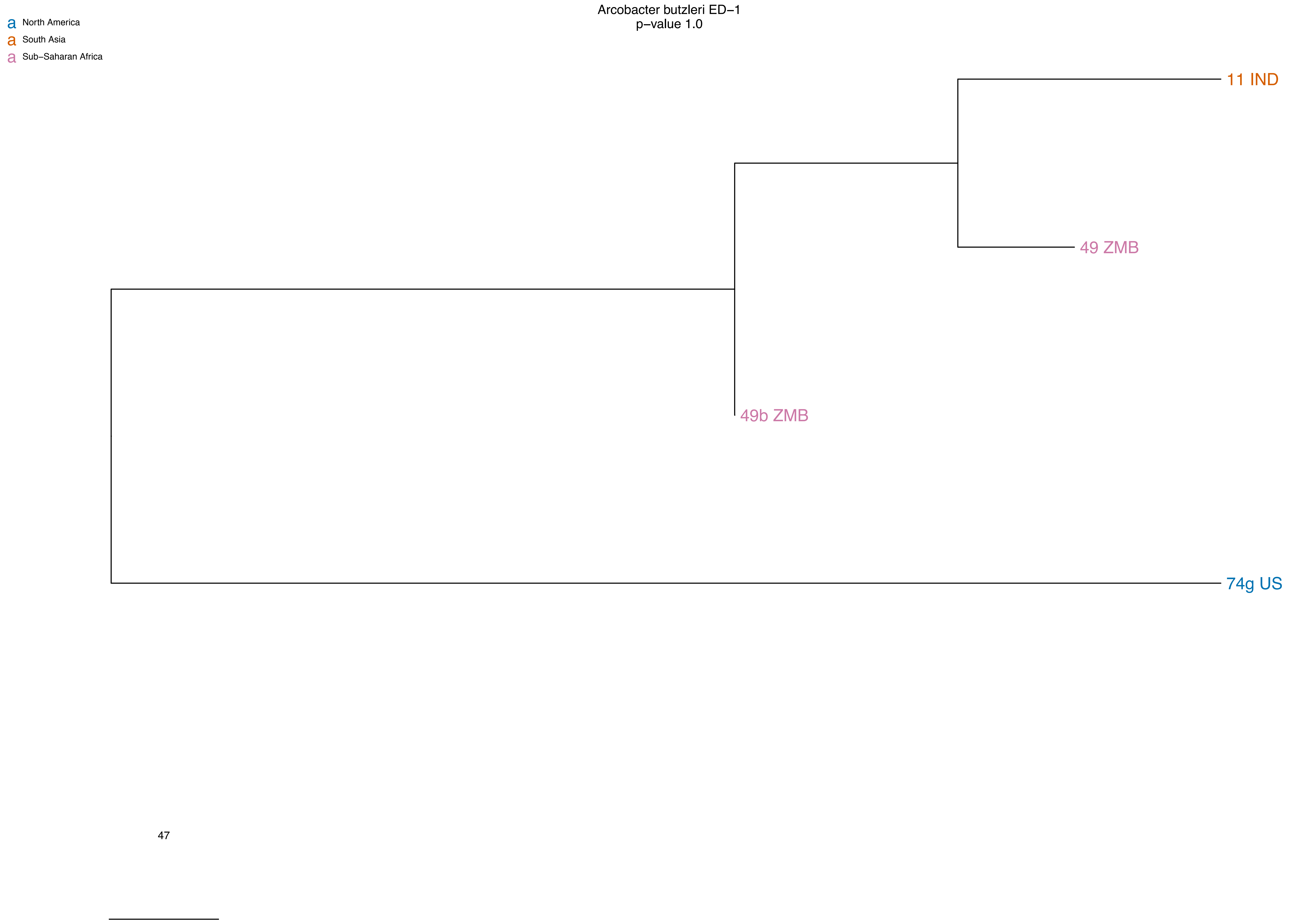
North America
South Asia
Sub–Saharan Africa
Arcobacter butzleri ED–1
p–value 1.0
11 IND
49 ZMB
49b ZMB
74g US
47

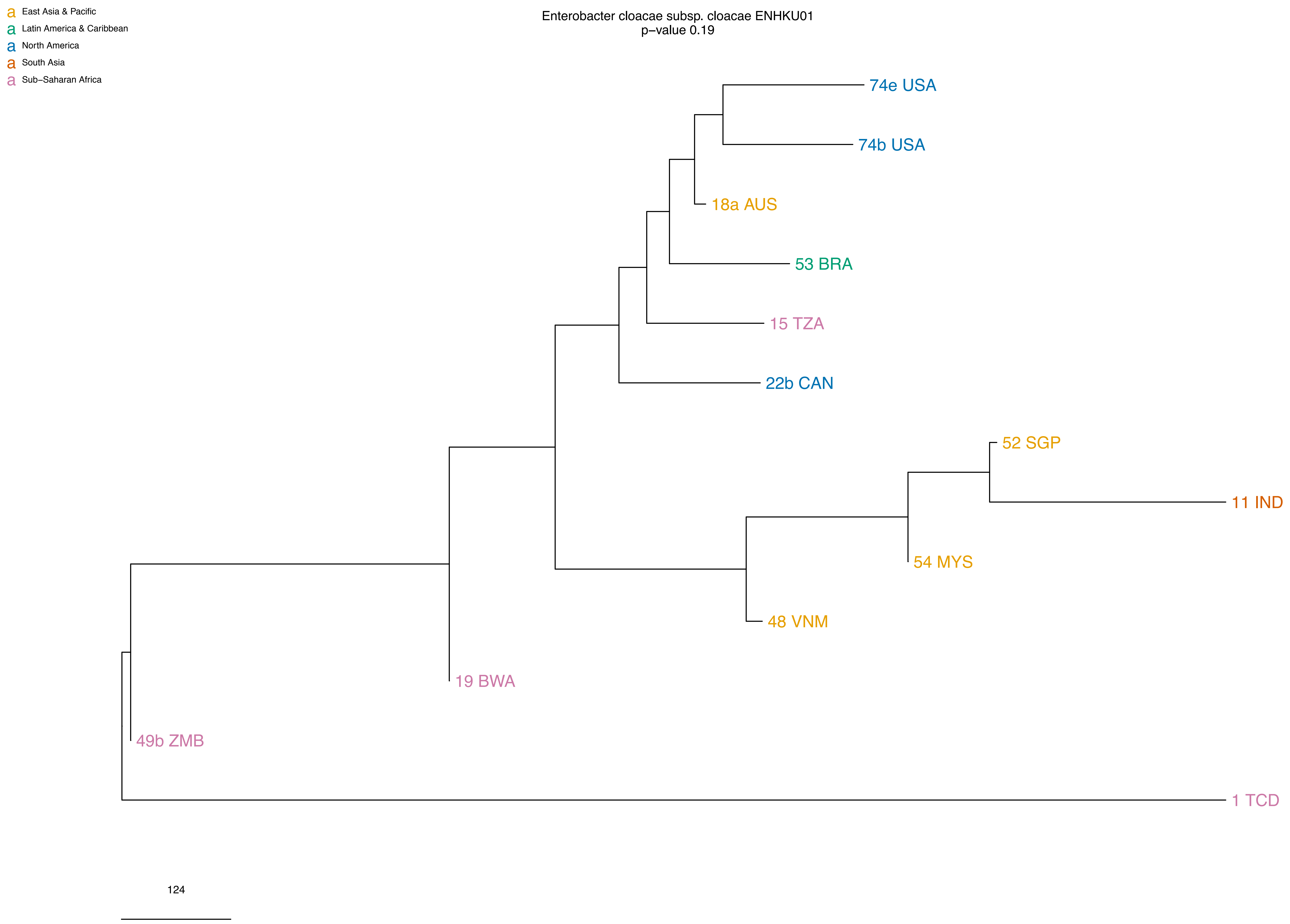

a East Asia & Pacific
a Latin America & Caribbean
a North America
a South Asia
a Sub–Saharan Africa
Enterobacter cloacae subsp. cloacae ENHKU01
p–value 0.19
74e USA
74b USA
18a AUS
53 BRA
15 TZA
22b CAN
52 SGP
11 IND
54 MYS
48 VNM
19 BWA
49b ZMB
1 TCD
124

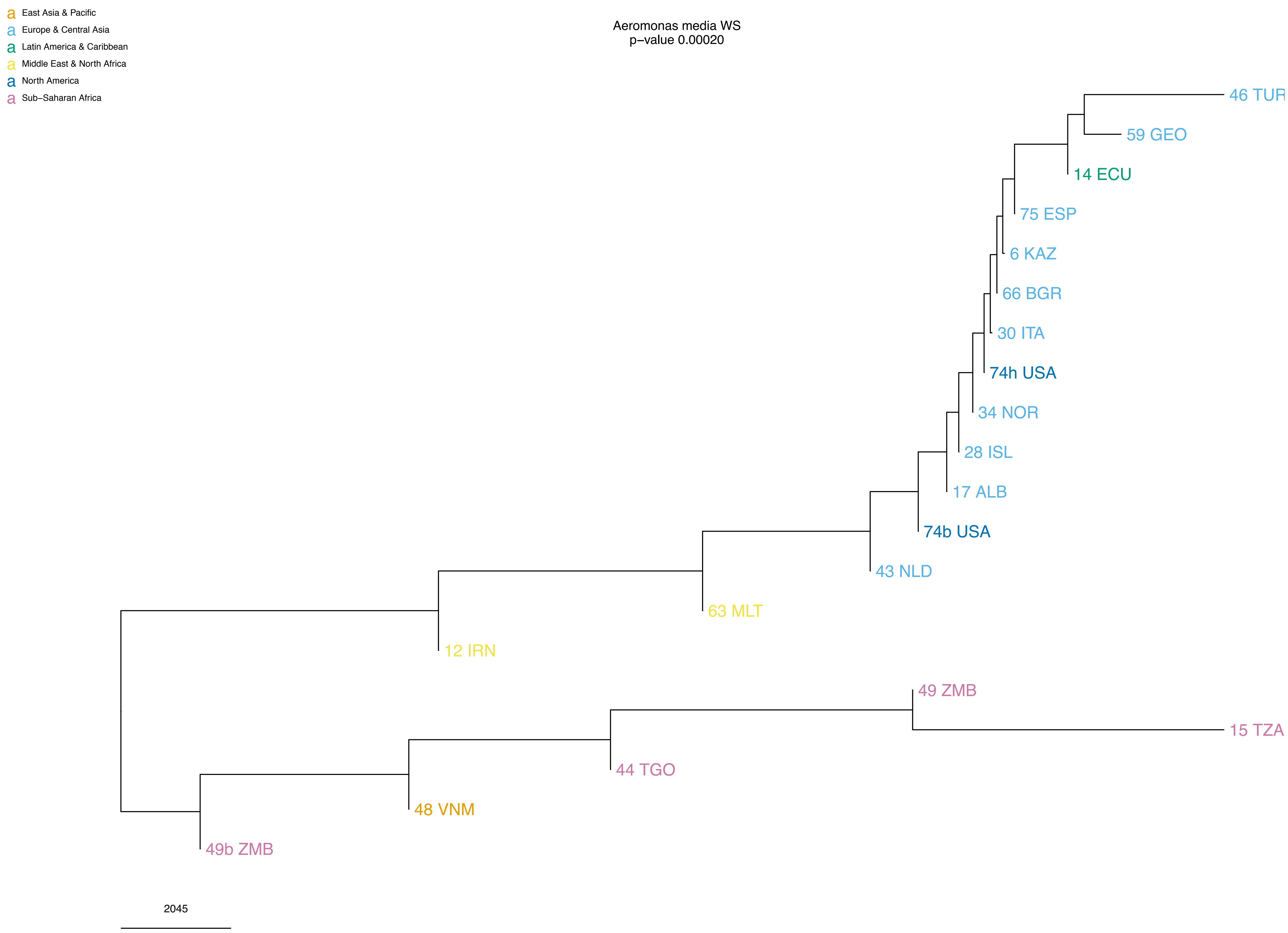
East Asia & Pacific
Europe & Central Asia
Latin America & Caribbean
Middle East & North Africa
North America
Sub–Saharan Africa
Aeromonas media WS
p–value 0.00020
46 TUR
59 GEO
14 ECU
75 ESP
6 KAZ
66 BGR
30 ITA
74h USA
34 NOR
28 ISL
17 ALB
74b USA
43 NLD
63 MLT
12 IRN
49 ZMB
15 TZA
44 TGO
48 VNM
49b ZMB
2045

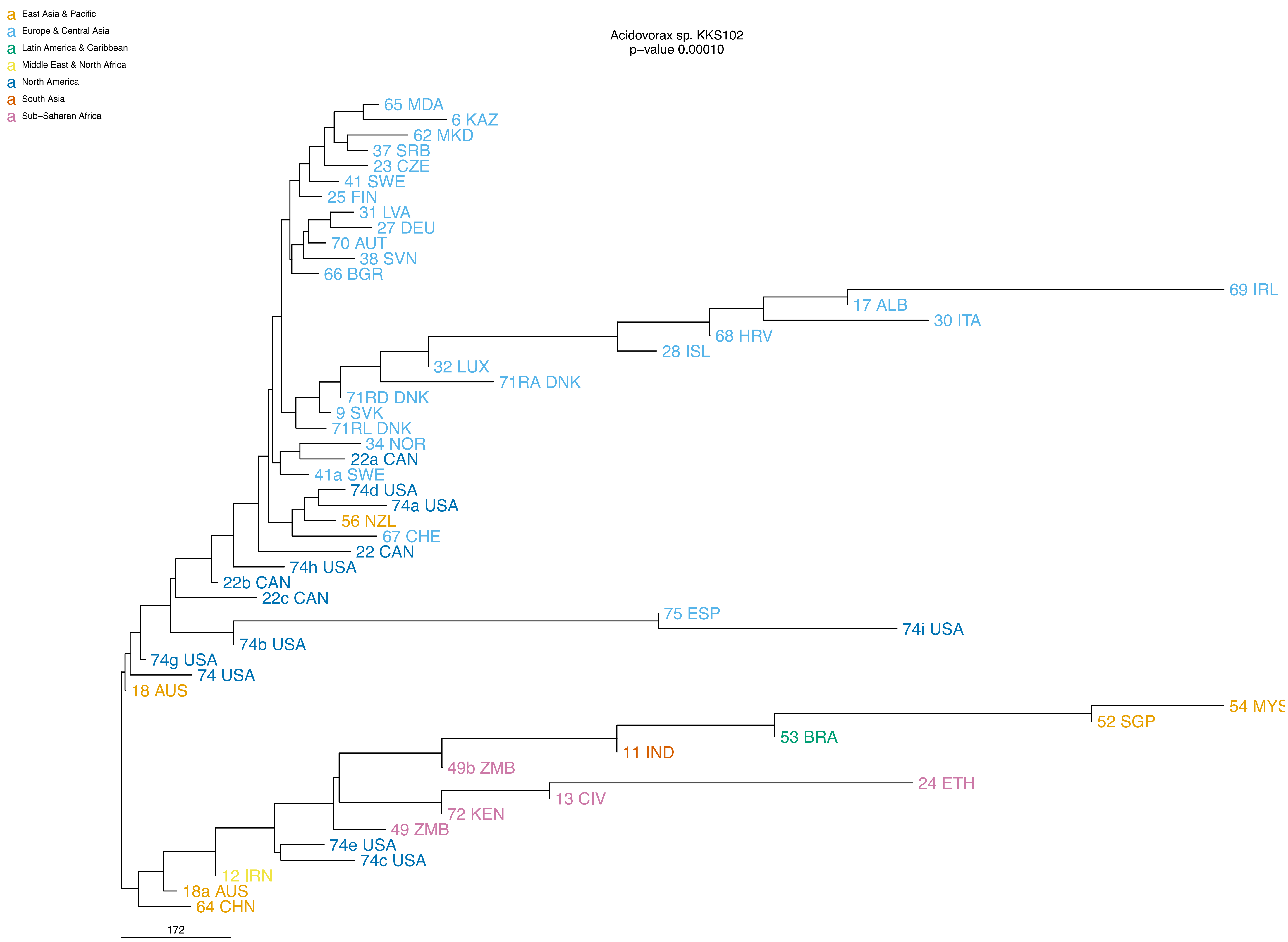

East Asia & Pacific
Europe & Central Asia
Latin America & Caribbean
Middle East & North Africa
North America
South Asia
Sub–Saharan Africa
Acidovorax sp. KKS102
p–value 0.00010
65 MDA
6 KAZ
62 MKD
37 SRB
23 CZE
41 SWE
25 FIN
31 LVA
27 DEU
70 AUT
38 SVN
66 BGR
69 IRL
17 ALB
30 ITA
68 HRV
28 ISL
32 LUX
71RA DNK
71RD DNK
9 SVK
71RL DNK
34 NOR
22a CAN
41a SWE
74d USA
74a USA
56 NZL
67 CHE
22 CAN
74h USA
22b CAN
22c CAN
75 ESP
74i USA
74b USA
74g USA
74 USA
18 AUS
54 MYS
52 SGP
53 BRA
11 IND
49b ZMB
24 ETH
13 CIV
72 KEN
49 ZMB
74e USA
74c USA
12 IRN
18a AUS
64 CHN
172

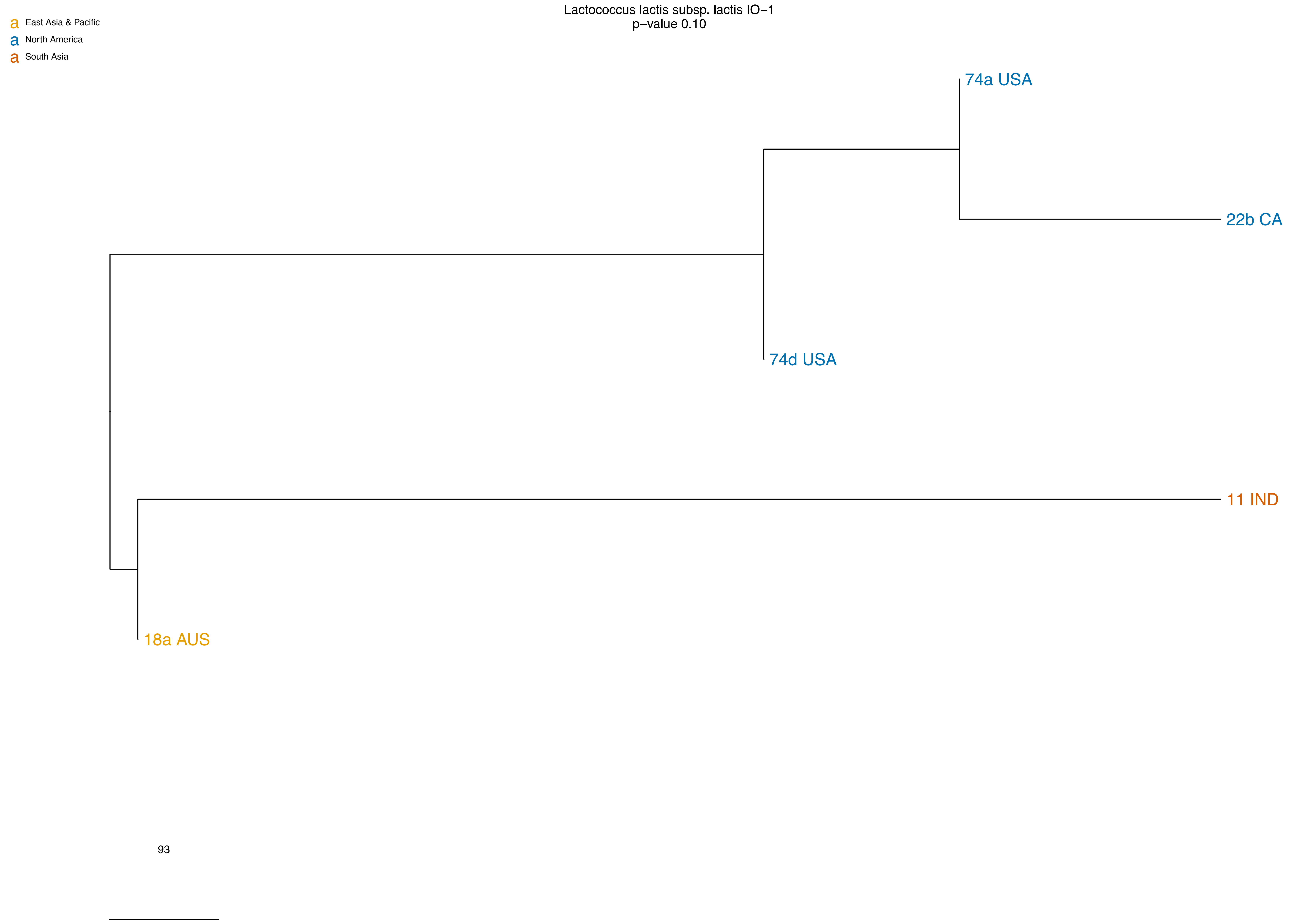

a East Asia & Pacific
a North America
a South Asia
Lactococcus lactis subsp. lactis IO−1
p−value 0.10
74a USA
22b CA
74d USA
11 IND
18a AUS
93

Raoultella ornithinolytica B6
p–value 1.0

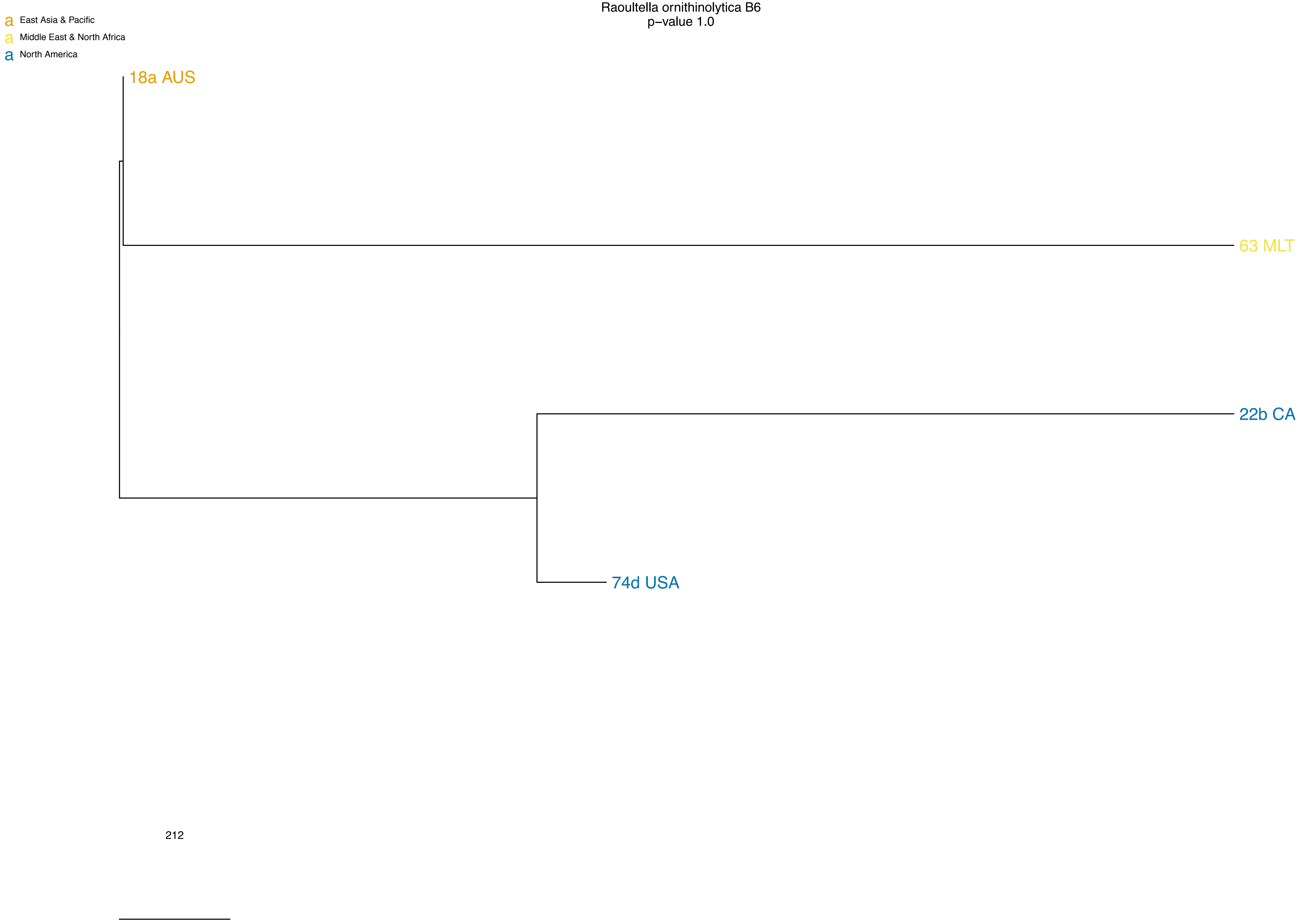

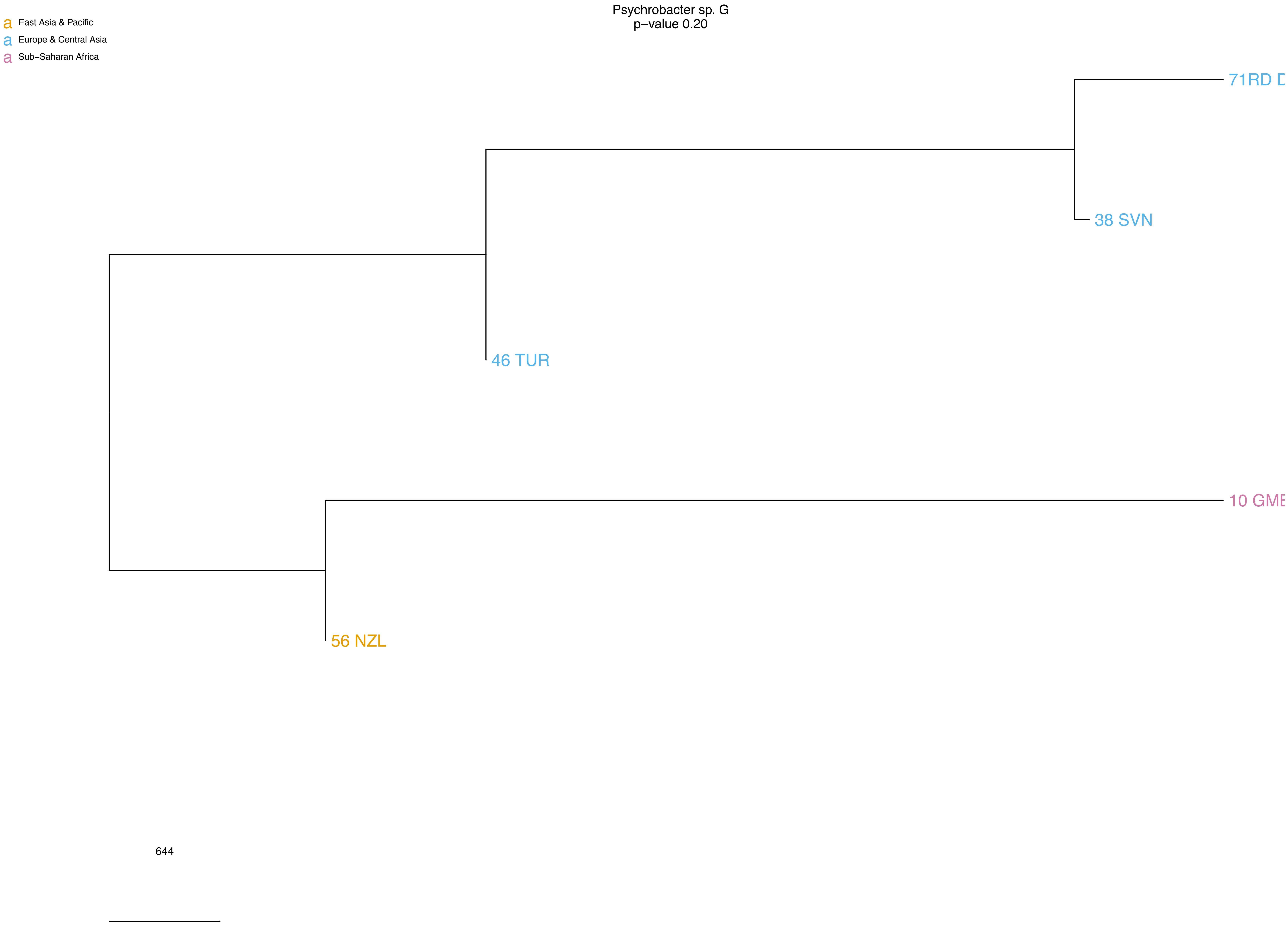

East Asia & Pacific
Europe & Central Asia
Sub−Saharan Africa
Psychrobacter sp. G
p−value 0.20
71RD D
38 SVN
46 TUR
10 GMB
56 NZL
644

Streptococcus lutetiensis 033
p–value 1.0

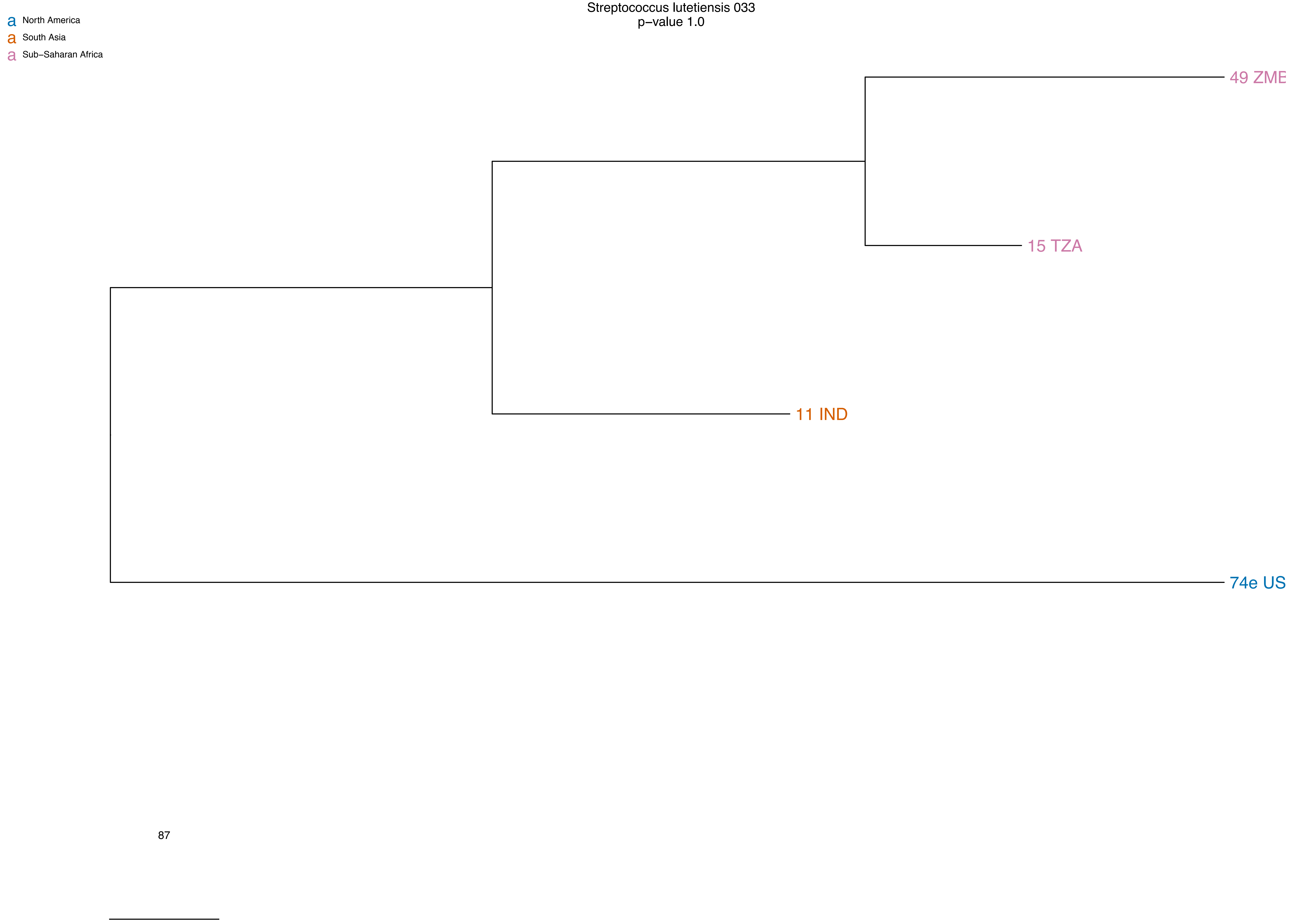

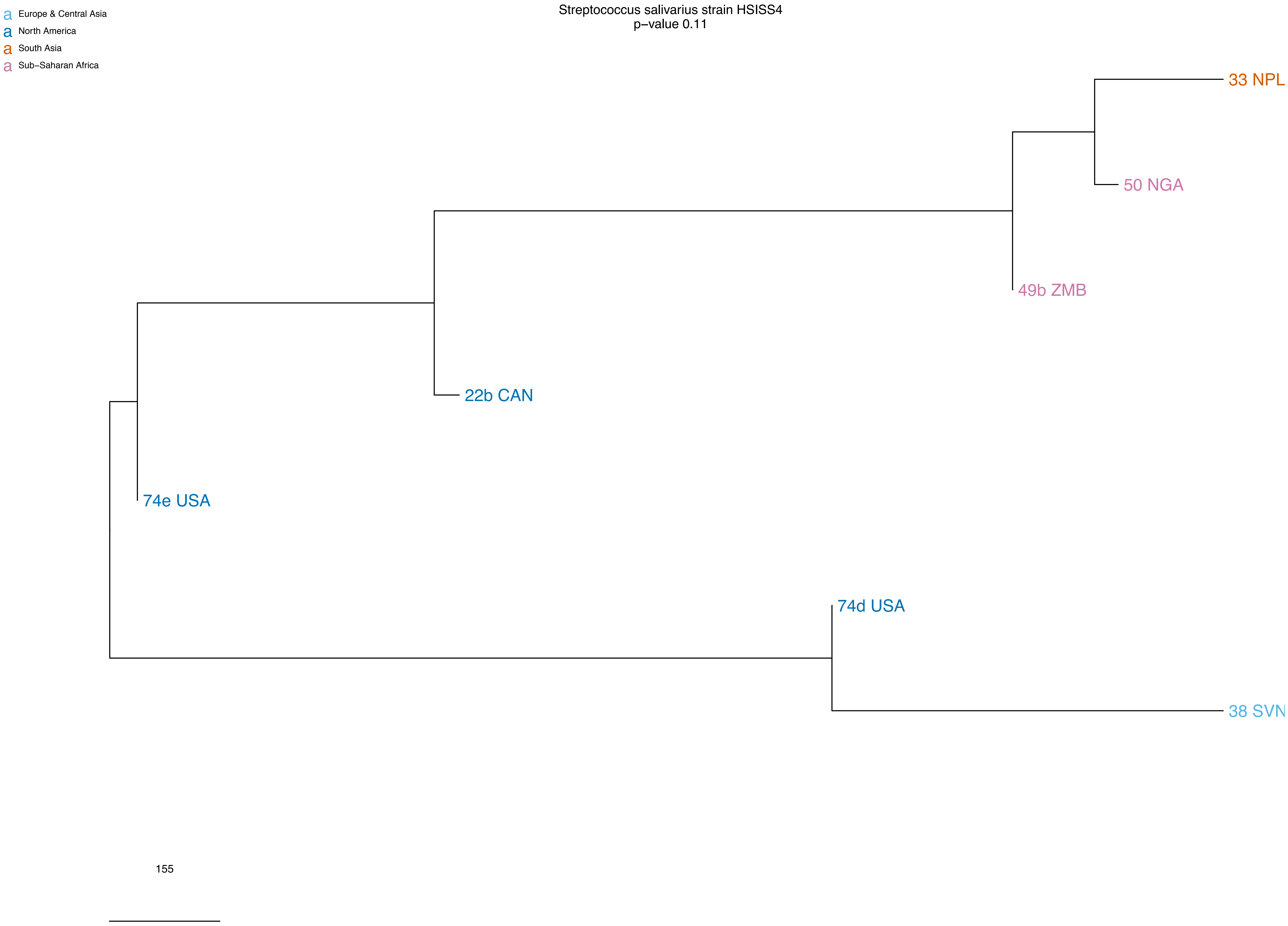
a Europe & Central Asia
a North America
a South Asia
a Sub−Saharan Africa
Streptococcus salivarius strain HSISS4
p−value 0.11
33 NPL
50 NGA
49b ZMB
22b CAN
74e USA
74d USA
38 SVN
155

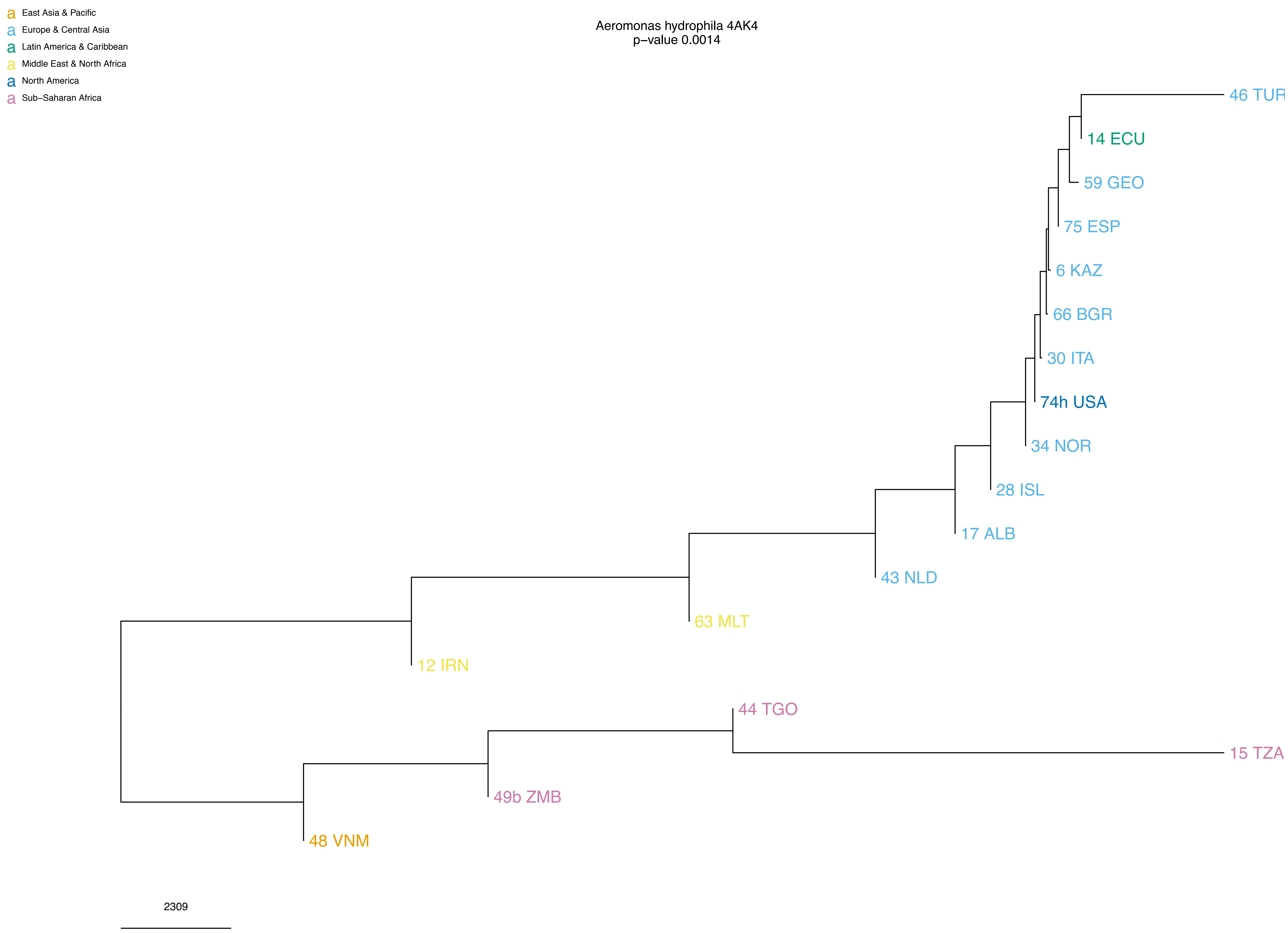
East Asia & Pacific
Europe & Central Asia
Latin America & Caribbean
Middle East & North Africa
North America
Sub–Saharan Africa
Aeromonas hydrophila 4AK4
p–value 0.0014
46 TUR
14 ECU
59 GEO
75 ESP
6 KAZ
66 BGR
30 ITA
74h USA
34 NOR
28 ISL
17 ALB
43 NLD
63 MLT
12 IRN
44 TGO
15 TZA
49b ZMB
48 VNM
2309

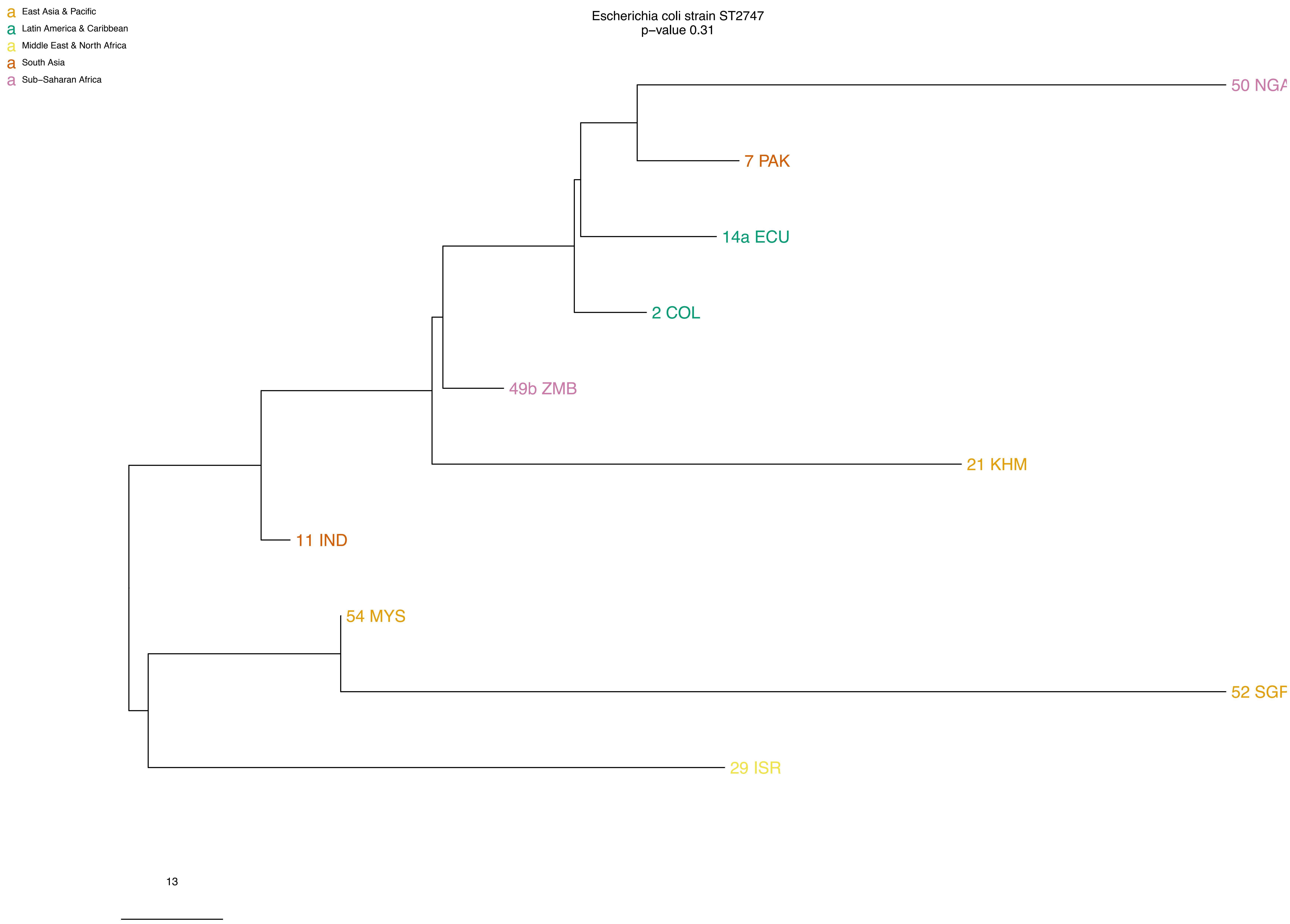
a East Asia & Pacific
a Latin America & Caribbean
a Middle East & North Africa
a South Asia
a Sub–Saharan Africa
Escherichia coli strain ST2747
p–value 0.31
7 PAK
14a ECU
2 COL
49b ZMB
21 KHM
11 IND
54 MYS
29 ISR
13

Aeromonas hydrophila YL17
p–value 1.0

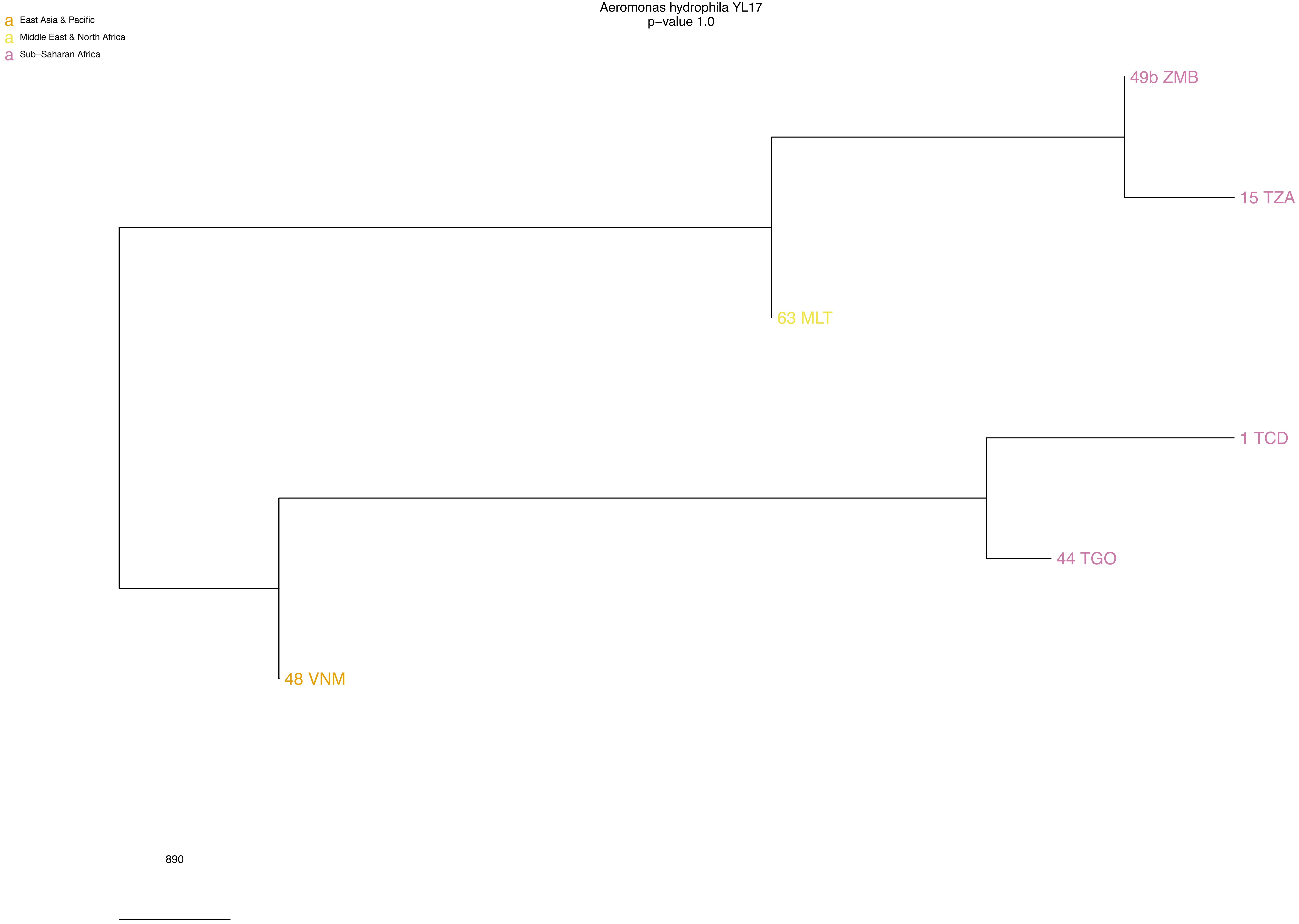

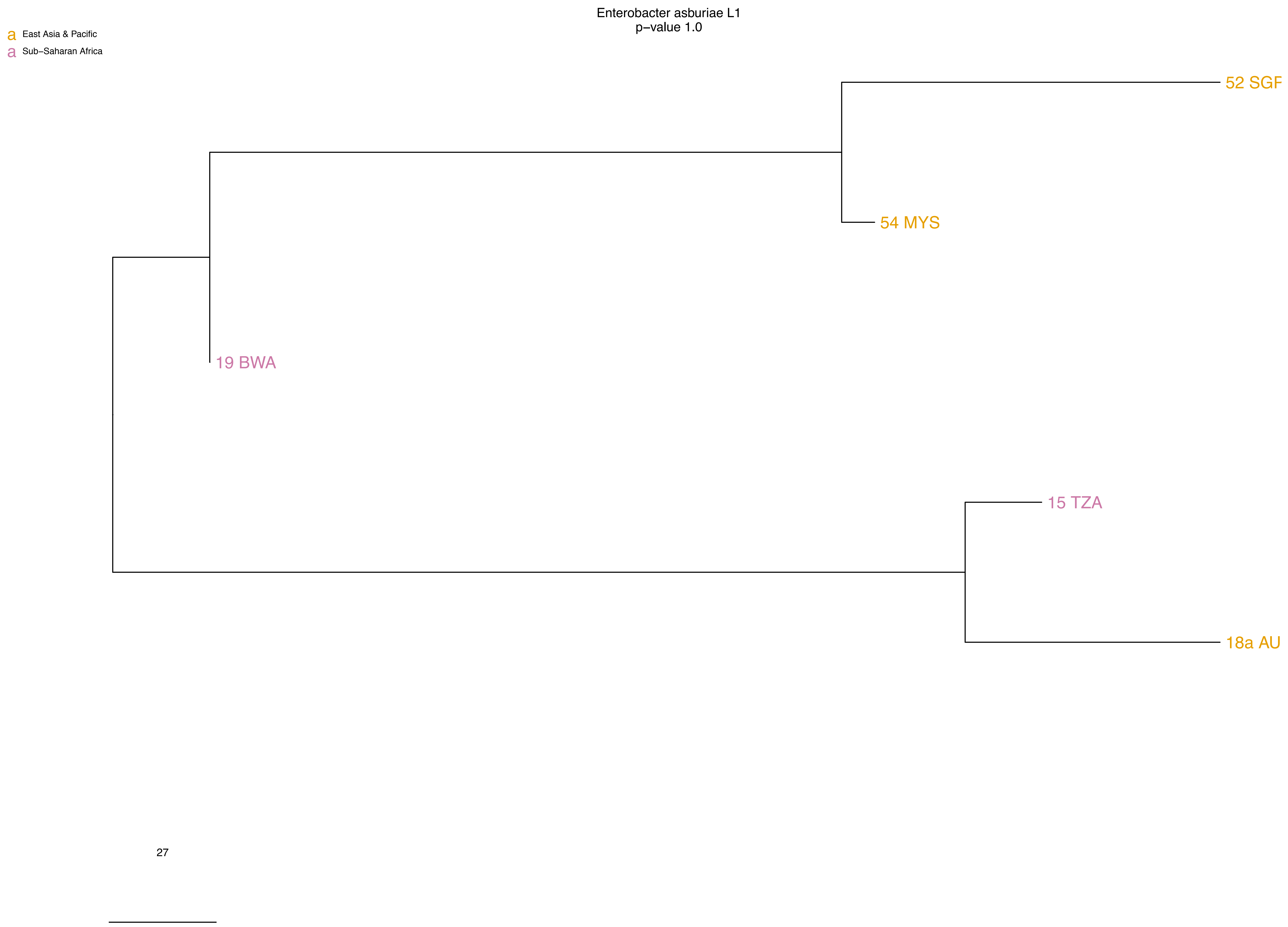

Enterobacter asburiae L1
p–value 1.0
East Asia & Pacific
Sub–Saharan Africa
52 SGF
54 MYS
19 BWA
15 TZA
18a AU
27

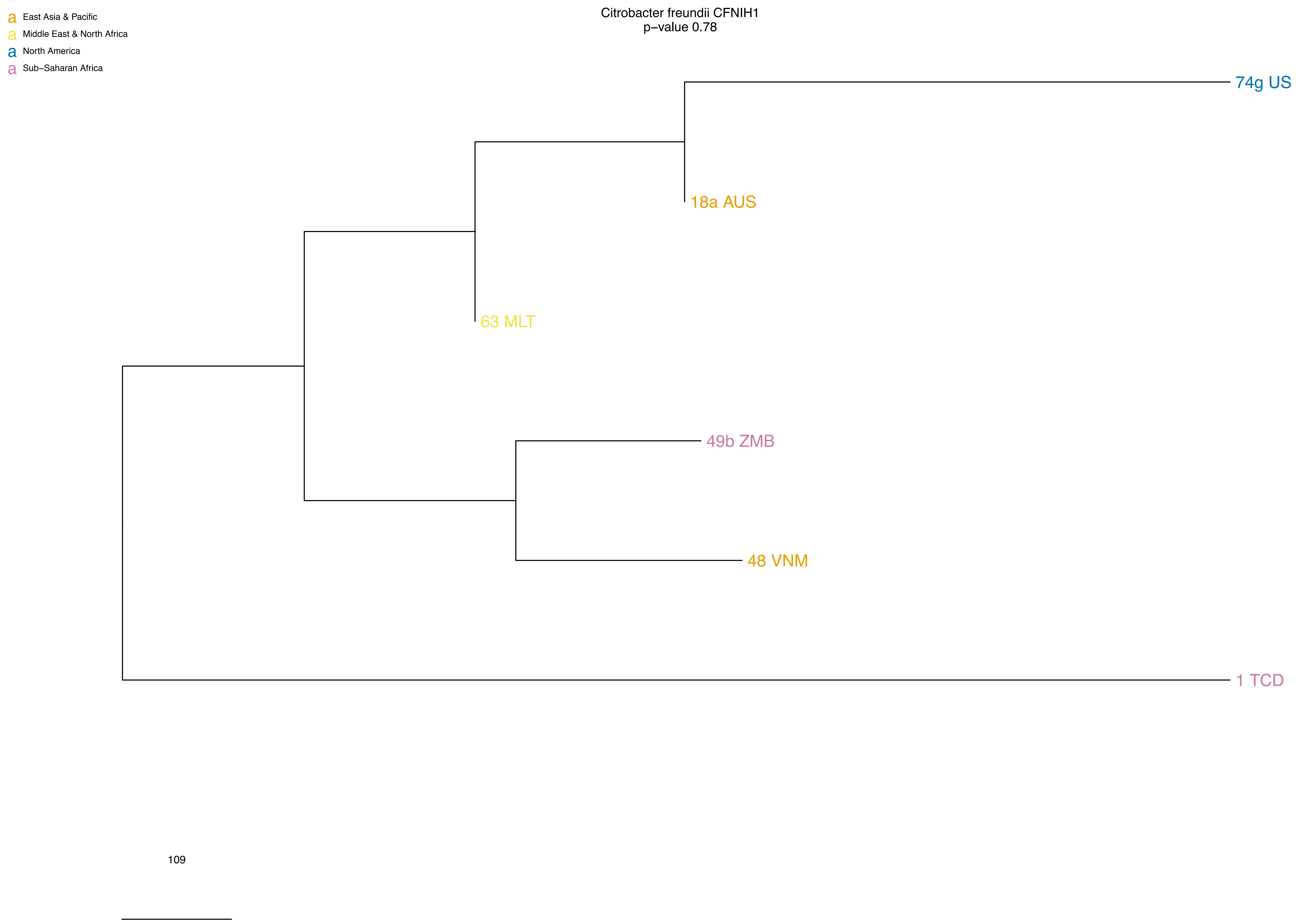

a East Asia & Pacific
a Middle East & North Africa
a North America
a Sub–Saharan Africa
Citrobacter freundii CFNIH1
p–value 0.78
74g US
18a AUS
63 MLT
49b ZMB
48 VNM
1 TCD
109

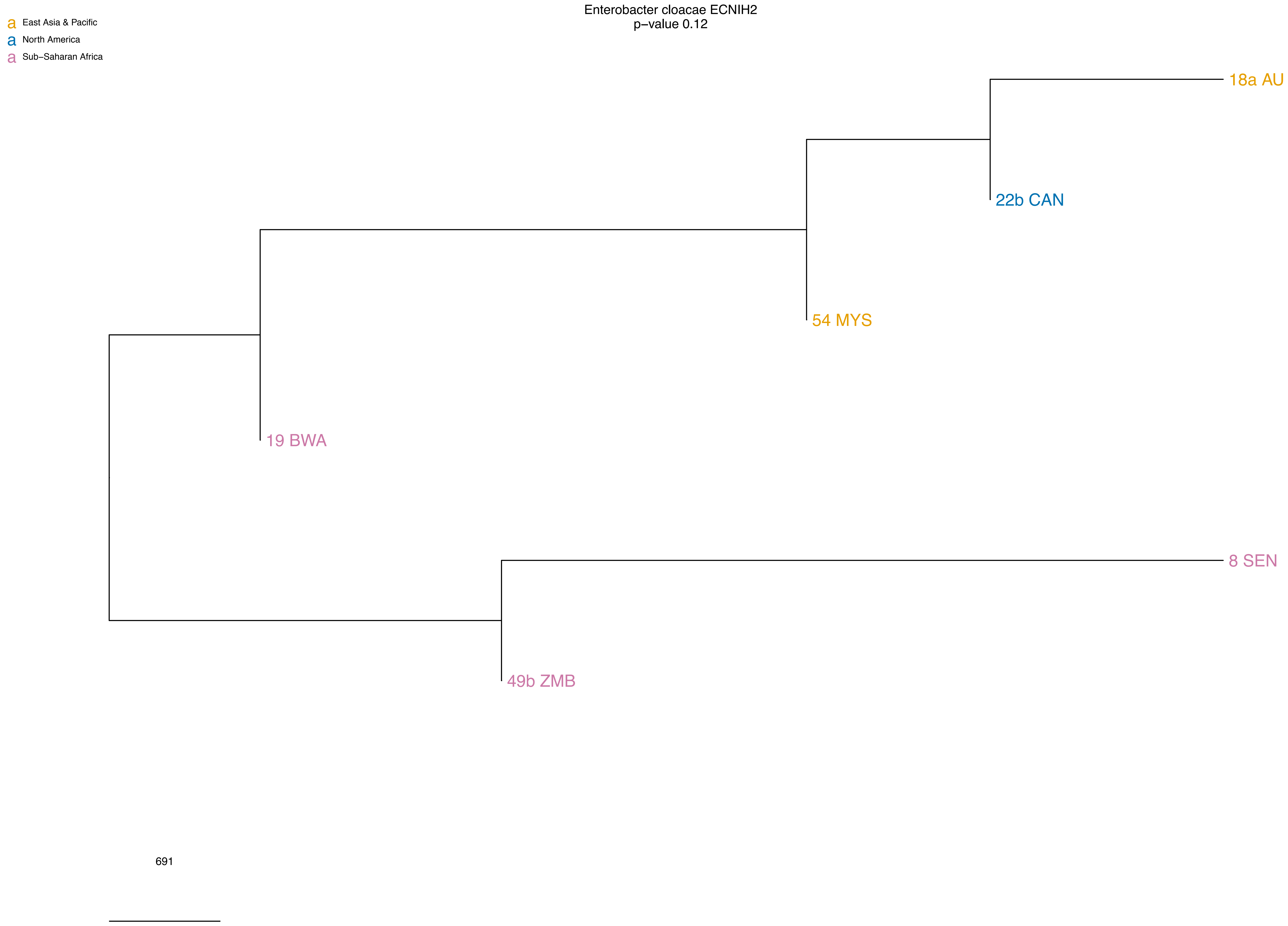

Enterobacter cloacae ECNIH2
p–value 0.12
a East Asia & Pacific
a North America
a Sub–Saharan Africa
18a AU
22b CAN
54 MYS
19 BWA
8 SEN
49b ZMB
691

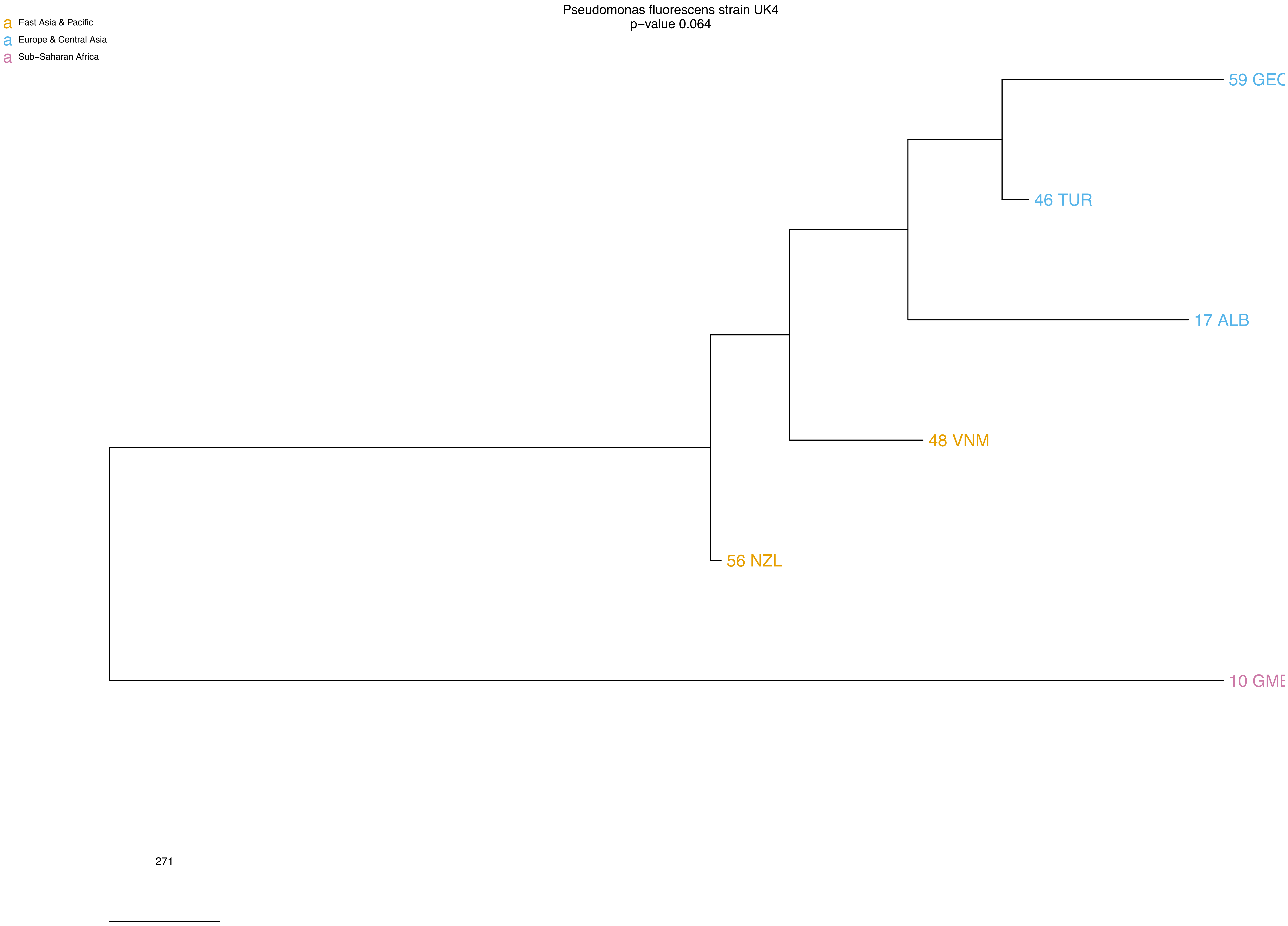

Pseudomonas fluorescens strain UK4
p–value 0.064
East Asia & Pacific
Europe & Central Asia
Sub–Saharan Africa
59 GEO
46 TUR
17 ALB
48 VNM
56 NZL
10 GMB
271

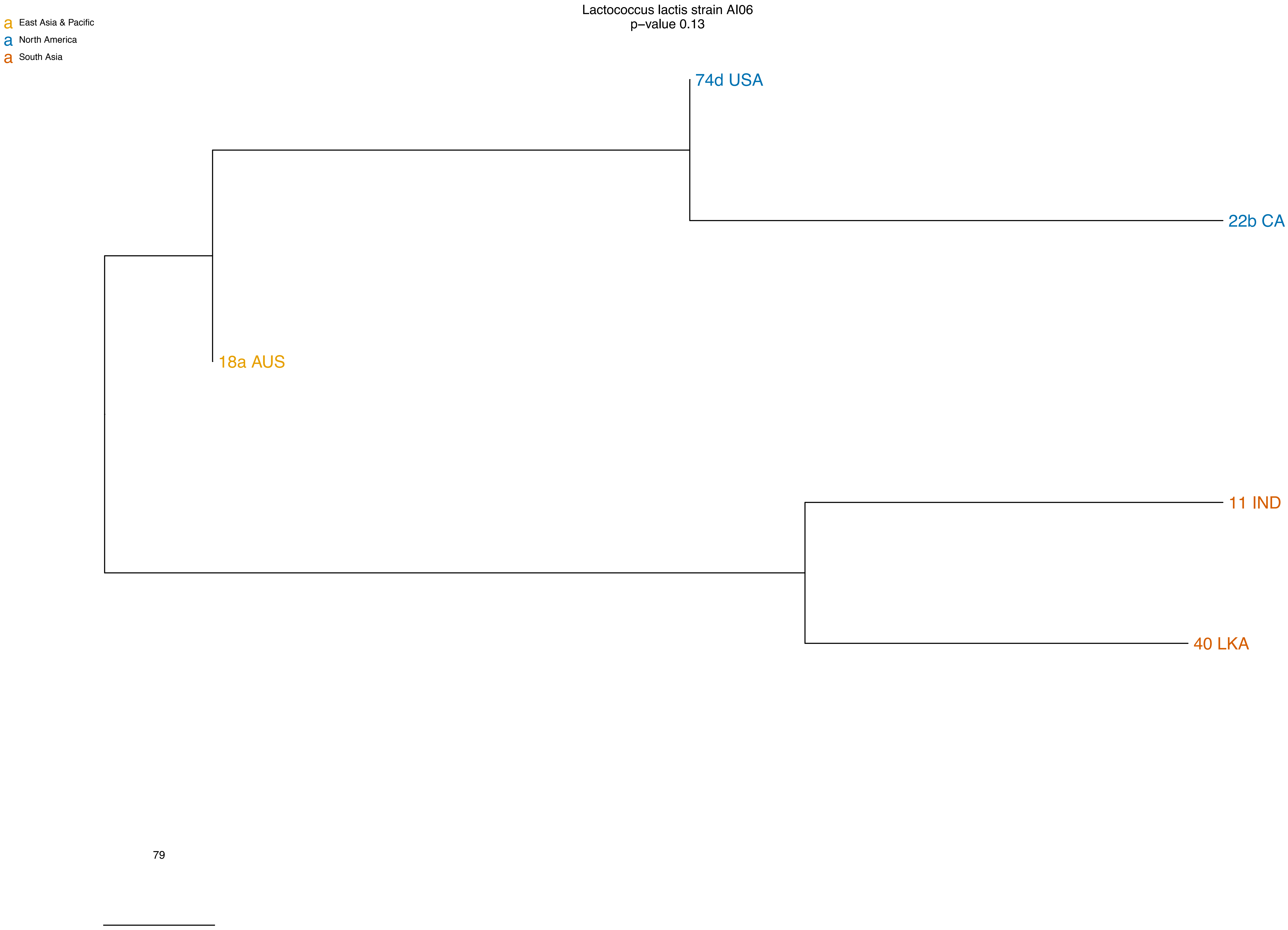

East Asia & Pacific
North America
South Asia
Lactococcus lactis strain AI06
p–value 0.13
74d USA
22b CA
18a AUS
11 IND
40 LKA
79

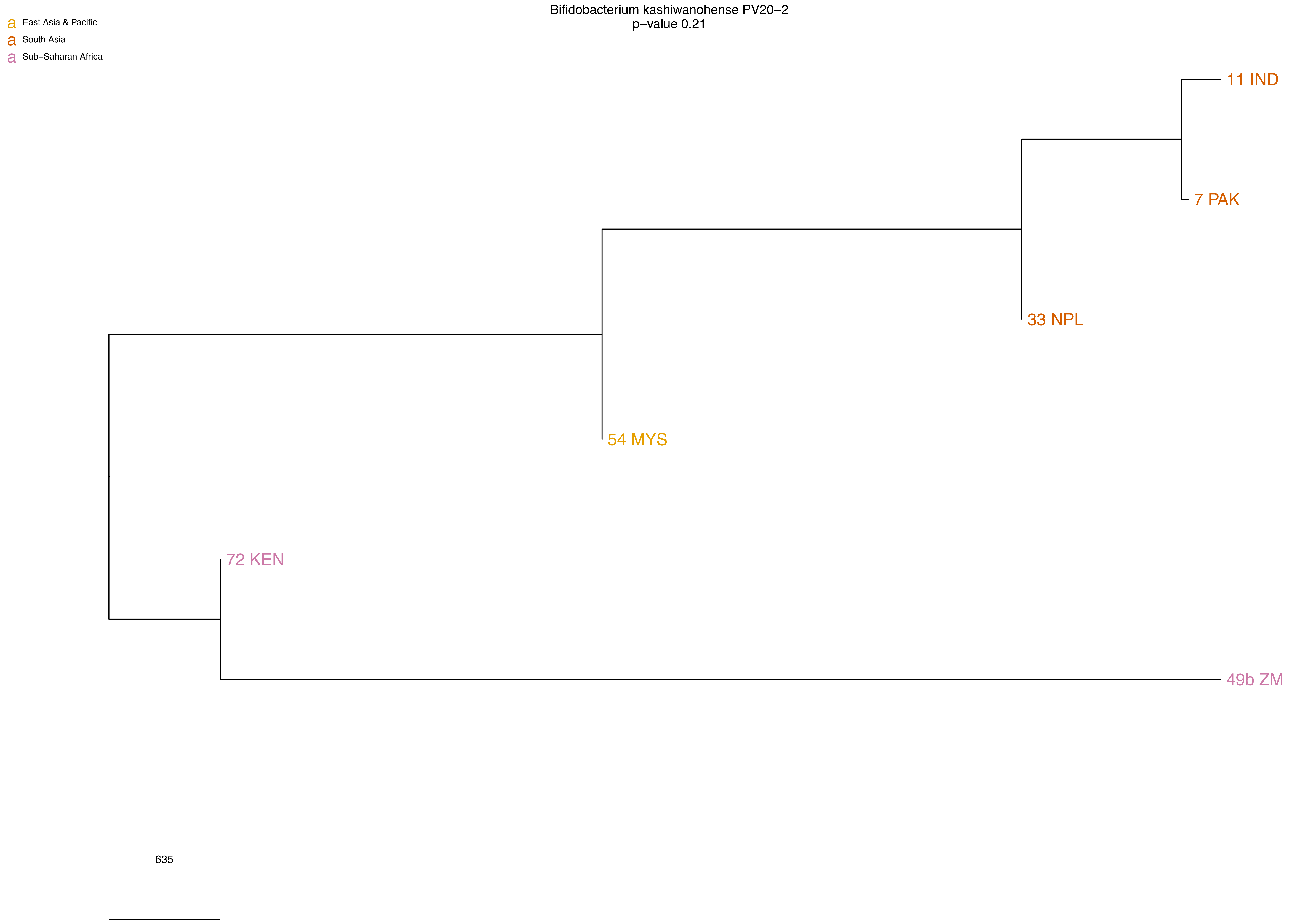

Bifidobacterium kashiwanohense PV20−2
p−value 0.21
a East Asia & Pacific
a South Asia
a Sub−Saharan Africa
11 IND
7 PAK
33 NPL
54 MYS
72 KEN
49b ZM
635

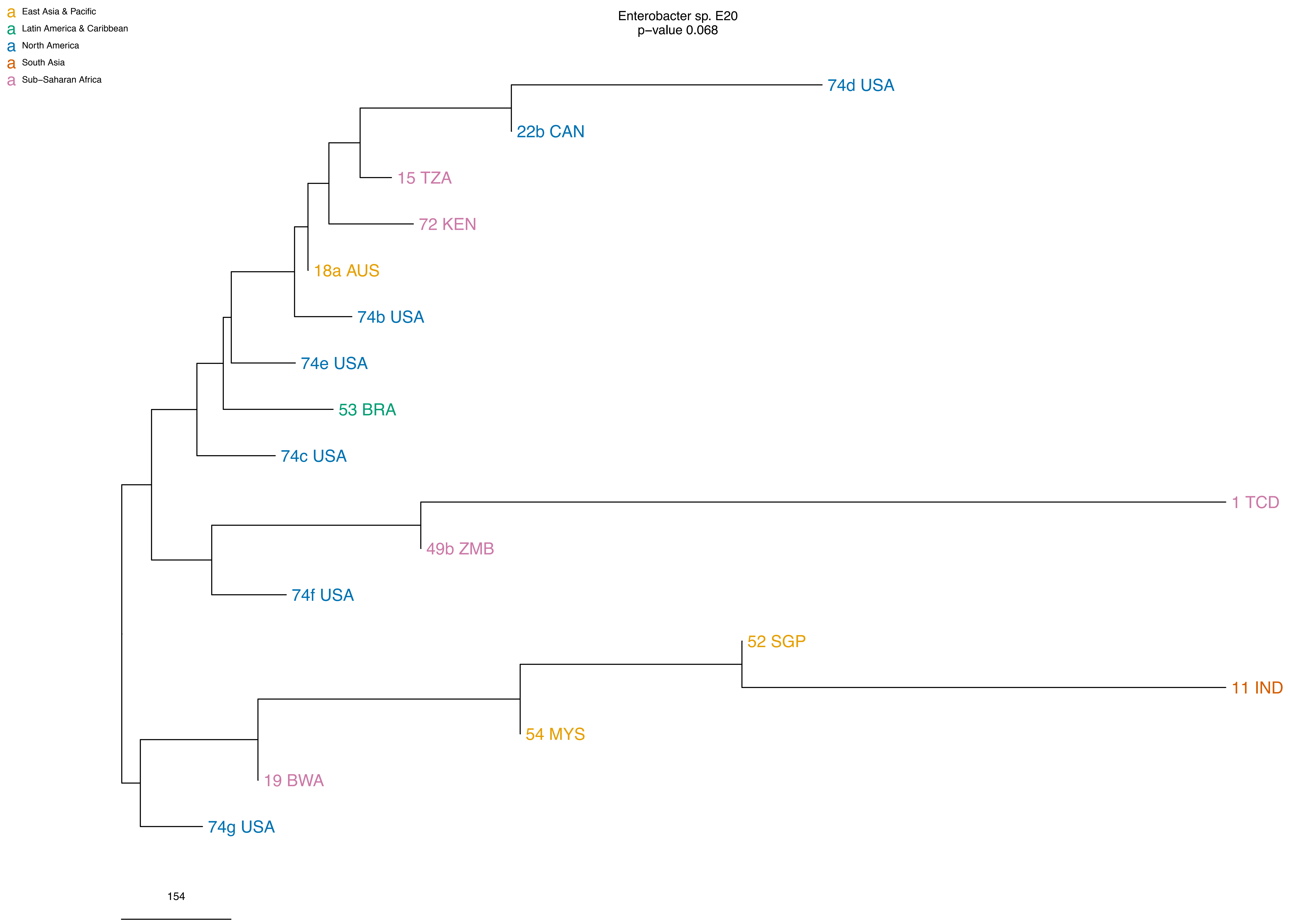

a East Asia & Pacific
a Latin America & Caribbean
a North America
a South Asia
a Sub–Saharan Africa
Enterobacter sp. E20
p–value 0.068
74d USA
22b CAN
15 TZA
72 KEN
18a AUS
74b USA
74e USA
53 BRA
74c USA
1 TCD
49b ZMB
74f USA
52 SGP
11 IND
54 MYS
19 BWA
74g USA
154

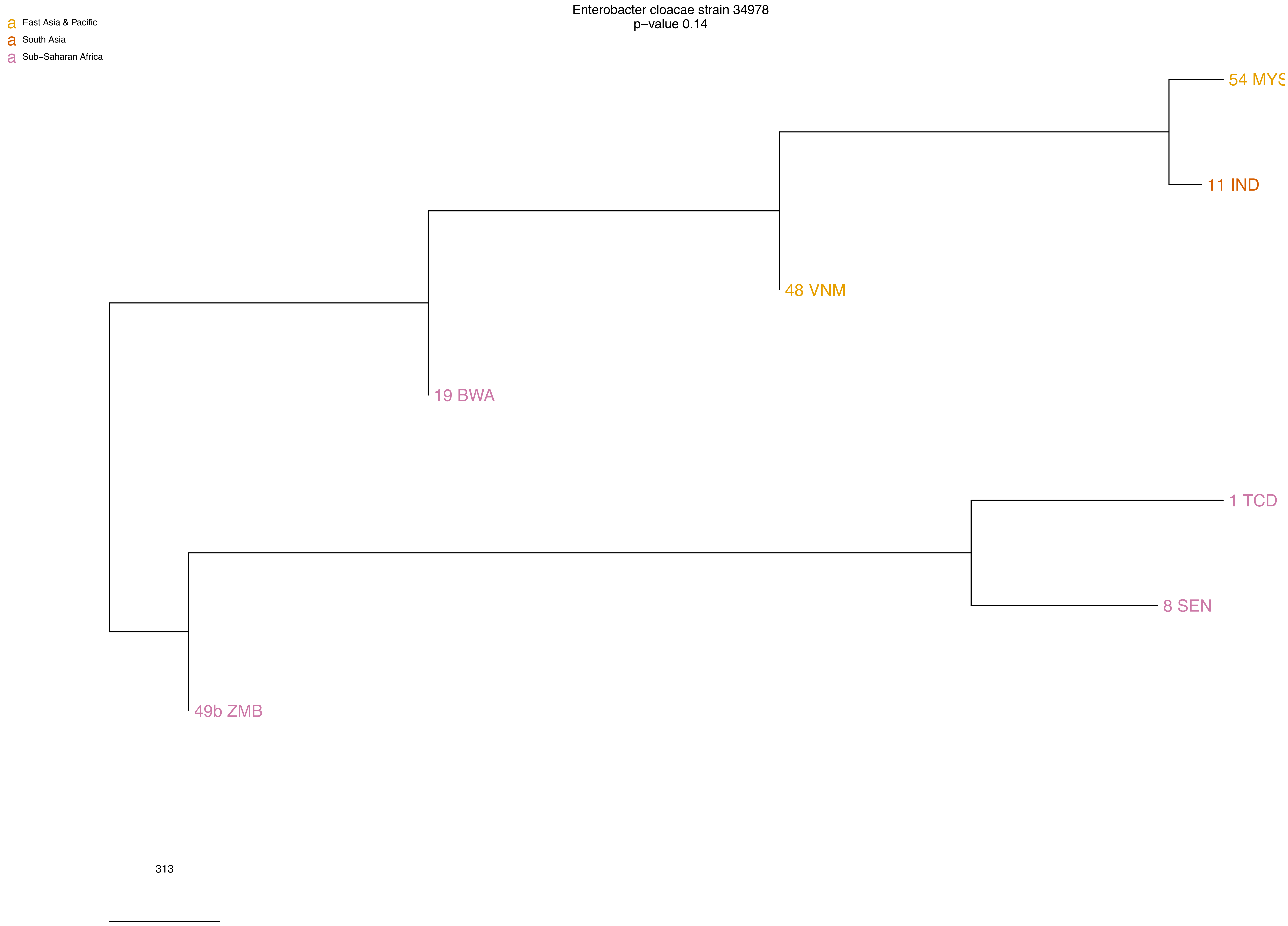
Enterobacter cloacae strain 34978
p–value 0.14
East Asia & Pacific
South Asia
Sub–Saharan Africa
54 MYS
11 IND
48 VNM
19 BWA
1 TCD
8 SEN
49b ZMB
313

Enterobacter asburiae strain 35734
p–value 0.38

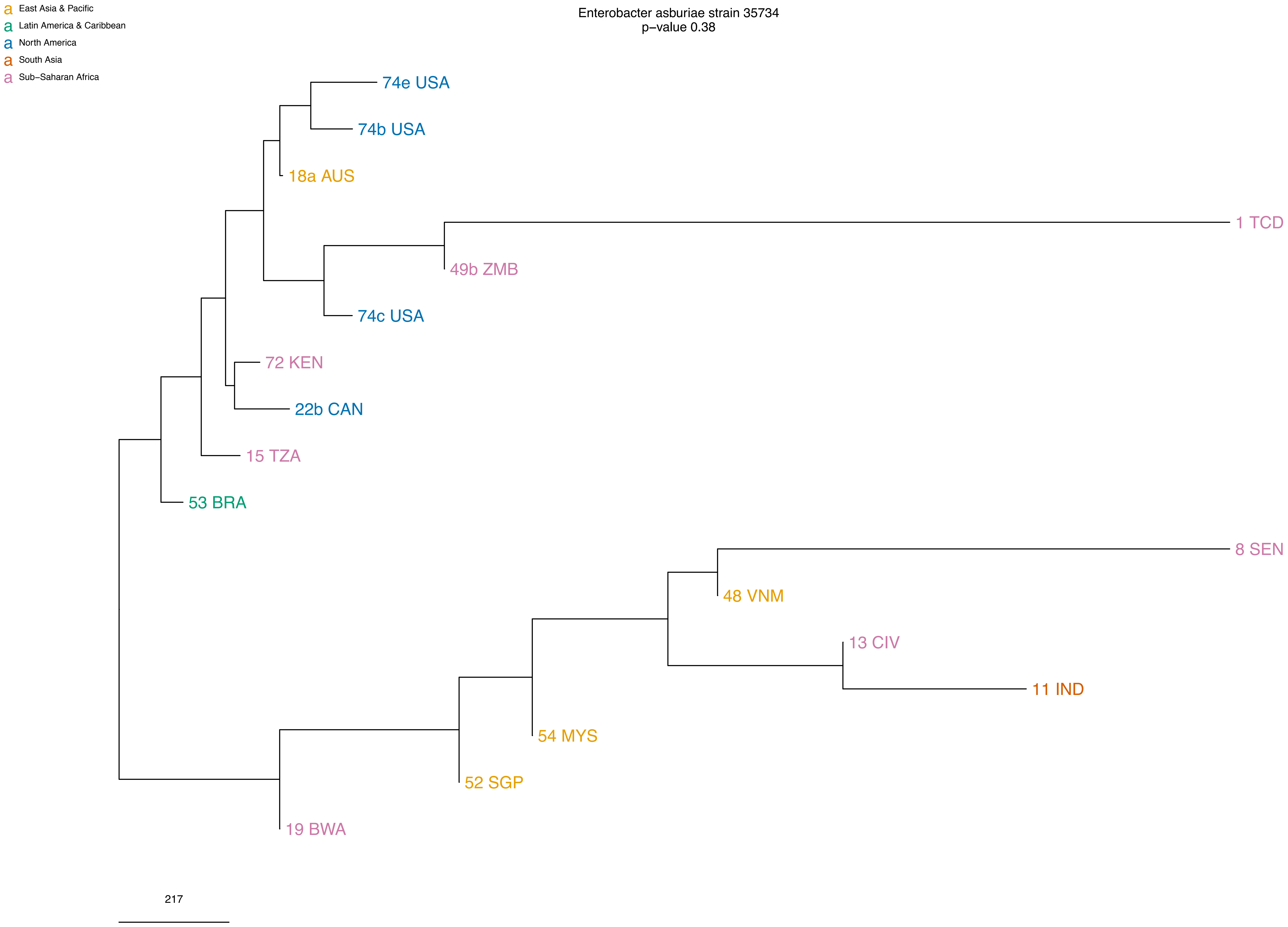

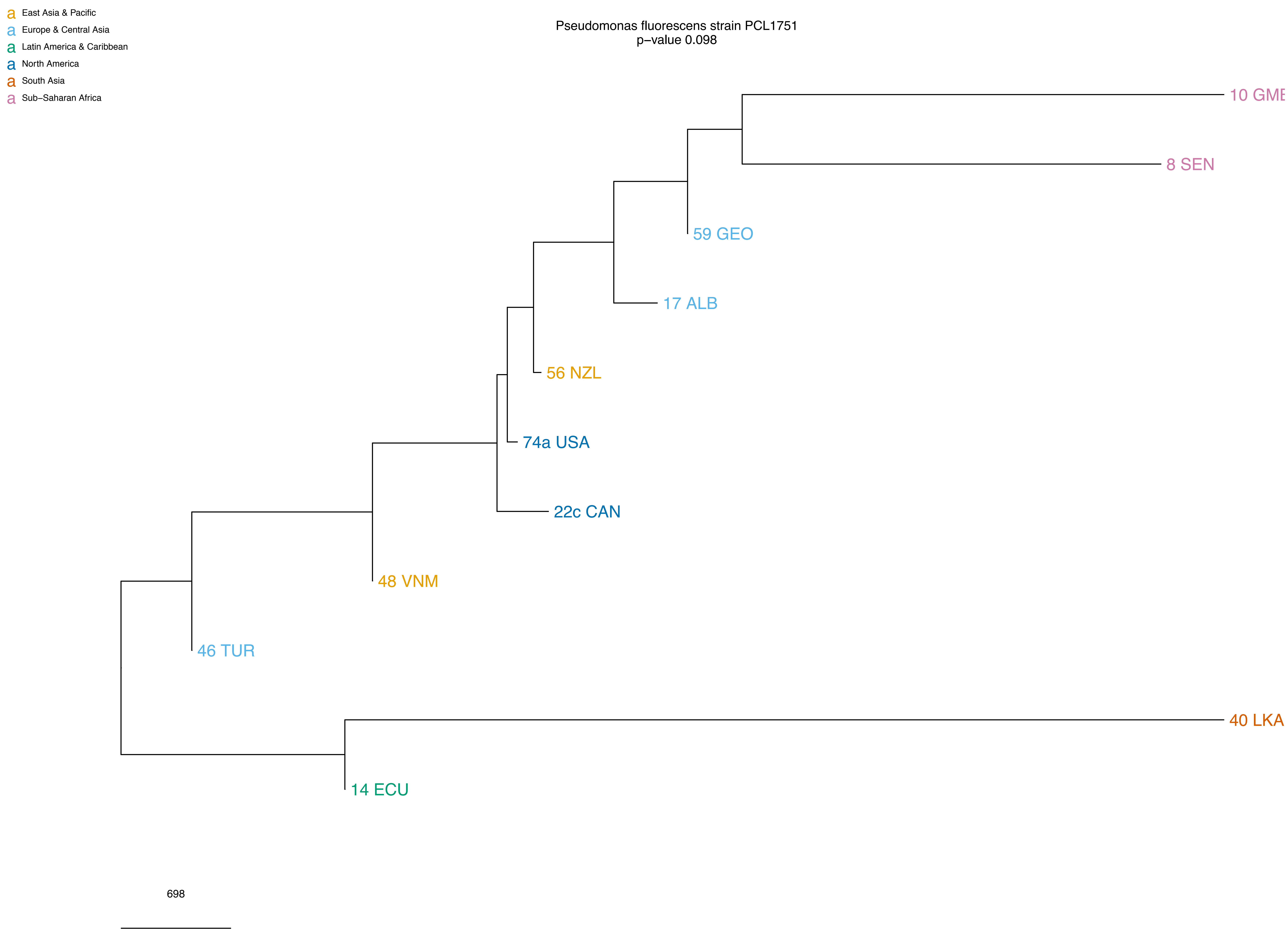

a East Asia & Pacific
a Europe & Central Asia
a Latin America & Caribbean
a North America
a South Asia
a Sub–Saharan Africa
Pseudomonas fluorescens strain PCL1751
p–value 0.098
10 GMB
8 SEN
59 GEO
17 ALB
56 NZL
74a USA
22c CAN
48 VNM
46 TUR
40 LKA
14 ECU
698

Aeromonas hydrophila strain AL06−06
p−value 0

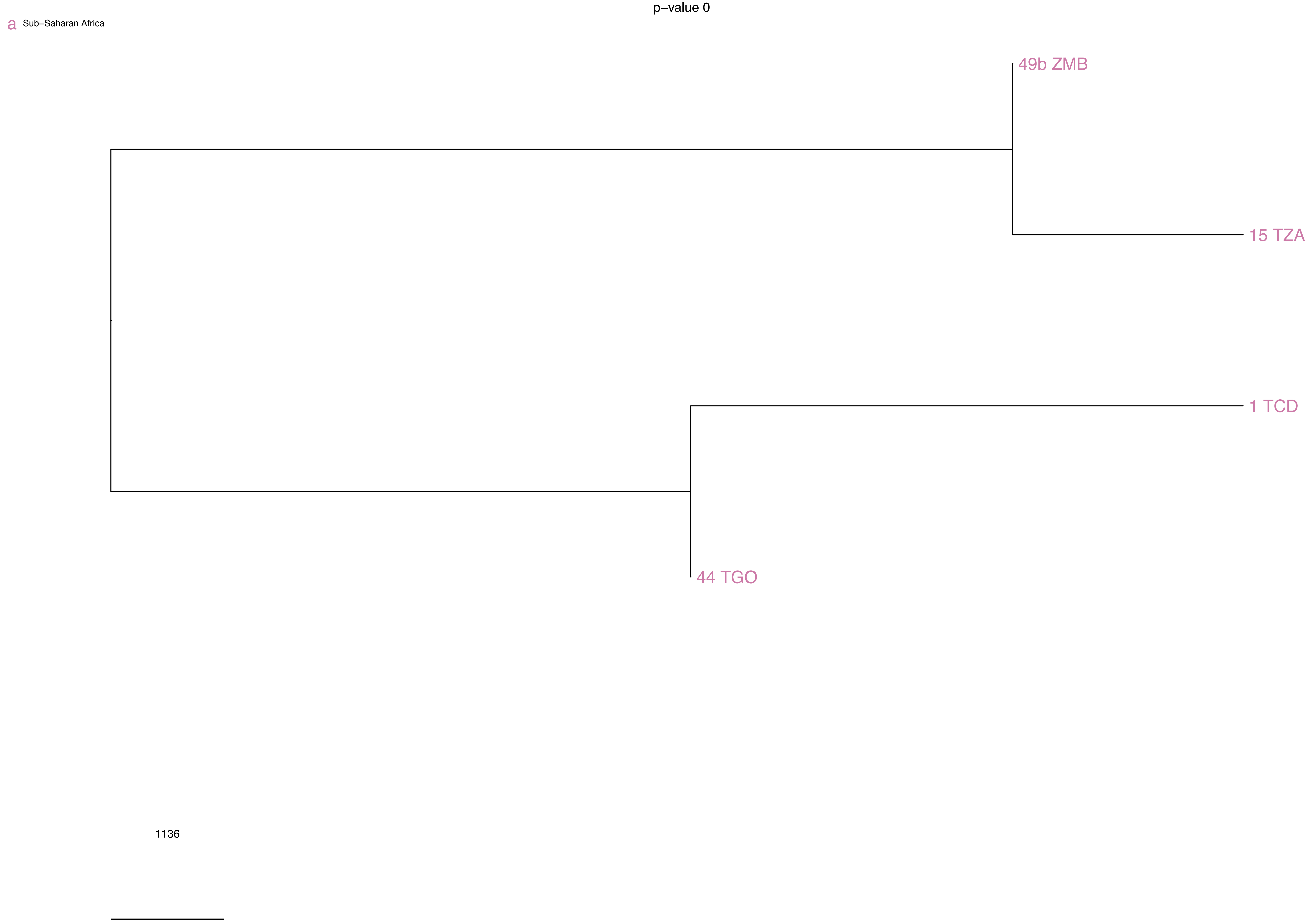

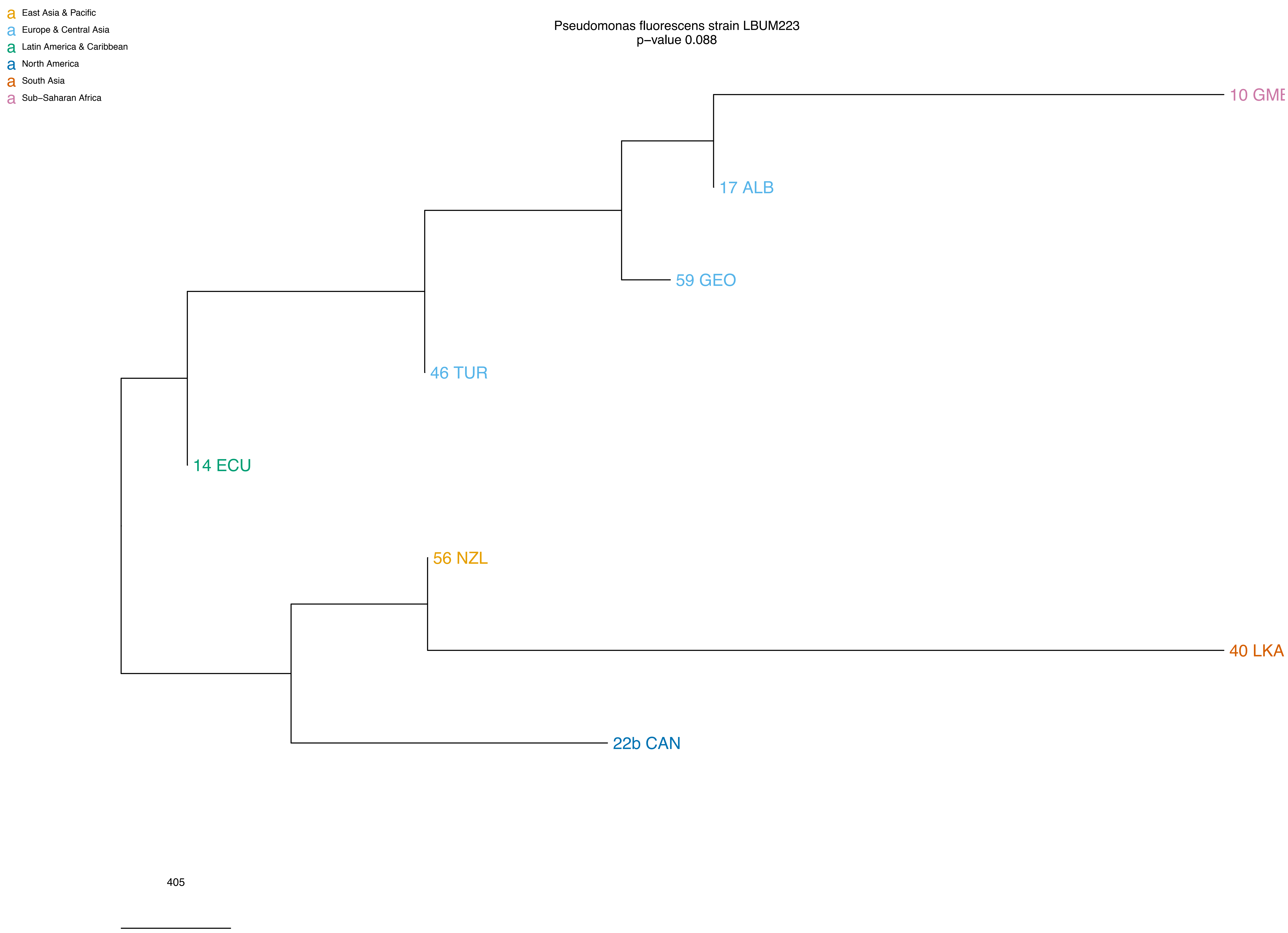
East Asia & Pacific
Europe & Central Asia
Latin America & Caribbean
North America
South Asia
Sub–Saharan Africa
Pseudomonas fluorescens strain LBUM223
p–value 0.088
10 GMB
17 ALB
59 GEO
46 TUR
14 ECU
56 NZL
40 LKA
22b CAN
405

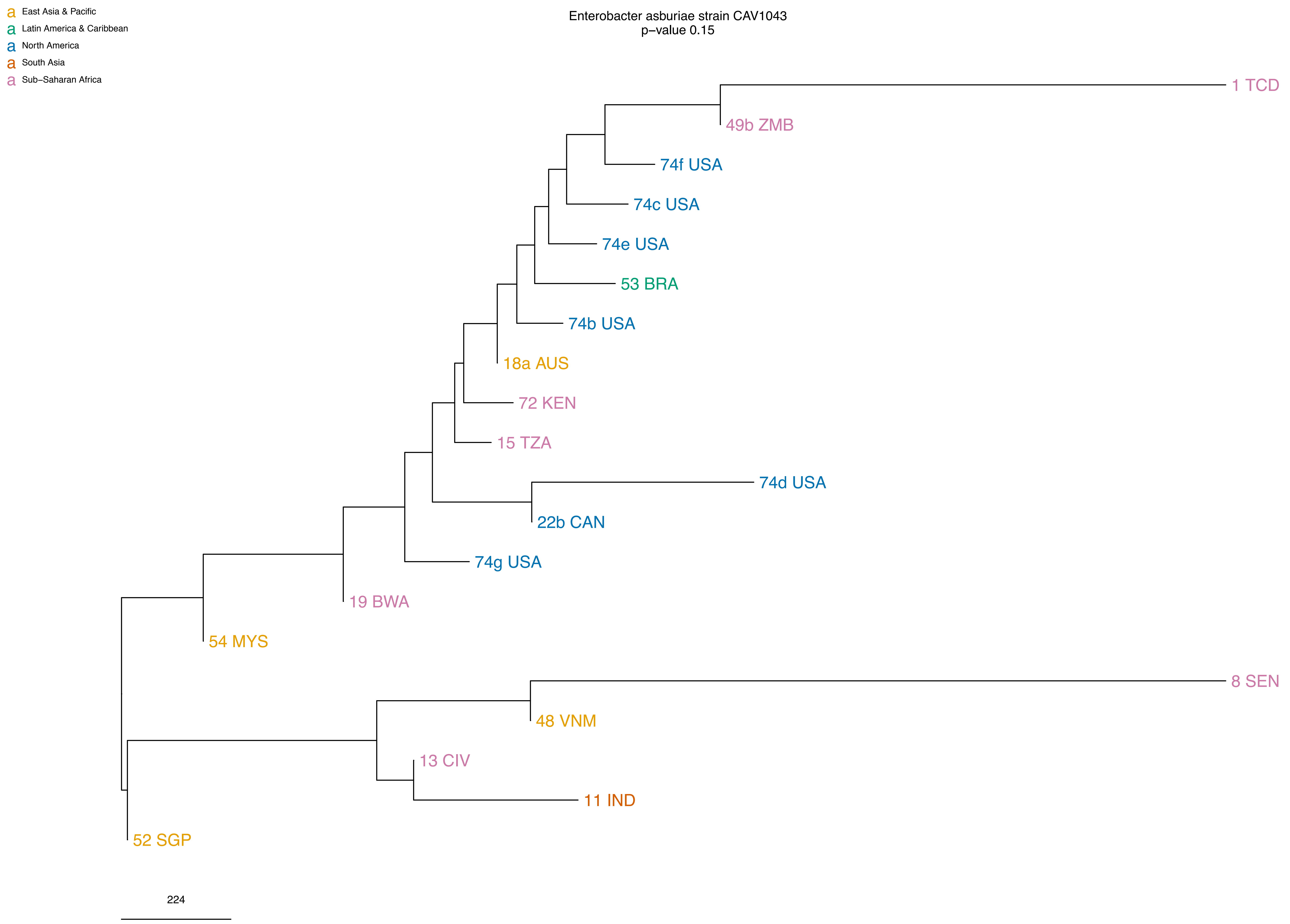
a East Asia & Pacific
a Latin America & Caribbean
a North America
a South Asia
a Sub–Saharan Africa
Enterobacter asburiae strain CAV1043
p–value 0.15
1 TCD
49b ZMB
74f USA
74c USA
74e USA
53 BRA
74b USA
18a AUS
72 KEN
15 TZA
74d USA
22b CAN
74g USA
19 BWA
54 MYS
8 SEN
48 VNM
13 CIV
11 IND
52 SGP
224

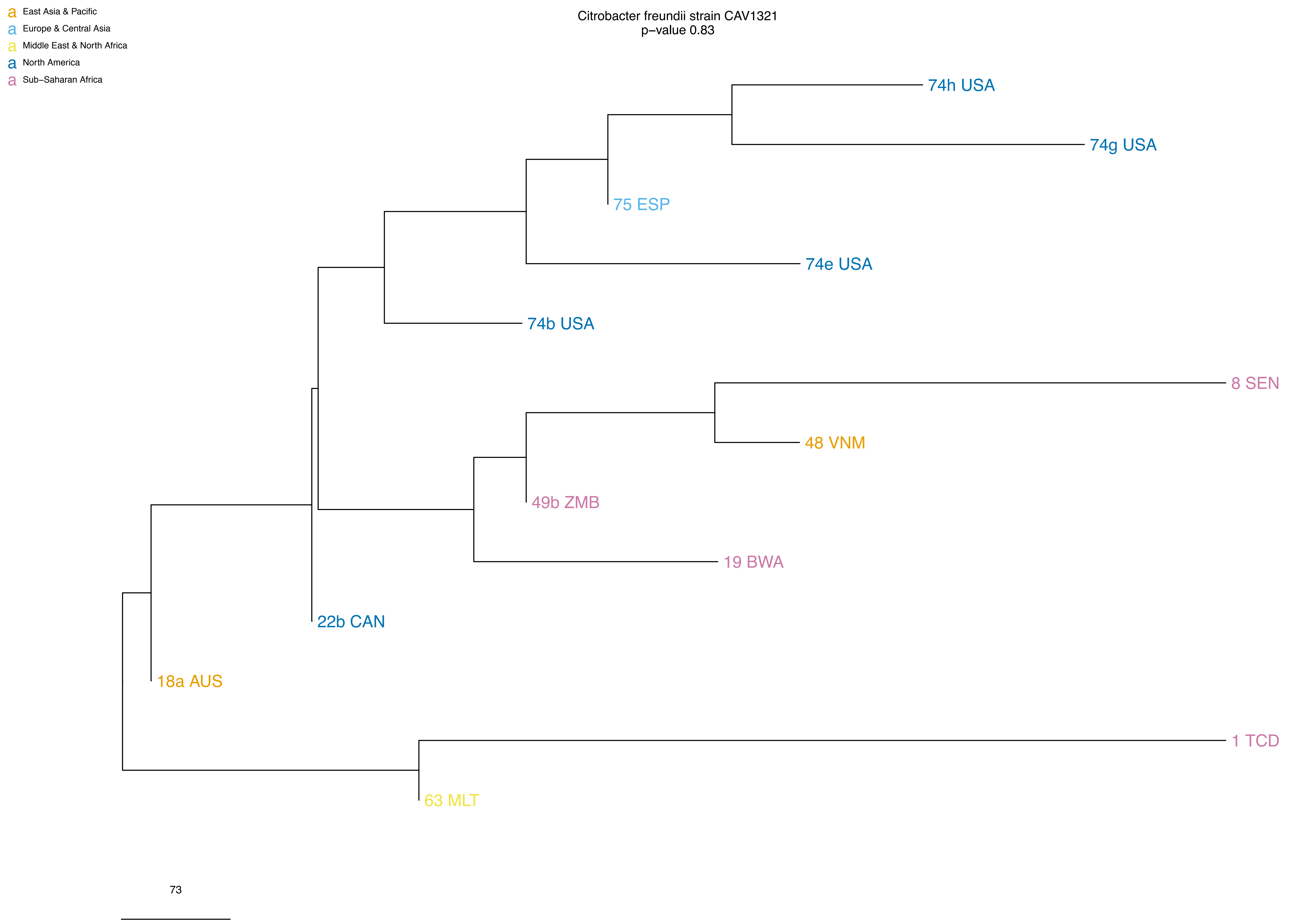

a East Asia & Pacific
a Europe & Central Asia
a Middle East & North Africa
a North America
a Sub–Saharan Africa
Citrobacter freundii strain CAV1321
p–value 0.83
74h USA
74g USA
75 ESP
74e USA
74b USA
8 SEN
48 VNM
49b ZMB
19 BWA
22b CAN
18a AUS
1 TCD
63 MLT
73

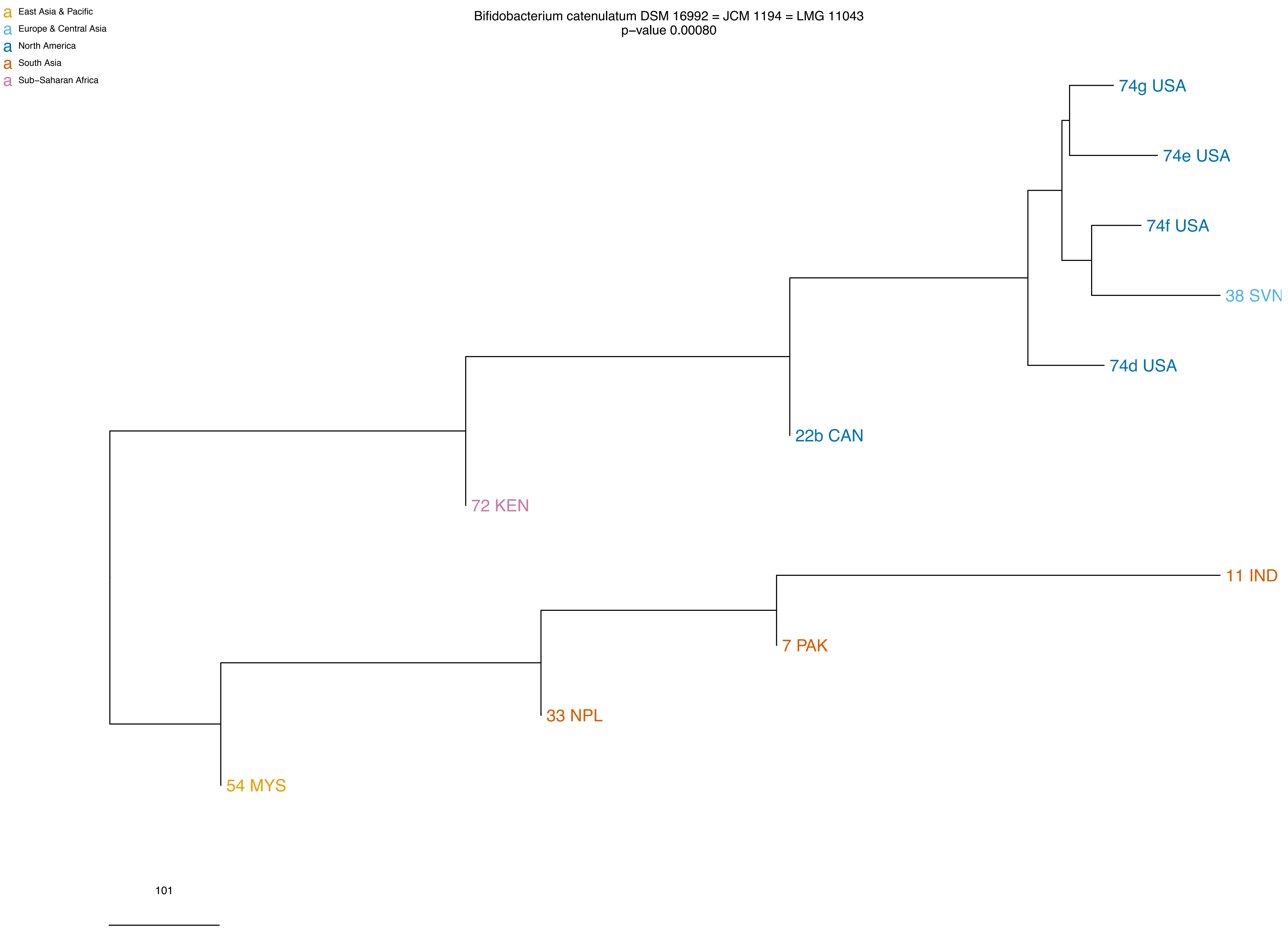

East Asia & Pacific
Europe & Central Asia
North America
South Asia
Sub–Saharan Africa
Bifidobacterium catenulatum DSM 16992 = JCM 1194 = LMG 11043
p–value 0.00080
74g USA
74e USA
74f USA
38 SVN
74d USA
22b CAN
72 KEN
11 IND
7 PAK
33 NPL
54 MYS
101

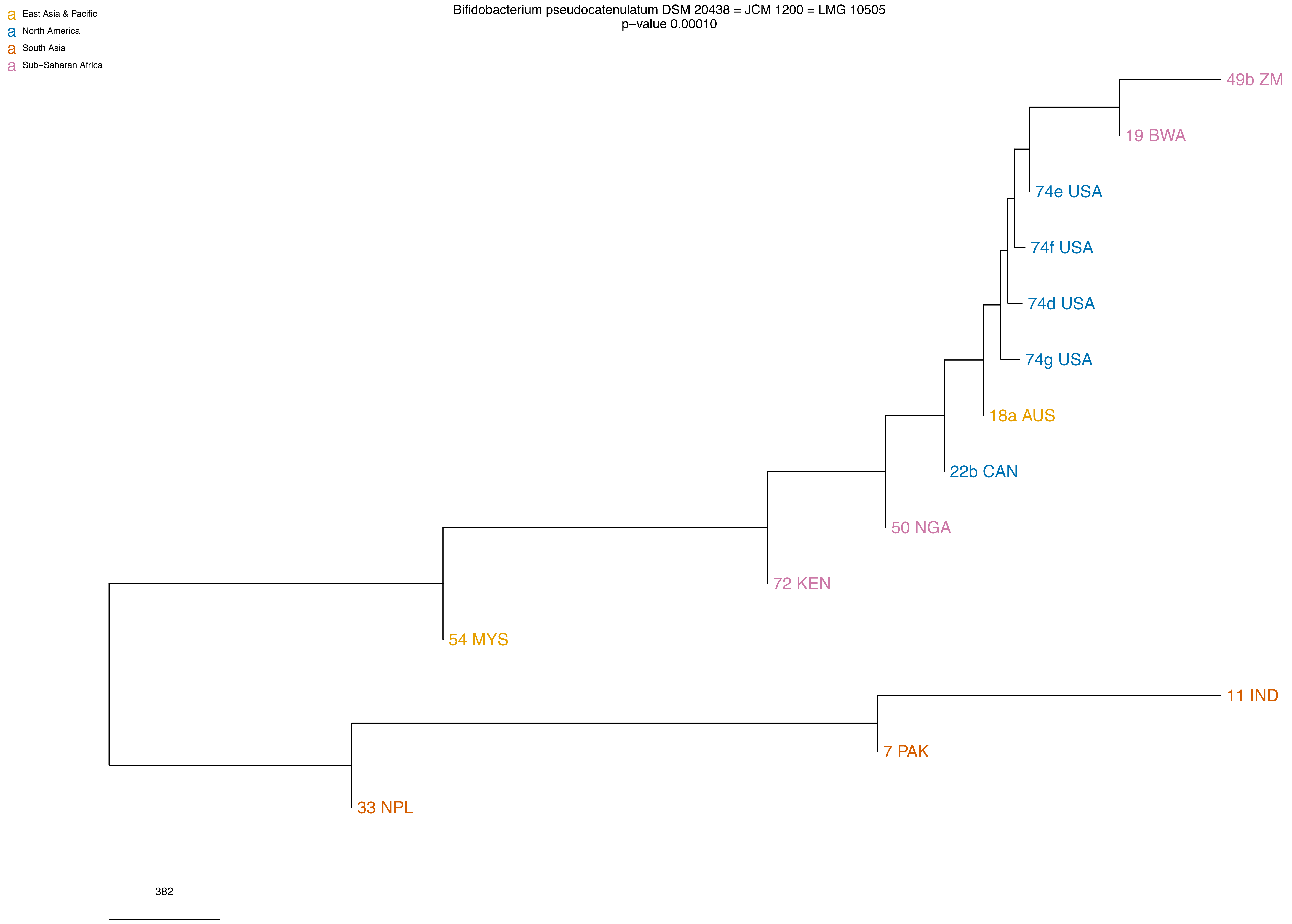

a East Asia & Pacific
a North America
a South Asia
a Sub–Saharan Africa
Bifidobacterium pseudocatenulatum DSM 20438 = JCM 1200 = LMG 10505
p–value 0.00010
49b ZM
19 BWA
74e USA
74f USA
74d USA
74g USA
18a AUS
22b CAN
50 NGA
72 KEN
54 MYS
11 IND
7 PAK
33 NPL
382

Bifidobacterium kashiwanohense JCM 15439 = DSM 21854 DNA
p–value 0.20

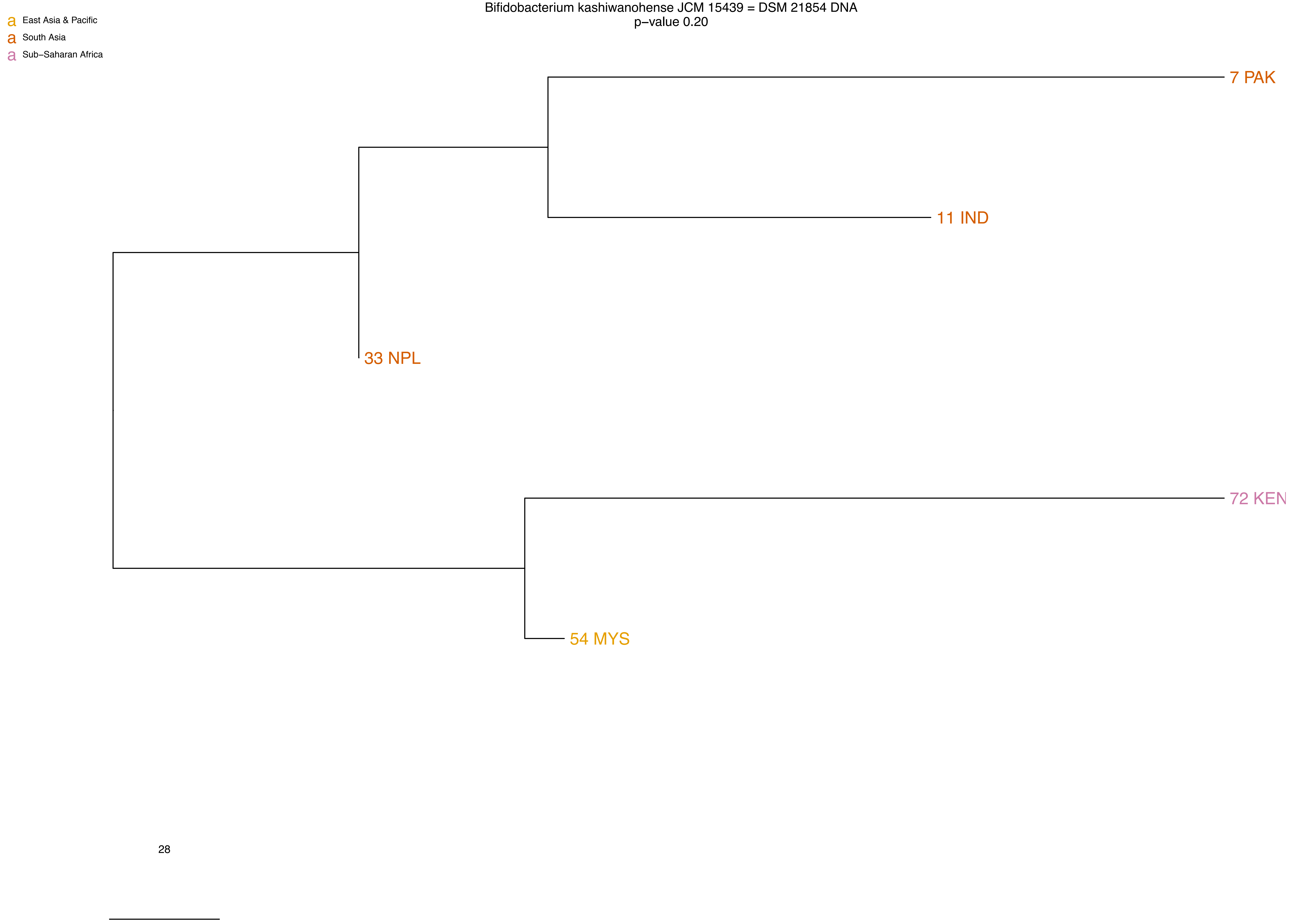

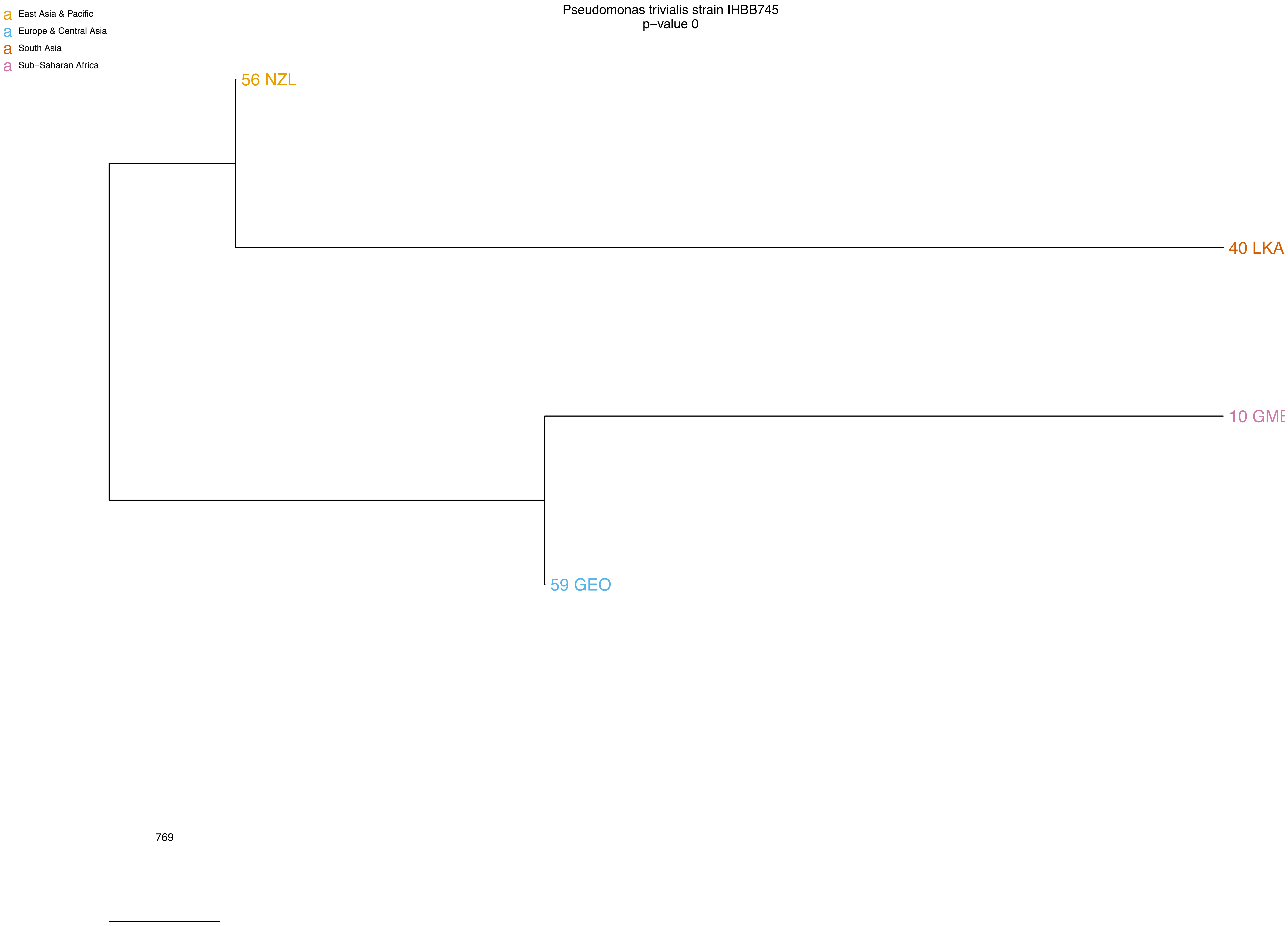

Pseudomonas trivialis strain IHBB745
p−value 0
a East Asia & Pacific
a Europe & Central Asia
a South Asia
a Sub−Saharan Africa
56 NZL
40 LKA
10 GMB
59 GEO
769

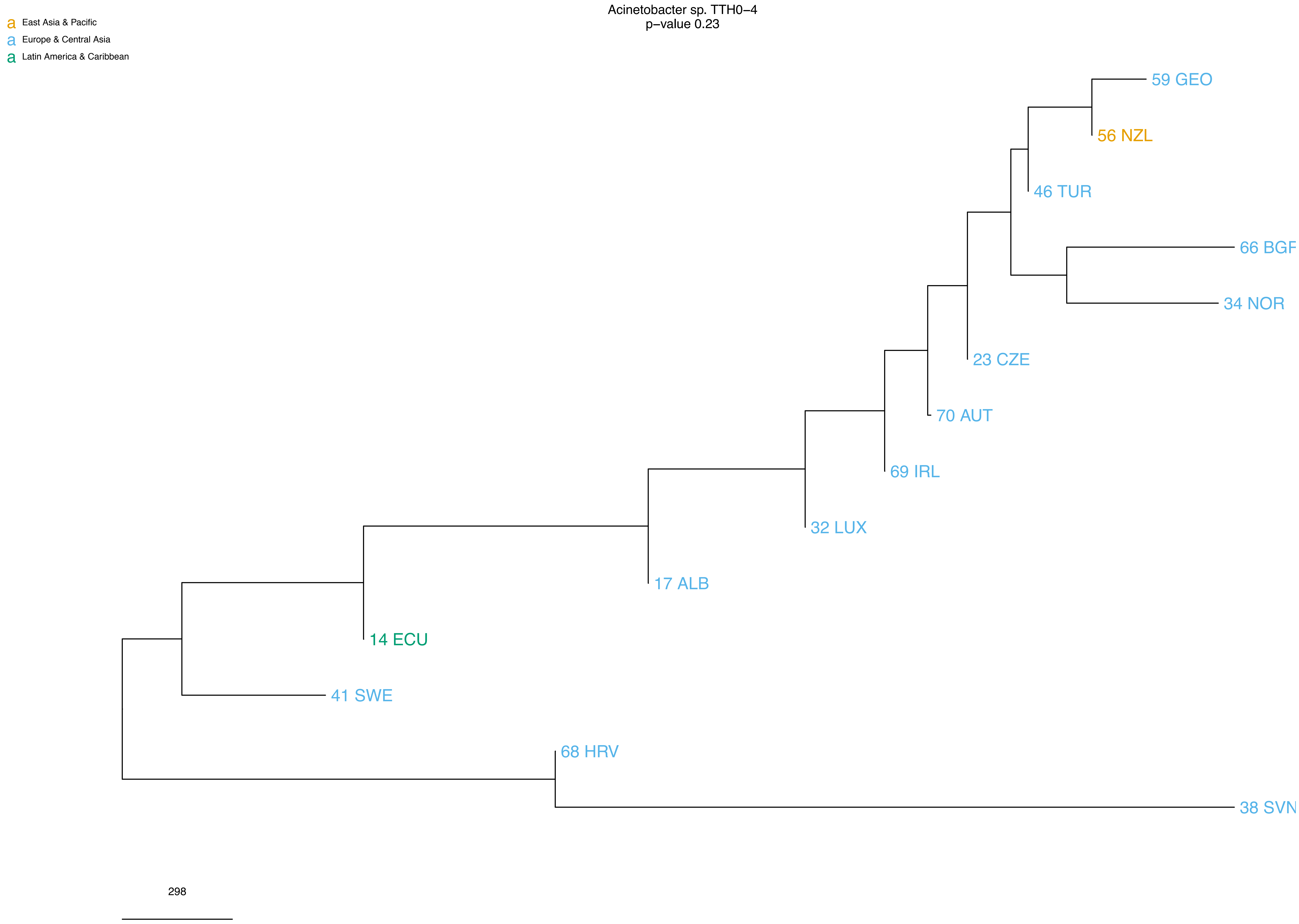
Acinetobacter sp. TTH0–4
p–value 0.23
East Asia & Pacific
Europe & Central Asia
Latin America & Caribbean
59 GEO
56 NZL
46 TUR
66 BGR
34 NOR
23 CZE
70 AUT
69 IRL
32 LUX
17 ALB
14 ECU
41 SWE
68 HRV
38 SVN
298

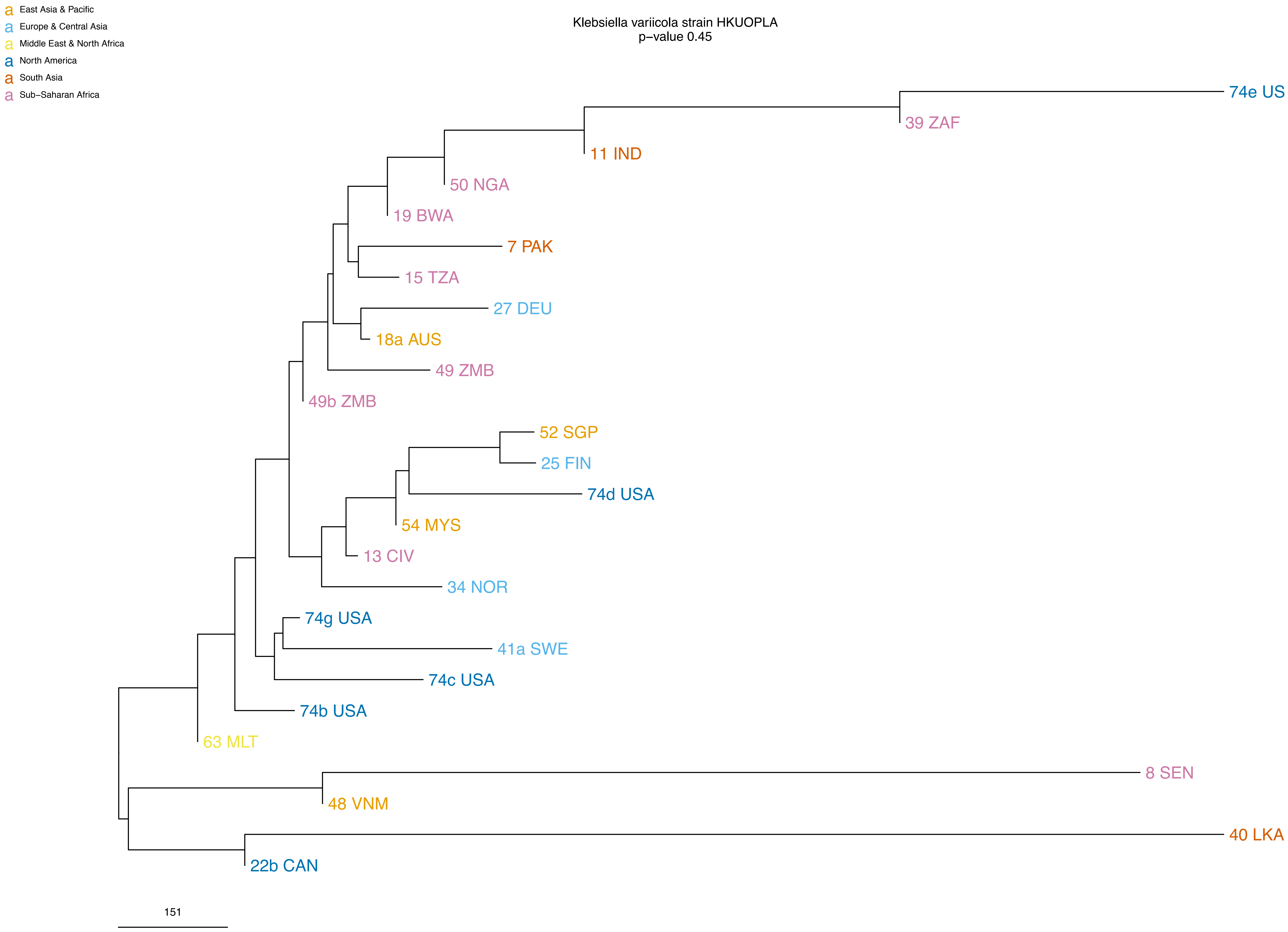

East Asia & Pacific
Europe & Central Asia
Middle East & North Africa
North America
South Asia
Sub–Saharan Africa
Klebsiella variicola strain HKUOPLA
p–value 0.45
74e US
39 ZAF
11 IND
50 NGA
19 BWA
7 PAK
15 TZA
27 DEU
18a AUS
49 ZMB
49b ZMB
52 SGP
25 FIN
74d USA
54 MYS
13 CIV
34 NOR
74g USA
41a SWE
74c USA
74b USA
63 MLT
8 SEN
48 VNM
40 LKA
22b CAN
151

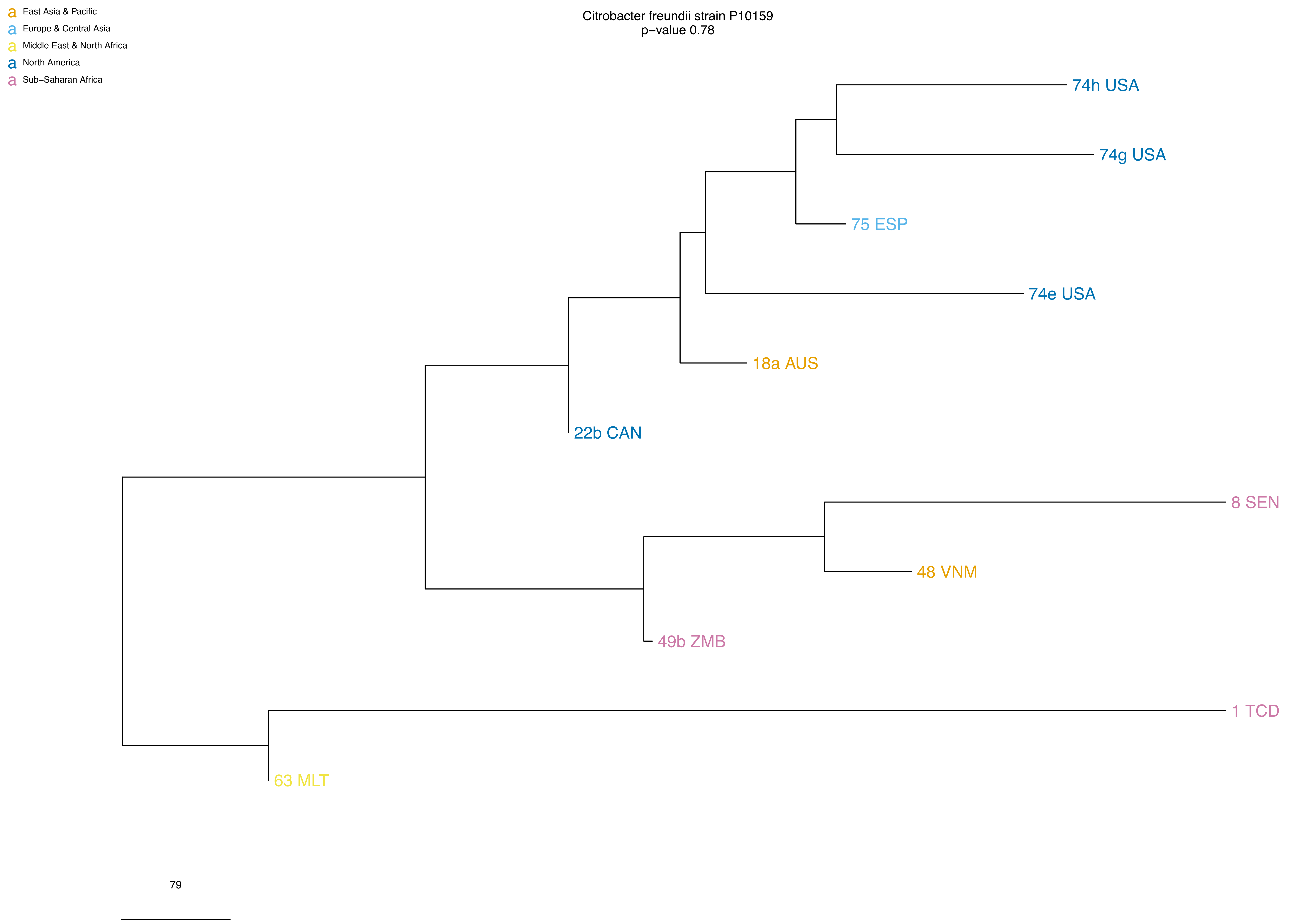

a East Asia & Pacific
a Europe & Central Asia
a Middle East & North Africa
a North America
a Sub–Saharan Africa
Citrobacter freundii strain P10159
p–value 0.78
74h USA
74g USA
75 ESP
74e USA
18a AUS
22b CAN
8 SEN
48 VNM
49b ZMB
1 TCD
63 MLT
79

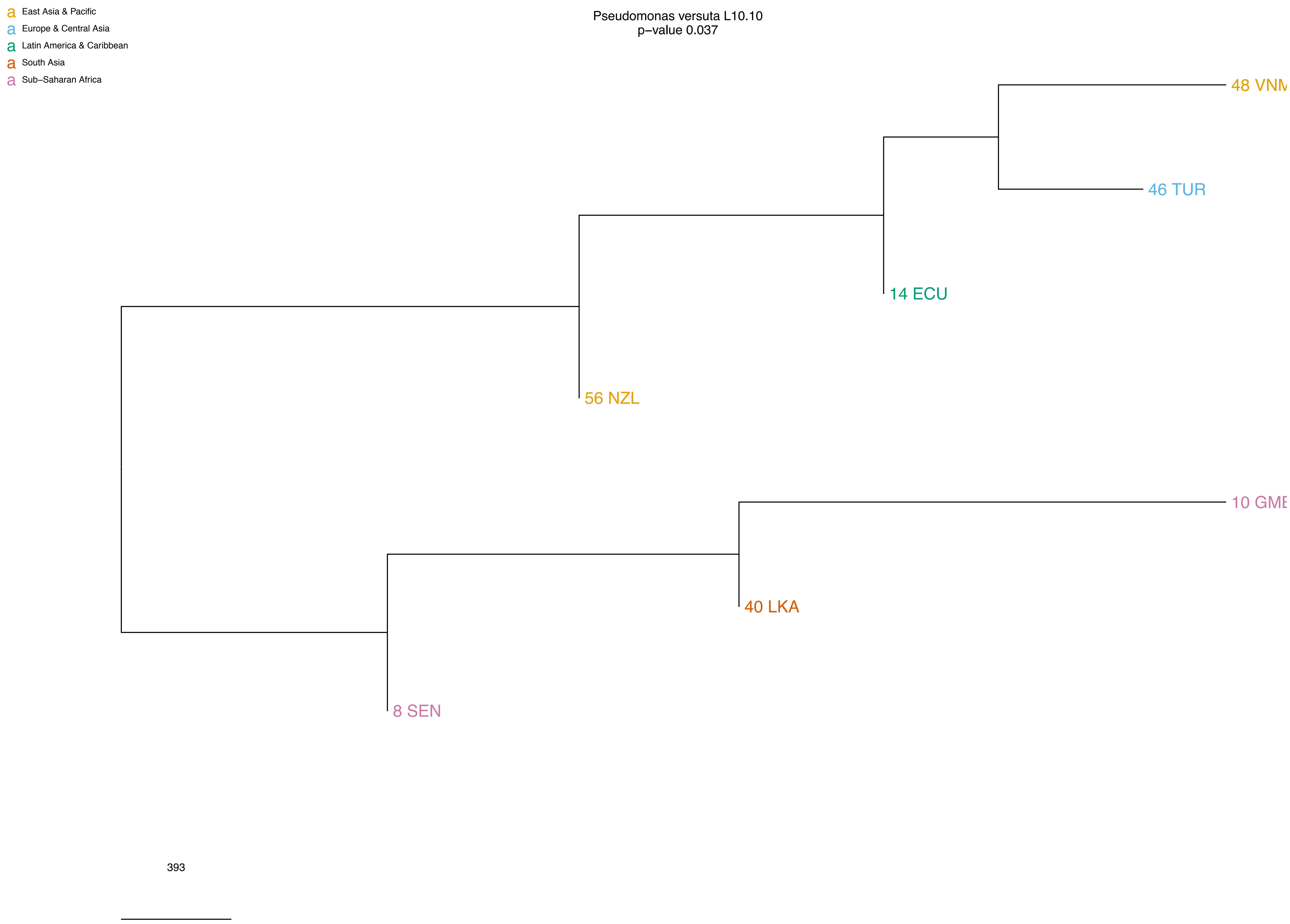

East Asia & Pacific
Europe & Central Asia
Latin America & Caribbean
South Asia
Sub–Saharan Africa
Pseudomonas versuta L10.10
p–value 0.037
48 VNM
46 TUR
14 ECU
56 NZL
10 GMB
40 LKA
8 SEN
393

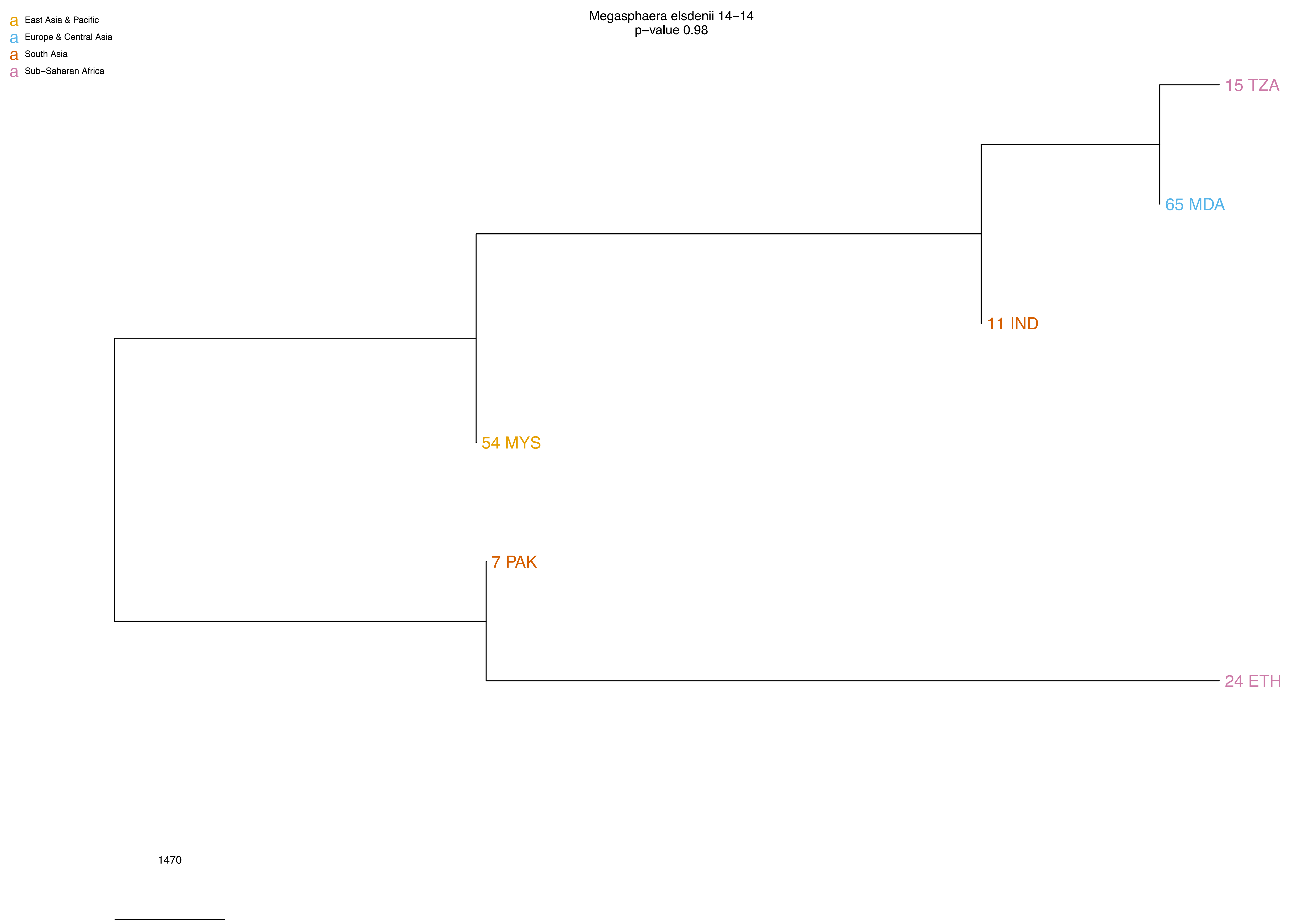

a East Asia & Pacific
a Europe & Central Asia
a South Asia
a Sub−Saharan Africa
Megasphaera elsdenii 14−14
p−value 0.98
15 TZA
65 MDA
11 IND
54 MYS
7 PAK
24 ETH
1470

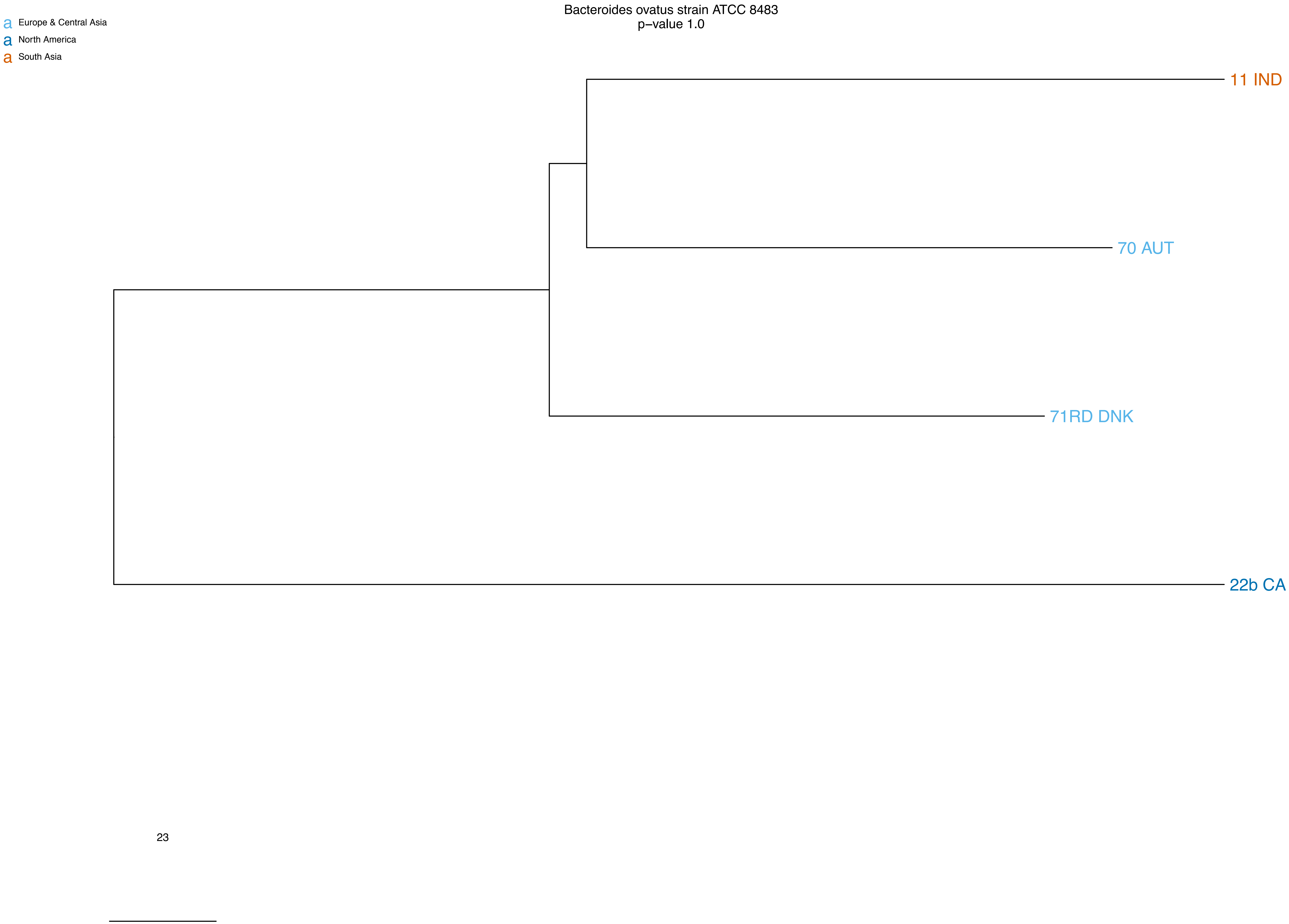

Europe & Central Asia
North America
South Asia
Bacteroides ovatus strain ATCC 8483
p–value 1.0
11 IND
70 AUT
71RD DNK
22b CA
23

Comamonas testosteroni P19
p−value 0.64

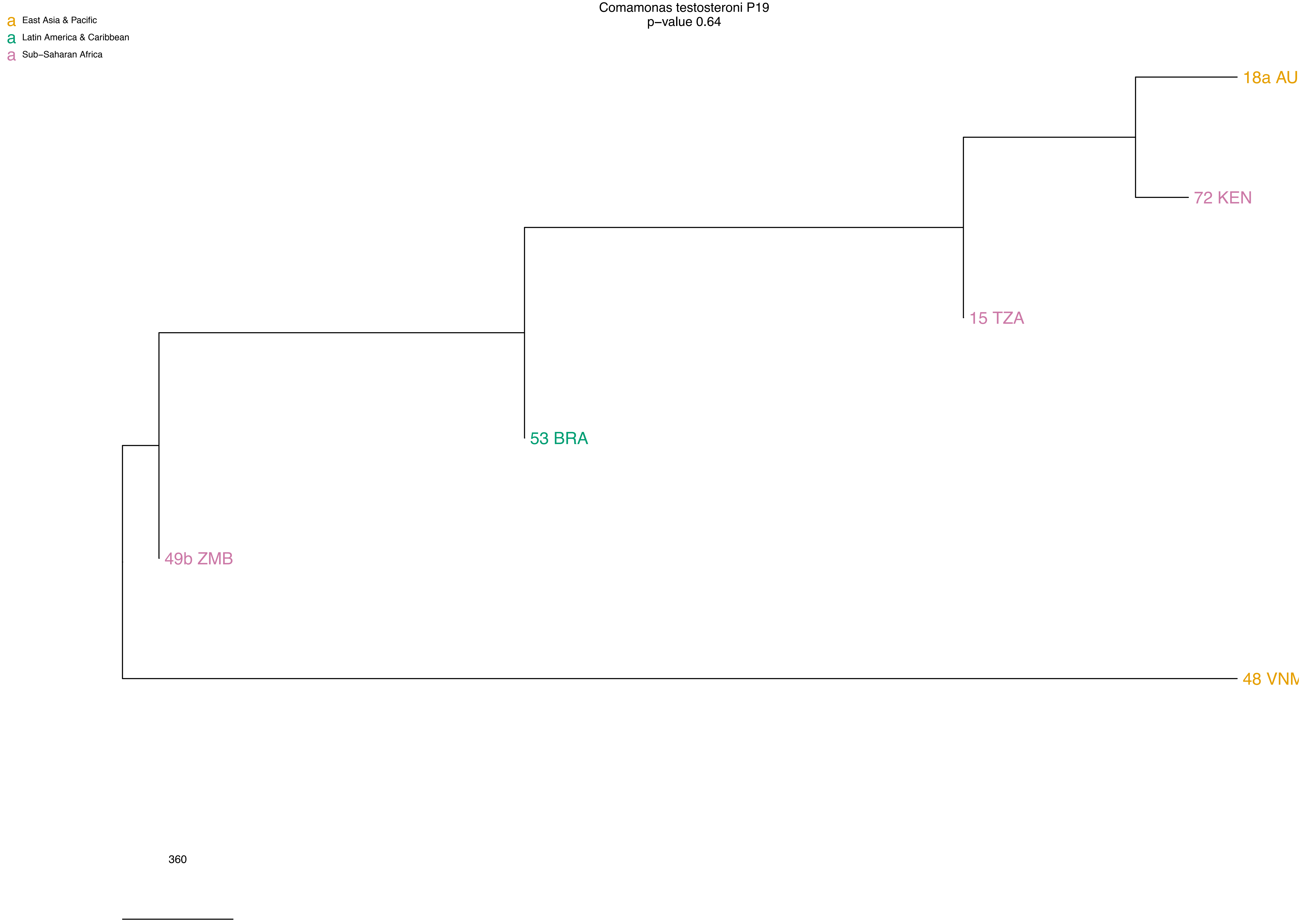

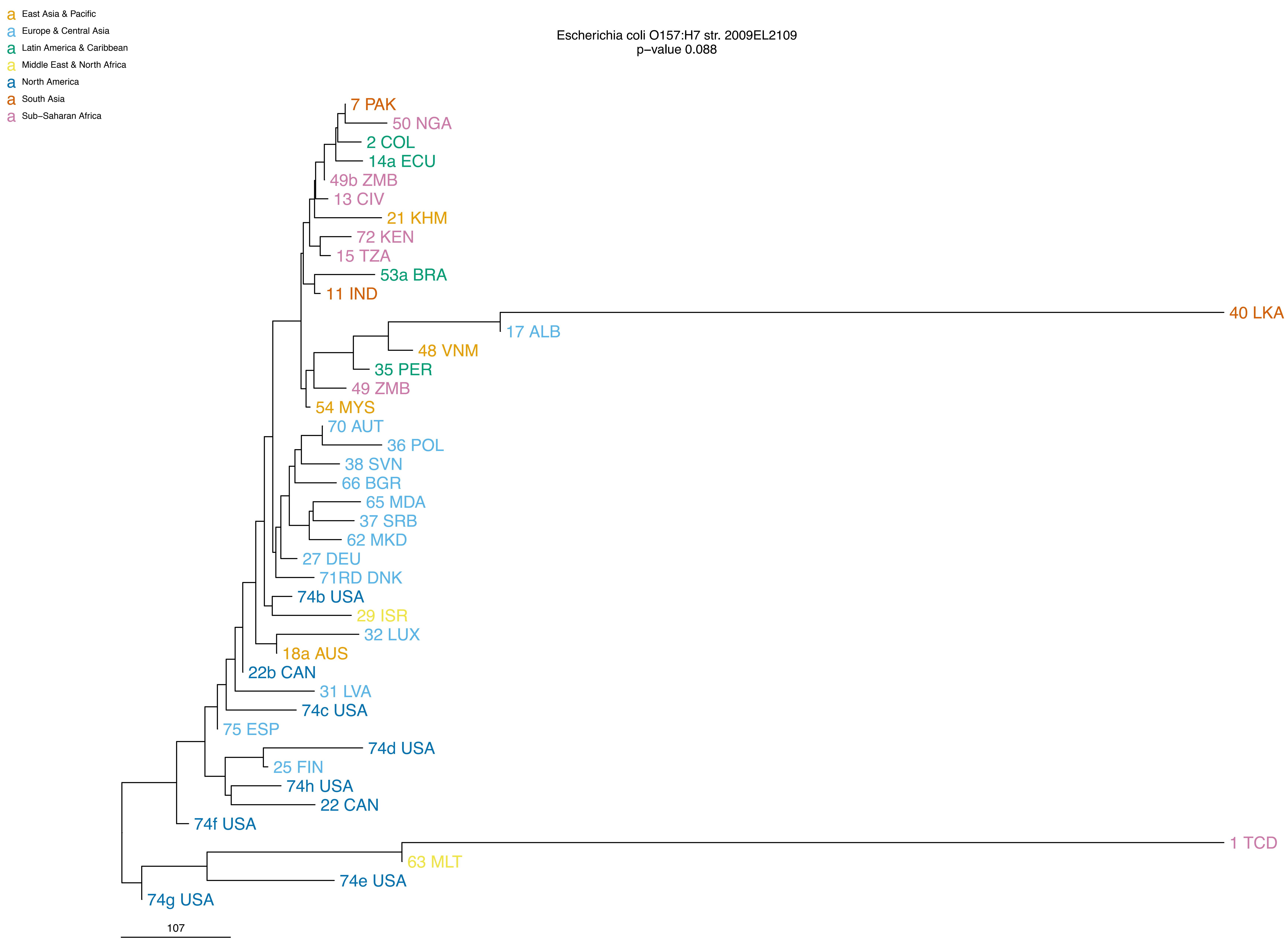

East Asia & Pacific
Europe & Central Asia
Latin America & Caribbean
Middle East & North Africa
North America
South Asia
Sub–Saharan Africa
Escherichia coli O157:H7 str. 2009EL2109
p–value 0.088
7 PAK
50 NGA
2 COL
14a ECU
49b ZMB
13 CIV
21 KHM
72 KEN
15 TZA
53a BRA
11 IND
40 LKA
17 ALB
48 VNM
35 PER
49 ZMB
54 MYS
70 AUT
36 POL
38 SVN
66 BGR
65 MDA
37 SRB
62 MKD
27 DEU
71RD DNK
74b USA
29 ISR
32 LUX
18a AUS
22b CAN
31 LVA
74c USA
75 ESP
74d USA
25 FIN
74h USA
22 CAN
74f USA
1 TCD
63 MLT
74e USA
74g USA
107

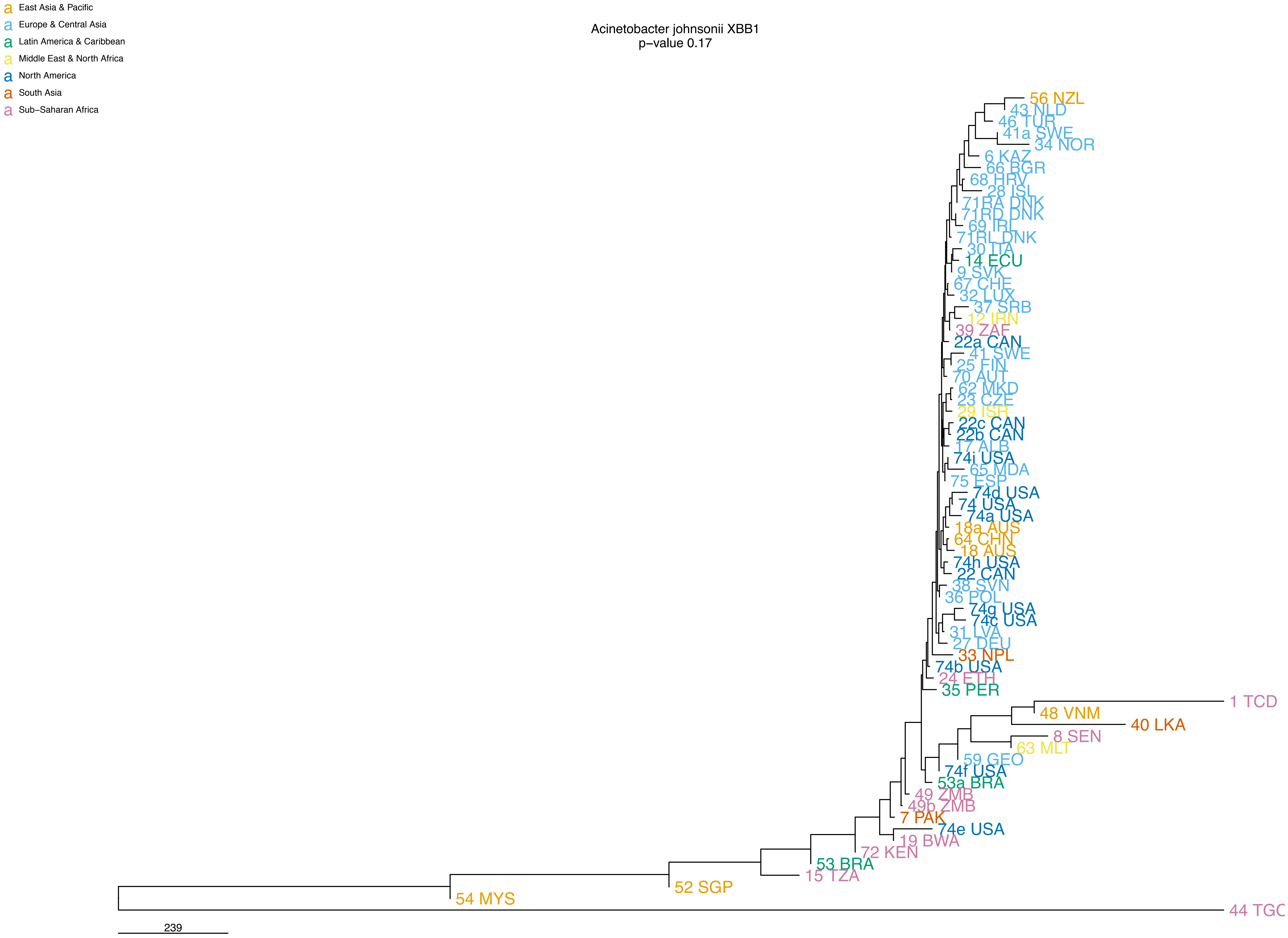
a East Asia & Pacific
a Europe & Central Asia
a Latin America & Caribbean
a Middle East & North Africa
a North America
a South Asia
a Sub–Saharan Africa
Acinetobacter johnsonii XBB1
p–value 0.17
56 NZL
43 NLD
46 TUR
41a SWE
34 NOR
6 KAZ
66 BGR
68 HRV
28 ISL
71RA DNK
71RD DNK
69 IRL
71RL DNK
30 ITA
14 ECU
9 SVK
67 CHE
32 LUX
37 SRB
12 IRN
39 ZAF
22a CAN
41 SWE
25 FIN
70 AUT
62 MKD
23 CZE
29 ISR
22c CAN
22b CAN
17 ALB
74i USA
65 MDA
75 ESP
74d USA
74 USA
74a USA
18a AUS
64 CHN
18 AUS
74h USA
22 CAN
38 SVN
36 POL
74g USA
74c USA
31 LVA
27 DEU
33 NPL
74b USA
24 ETH
35 PER
1 TCD
48 VNM
40 LKA
8 SEN
63 MLT
59 GEO
74f USA
53a BRA
49 ZMB
49b ZMB
7 PAK
74e USA
19 BWA
72 KEN
53 BRA
15 TZA
52 SGP
54 MYS
44 TGC
239

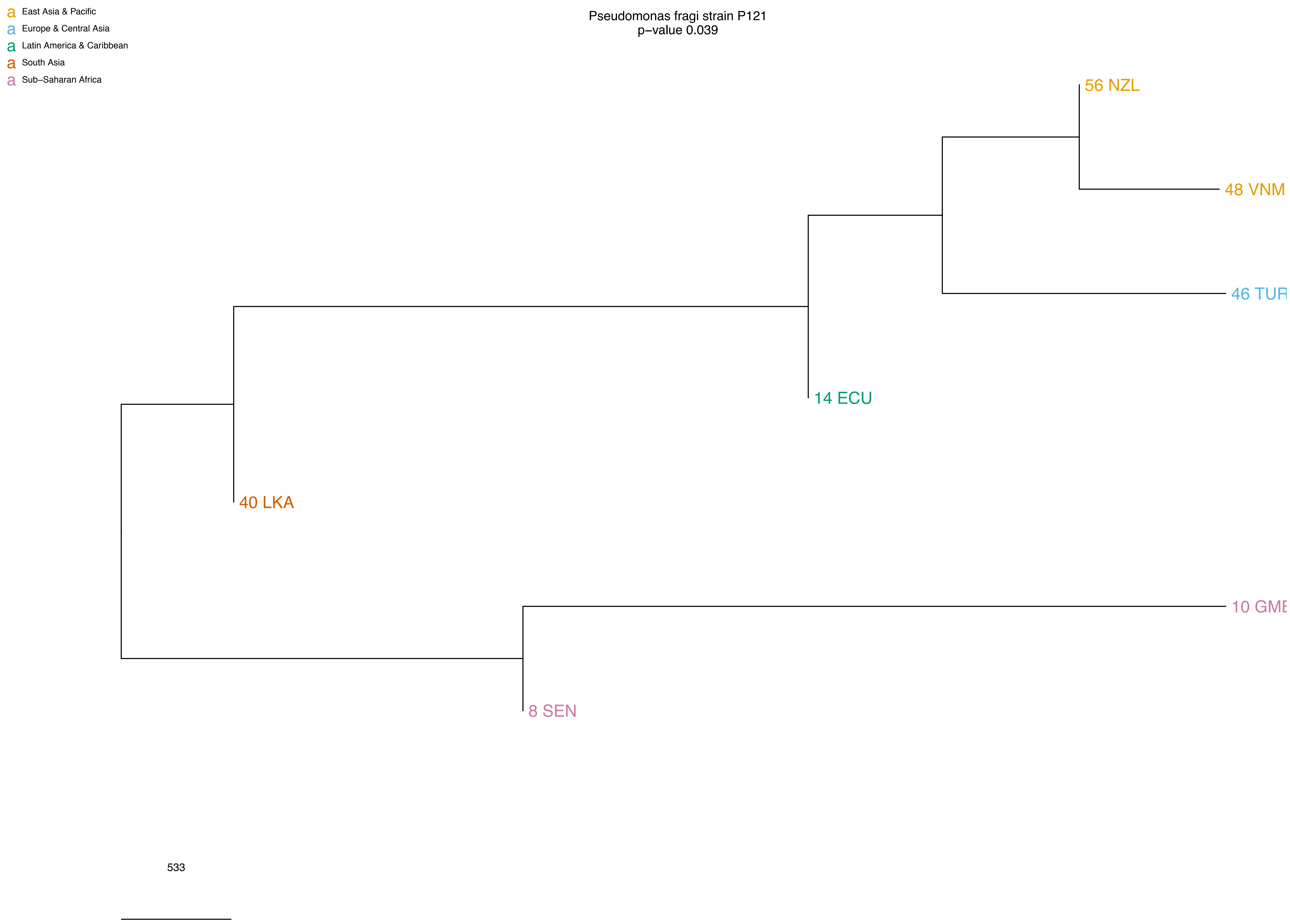

East Asia & Pacific
Europe & Central Asia
Latin America & Caribbean
South Asia
Sub–Saharan Africa
Pseudomonas fragi strain P121
p–value 0.039
56 NZL
48 VNM
46 TUR
14 ECU
40 LKA
10 GMB
8 SEN
533

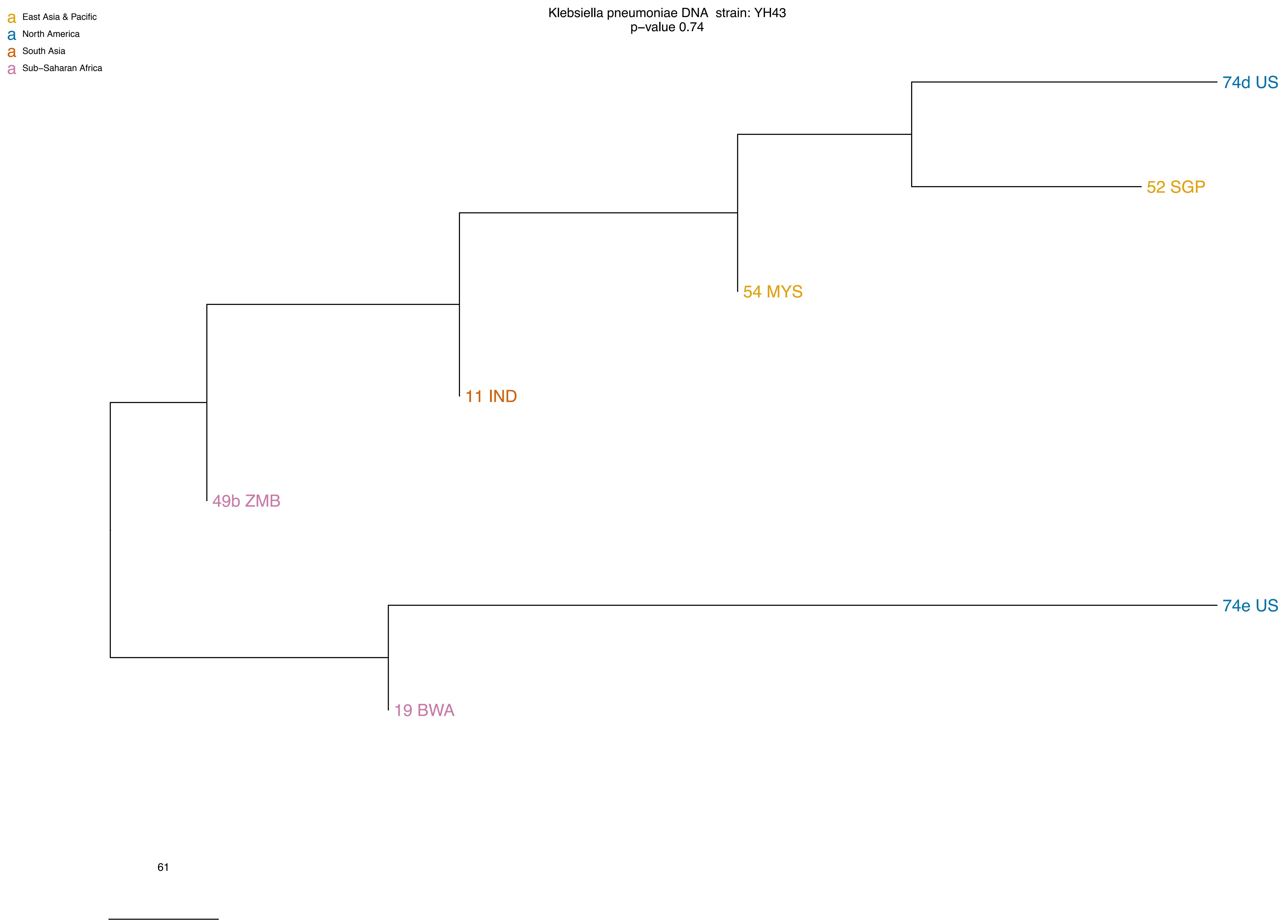

Klebsiella pneumoniae DNA strain: YH43
p−value 0.74
a East Asia & Pacific
a North America
a South Asia
a Sub−Saharan Africa
74d US
52 SGP
54 MYS
11 IND
49b ZMB
74e US
19 BWA
61

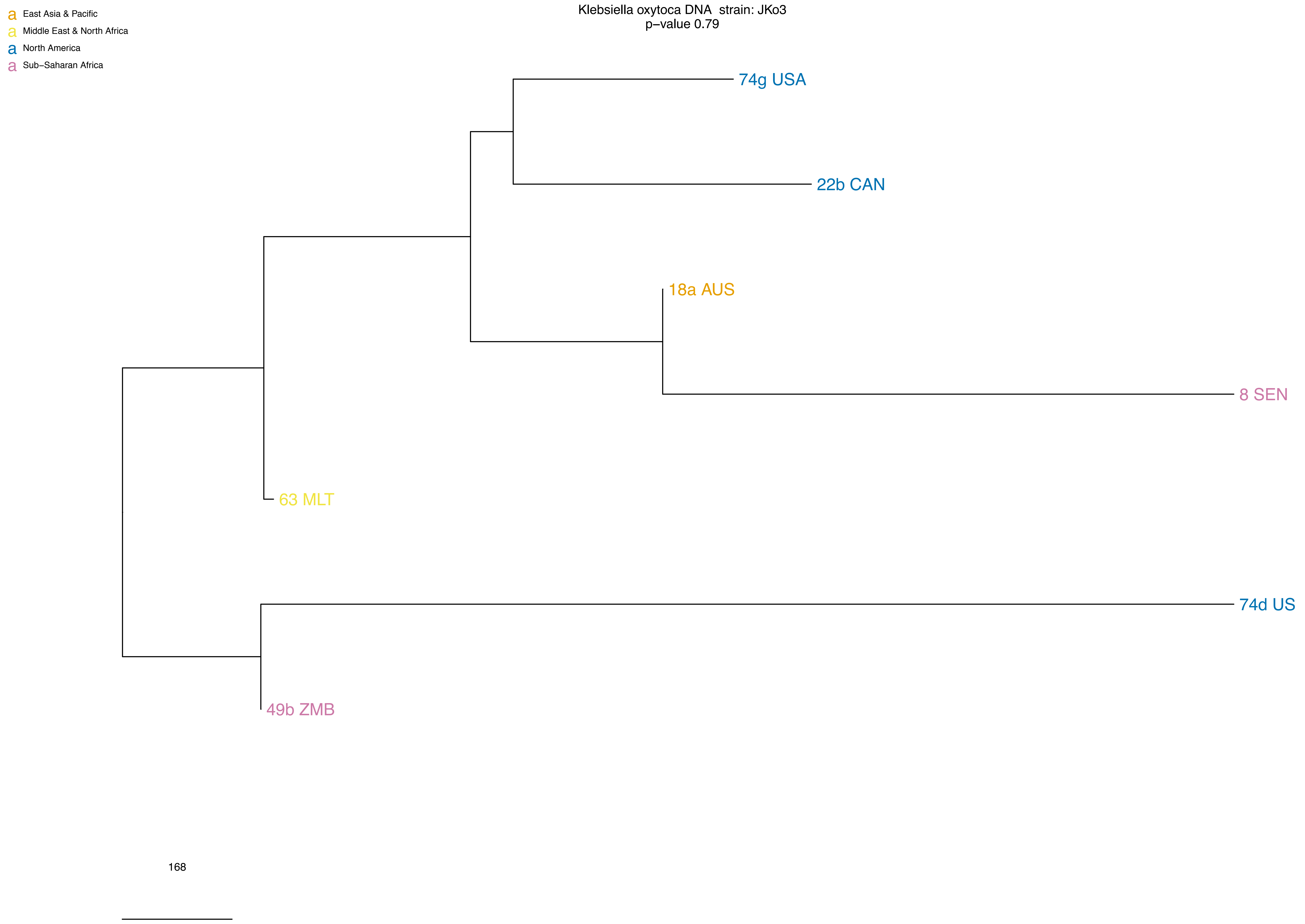
Klebsiella oxytoca DNA strain: JKo3
p–value 0.79
a East Asia & Pacific
a Middle East & North Africa
a North America
a Sub–Saharan Africa
74g USA
22b CAN
18a AUS
8 SEN
63 MLT
74d US
49b ZMB
168

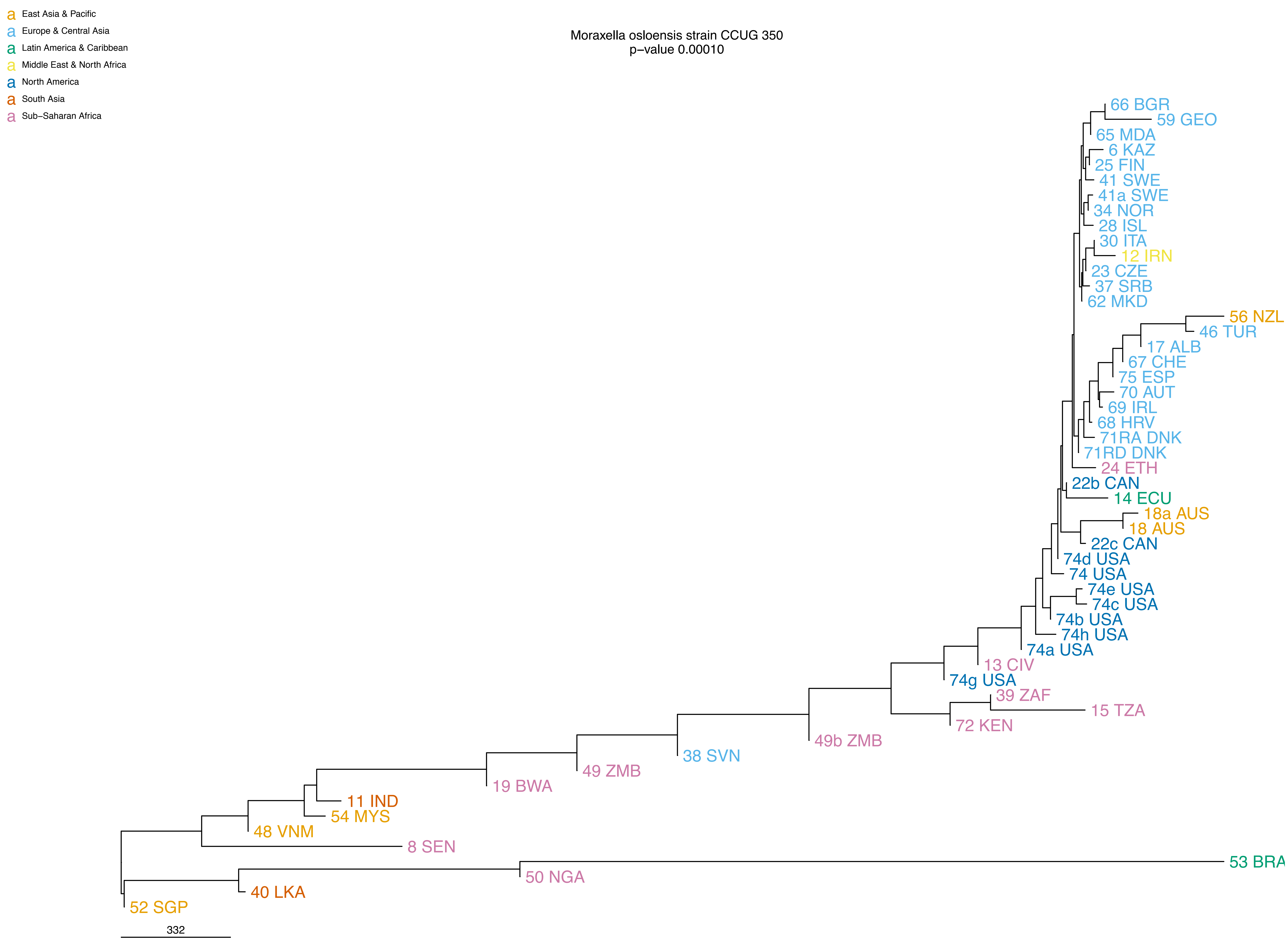
a East Asia & Pacific
a Europe & Central Asia
a Latin America & Caribbean
a Middle East & North Africa
a North America
a South Asia
a Sub–Saharan Africa
Moraxella osloensis strain CCUG 350
p–value 0.00010
66 BGR
59 GEO
65 MDA
6 KAZ
25 FIN
41 SWE
41a SWE
34 NOR
28 ISL
30 ITA
12 IRN
23 CZE
37 SRB
62 MKD
56 NZL
46 TUR
17 ALB
67 CHE
75 ESP
70 AUT
69 IRL
68 HRV
71RA DNK
71RD DNK
24 ETH
22b CAN
14 ECU
18a AUS
18 AUS
22c CAN
74d USA
74 USA
74e USA
74c USA
74b USA
74h USA
74a USA
13 CIV
74g USA
39 ZAF
15 TZA
72 KEN
49b ZMB
38 SVN
49 ZMB
19 BWA
11 IND
54 MYS
48 VNM
8 SEN
53 BRA
50 NGA
40 LKA
52 SGP
332

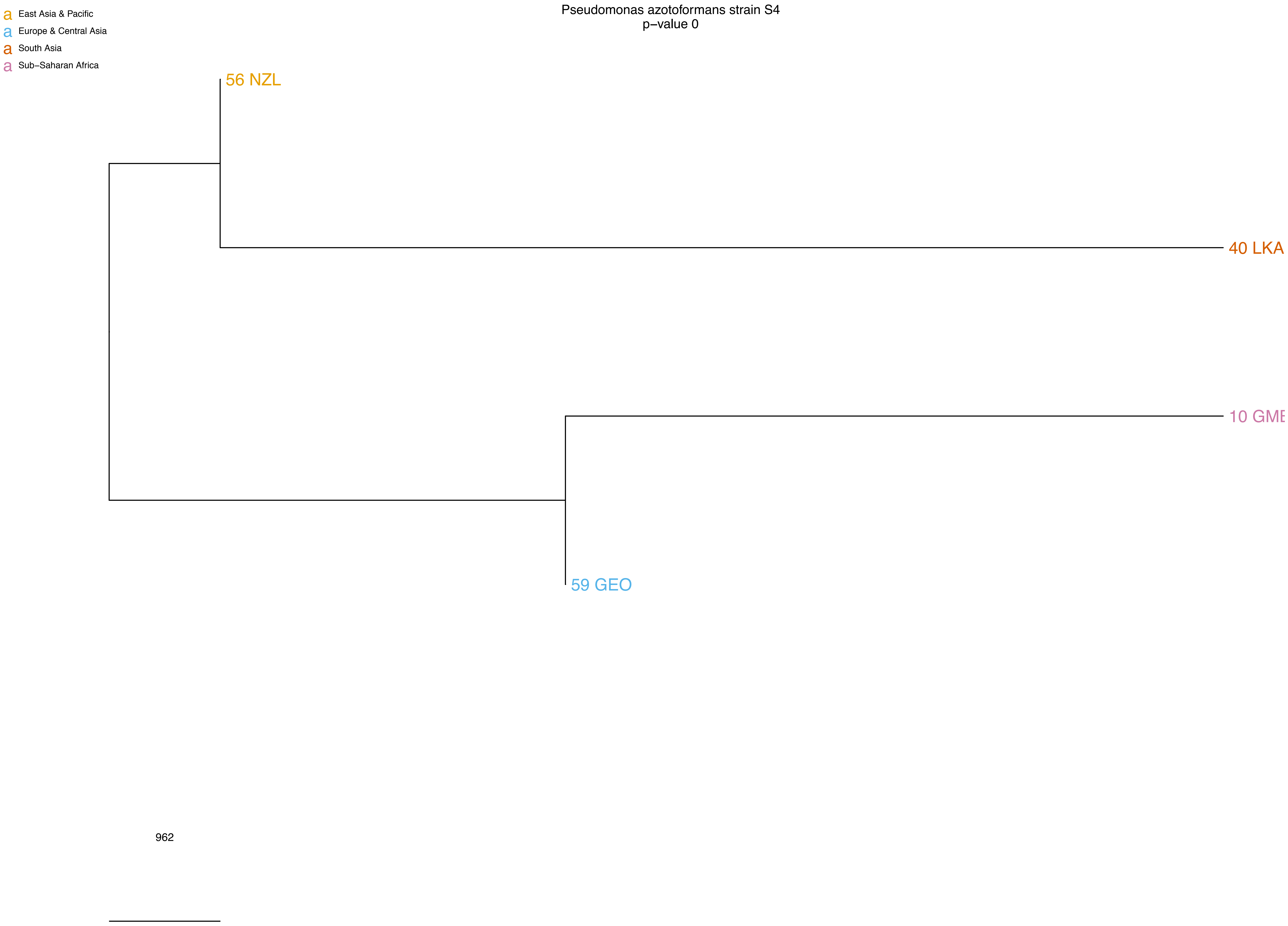

Pseudomonas azotoformans strain S4
p−value 0
East Asia & Pacific
Europe & Central Asia
South Asia
Sub−Saharan Africa
56 NZL
40 LKA
10 GMB
59 GEO
962

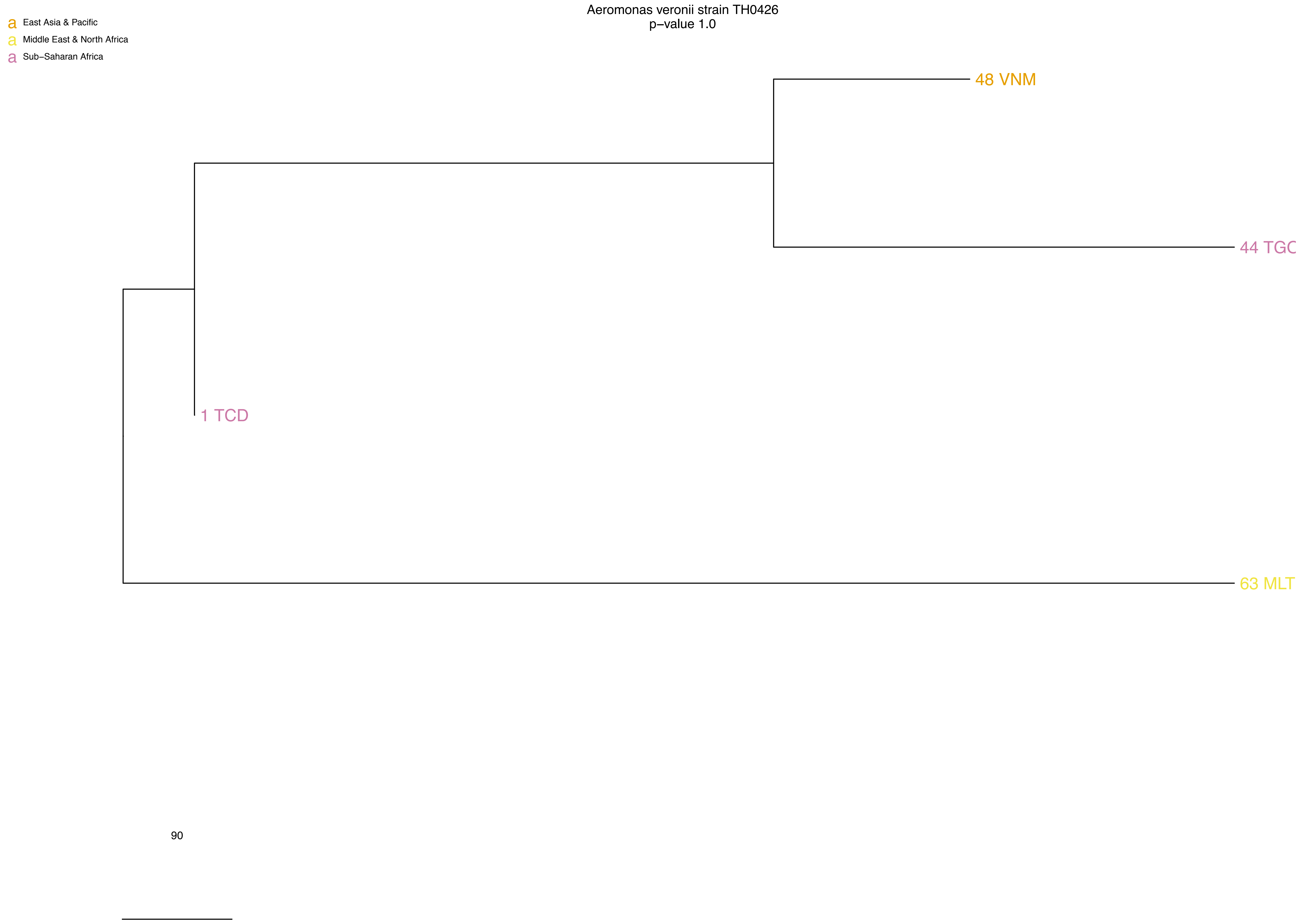

Aeromonas veronii strain TH0426
p–value 1.0
East Asia & Pacific
Middle East & North Africa
Sub–Saharan Africa
48 VNM
44 TGC
1 TCD
63 MLT
90

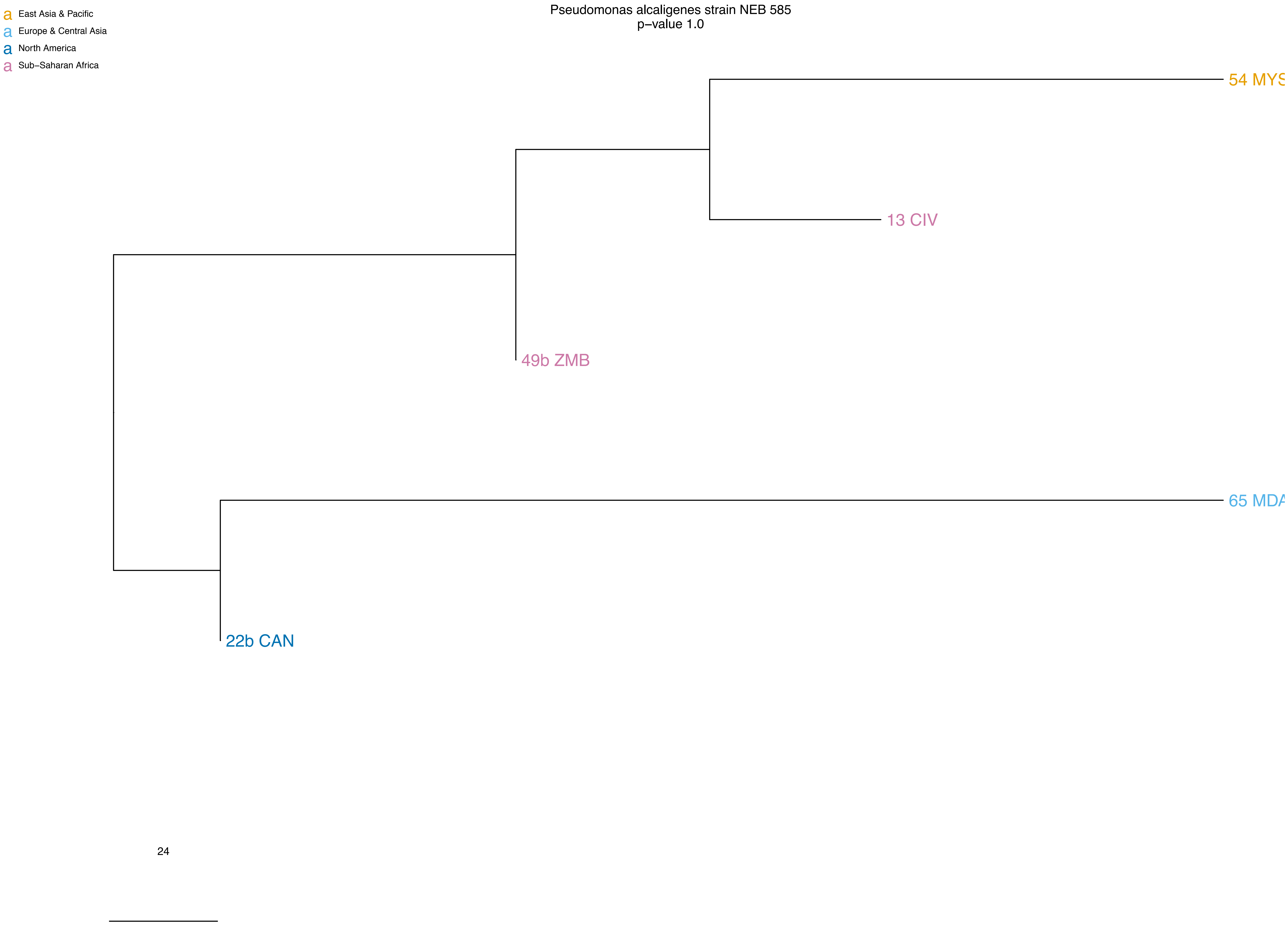

a East Asia & Pacific
a Europe & Central Asia
a North America
a Sub−Saharan Africa
Pseudomonas alcaligenes strain NEB 585
p−value 1.0
54 MYS
13 CIV
49b ZMB
65 MDA
22b CAN
24

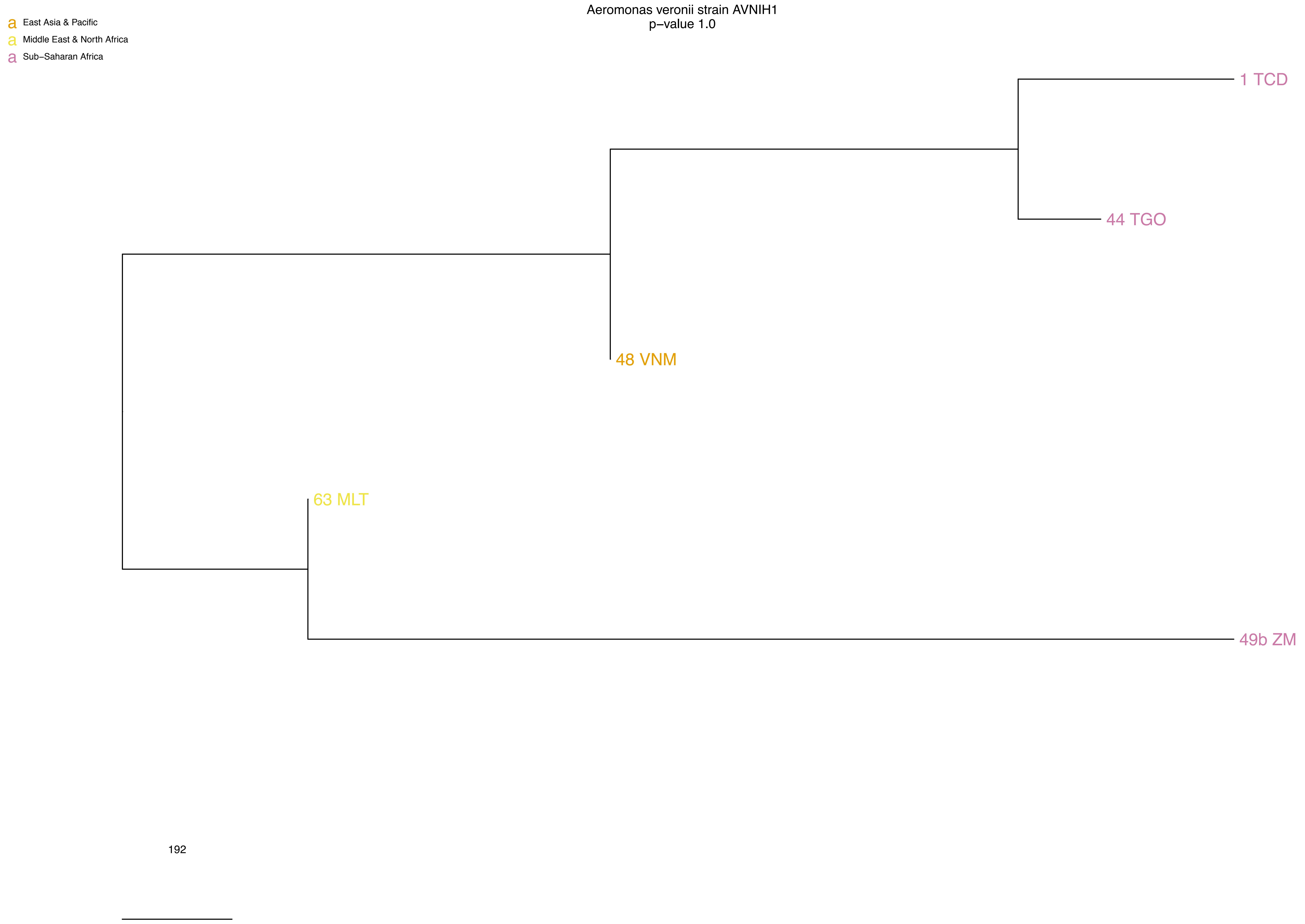
Aeromonas veronii strain AVNIH1
p–value 1.0
East Asia & Pacific
Middle East & North Africa
Sub–Saharan Africa
1 TCD
44 TGO
48 VNM
63 MLT
49b ZM
192

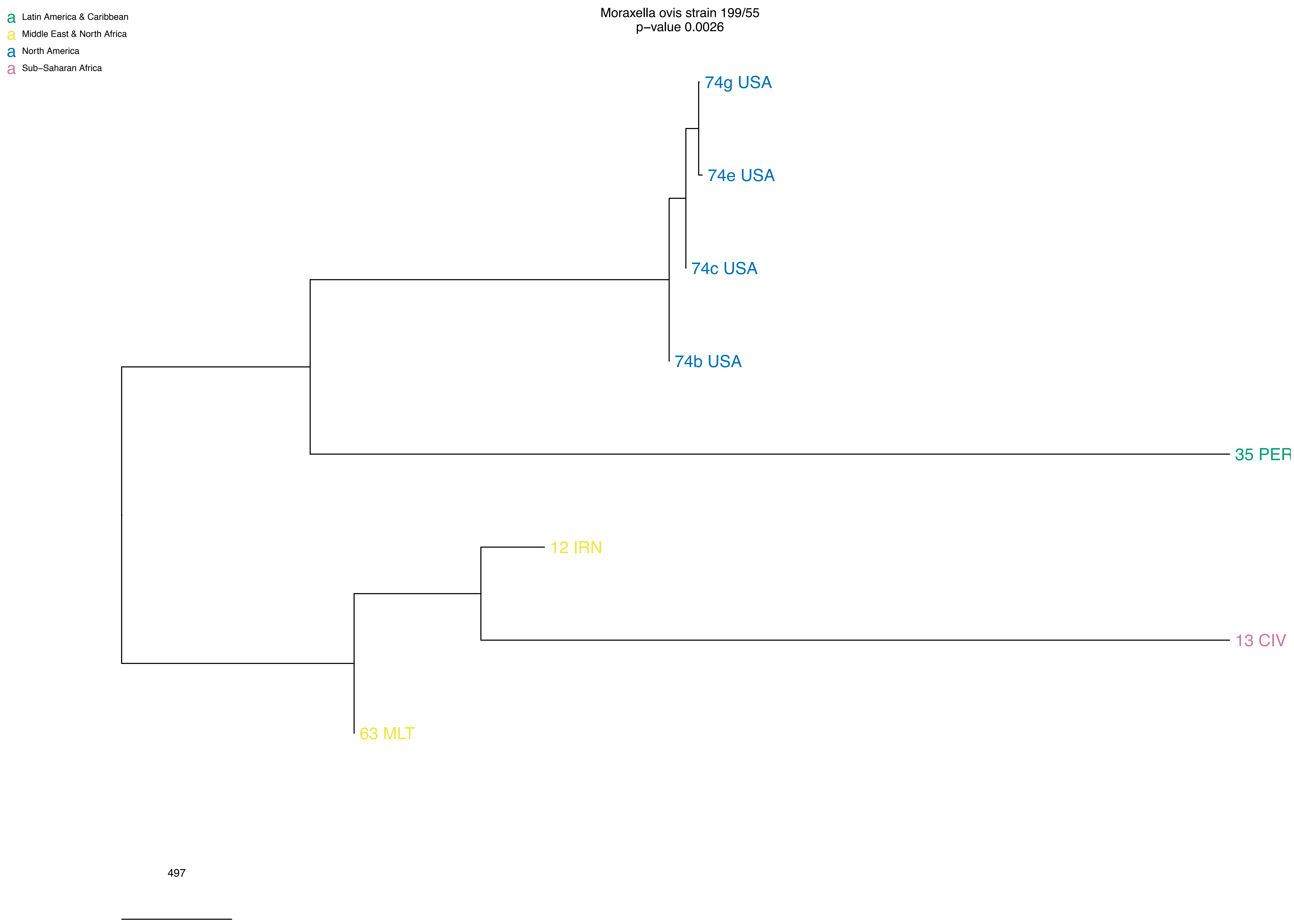

Latin America & Caribbean
Middle East & North Africa
North America
Sub–Saharan Africa
Moraxella ovis strain 199/55
p–value 0.0026
74g USA
74e USA
74c USA
74b USA
35 PER
12 IRN
13 CIV
63 MLT
497

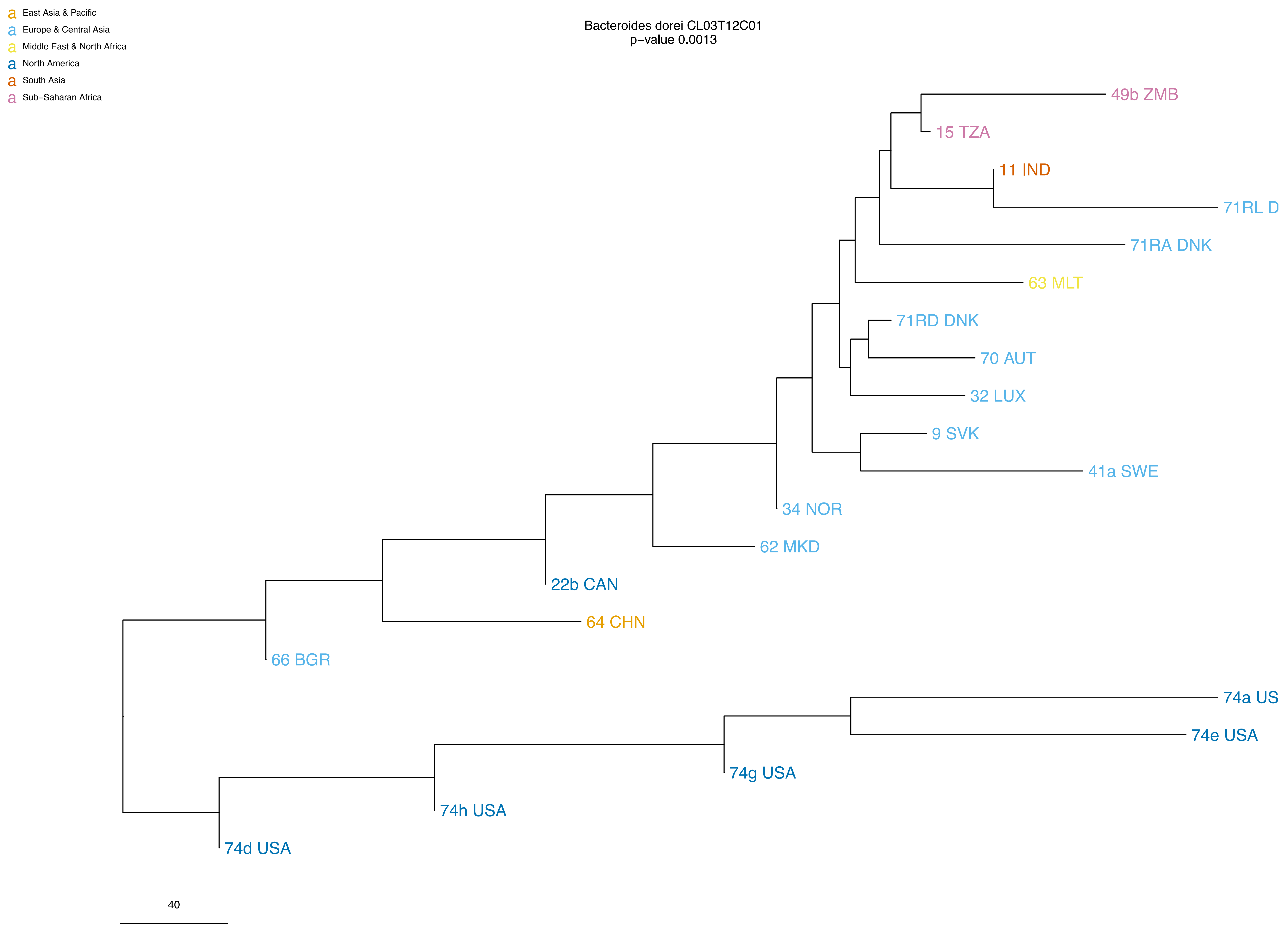

East Asia & Pacific
Europe & Central Asia
Middle East & North Africa
North America
South Asia
Sub–Saharan Africa
Bacteroides dorei CL03T12C01
p–value 0.0013
49b ZMB
15 TZA
11 IND
71RL D
71RA DNK
63 MLT
71RD DNK
70 AUT
32 LUX
9 SVK
41a SWE
34 NOR
62 MKD
22b CAN
64 CHN
66 BGR
74a US
74e USA
74g USA
74h USA
74d USA
40

Pseudomonas antarctica strain PAMC 27494
p–value 0

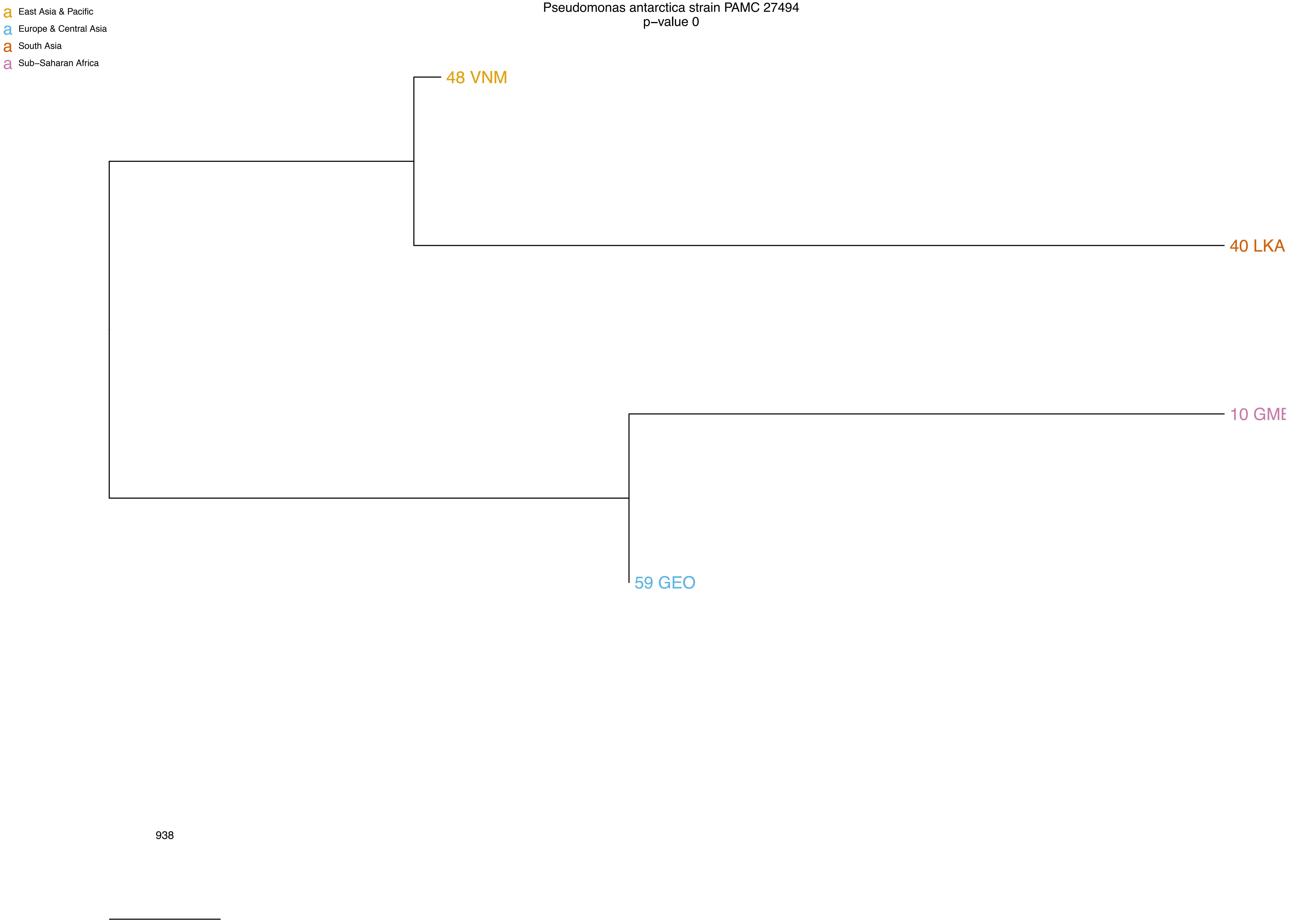

Escherichia coli O36:H14 strain 06−00048
p−value 1.0

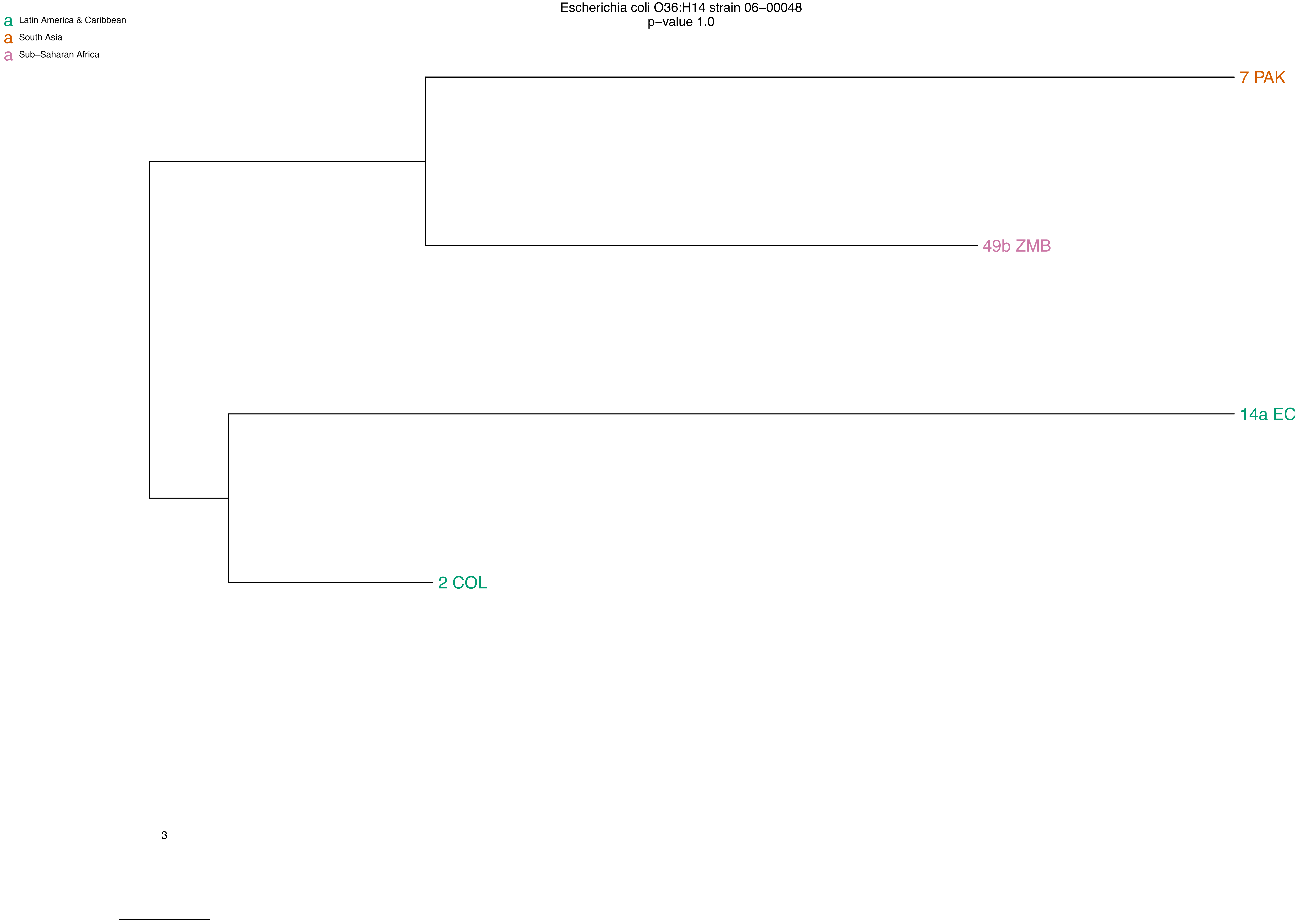

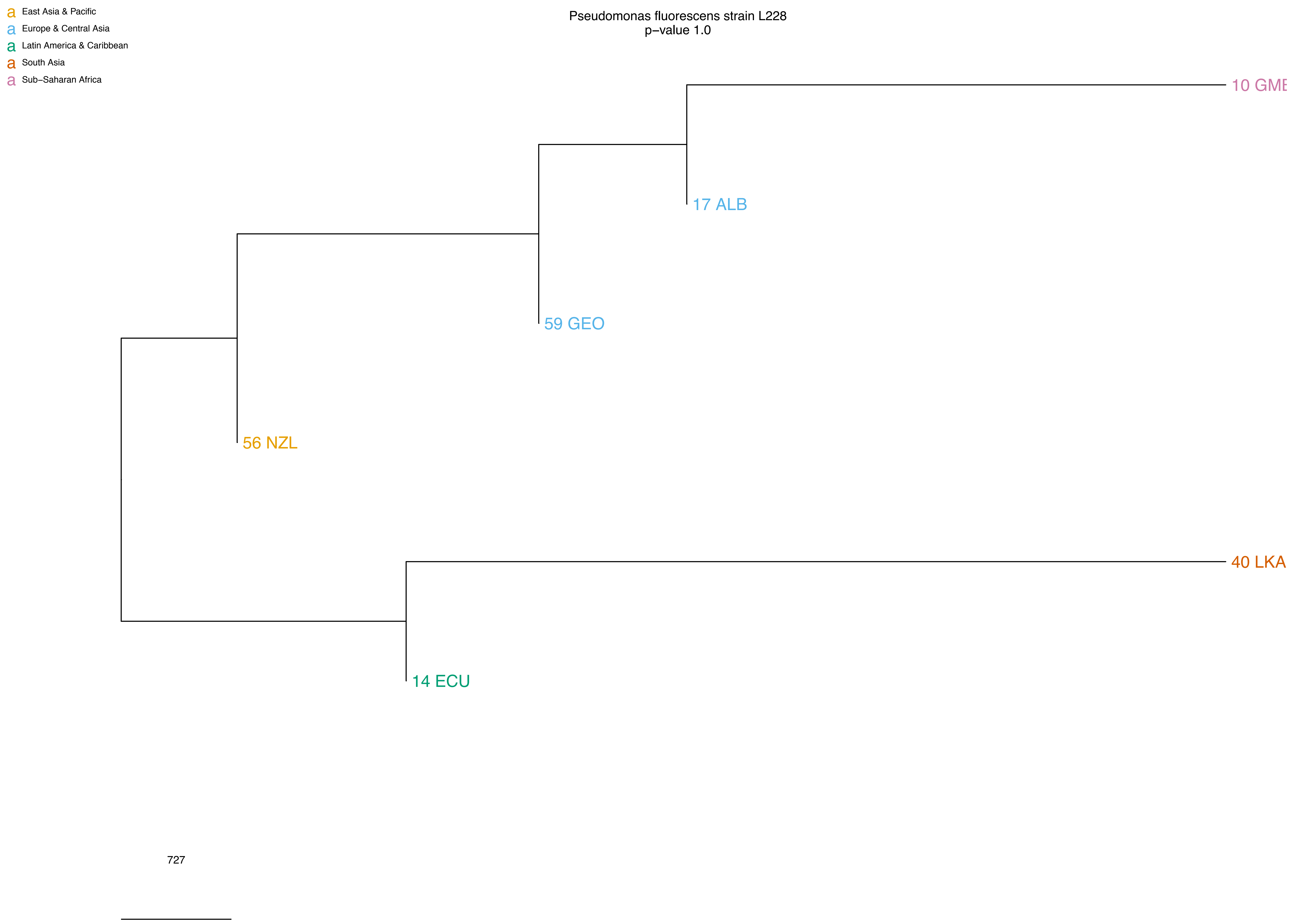
a East Asia & Pacific
a Europe & Central Asia
a Latin America & Caribbean
a South Asia
a Sub–Saharan Africa
Pseudomonas fluorescens strain L228
p–value 1.0
10 GMB
17 ALB
59 GEO
56 NZL
40 LKA
14 ECU
727

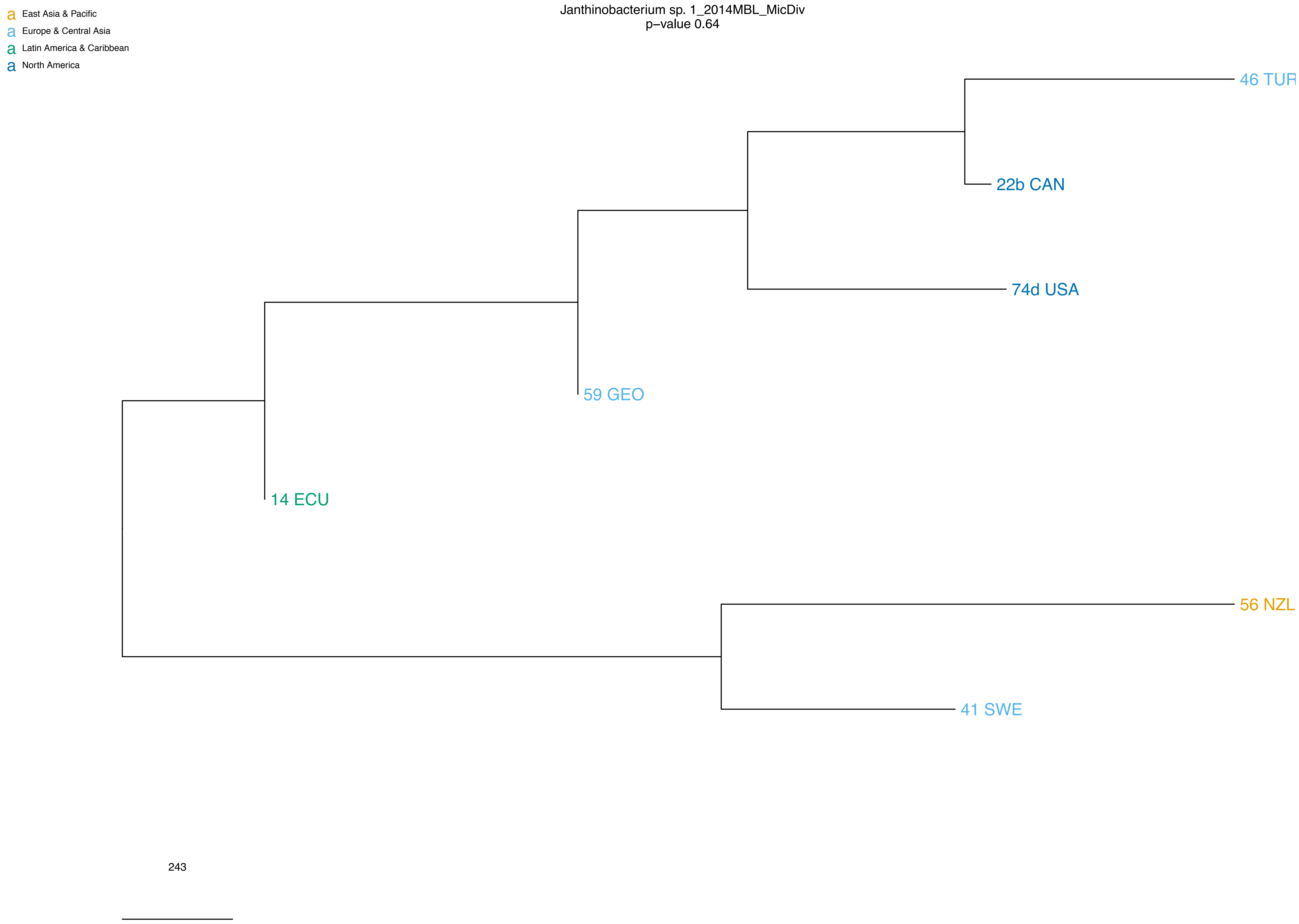

East Asia & Pacific
Europe & Central Asia
Latin America & Caribbean
North America
Janthinobacterium sp. 1_2014MBL_MicDiv
p−value 0.64
46 TUR
22b CAN
74d USA
59 GEO
14 ECU
56 NZL
41 SWE
243

Lactobacillus delbrueckii subsp. sunkii strain JCM 17838
p–value 1.0

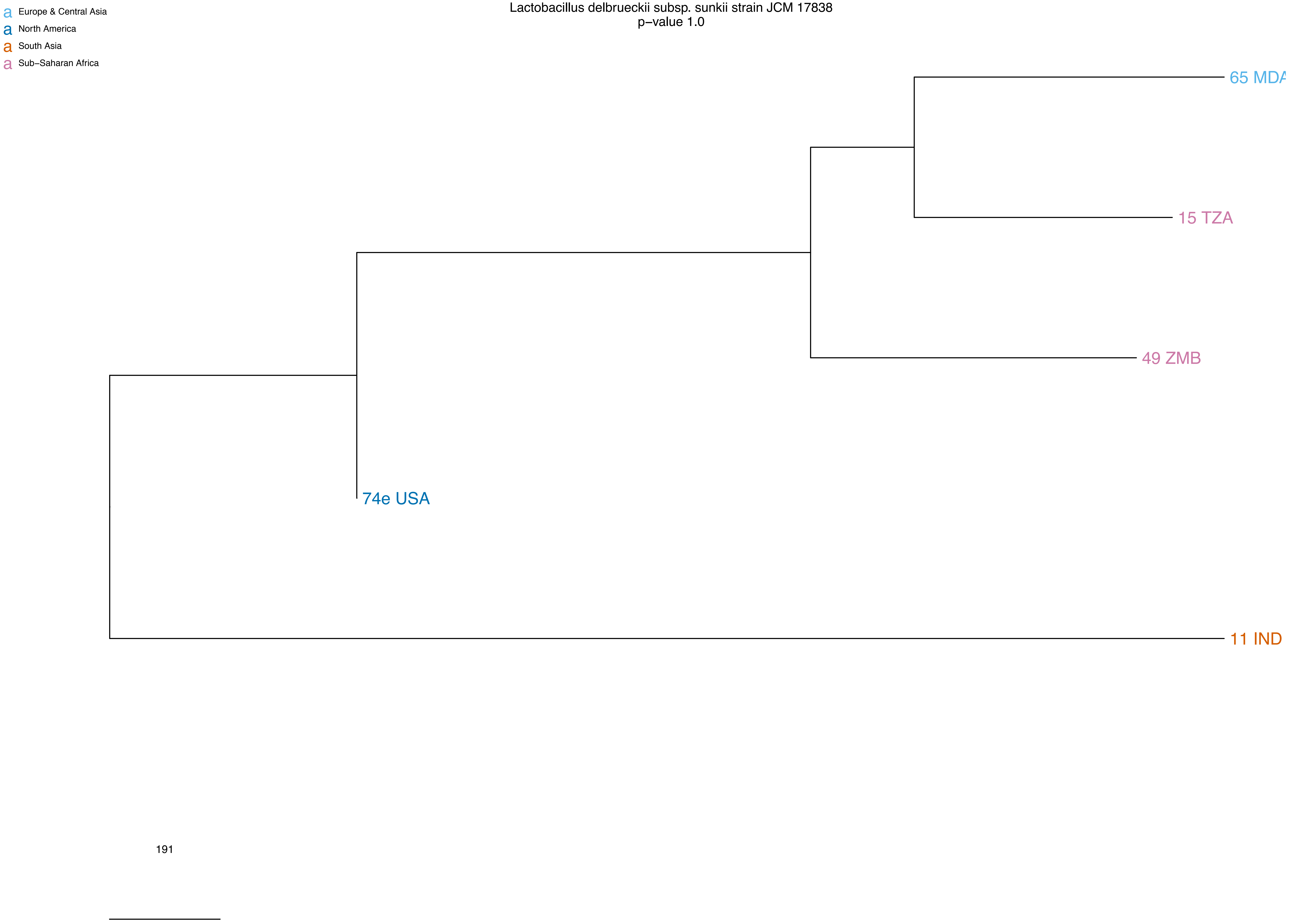

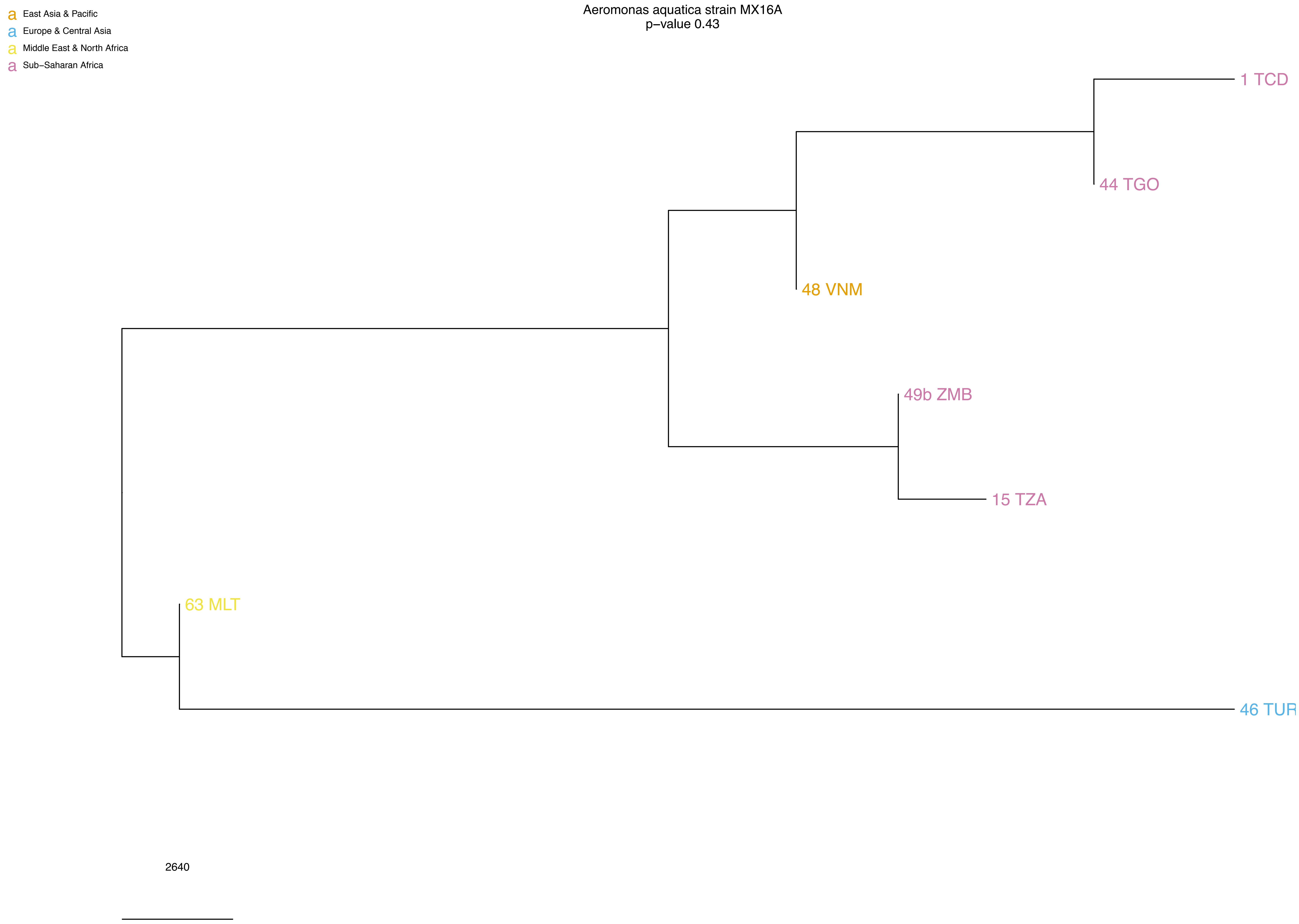

a East Asia & Pacific
a Europe & Central Asia
a Middle East & North Africa
a Sub–Saharan Africa
Aeromonas aquatica strain MX16A
p–value 0.43
1 TCD
44 TGO
48 VNM
49b ZMB
15 TZA
63 MLT
46 TUR
2640

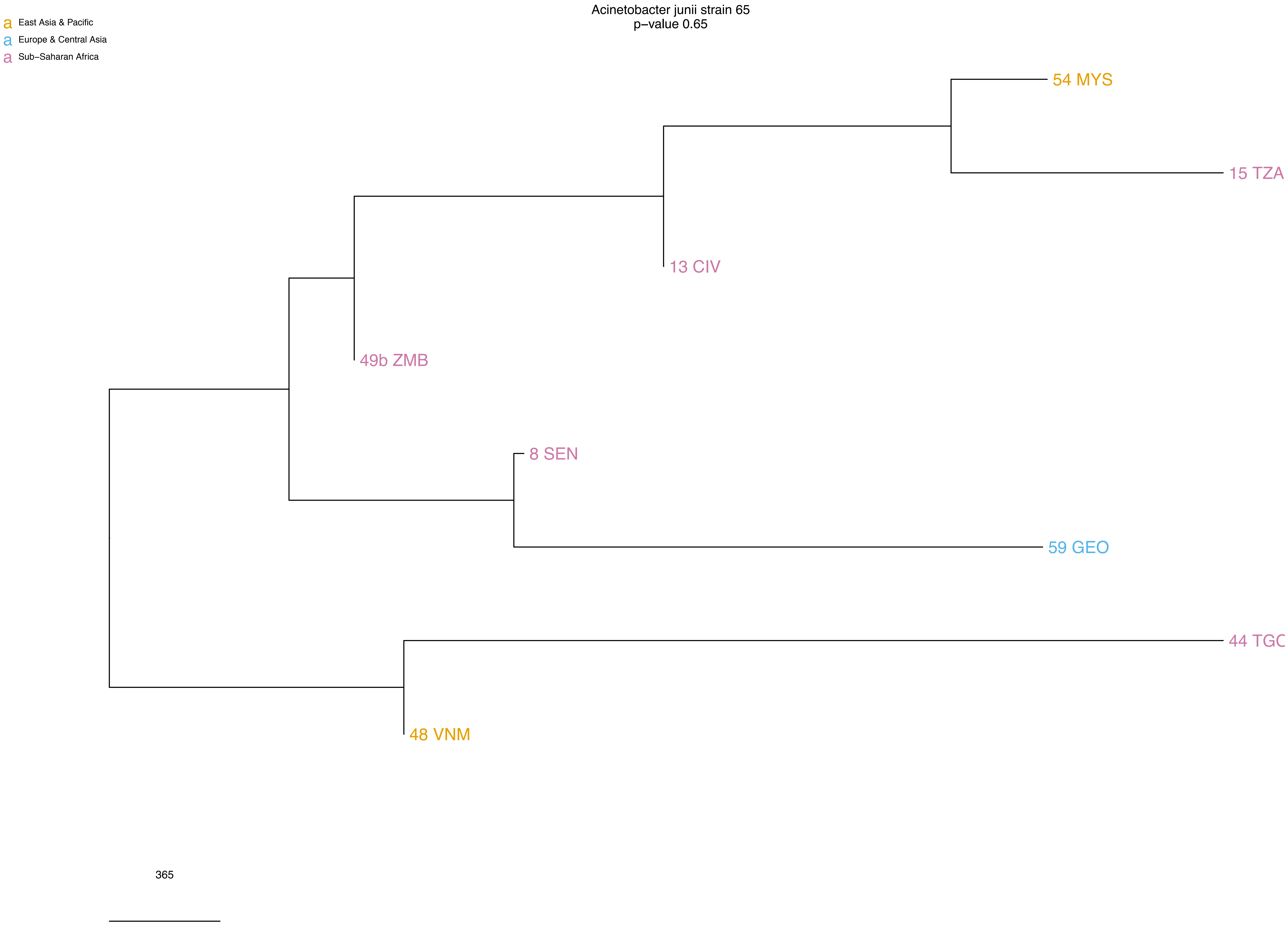

Acinetobacter junii strain 65
p–value 0.65
East Asia & Pacific
Europe & Central Asia
Sub–Saharan Africa
54 MYS
15 TZA
13 CIV
49b ZMB
8 SEN
59 GEO
44 TGO
48 VNM
365

Acinetobacter schindleri strain ACE
p–value 1.0

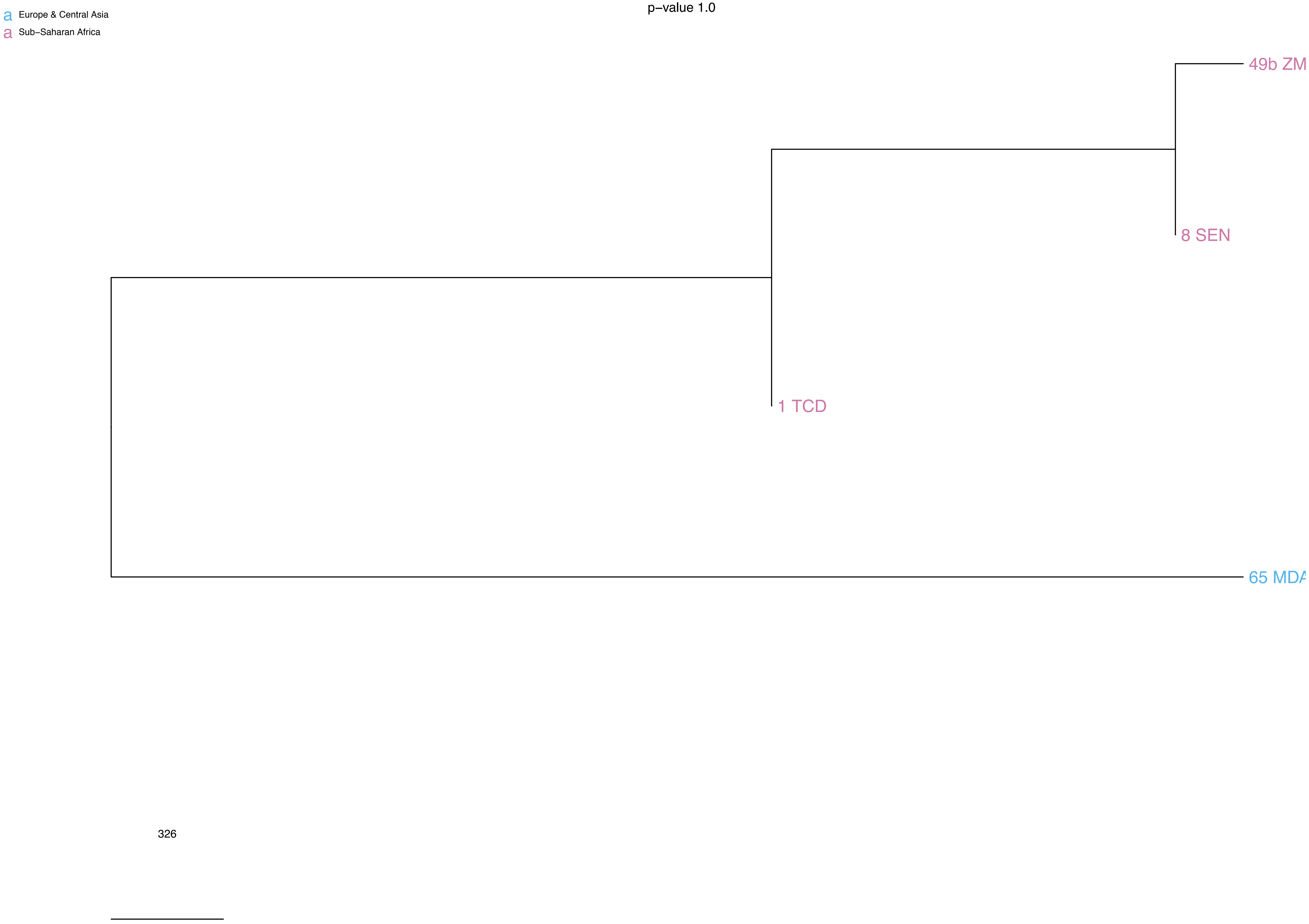

Supplement: Supplementary file 2 — Supplementary information2 [file 41598_2020_59292_MOESM2_ESM.pdf]
